# Supplementary material for: Mitochondrial protein import clogging as a mechanism of disease
Source: eLife. 2023 May 2;12:e84330. doi: 10.7554/eLife.84330 (PMC10208645; doi:10.7554/eLife.84330)
Supplement: Figure 1—source data 1. [file elife-84330-fig1-data1.zip › Figure 1-source data 1/Figure 1-source data_with annotation.pdf]

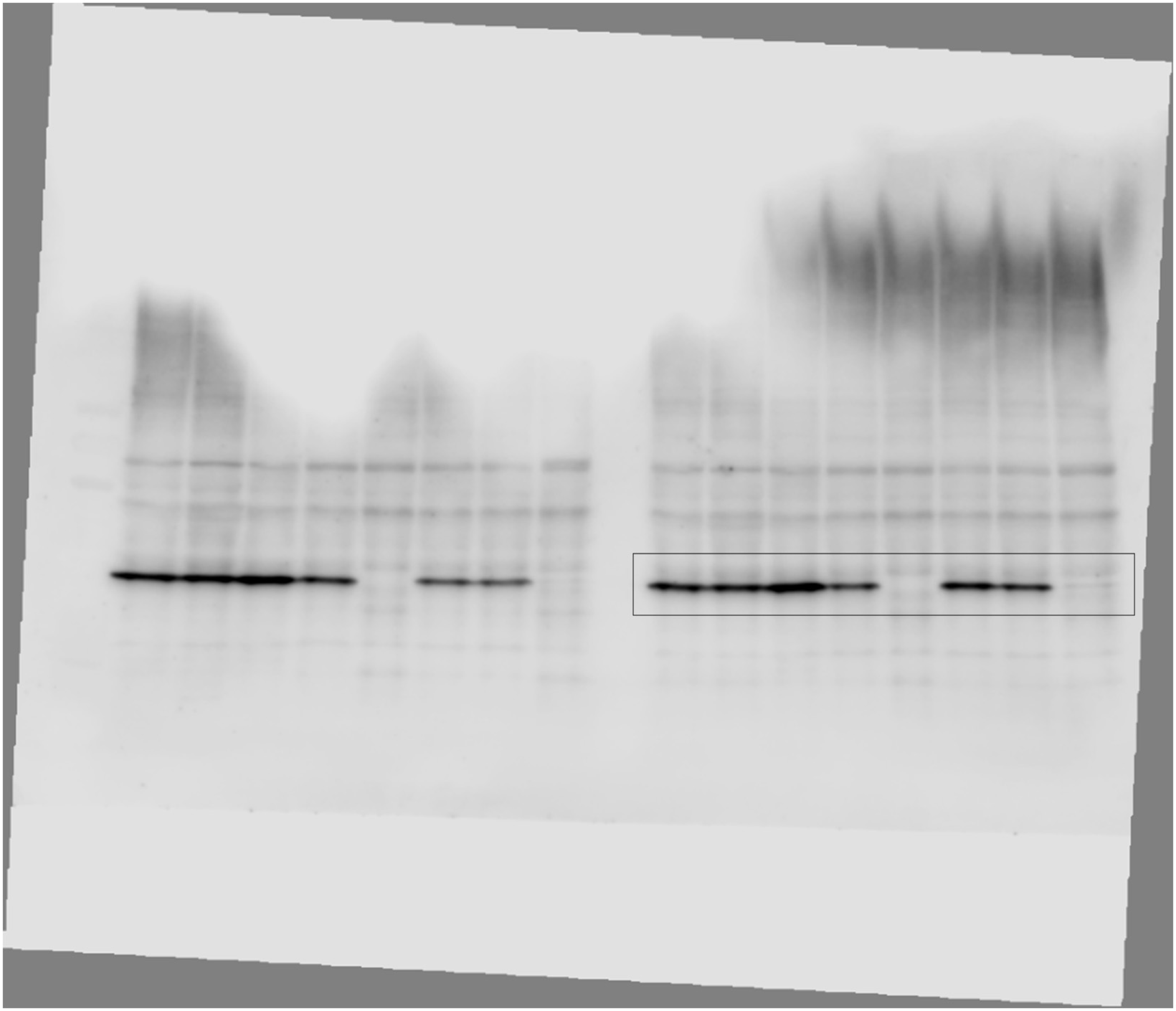

Cropped area for Figure 1E
Aac2 short

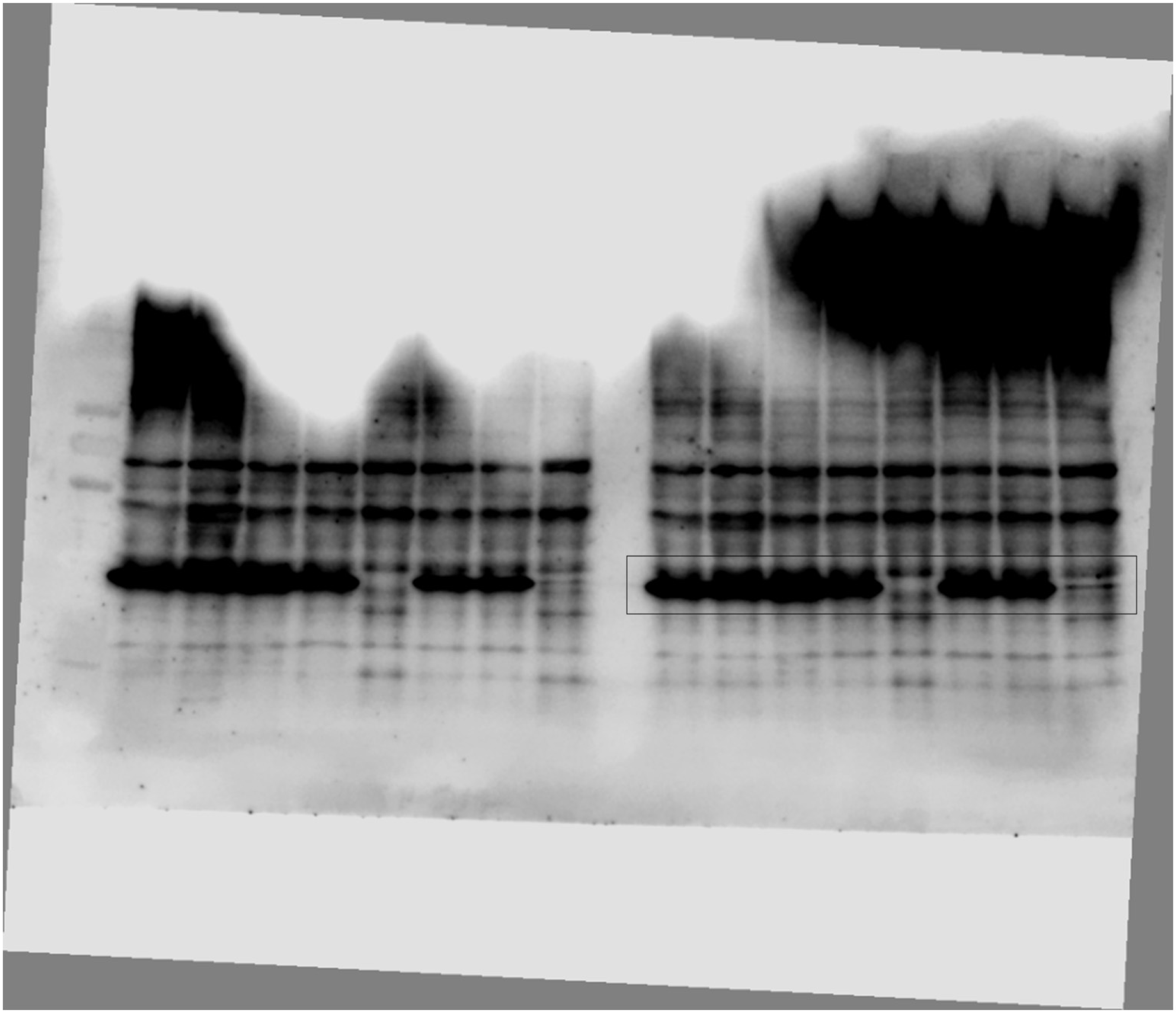

Cropped area for Figure 1E
Aac2 Long

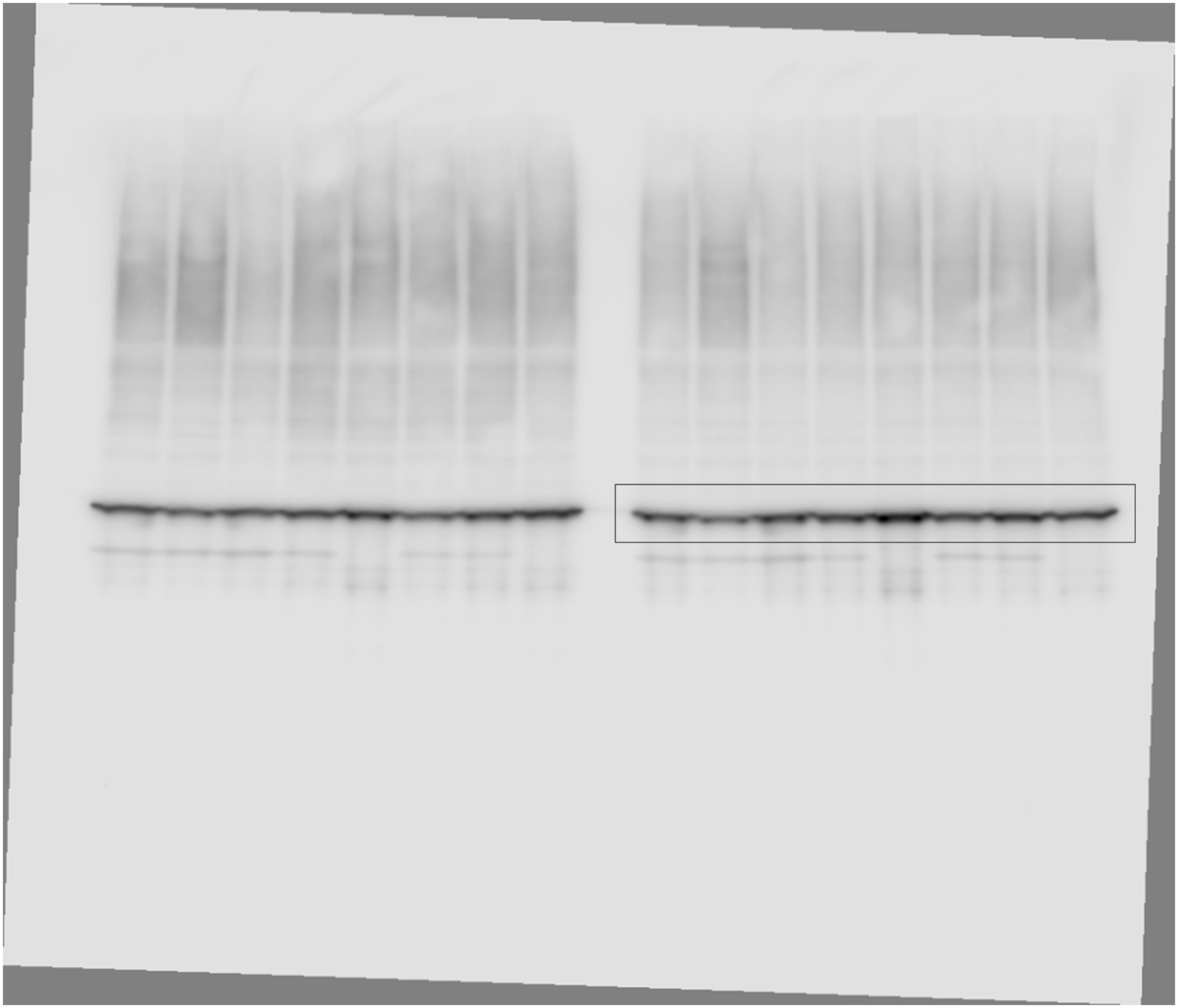

Cropped are for Figure1E
Ilv5

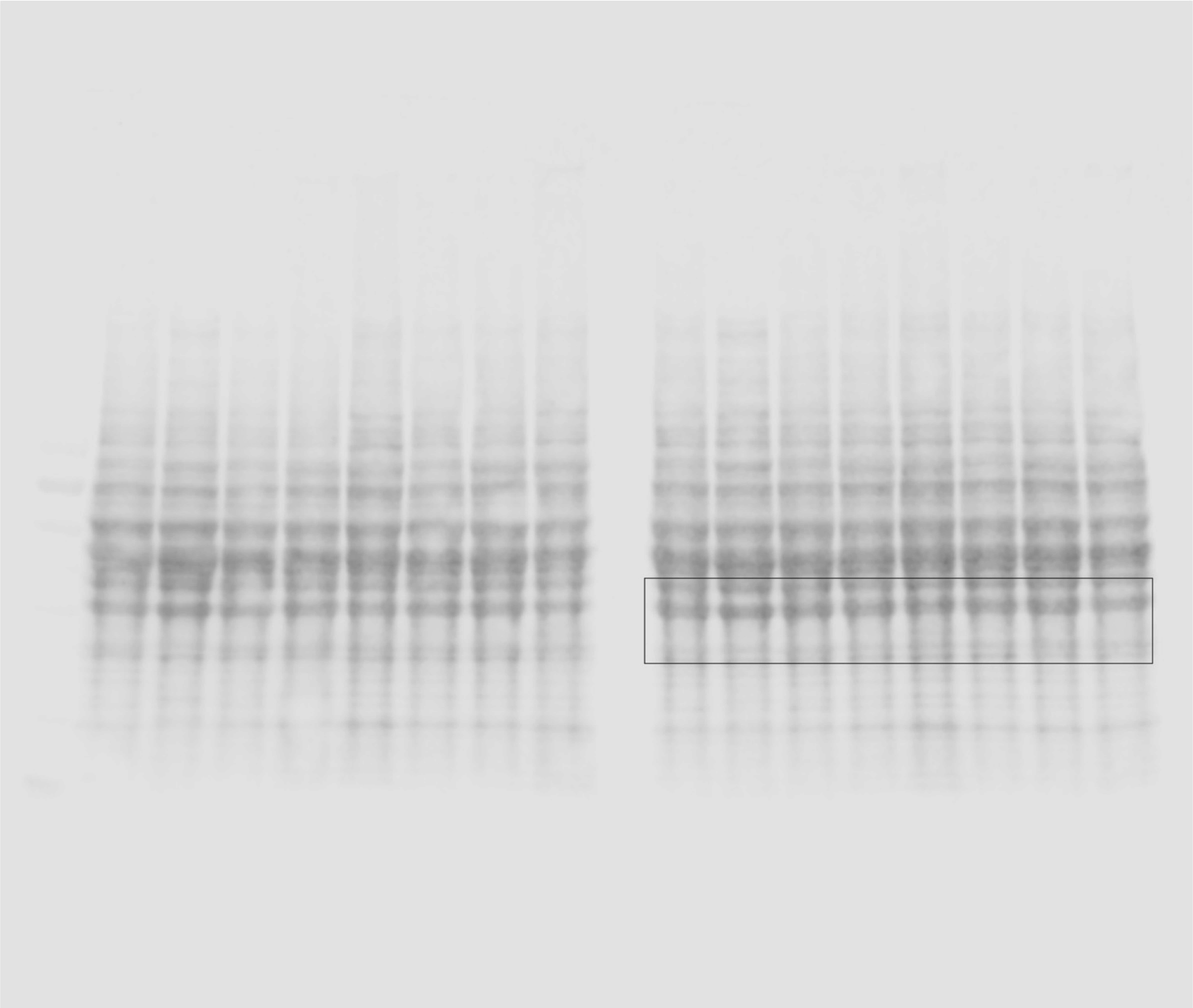

Cropped area for Figure 1E
Total Protein Stain
