# Supplementary figures and images for: Mitochondrial protein import clogging as a mechanism of disease
Source: eLife. 2023 May 2;12:e84330. doi: 10.7554/eLife.84330 (PMC10208645; doi:10.7554/eLife.84330)

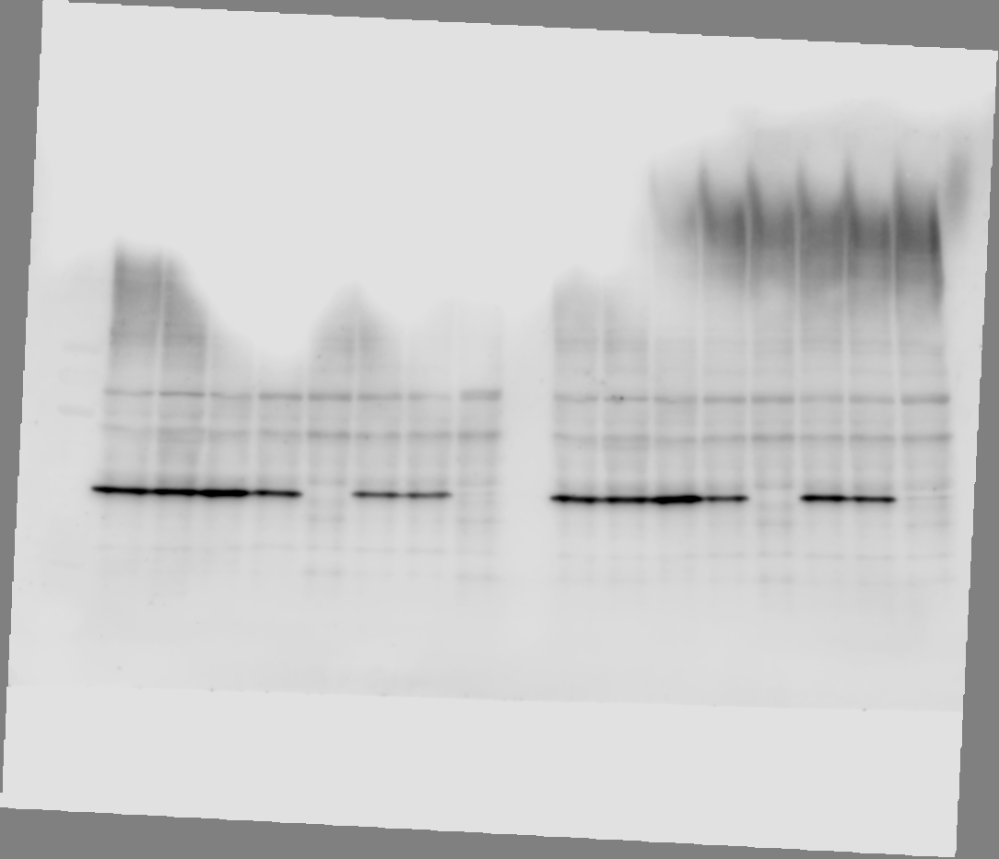

Supplement: Figure 1—source data 1. [file elife-84330-fig1-data1.zip › Figure 1-source data 1/1F-AAC2-Short.tif]

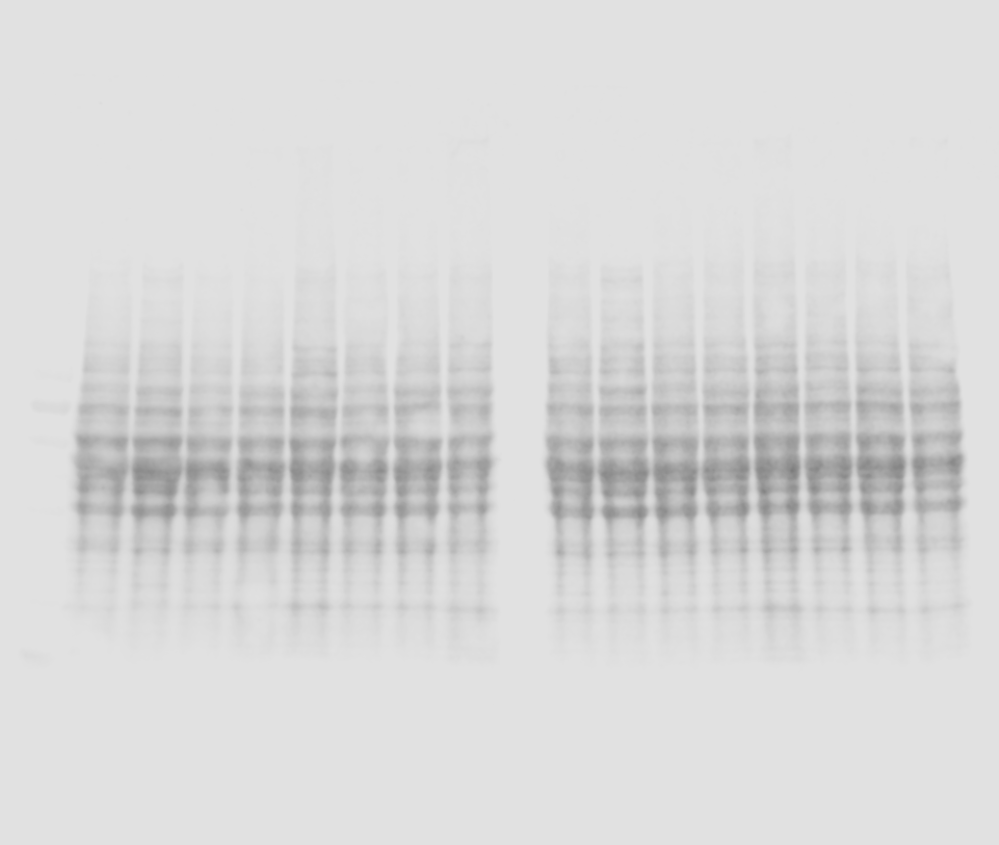

Supplement: Figure 1—source data 1. [file elife-84330-fig1-data1.zip › Figure 1-source data 1/1F-TPS.tif]

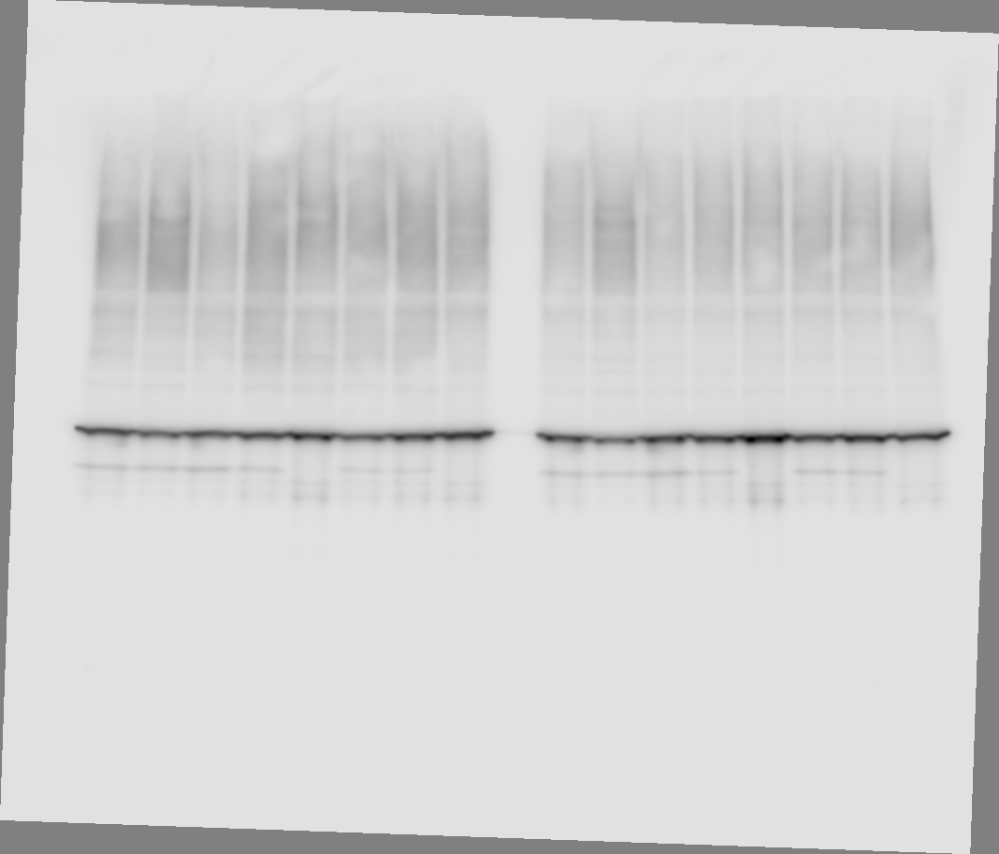

Supplement: Figure 1—source data 1. [file elife-84330-fig1-data1.zip › Figure 1-source data 1/1F-ILV5.tif]

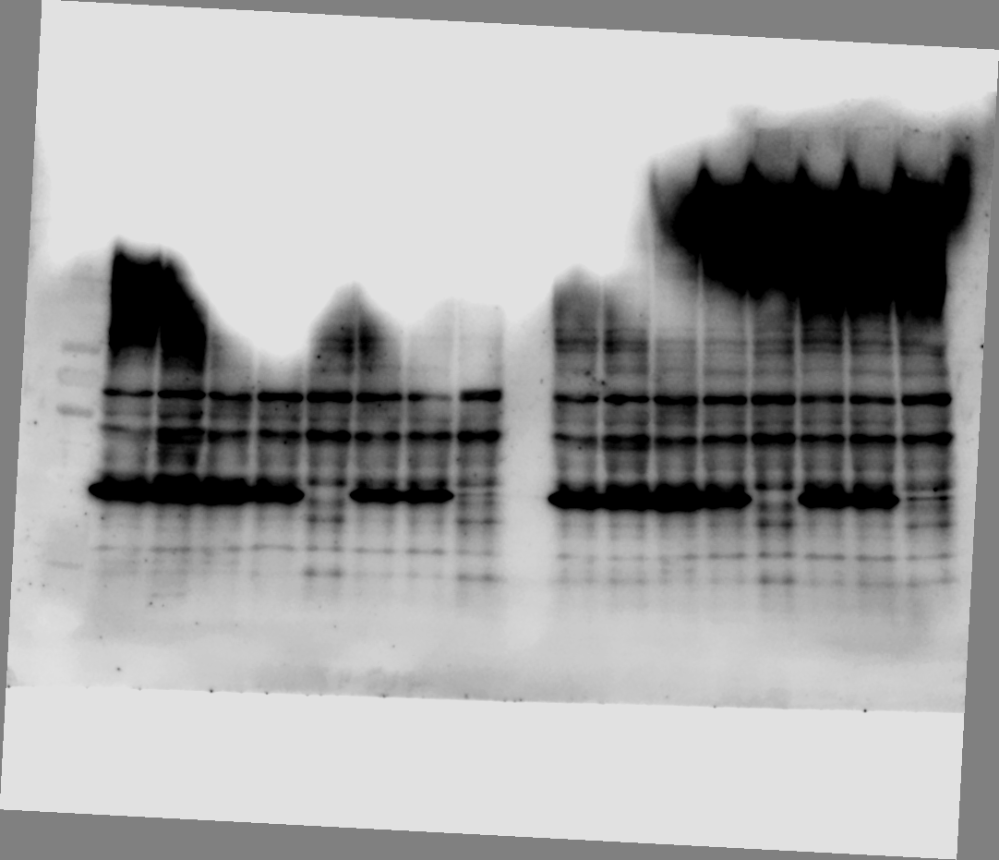

Supplement: Figure 1—source data 1. [file elife-84330-fig1-data1.zip › Figure 1-source data 1/1F-AAC2 long.tif]

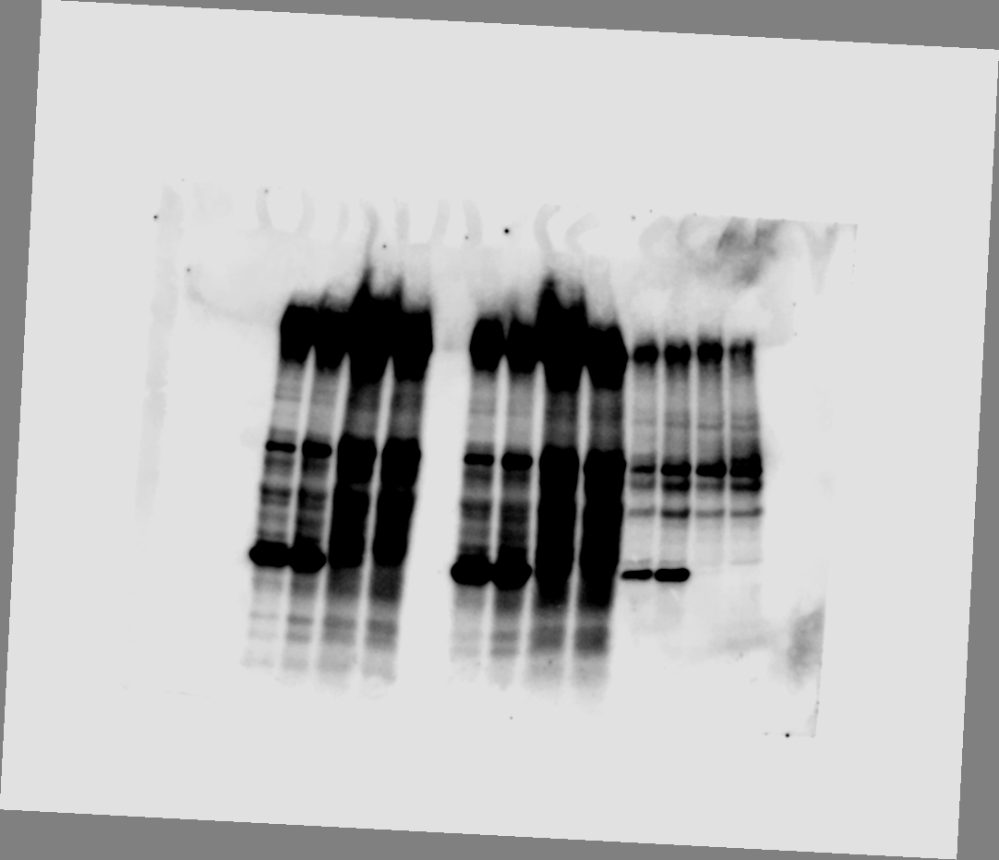

Supplement: Figure 1—figure supplement 1—source data 1. [file elife-84330-fig1-figsupp1-data1.zip › Figure 1-figure supplement 1/F1-fs1C-AAC2 right.tif]

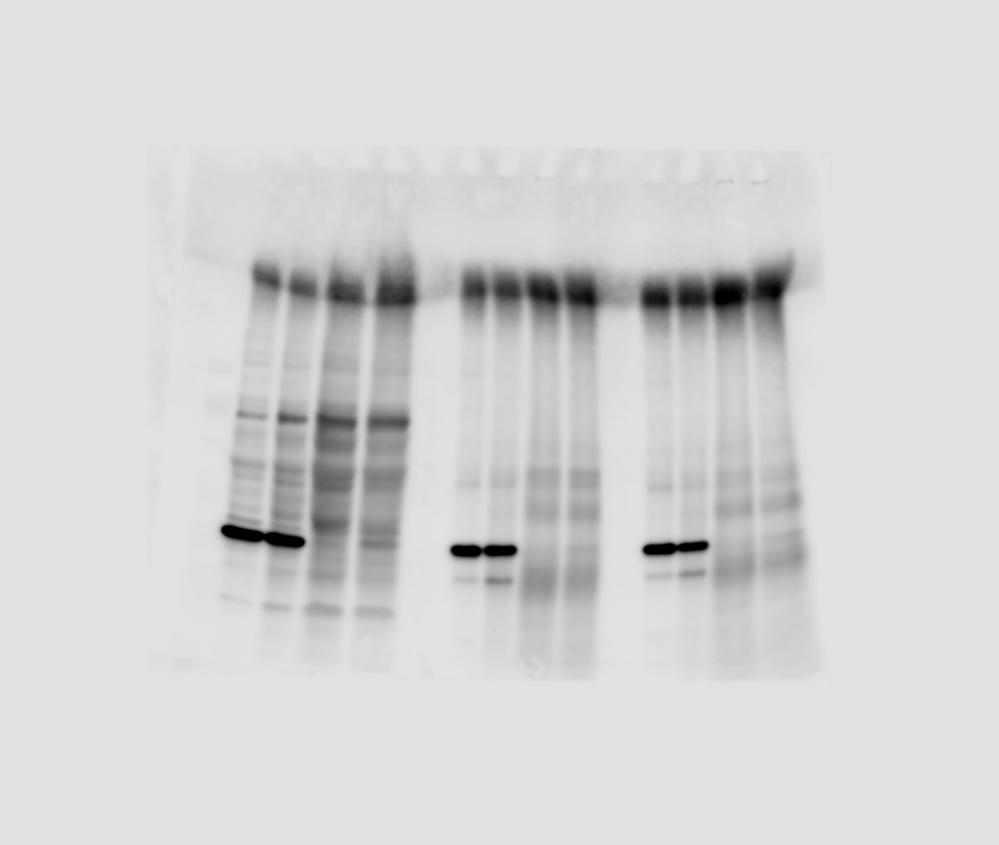

Supplement: Figure 1—figure supplement 1—source data 1. [file elife-84330-fig1-figsupp1-data1.zip › Figure 1-figure supplement 1/F1-fs1B.tif]

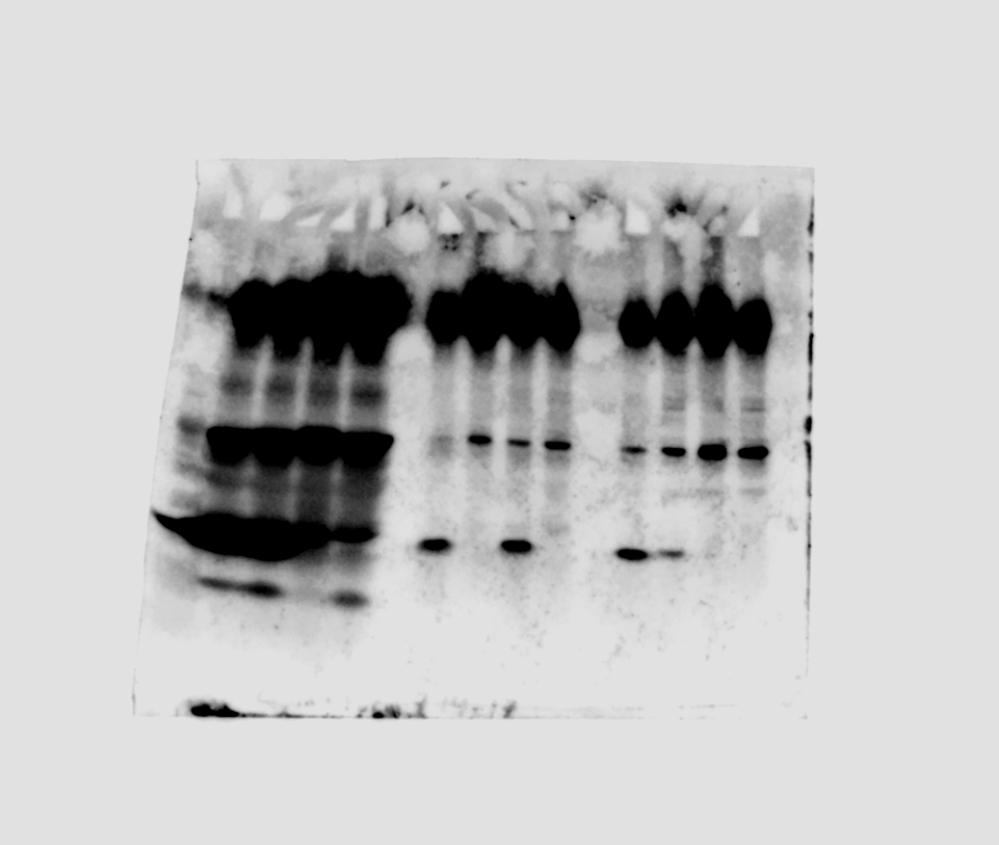

Supplement: Figure 1—figure supplement 1—source data 1. [file elife-84330-fig1-figsupp1-data1.zip › Figure 1-figure supplement 1/F1-fs1C-AAC2 Left.tif]

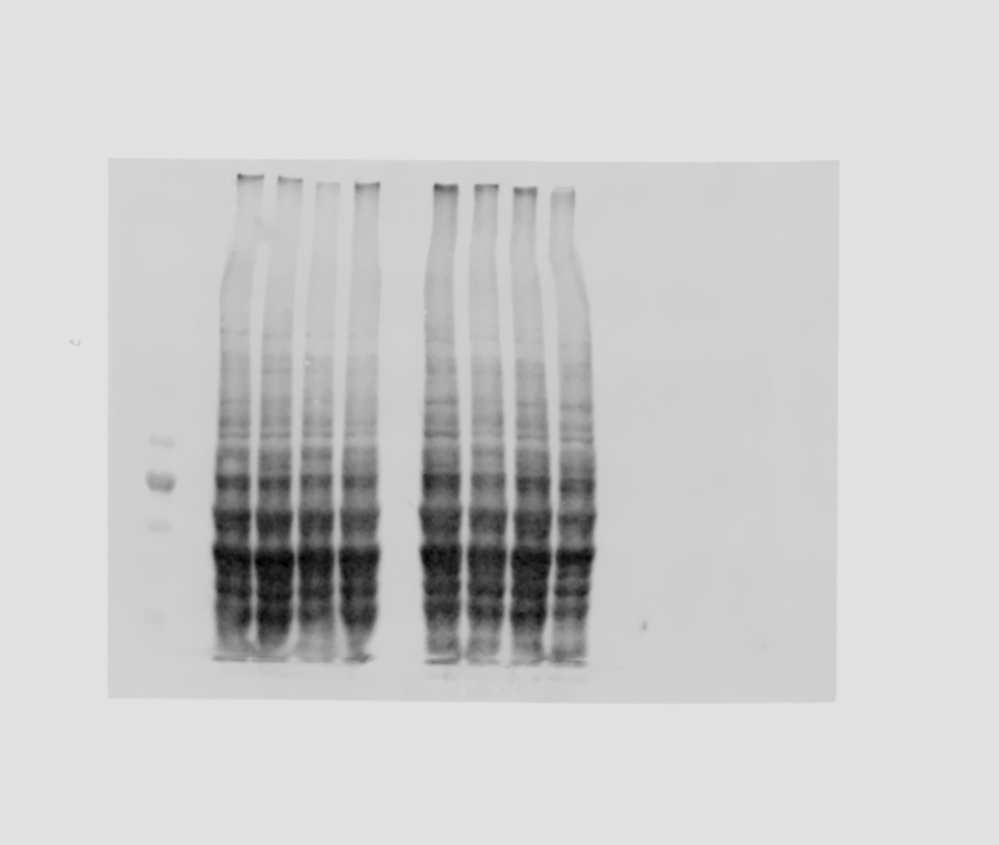

Supplement: Figure 2—source data 1. [file elife-84330-fig2-data1.zip › Figure 2-source data 1/2G TPS.tif]

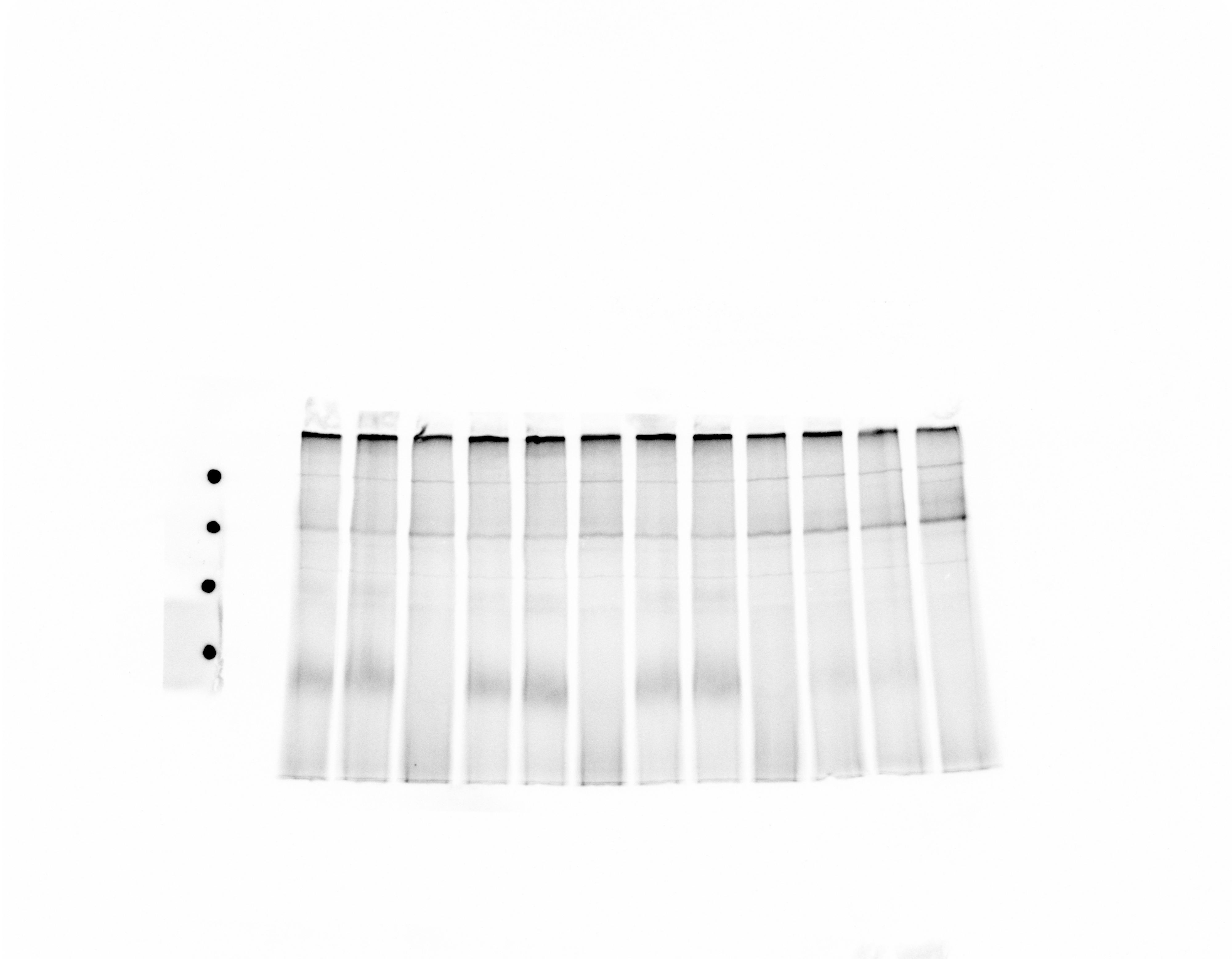

Supplement: Figure 2—source data 1. [file elife-84330-fig2-data1.zip › Figure 2-source data 1/Fig.2A.tif]

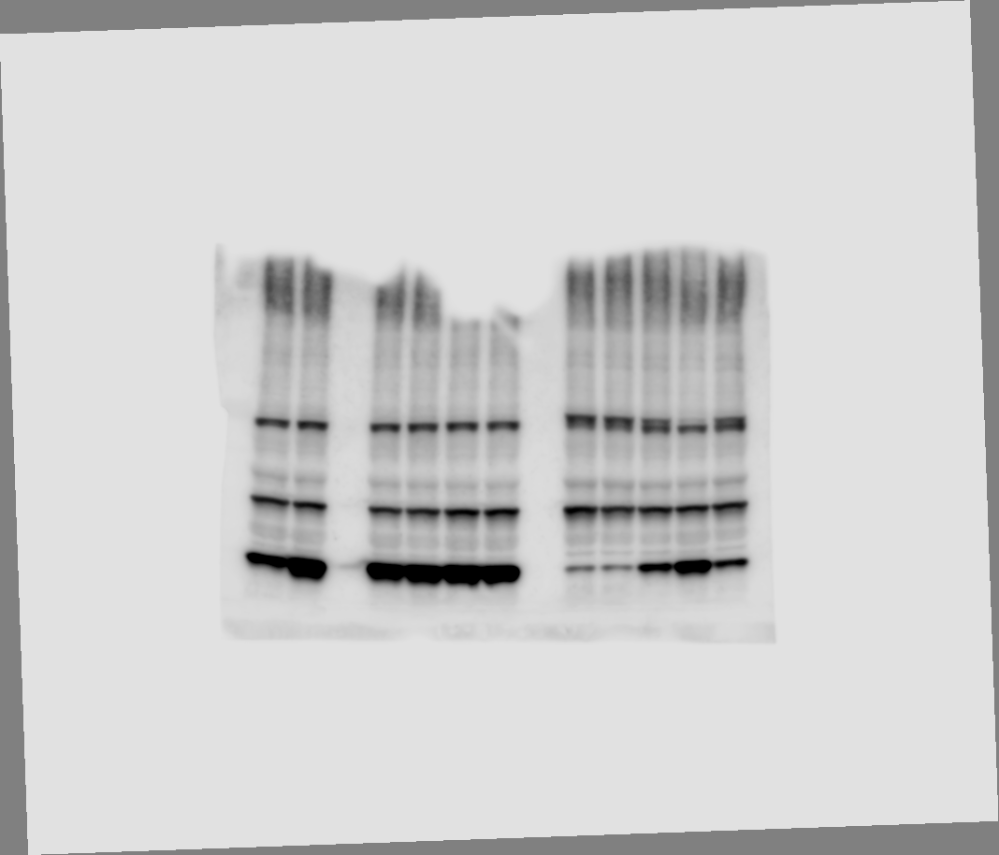

Supplement: Figure 2—source data 1. [file elife-84330-fig2-data1.zip › Figure 2-source data 1/2H Ilv5.tif]

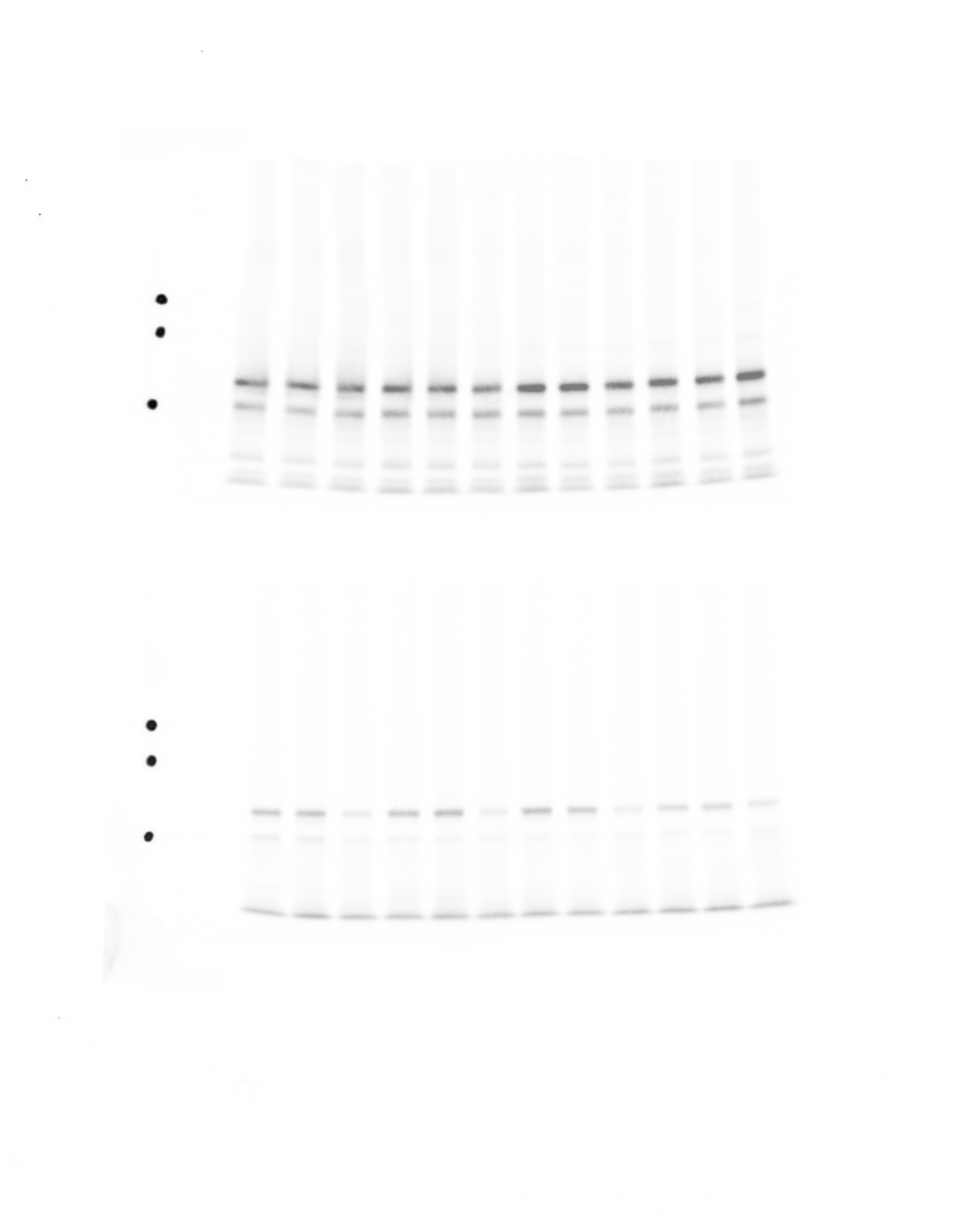

Supplement: Figure 2—source data 1. [file elife-84330-fig2-data1.zip › Figure 2-source data 1/Fig.2C.tif]

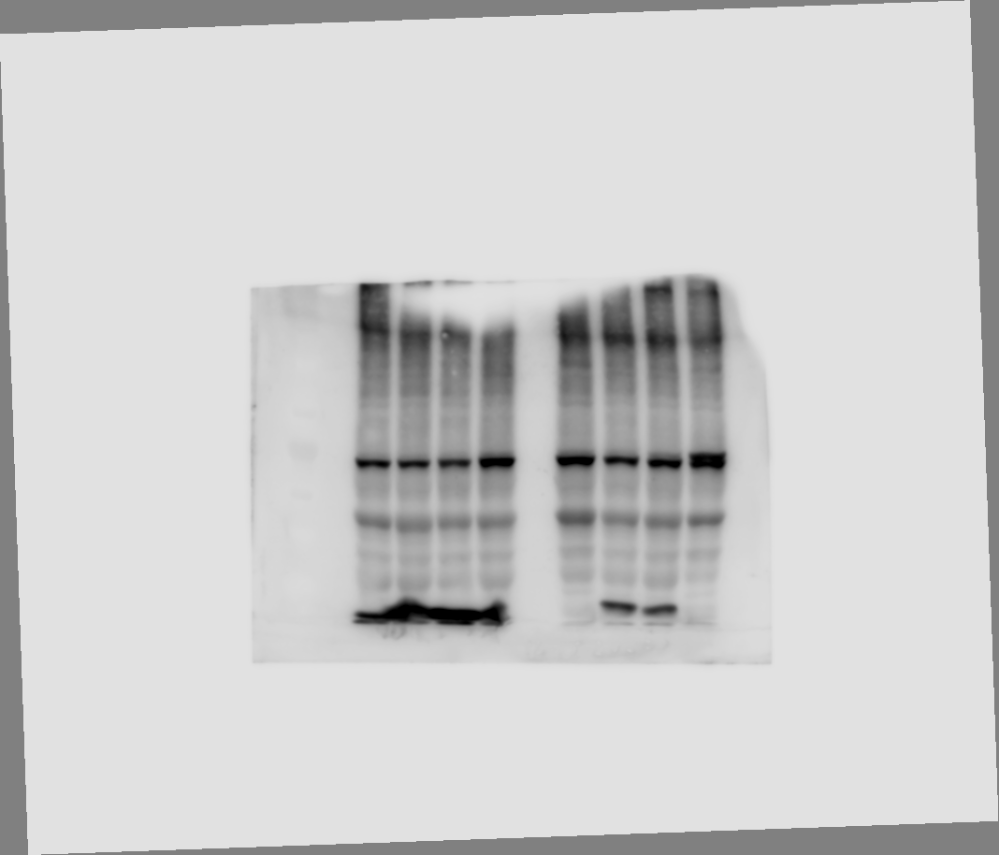

Supplement: Figure 2—source data 1. [file elife-84330-fig2-data1.zip › Figure 2-source data 1/2G Aac2.tif]

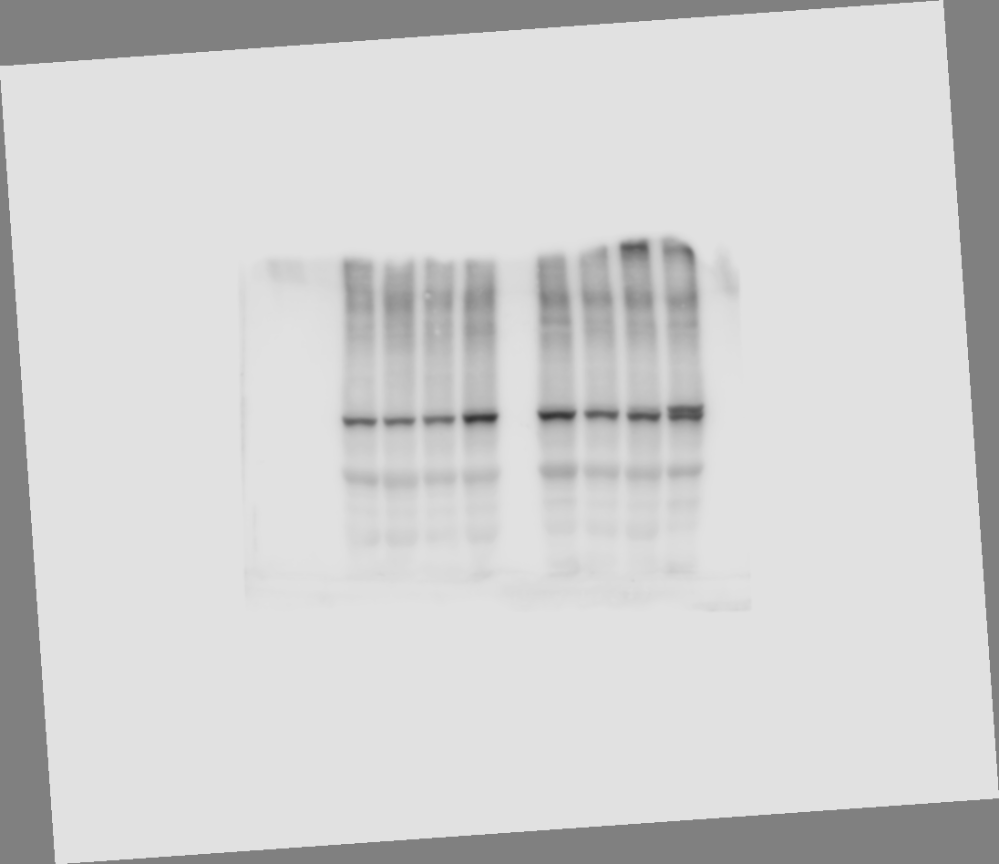

Supplement: Figure 2—source data 1. [file elife-84330-fig2-data1.zip › Figure 2-source data 1/2G HSP60.tif]

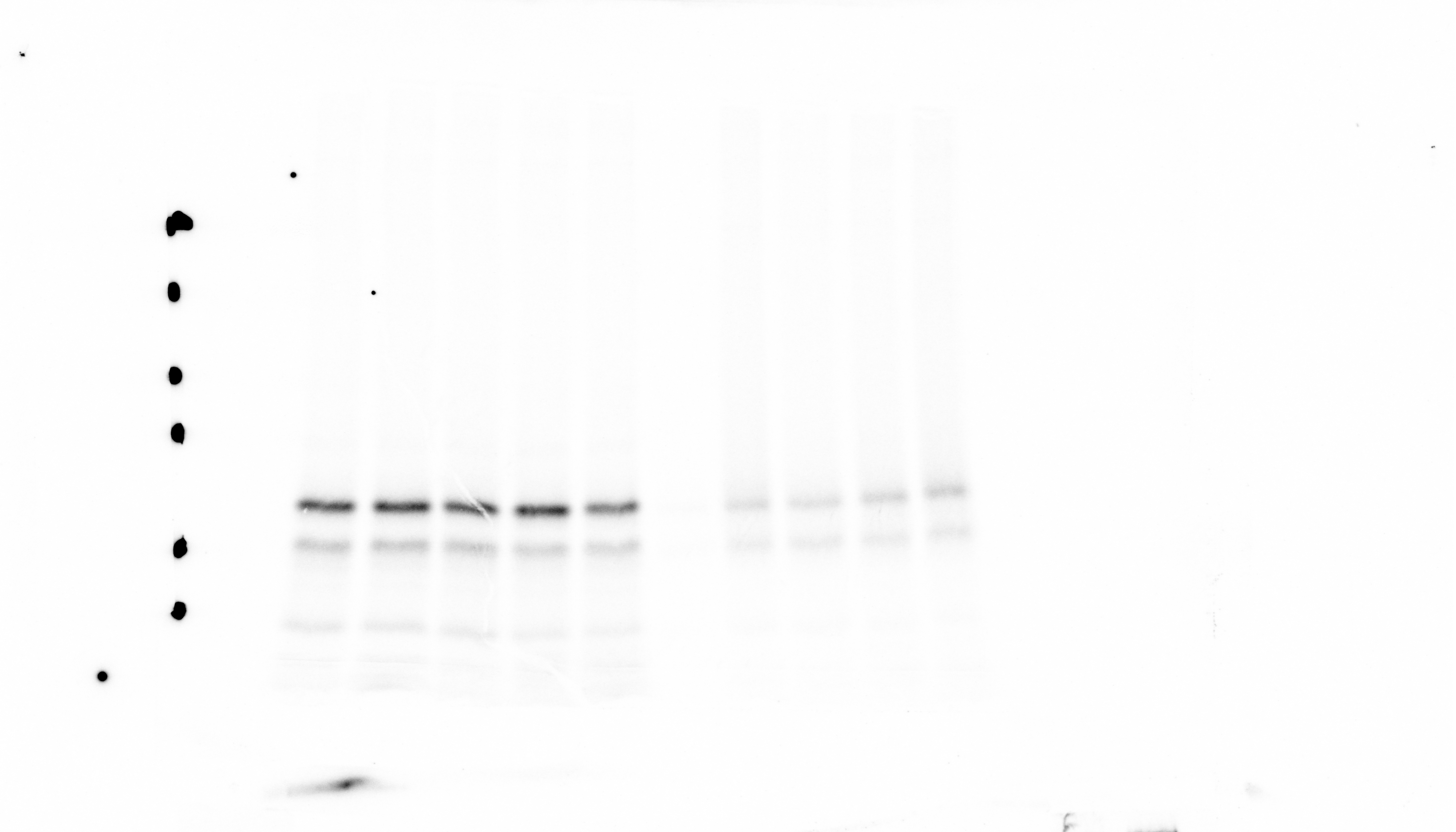

Supplement: Figure 2—source data 1. [file elife-84330-fig2-data1.zip › Figure 2-source data 1/Fig.2E.tif]

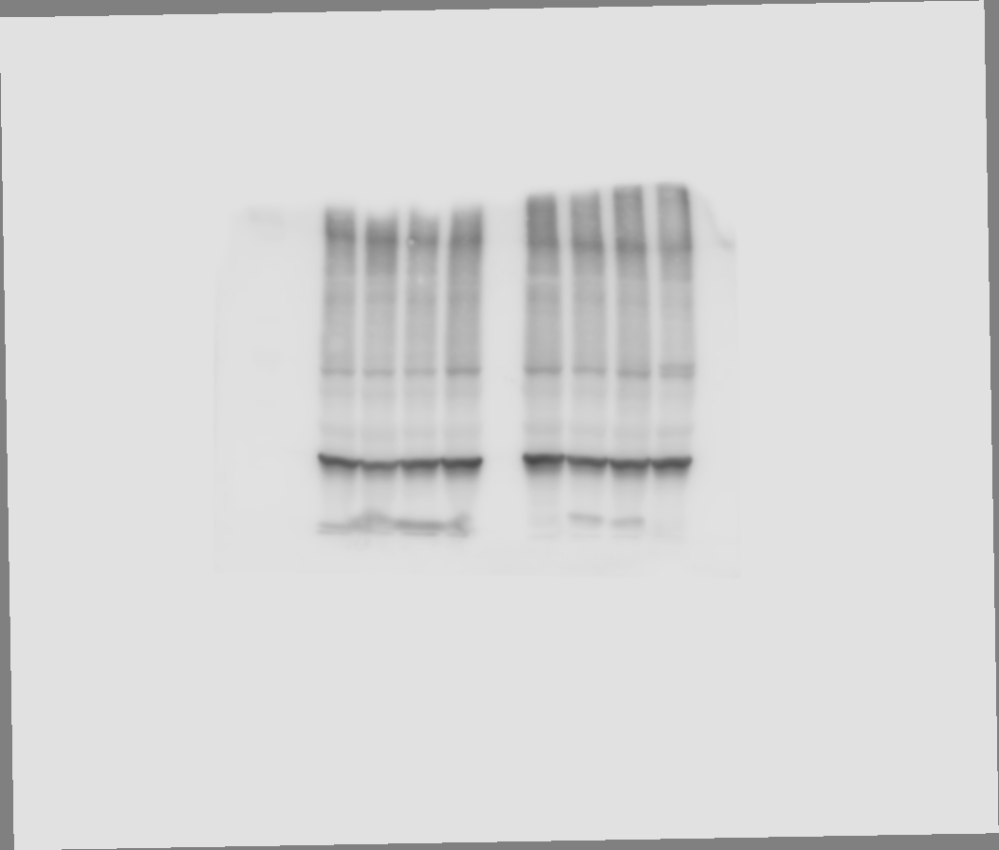

Supplement: Figure 2—source data 1. [file elife-84330-fig2-data1.zip › Figure 2-source data 1/2G Ilv5.tif]

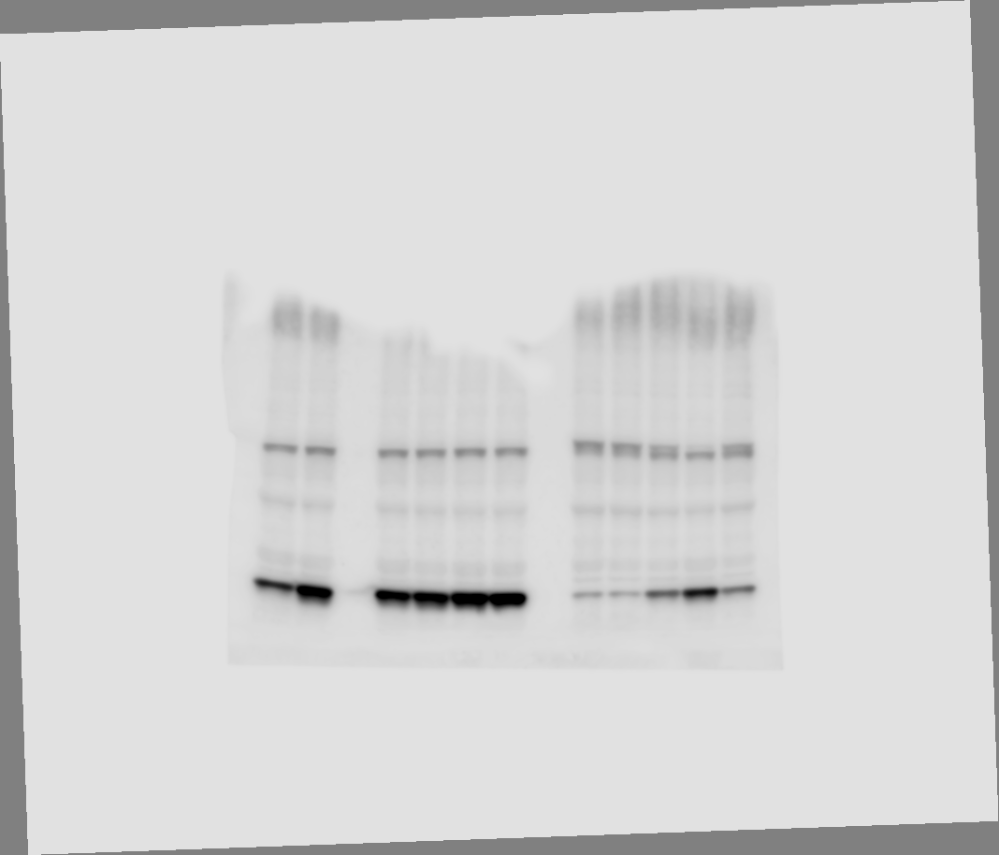

Supplement: Figure 2—source data 1. [file elife-84330-fig2-data1.zip › Figure 2-source data 1/2H Aac2.tif]

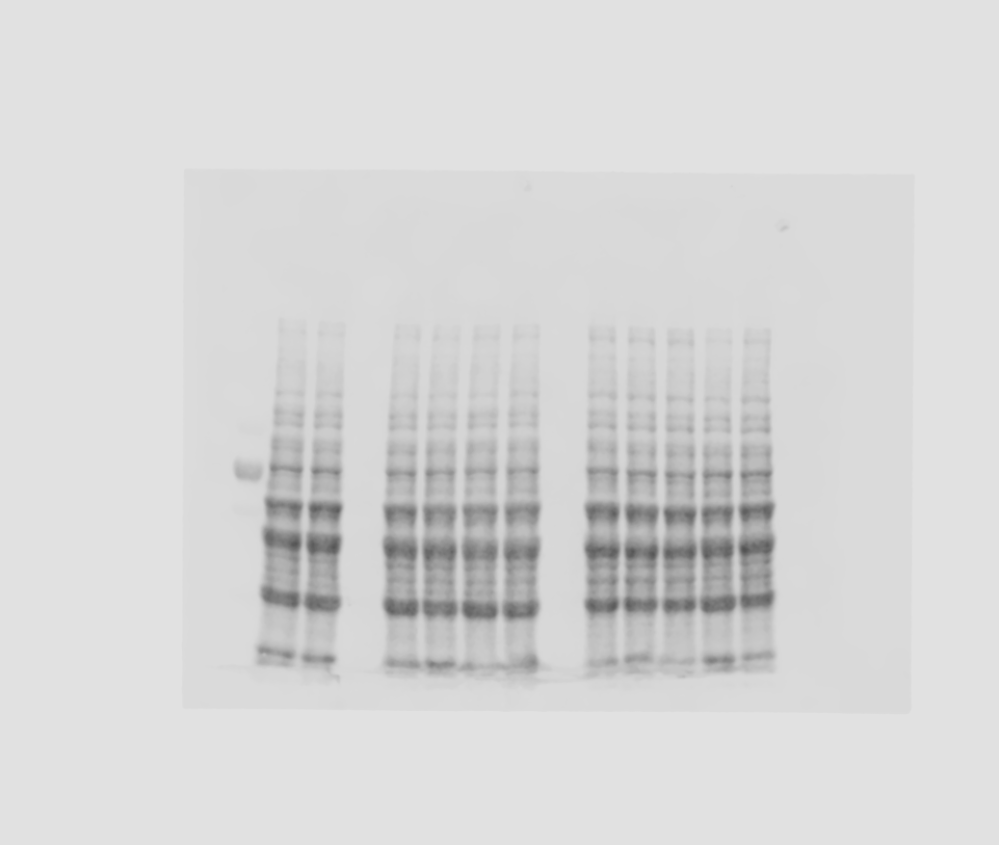

Supplement: Figure 2—source data 1. [file elife-84330-fig2-data1.zip › Figure 2-source data 1/2H TPS.tif]

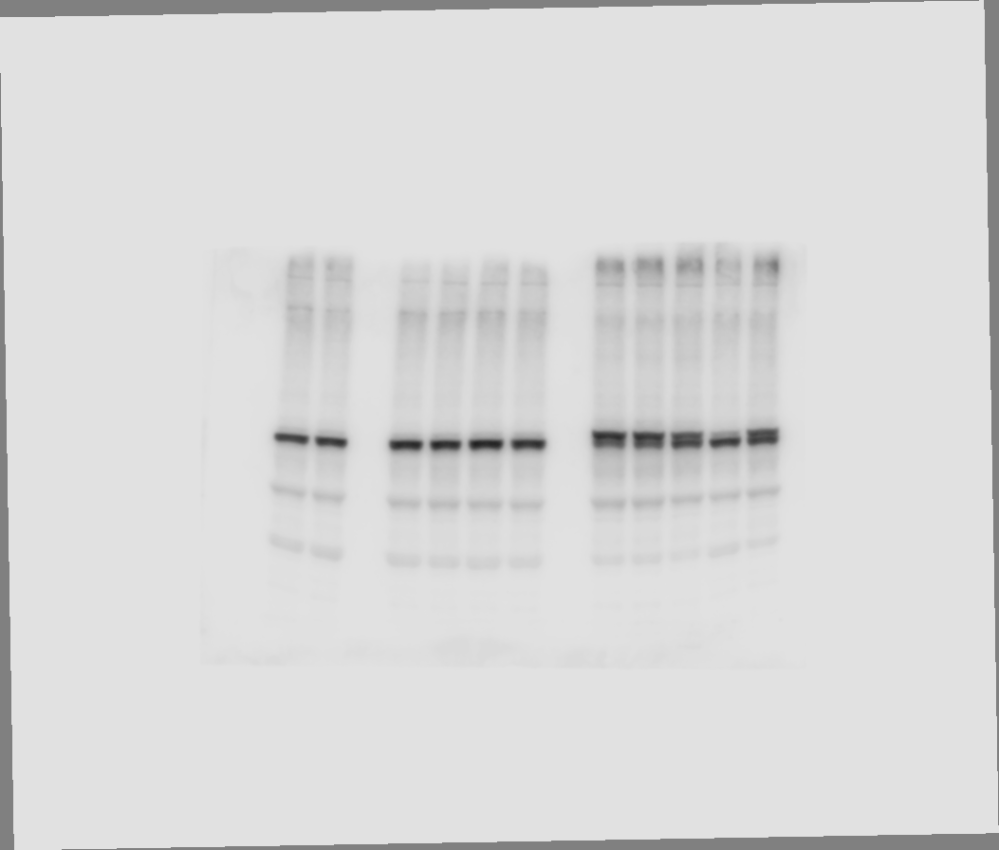

Supplement: Figure 2—source data 1. [file elife-84330-fig2-data1.zip › Figure 2-source data 1/2H Hsp60.tif]

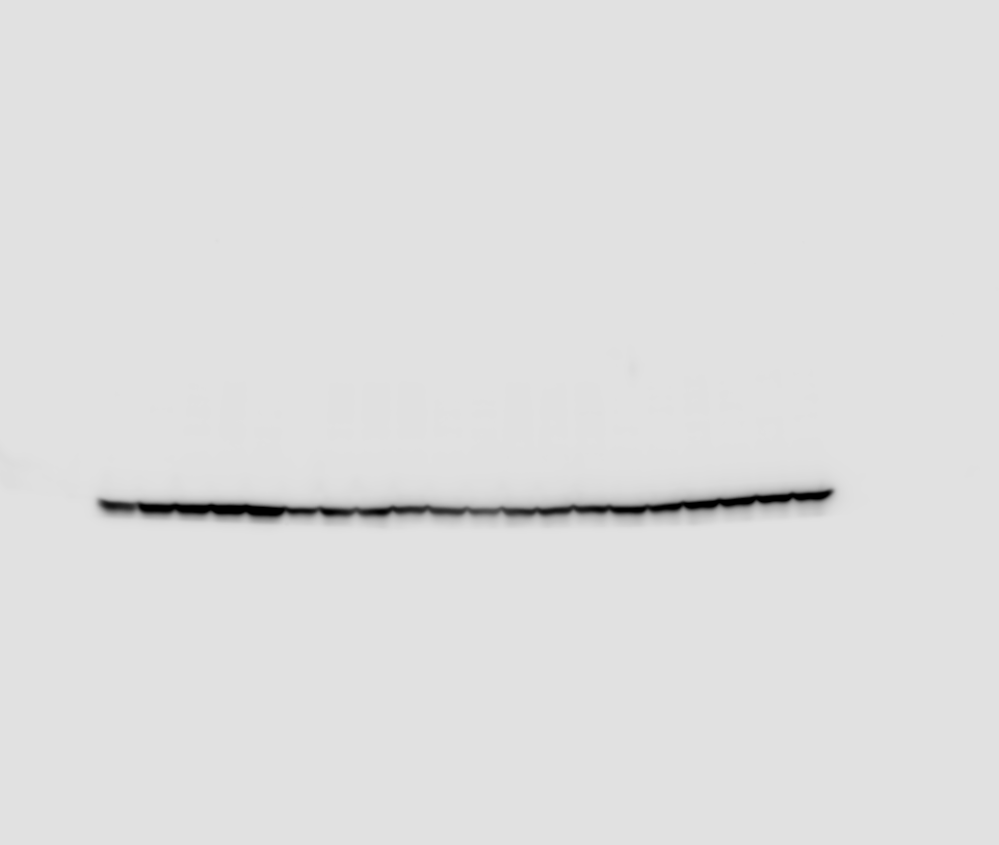

Supplement: Figure 2—figure supplement 1—source data 1. [file elife-84330-fig2-figsupp1-data1.zip › Figure 2-figure supplement 1-source data 1/Fig2-fs1C_Pgk1.tiff]

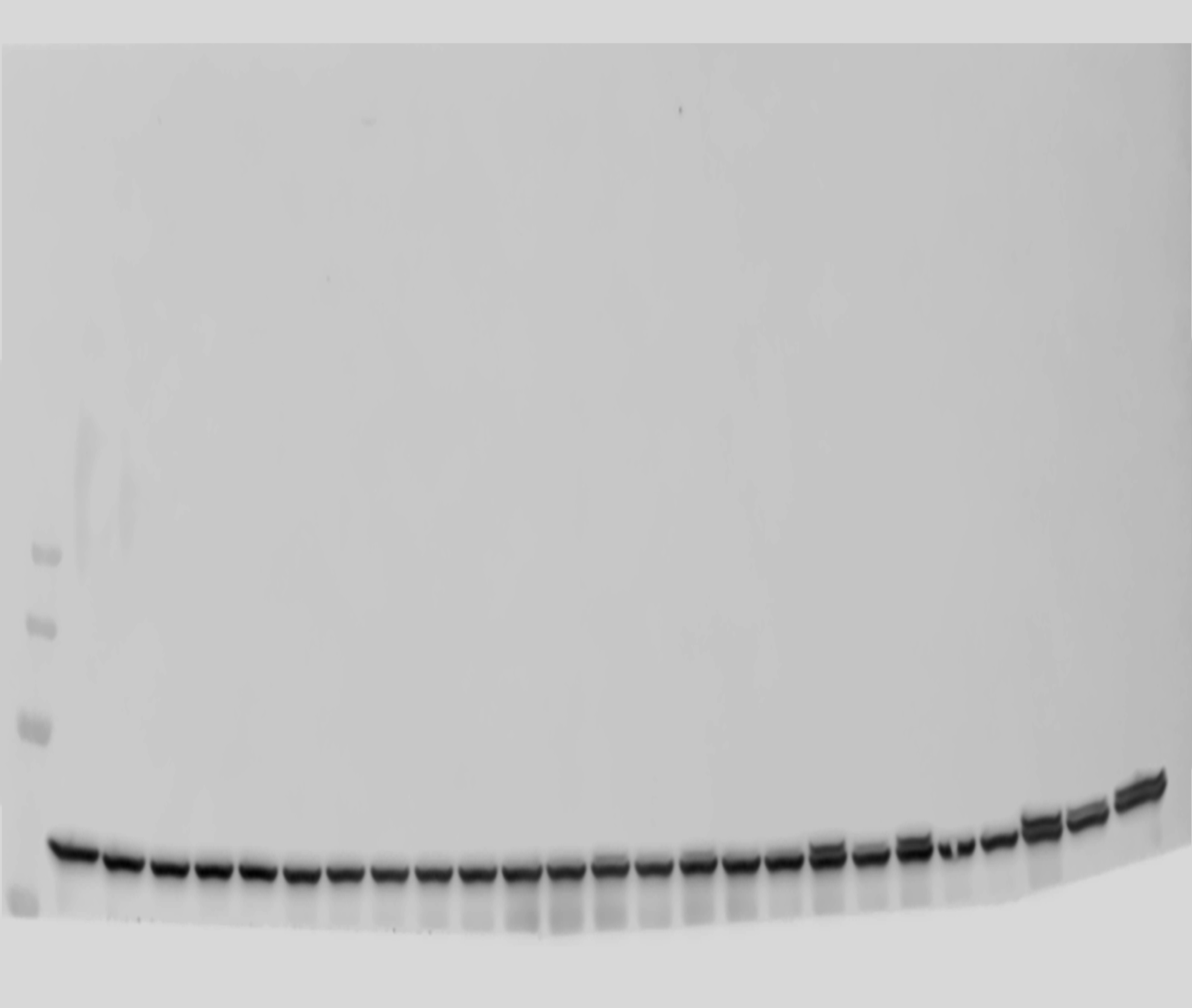

Supplement: Figure 2—figure supplement 1—source data 1. [file elife-84330-fig2-figsupp1-data1.zip › Figure 2-figure supplement 1-source data 1/Fig2-fs1B_Hsp60.tif]

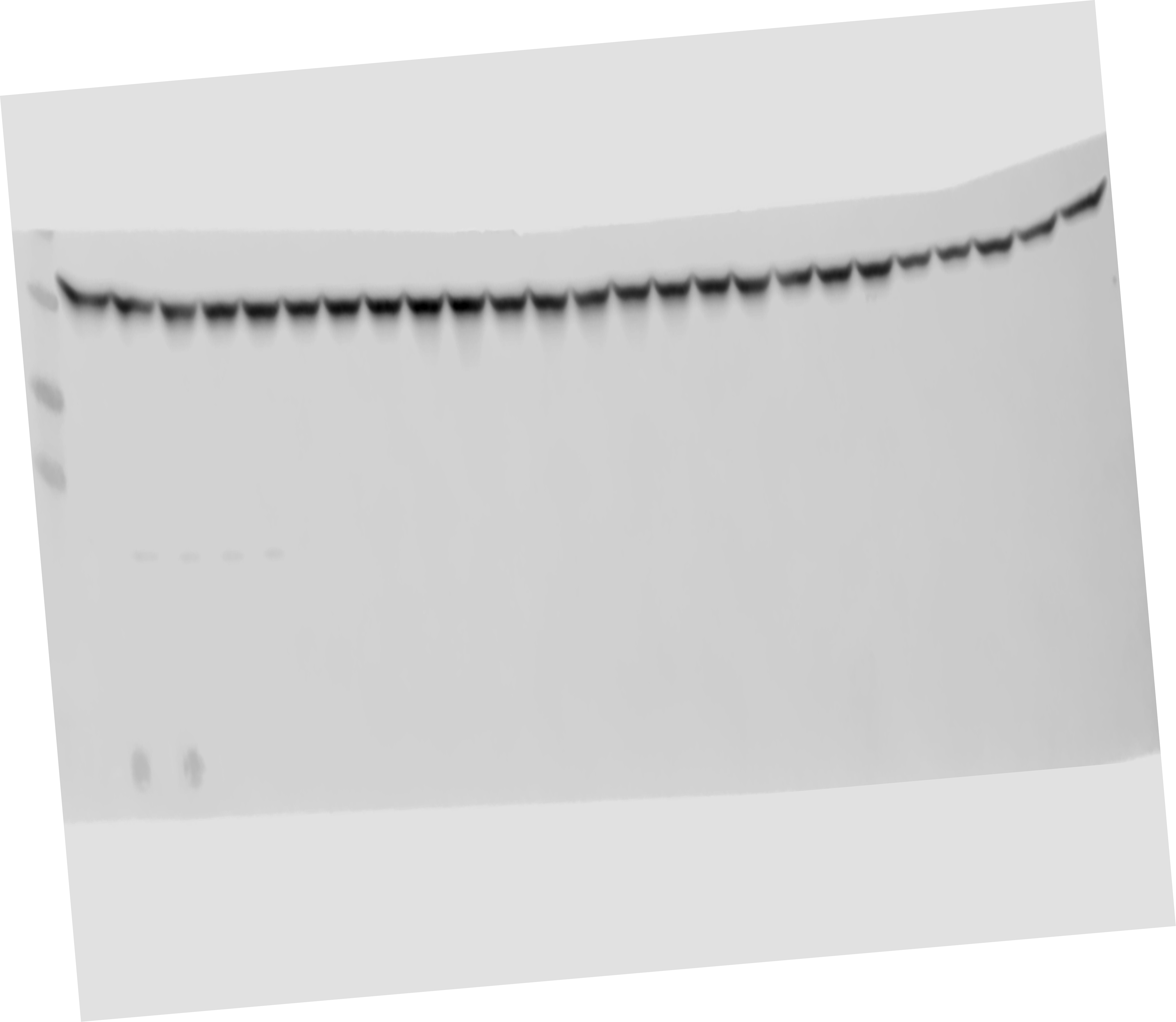

Supplement: Figure 2—figure supplement 1—source data 1. [file elife-84330-fig2-figsupp1-data1.zip › Figure 2-figure supplement 1-source data 1/Fig2-fs1B_Pgk1.tif]

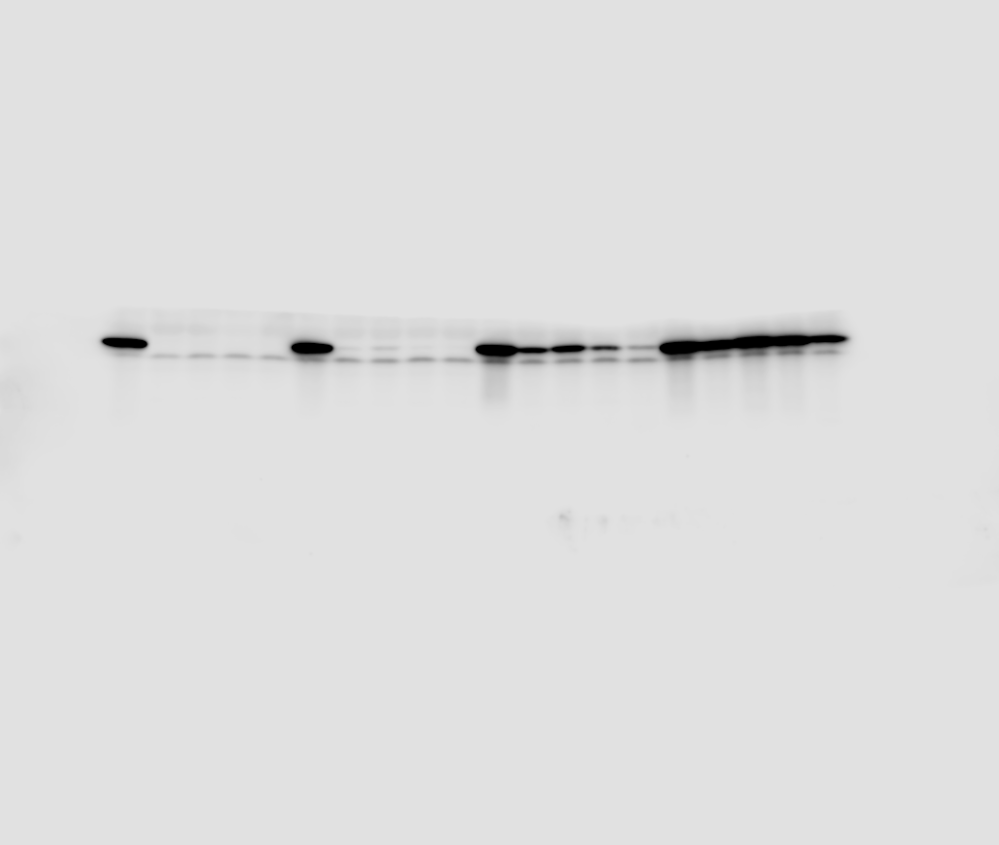

Supplement: Figure 2—figure supplement 1—source data 1. [file elife-84330-fig2-figsupp1-data1.zip › Figure 2-figure supplement 1-source data 1/Fig2-fs1C_Aac2.tiff]

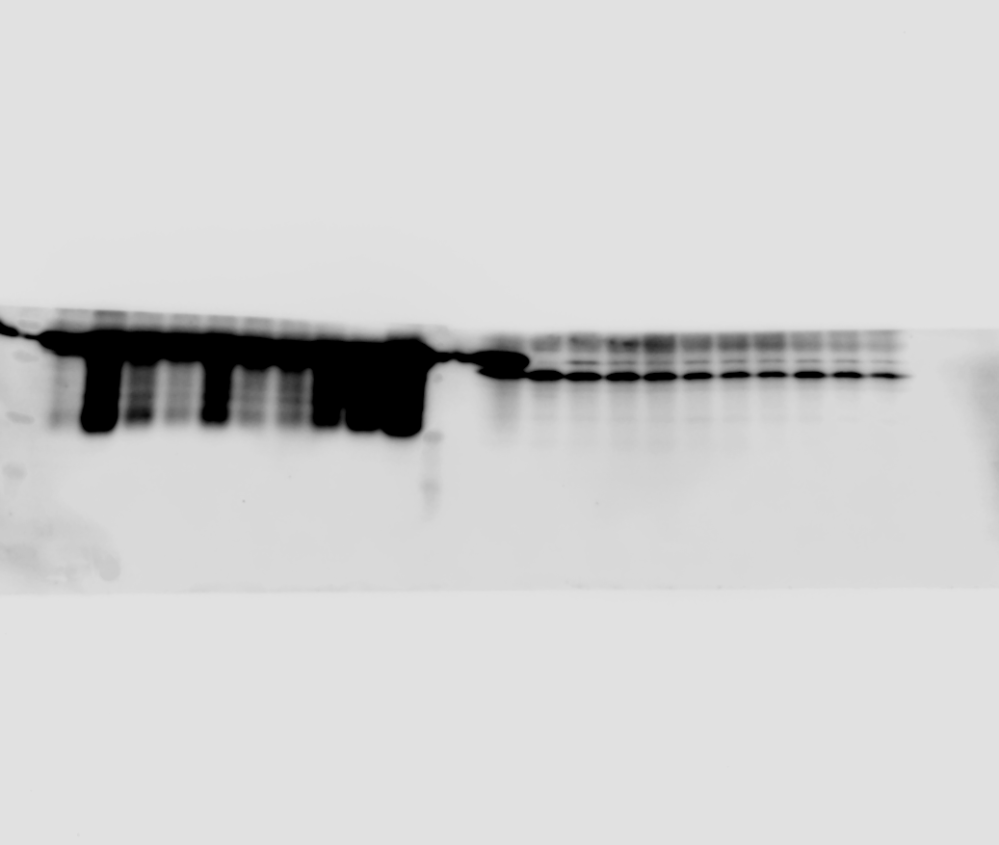

Supplement: Figure 3—source data 1. [file elife-84330-fig3-data1.zip › Figure 3-source data 1/Fig3C_Aac2.tiff]

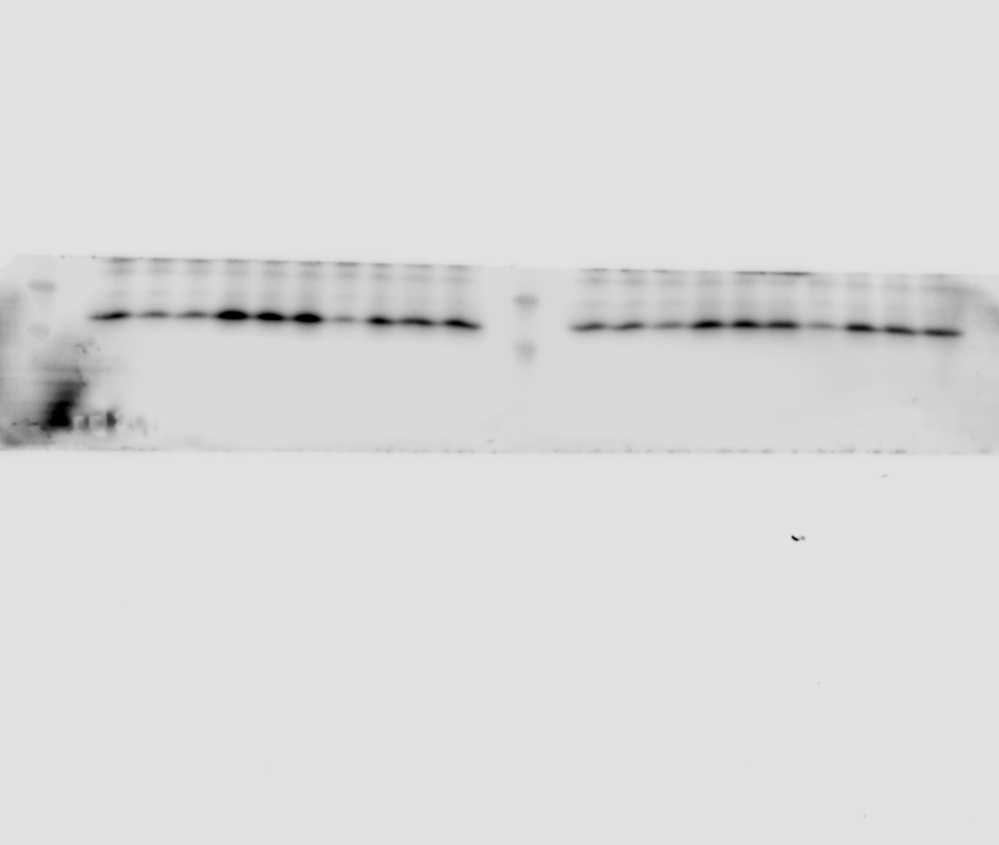

Supplement: Figure 3—source data 1. [file elife-84330-fig3-data1.zip › Figure 3-source data 1/Fig3A_Sml1.tiff]

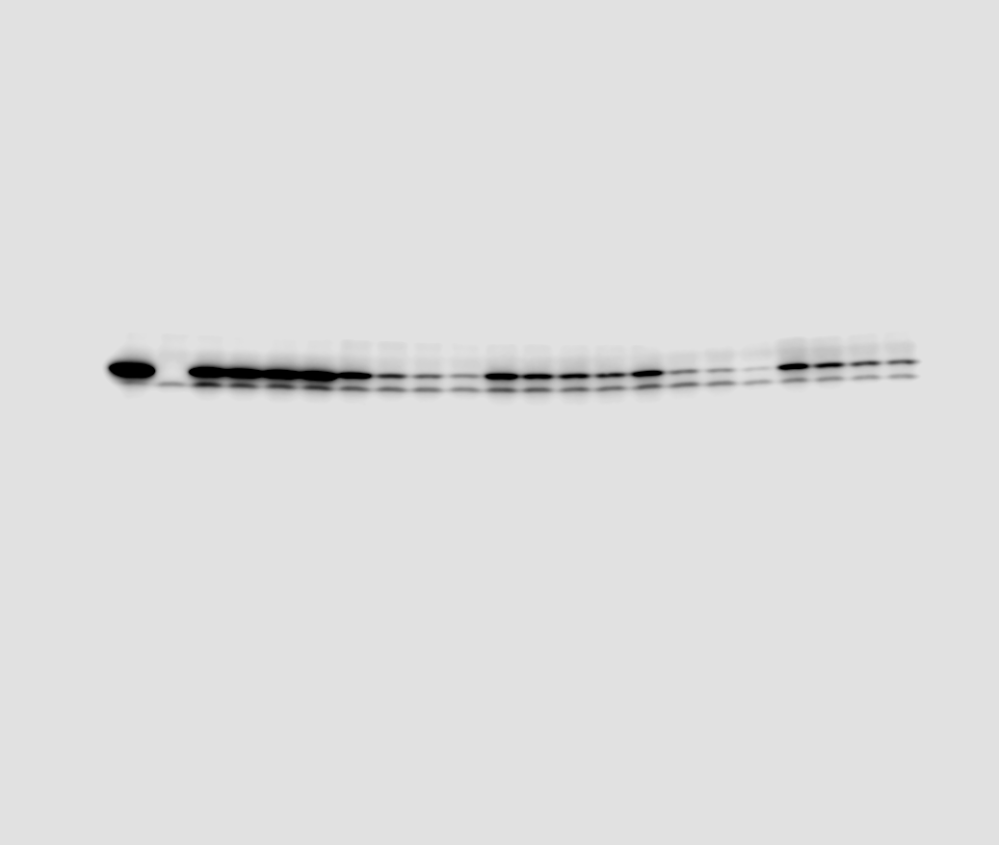

Supplement: Figure 3—source data 1. [file elife-84330-fig3-data1.zip › Figure 3-source data 1/Fig3D_Aac2.tiff]

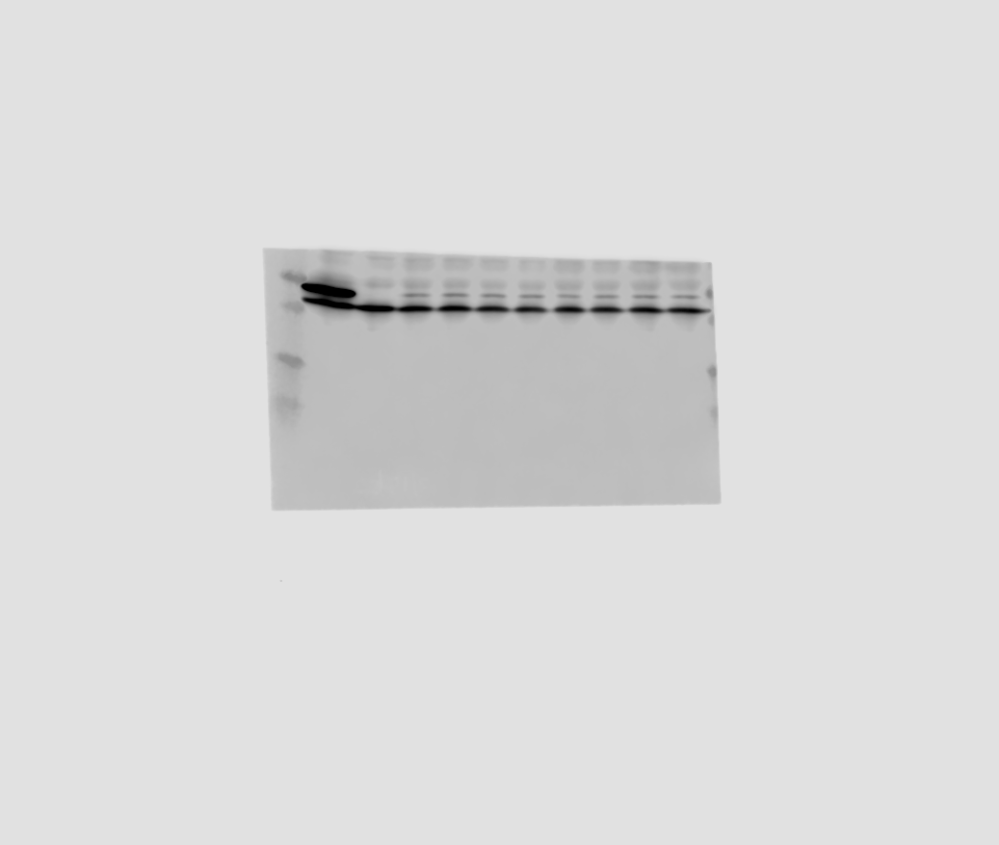

Supplement: Figure 3—source data 1. [file elife-84330-fig3-data1.zip › Figure 3-source data 1/Fig3A_Aac2.tiff]

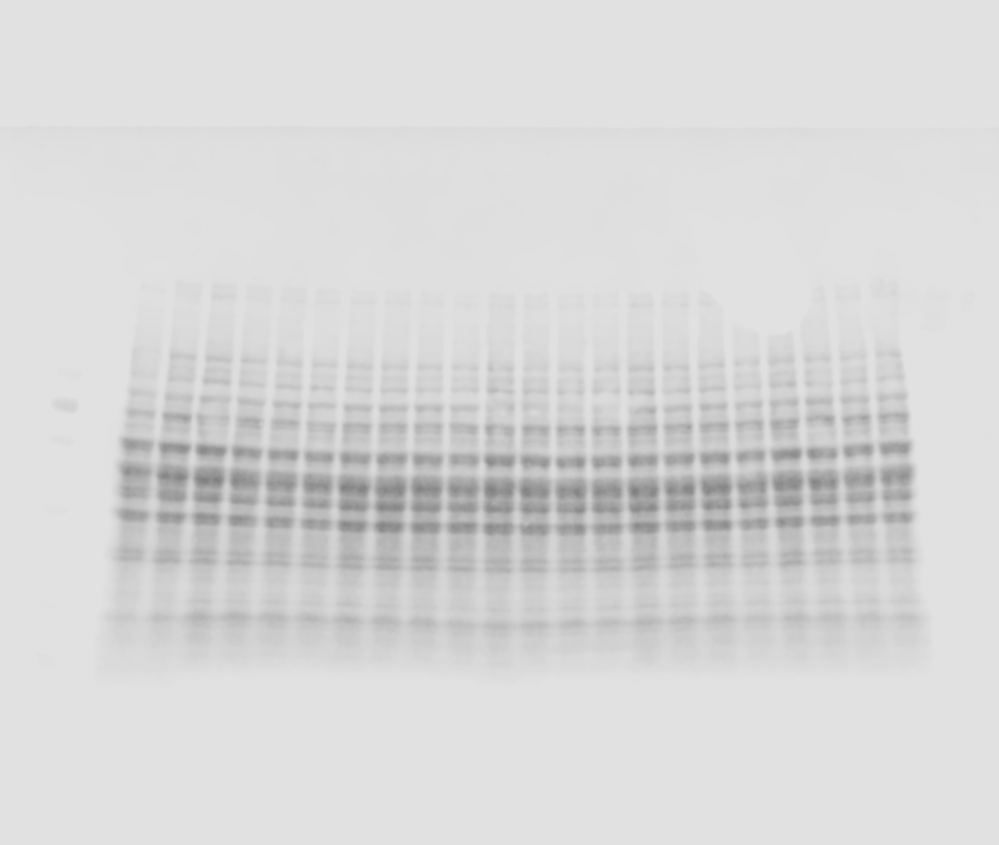

Supplement: Figure 3—source data 1. [file elife-84330-fig3-data1.zip › Figure 3-source data 1/Fig3D_TPS.tiff]

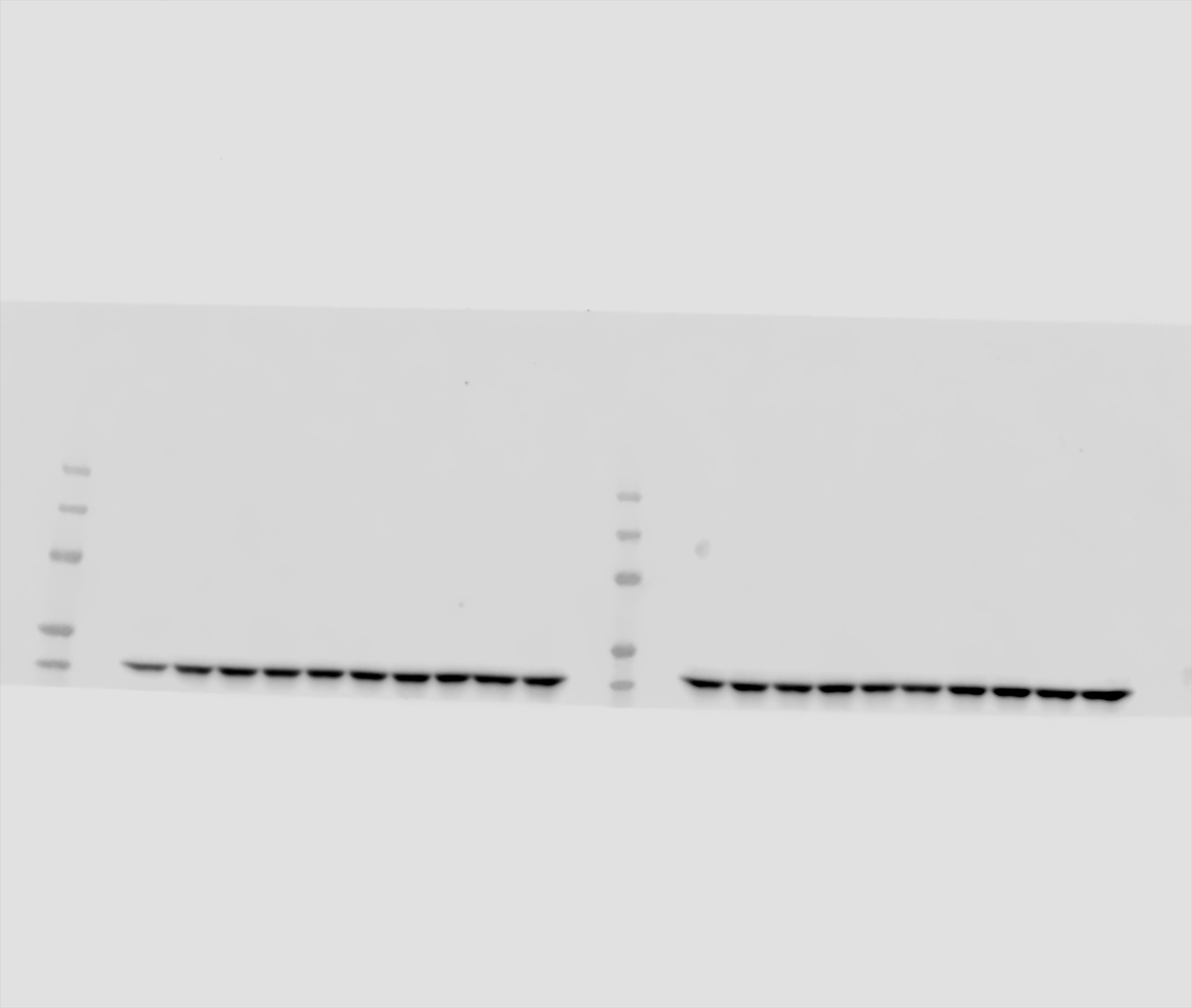

Supplement: Figure 3—source data 1. [file elife-84330-fig3-data1.zip › Figure 3-source data 1/Fig3A_Pgk1.tif]

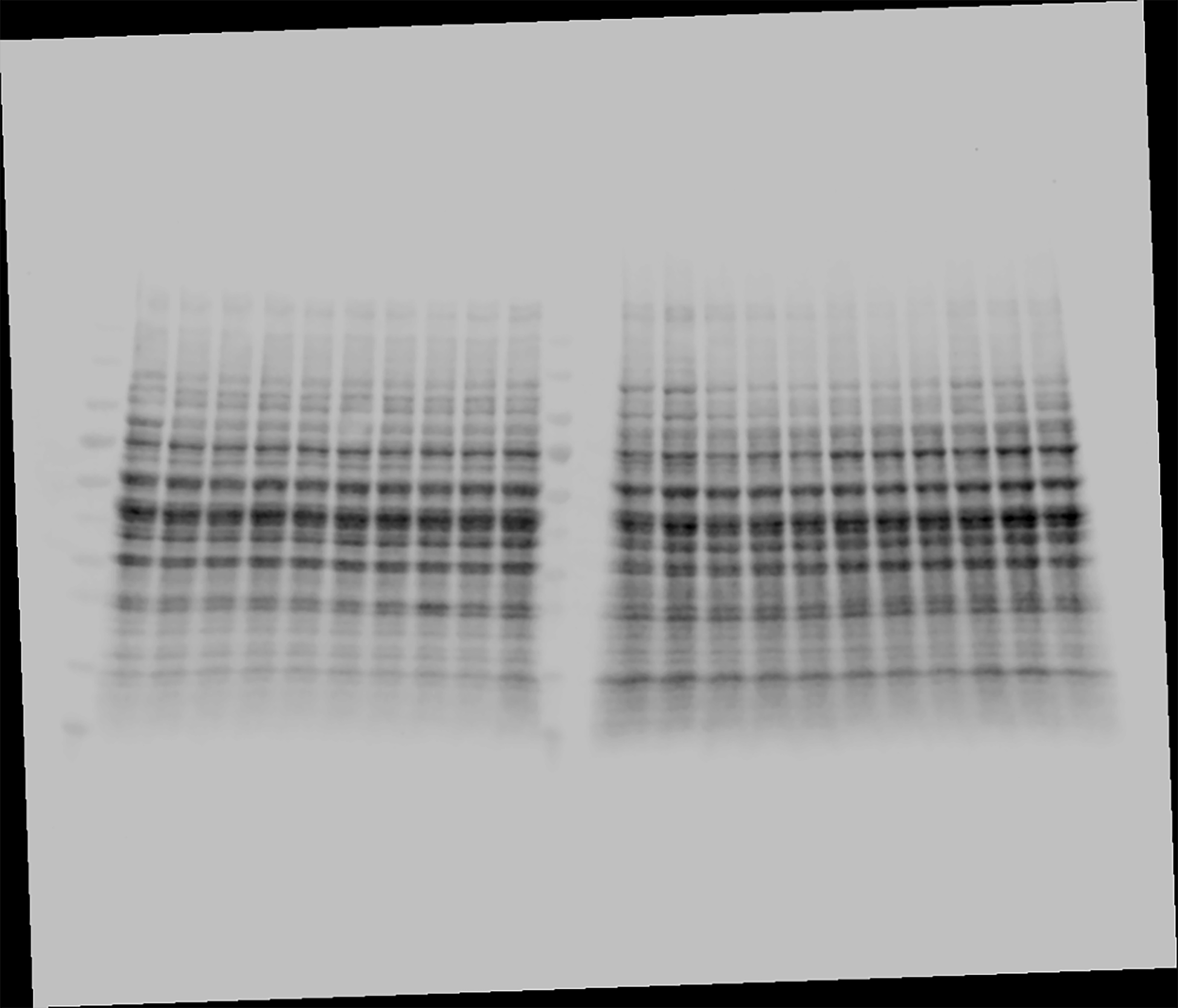

Supplement: Figure 3—source data 1. [file elife-84330-fig3-data1.zip › Figure 3-source data 1/Fig3C_TPS.tiff]

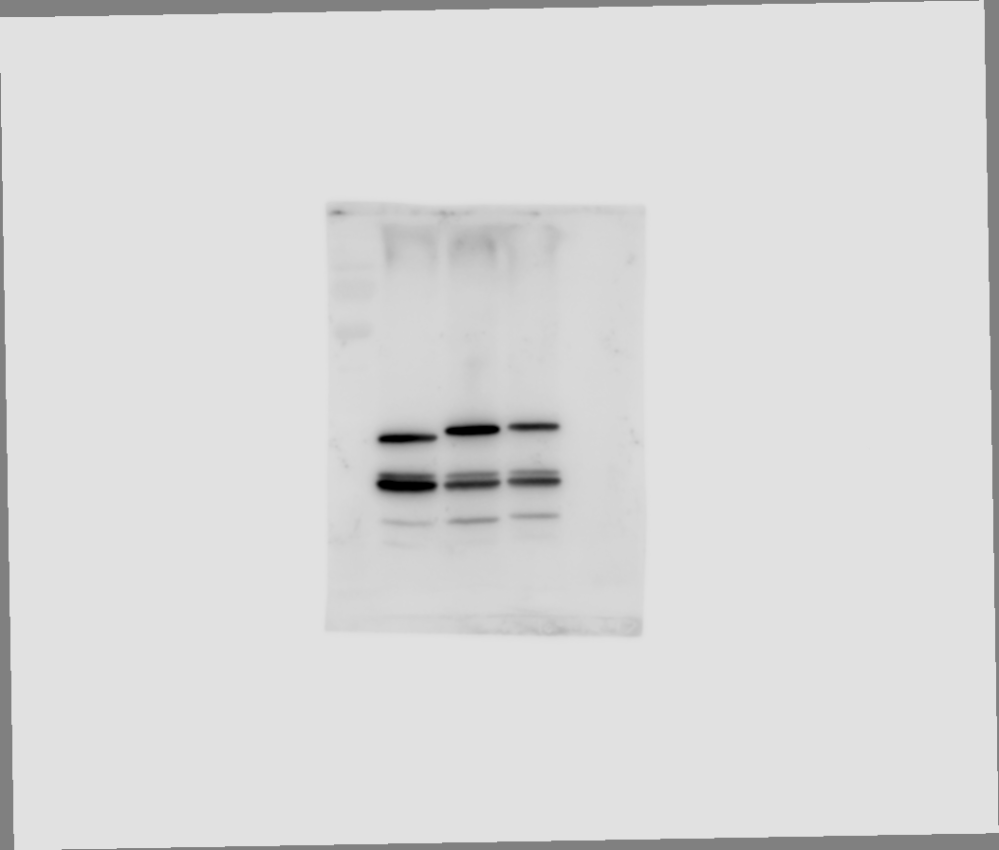

Supplement: Figure 4—figure supplement 1—source data 1. [file elife-84330-fig4-figsupp1-data1.zip › Figure 4-figure supplement 1-source data 1/Fig 4-fs1A Aac2.tif]

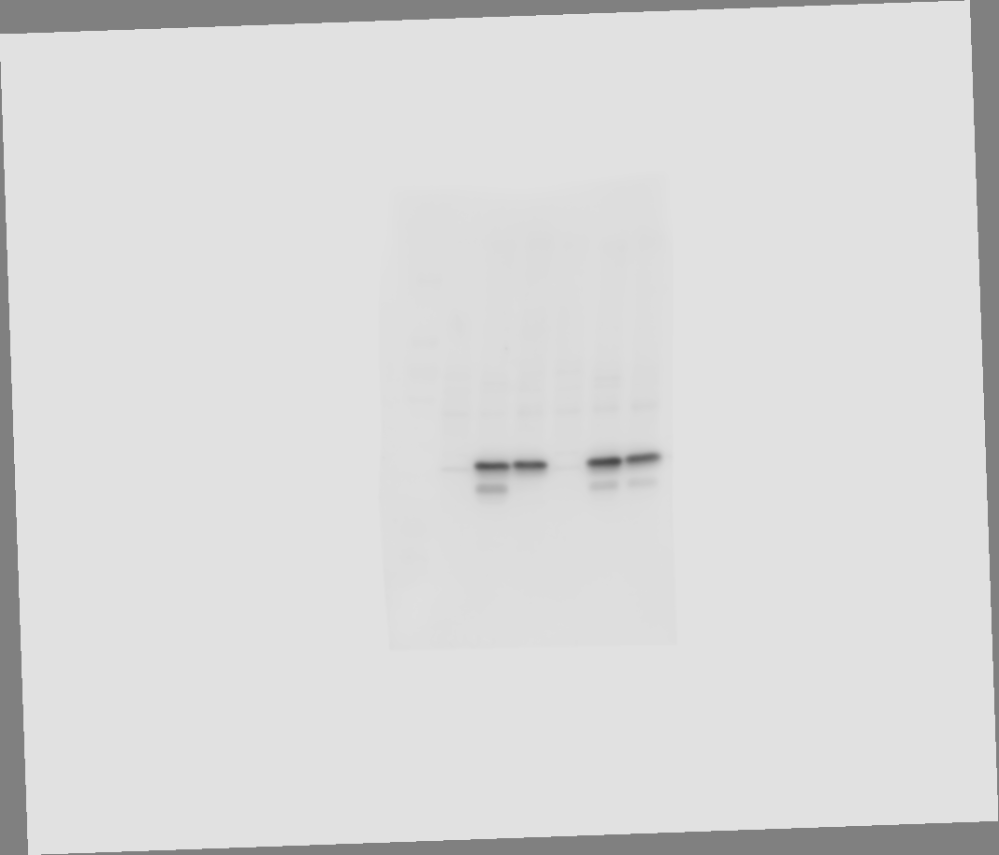

Supplement: Figure 4—figure supplement 1—source data 1. [file elife-84330-fig4-figsupp1-data1.zip › Figure 4-figure supplement 1-source data 1/Fig 4-fs1G Aac2 Eluate.tif]

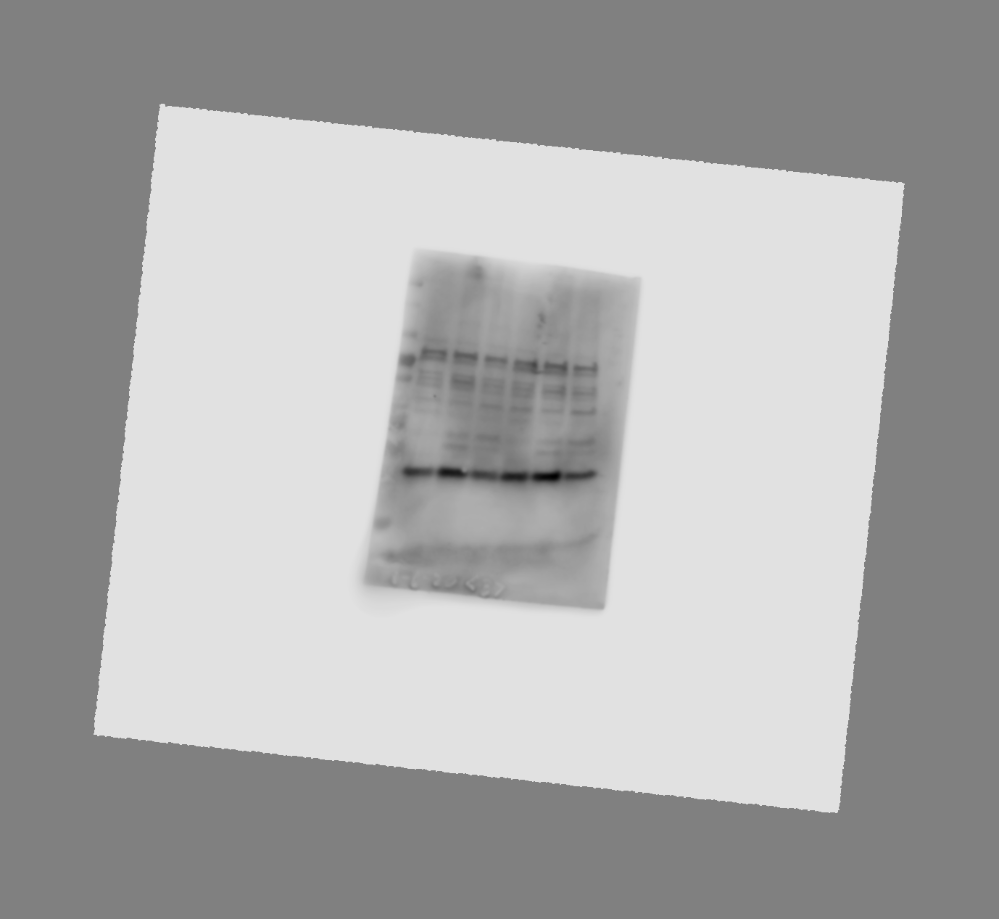

Supplement: Figure 4—figure supplement 1—source data 1. [file elife-84330-fig4-figsupp1-data1.zip › Figure 4-figure supplement 1-source data 1/Fig 4-fs1G Tim22 Input.tif]

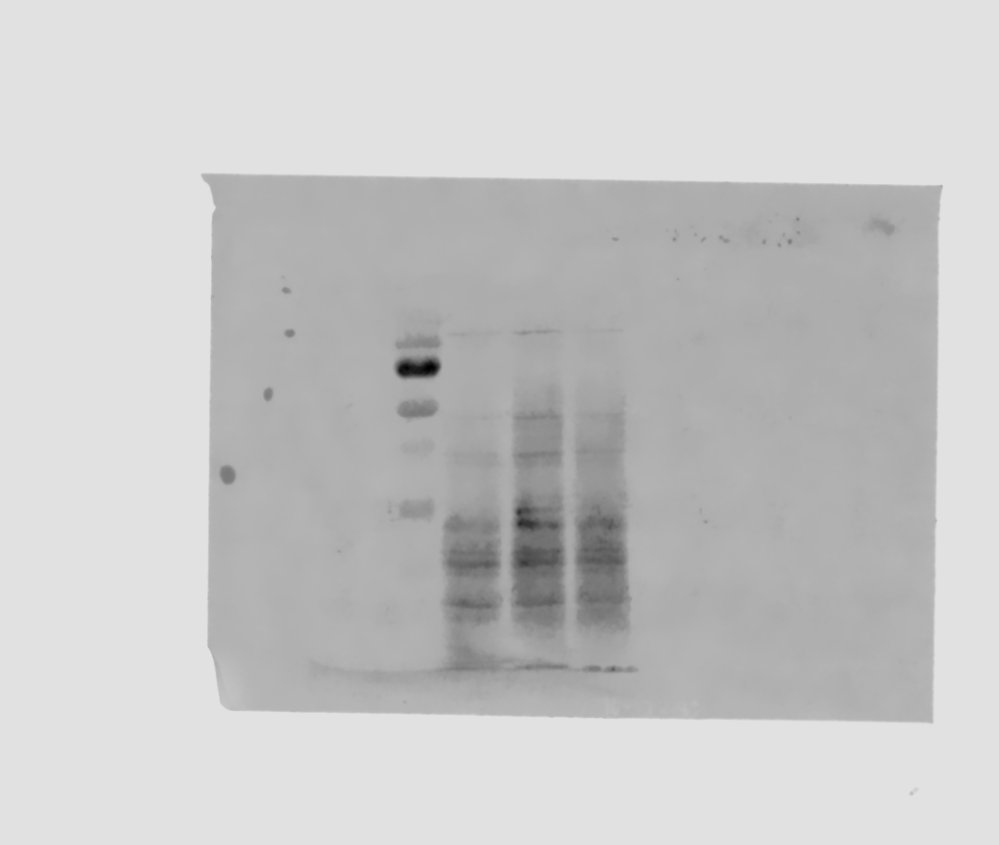

Supplement: Figure 4—figure supplement 1—source data 1. [file elife-84330-fig4-figsupp1-data1.zip › Figure 4-figure supplement 1-source data 1/Fig 4-fs1A TPS.tif]

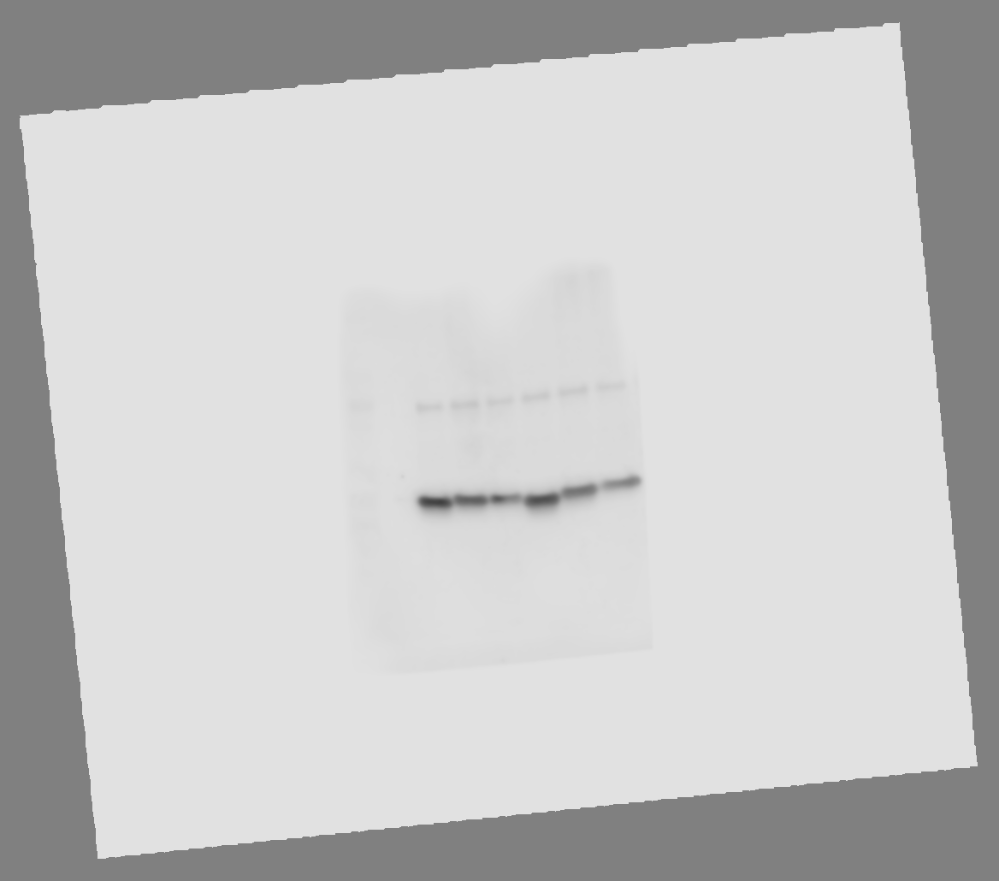

Supplement: Figure 4—figure supplement 1—source data 1. [file elife-84330-fig4-figsupp1-data1.zip › Figure 4-figure supplement 1-source data 1/Fig 4-fs1G Aac2 Input.tif]

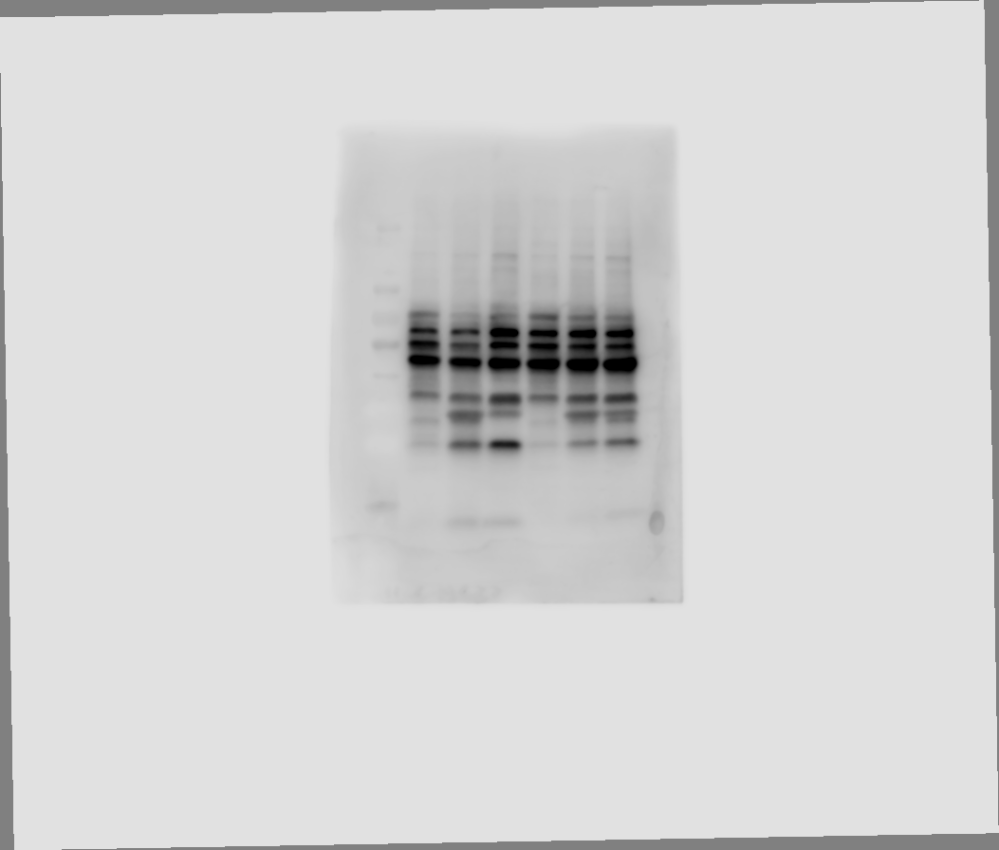

Supplement: Figure 4—figure supplement 1—source data 1. [file elife-84330-fig4-figsupp1-data1.zip › Figure 4-figure supplement 1-source data 1/Fig 4-fs1G Tim22 Eluate.tif]

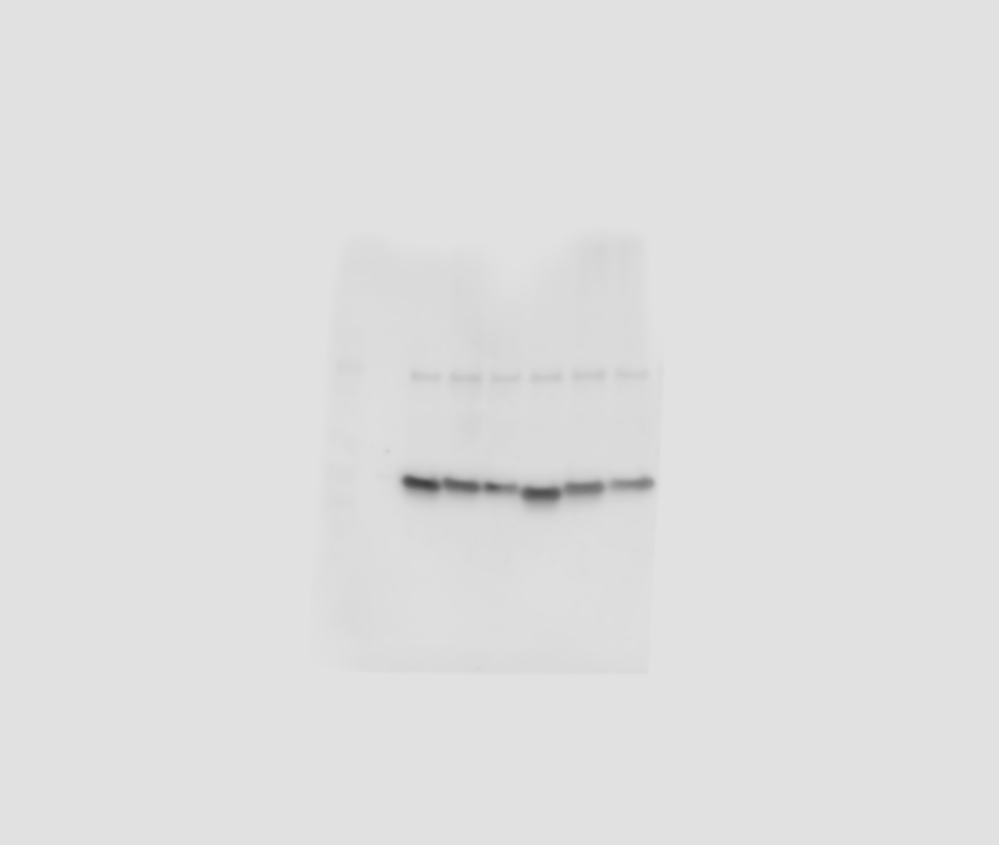

Supplement: Figure 4—figure supplement 2—source data 2. [file elife-84330-fig4-figsupp2-data2.zip › Figure 4-figure supplement 2-source datat 1/Fig 4-fs2C Aac2 Input.tif]

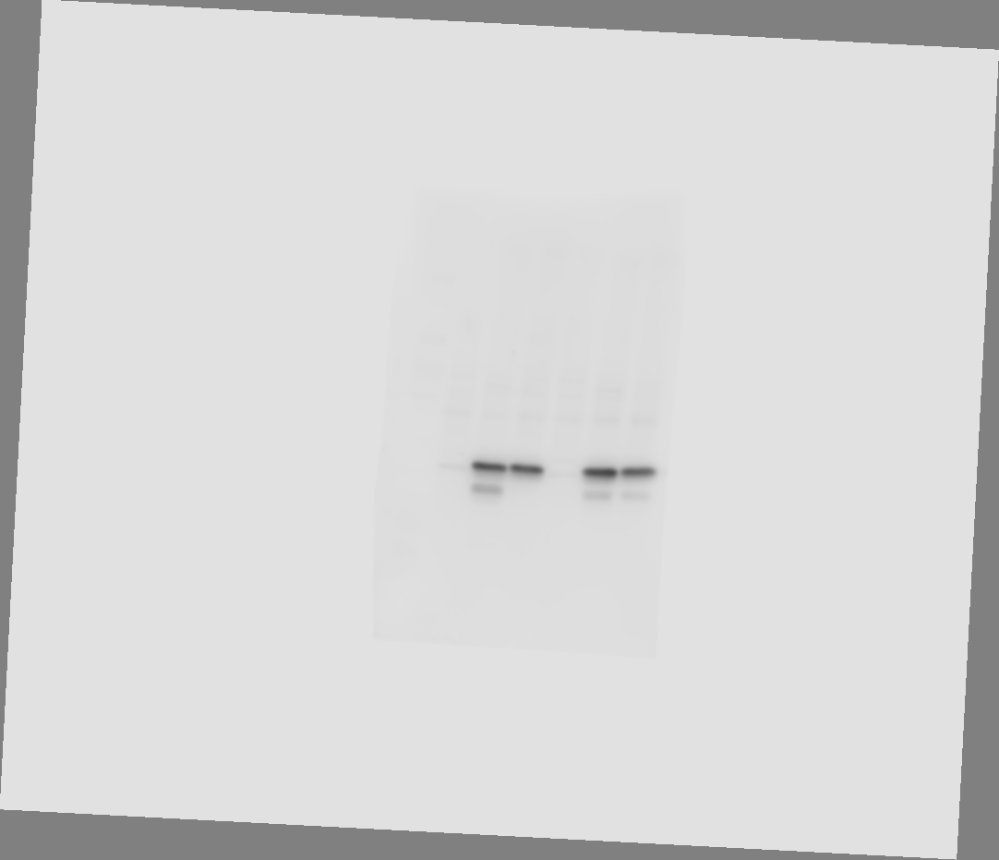

Supplement: Figure 4—figure supplement 2—source data 2. [file elife-84330-fig4-figsupp2-data2.zip › Figure 4-figure supplement 2-source datat 1/Fig 4-fs2C Aac2 Eluate.tif]

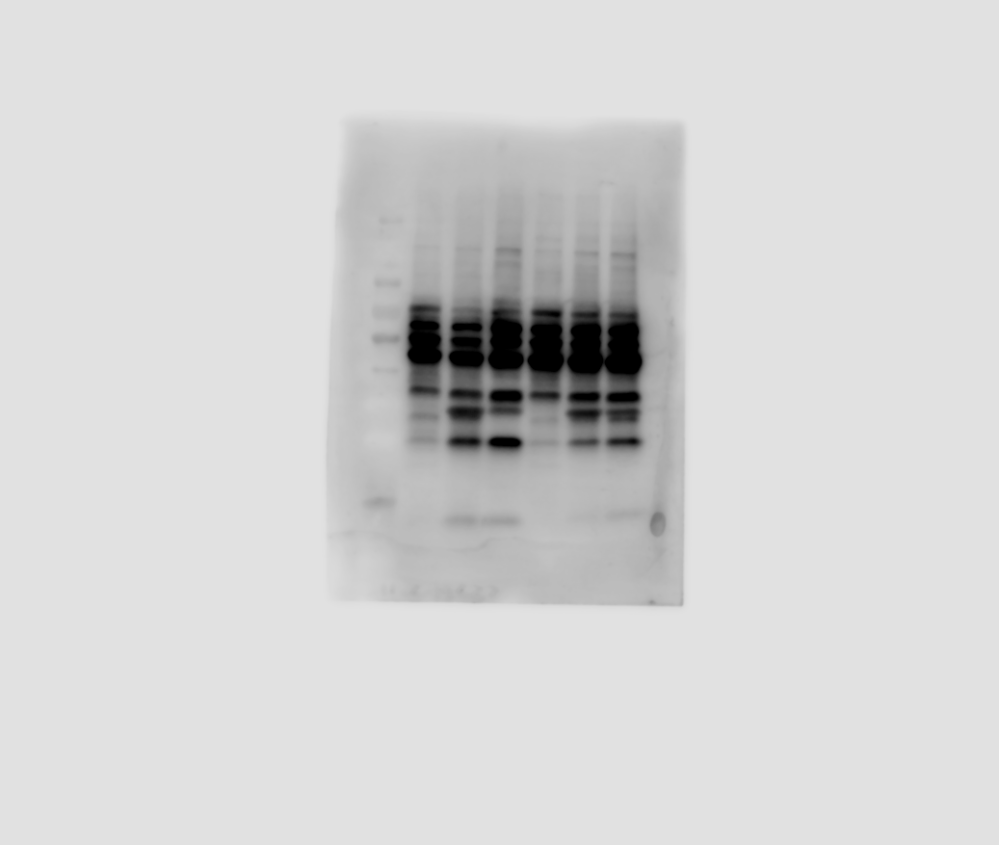

Supplement: Figure 4—figure supplement 2—source data 2. [file elife-84330-fig4-figsupp2-data2.zip › Figure 4-figure supplement 2-source datat 1/Fig 4-fs2C Tim22 Eluate.tif]

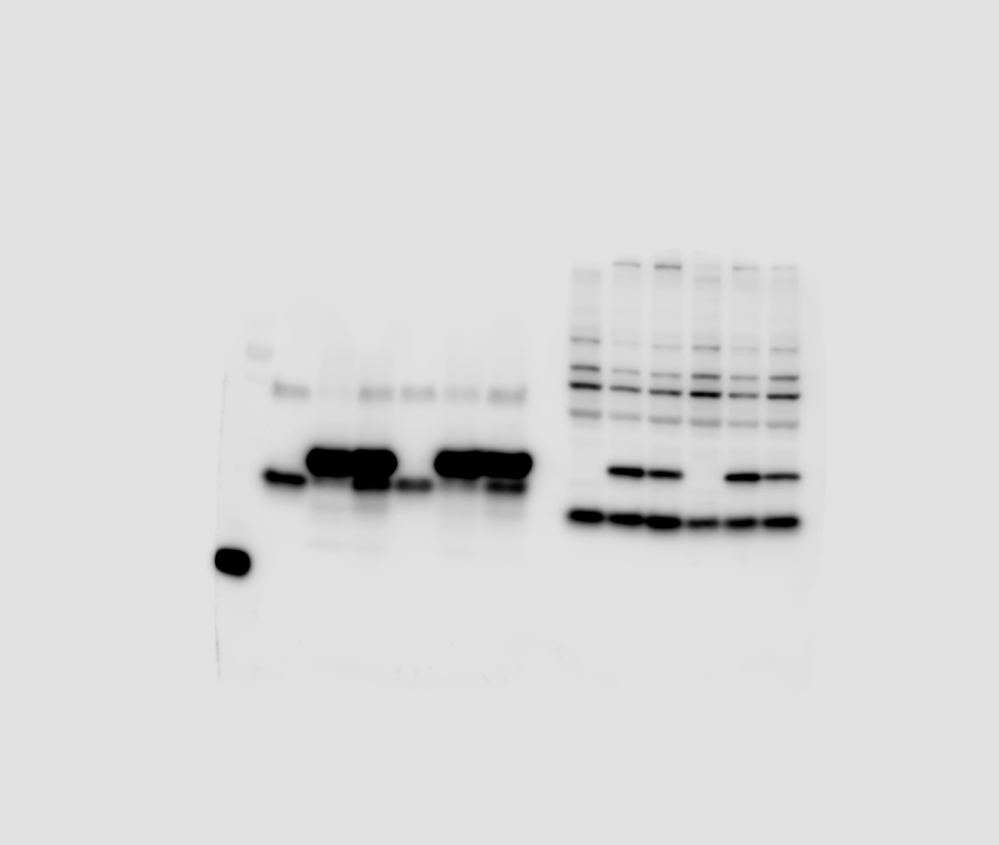

Supplement: Figure 5—source data 2. [file elife-84330-fig5-data2.zip › Figure 5-source data 1/5B Input HA.tif]

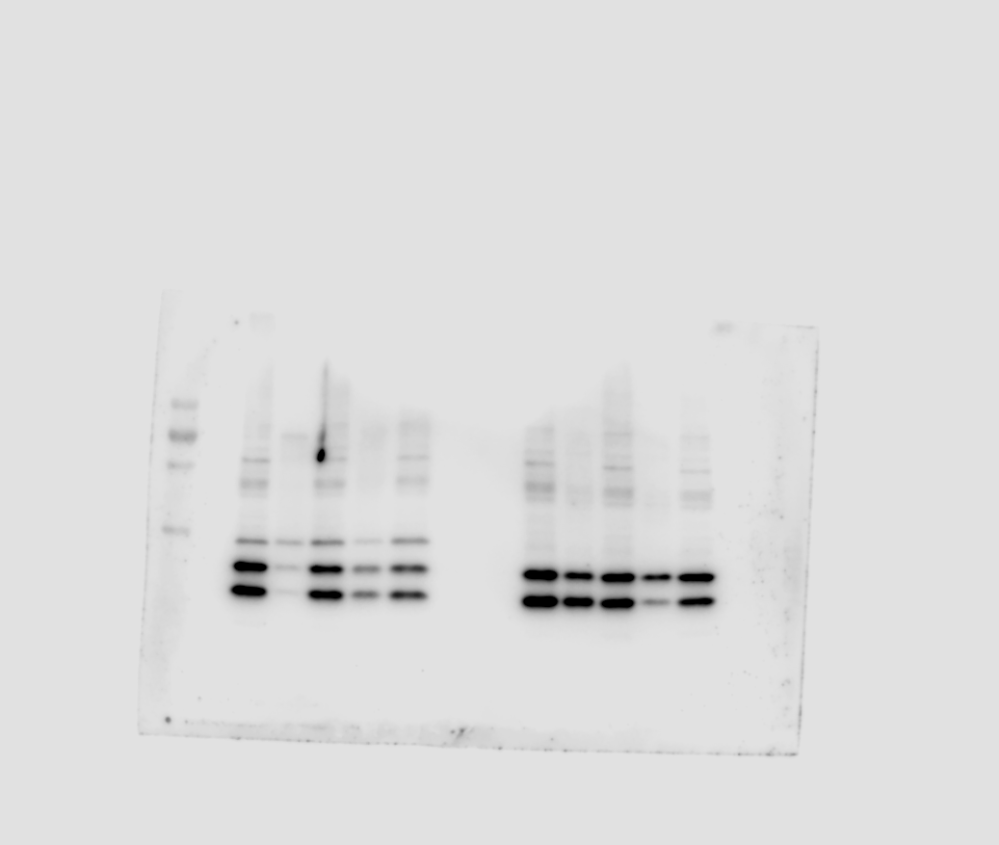

Supplement: Figure 5—source data 2. [file elife-84330-fig5-data2.zip › Figure 5-source data 1/5D Ant1_A114P_A123D anti-TFAM.tif]

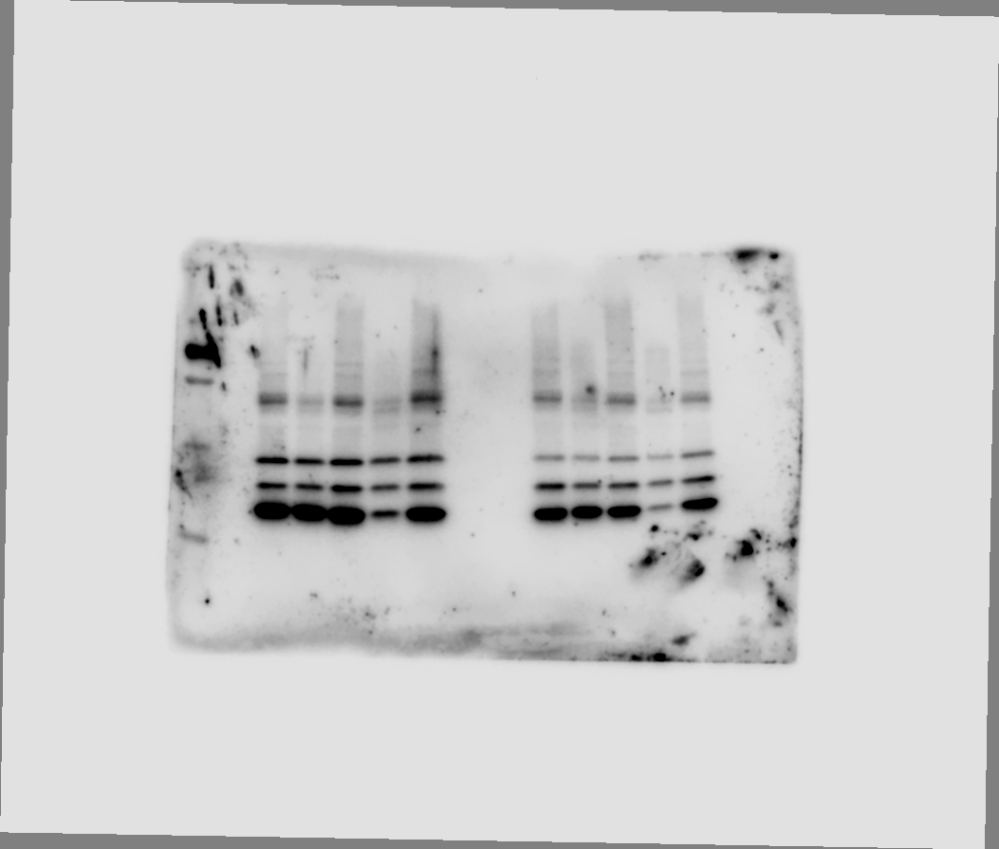

Supplement: Figure 5—source data 2. [file elife-84330-fig5-data2.zip › Figure 5-source data 1/5D Ant1 anti-TFAM.tif]

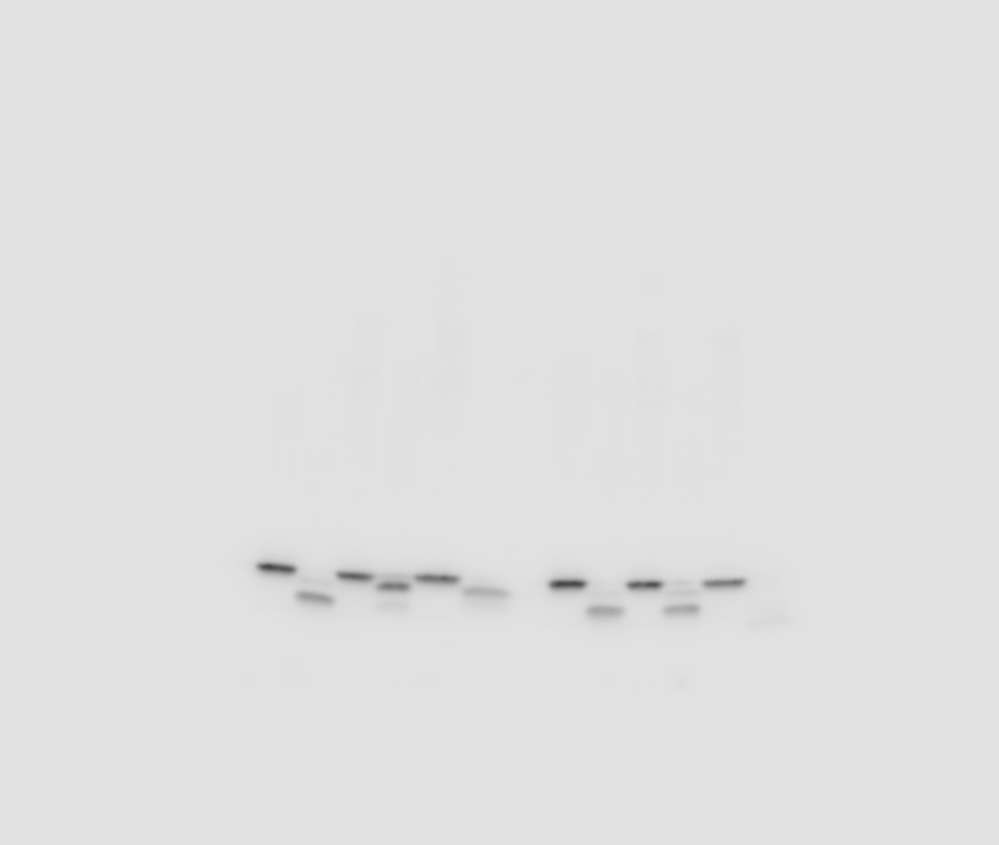

Supplement: Figure 5—source data 2. [file elife-84330-fig5-data2.zip › Figure 5-source data 1/5D Ant1_A114P_A123D anti-Tom20.tif]

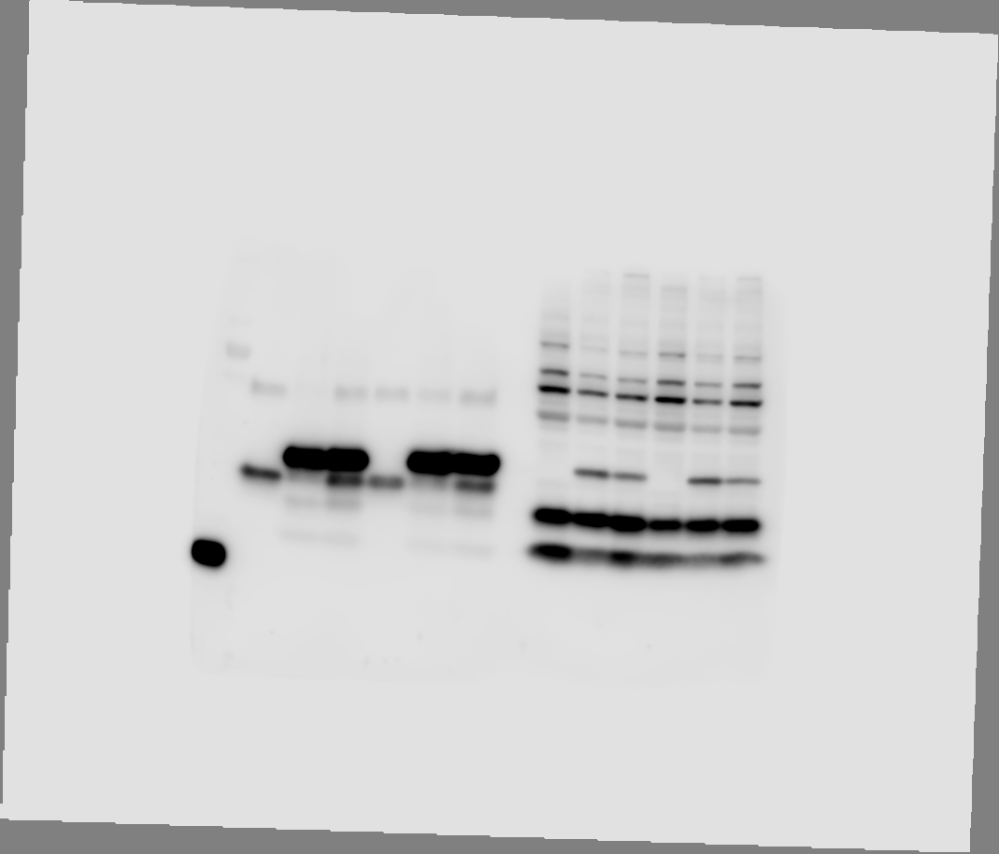

Supplement: Figure 5—source data 2. [file elife-84330-fig5-data2.zip › Figure 5-source data 1/5B Input Tom20.tif]

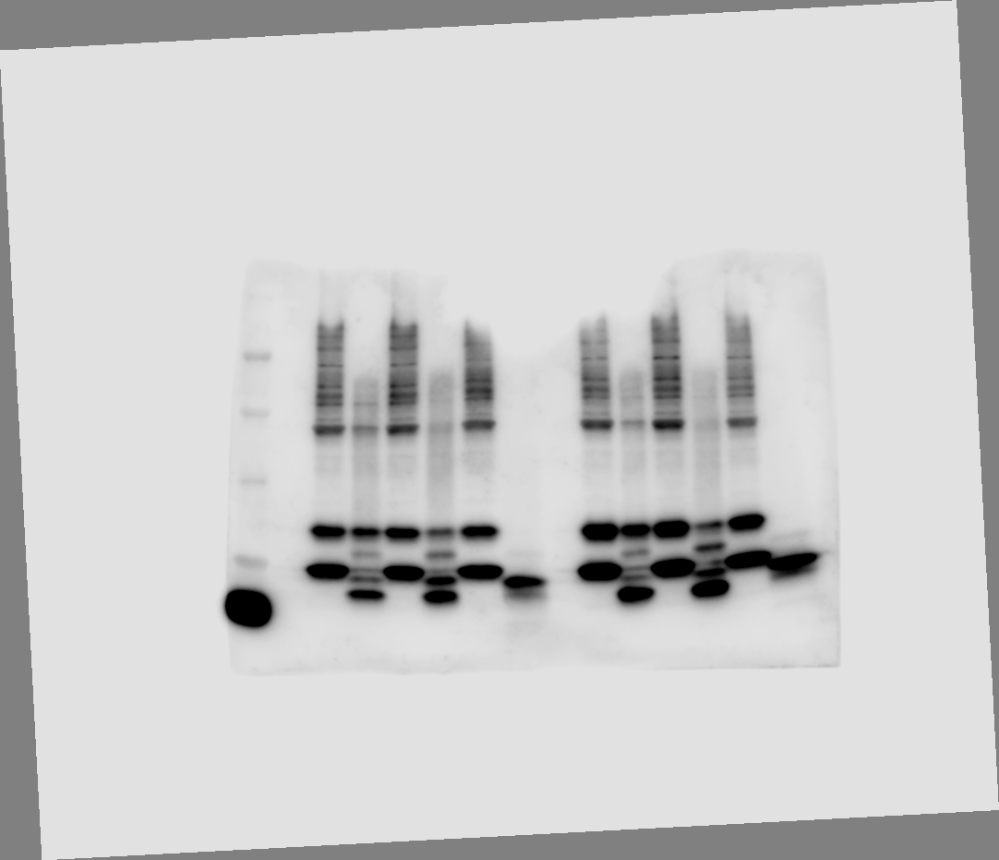

Supplement: Figure 5—source data 2. [file elife-84330-fig5-data2.zip › Figure 5-source data 1/5D Ant1_A114P anti-Tim22.tif]

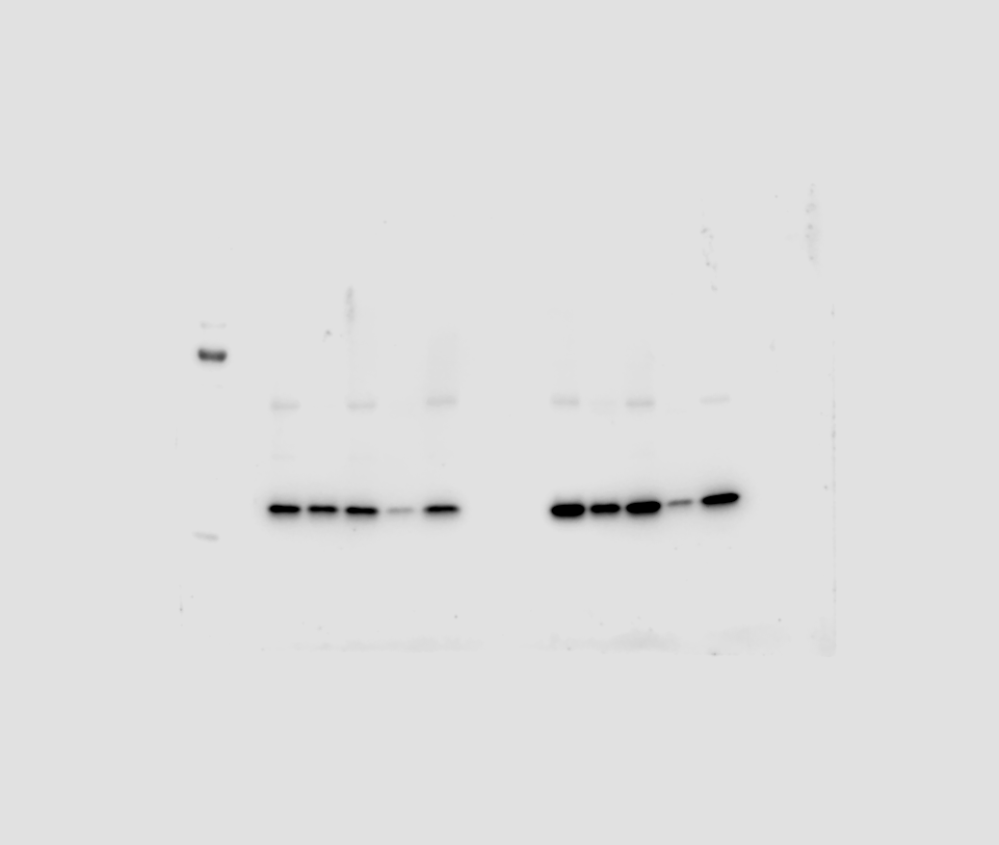

Supplement: Figure 5—source data 2. [file elife-84330-fig5-data2.zip › Figure 5-source data 1/5D Ant1_A114P anti-Smac.tif]

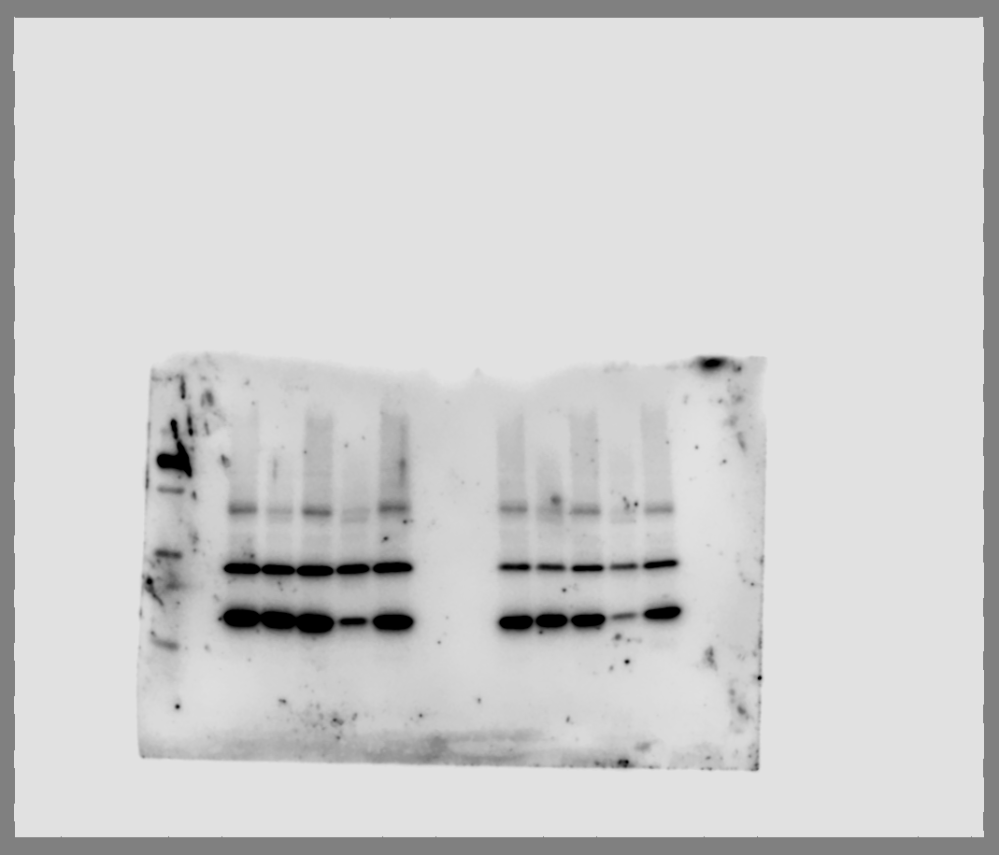

Supplement: Figure 5—source data 2. [file elife-84330-fig5-data2.zip › Figure 5-source data 1/5D Ant1_A123D anti-HA.tif]

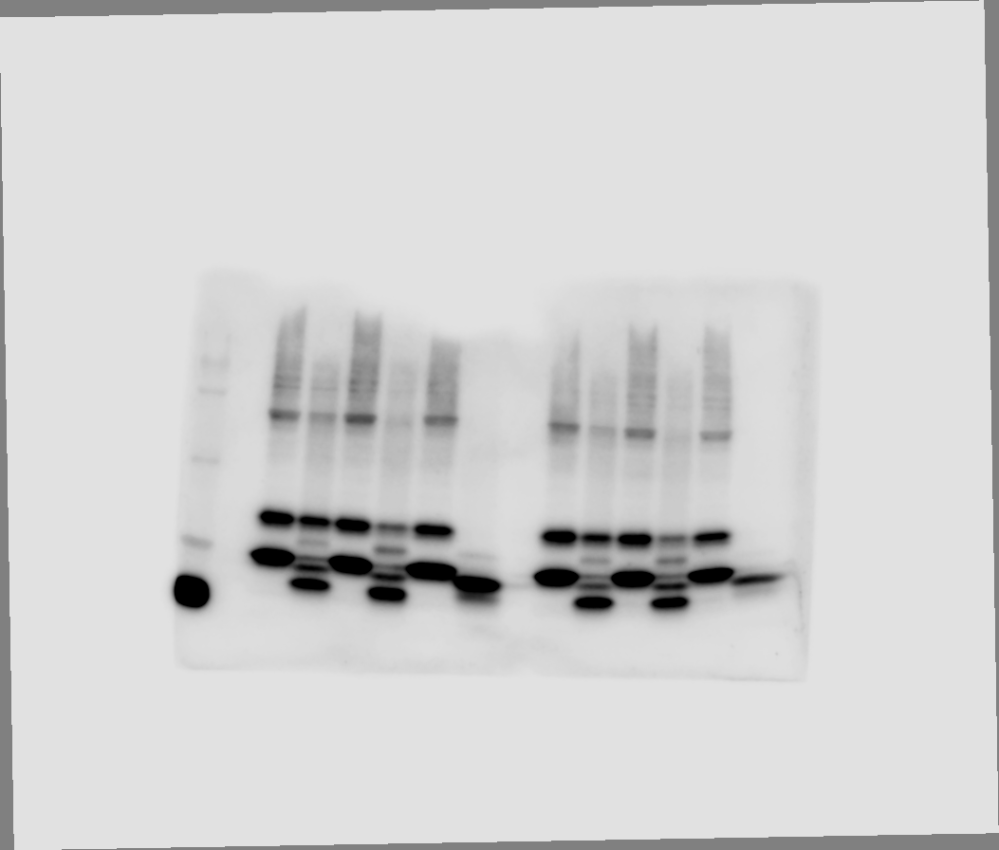

Supplement: Figure 5—source data 2. [file elife-84330-fig5-data2.zip › Figure 5-source data 1/5D Ant1_A123D anti-Tim22.tif]

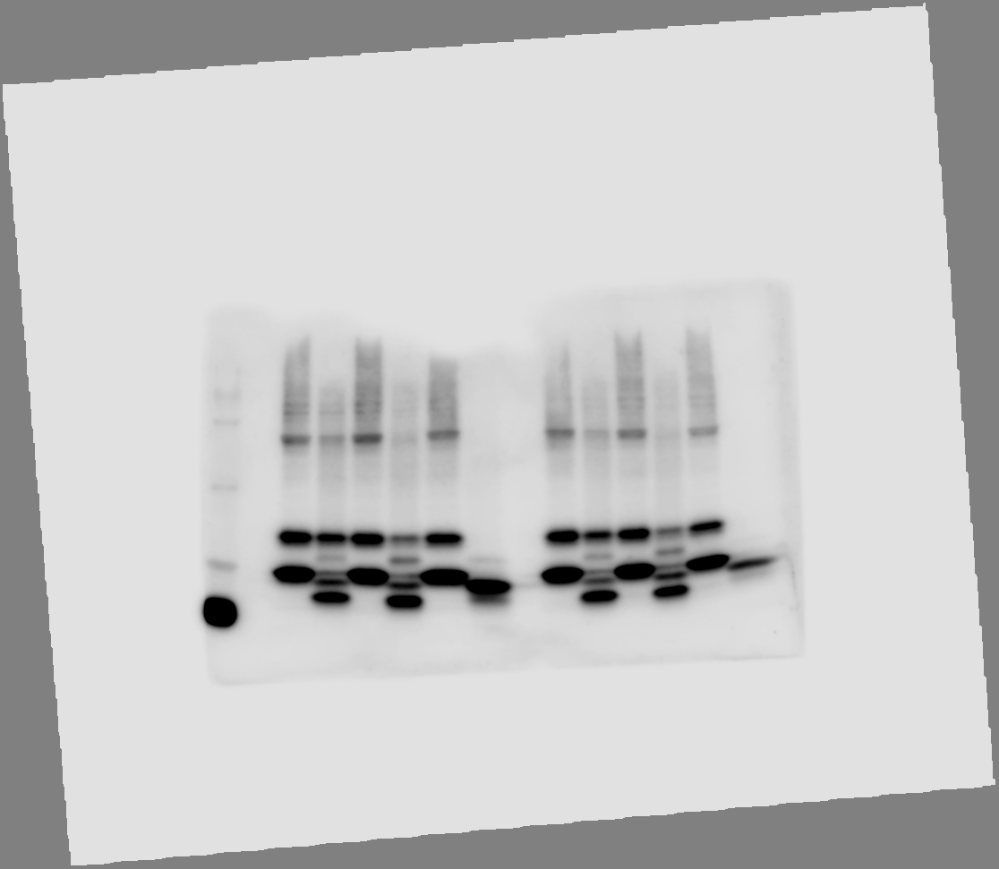

Supplement: Figure 5—source data 2. [file elife-84330-fig5-data2.zip › Figure 5-source data 1/5D Ant1 anti-Tim22.tif]

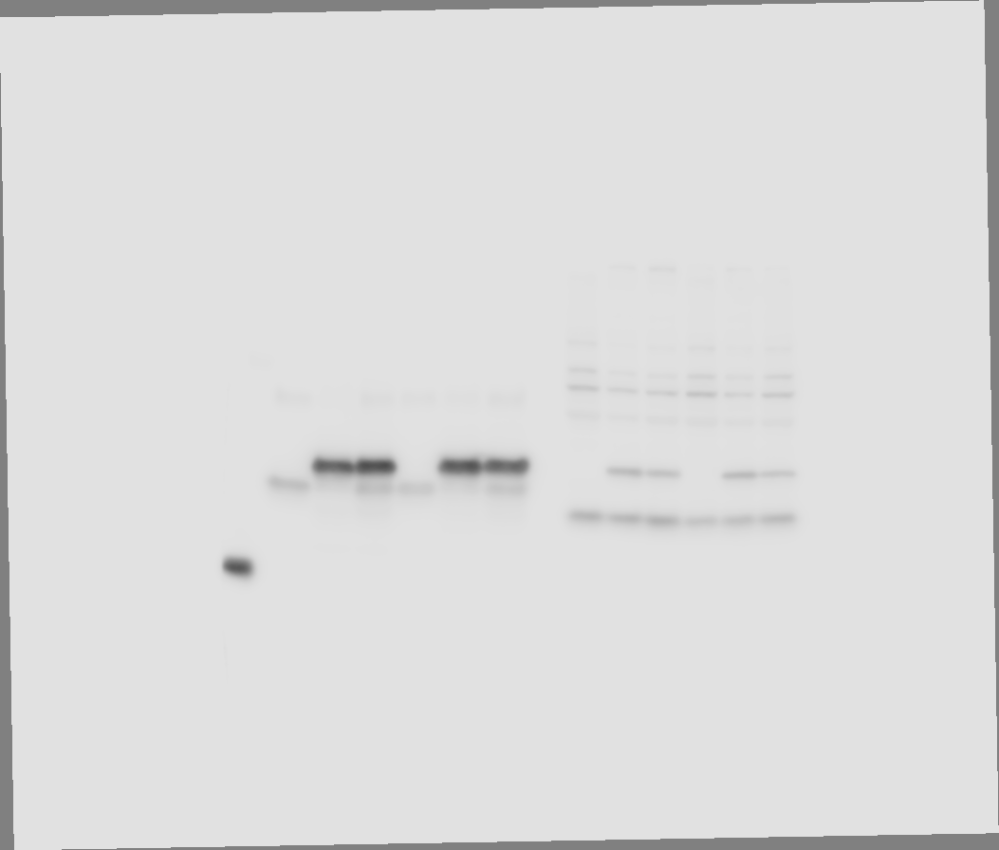

Supplement: Figure 5—source data 2. [file elife-84330-fig5-data2.zip › Figure 5-source data 1/5B IP HA.tif]

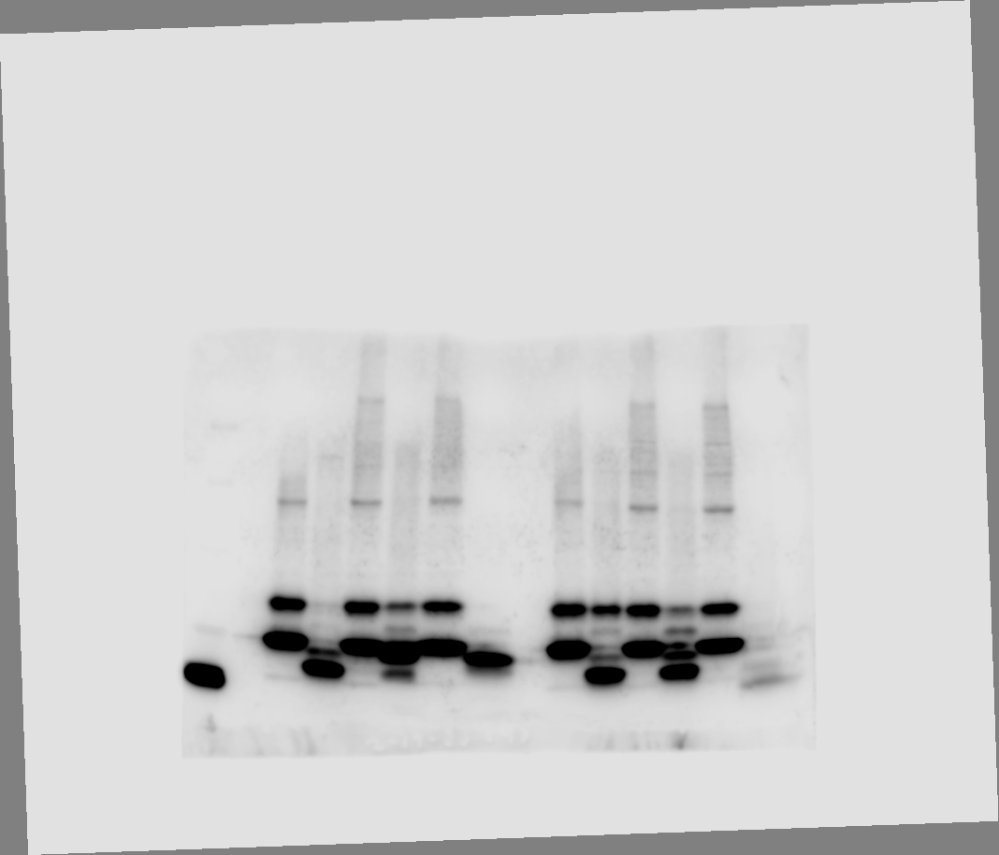

Supplement: Figure 5—source data 2. [file elife-84330-fig5-data2.zip › Figure 5-source data 1/5D Ant1_A114P_A123D anti-Tim22.tif]

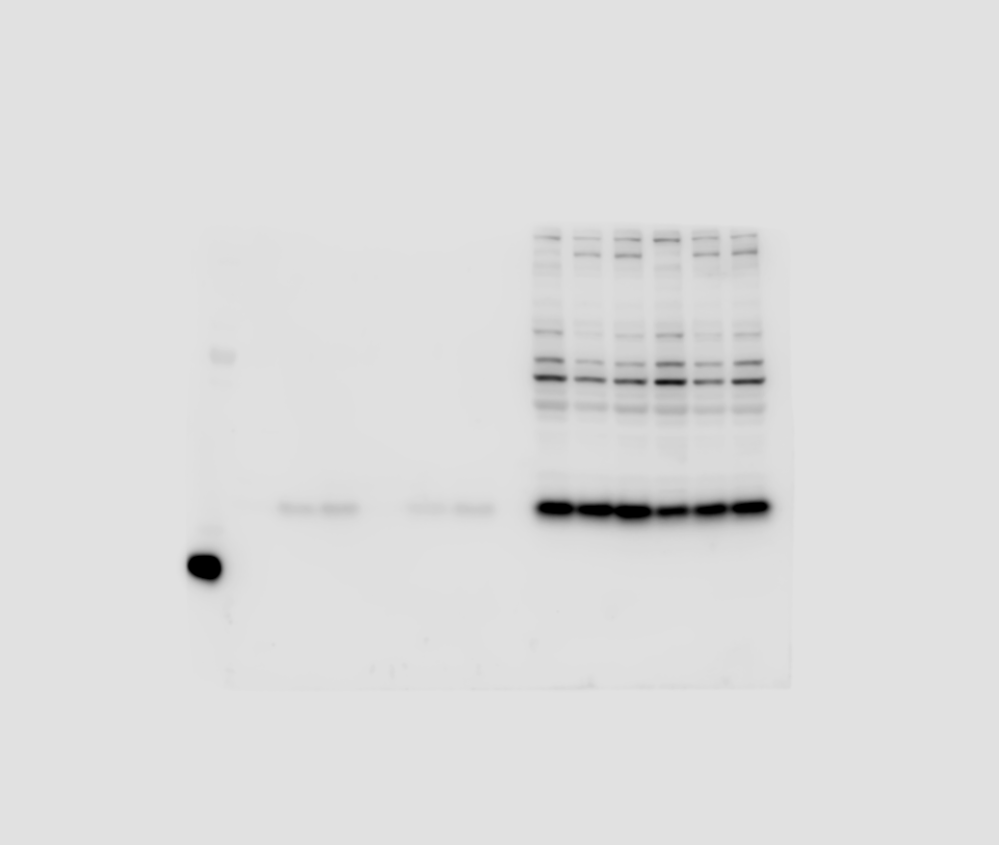

Supplement: Figure 5—source data 2. [file elife-84330-fig5-data2.zip › Figure 5-source data 1/5B Input Tim22.tif]

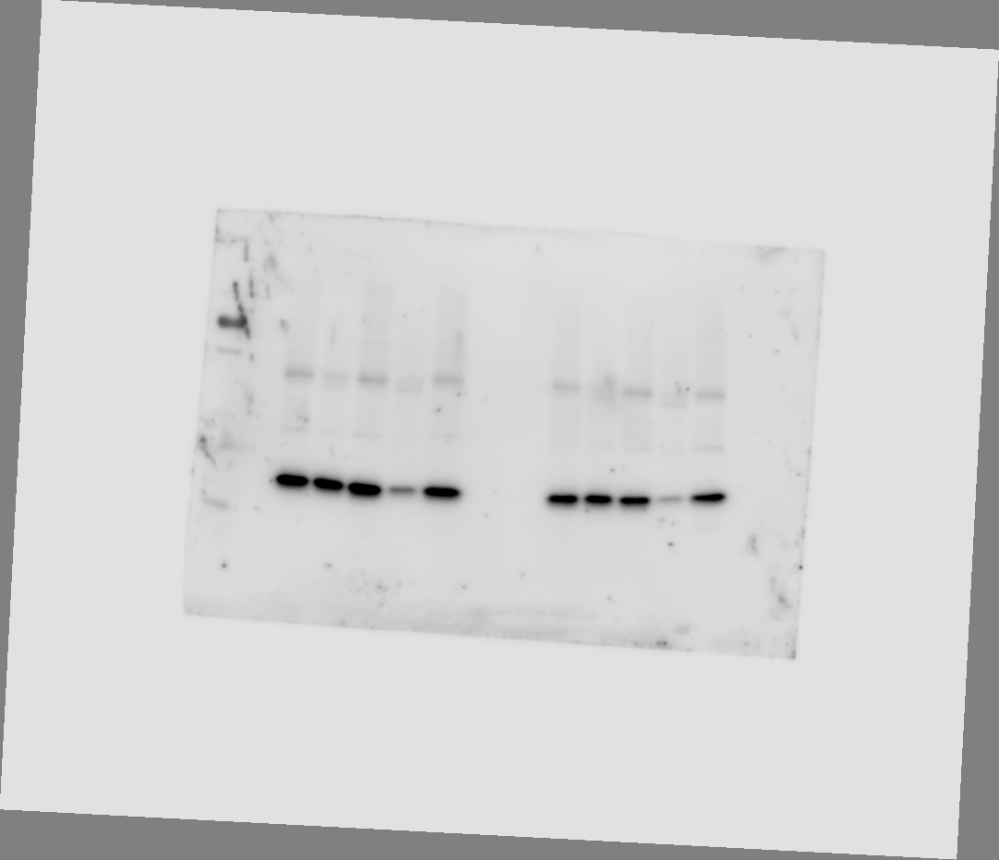

Supplement: Figure 5—source data 2. [file elife-84330-fig5-data2.zip › Figure 5-source data 1/5D Ant1_A123D anti-Smac.tif]

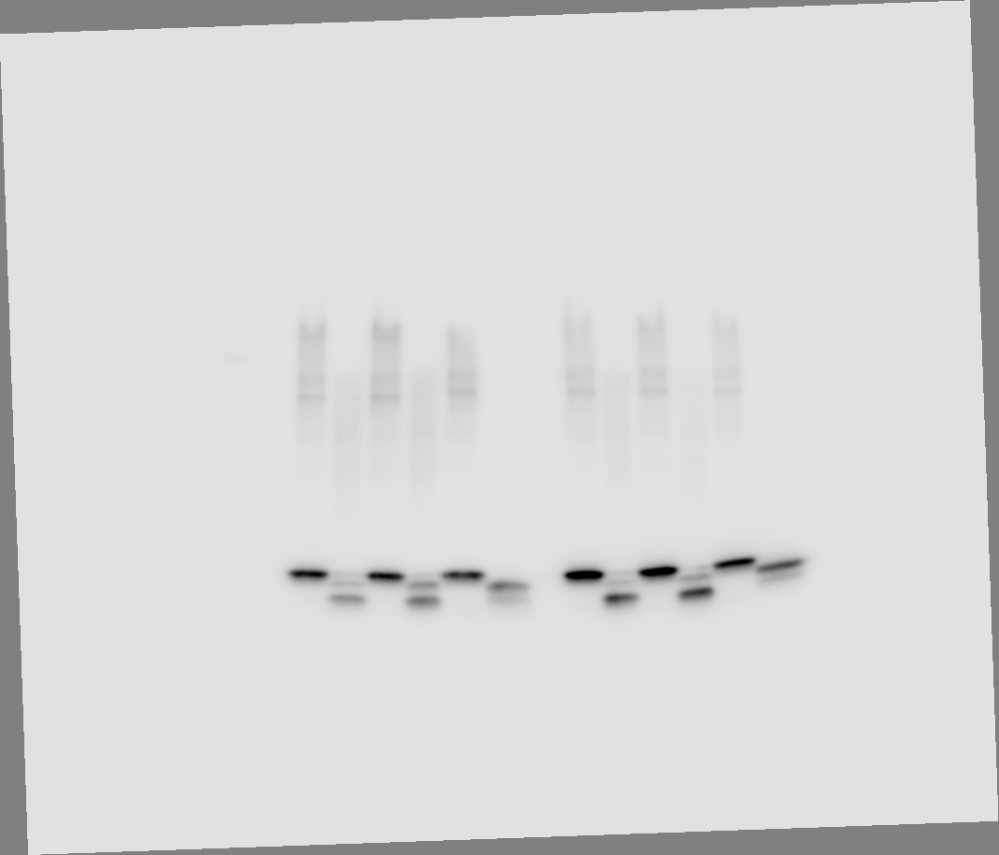

Supplement: Figure 5—source data 2. [file elife-84330-fig5-data2.zip › Figure 5-source data 1/5D Ant1_A114P anti-Tom20.tif]

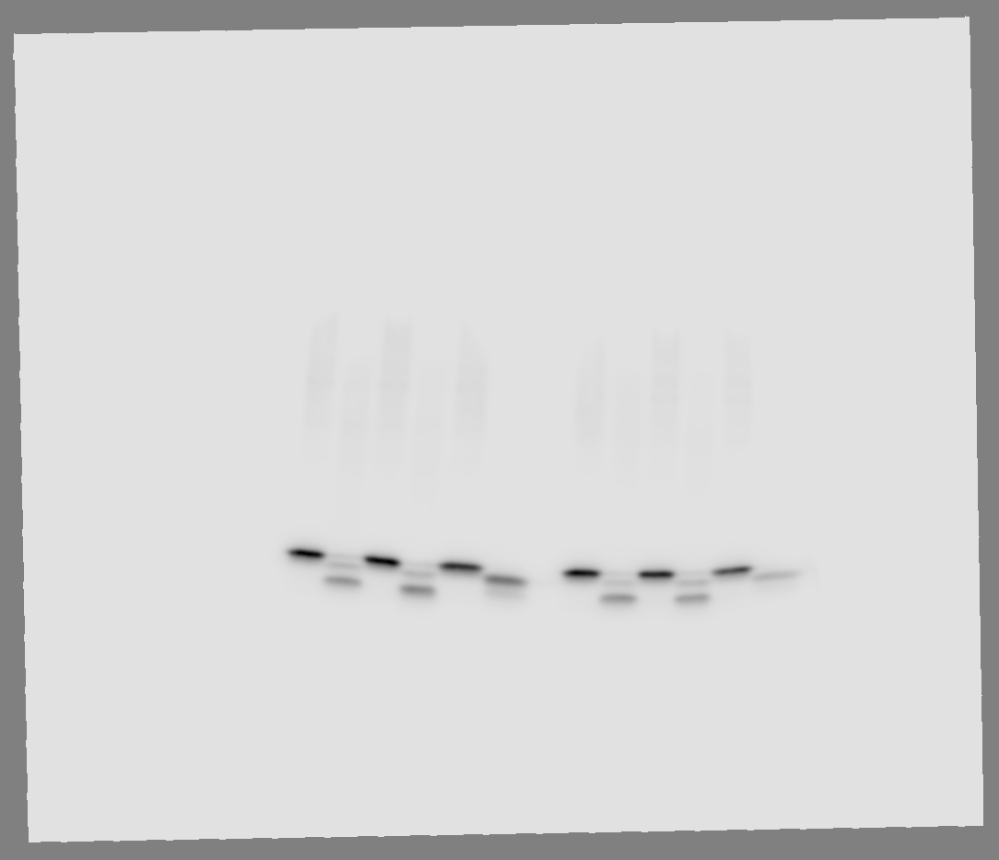

Supplement: Figure 5—source data 2. [file elife-84330-fig5-data2.zip › Figure 5-source data 1/5D Ant1_A123D anti-Tom20.tif]

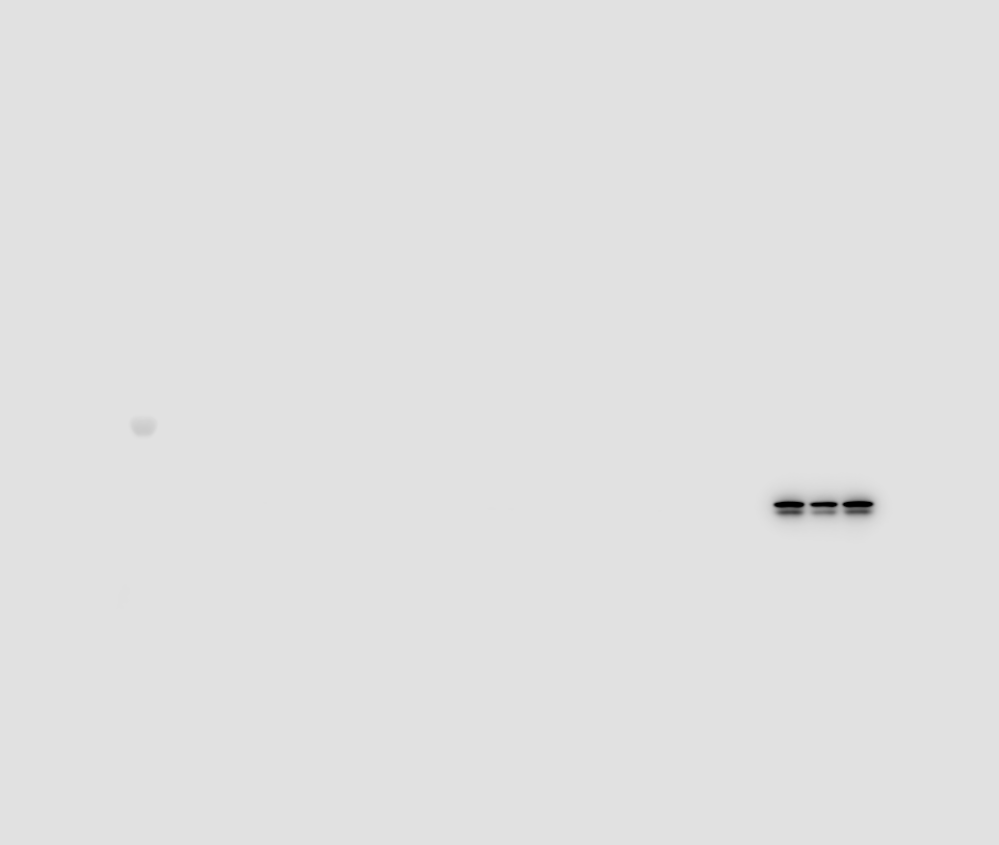

Supplement: Figure 5—source data 2. [file elife-84330-fig5-data2.zip › Figure 5-source data 1/5B Input Tom40.tif]

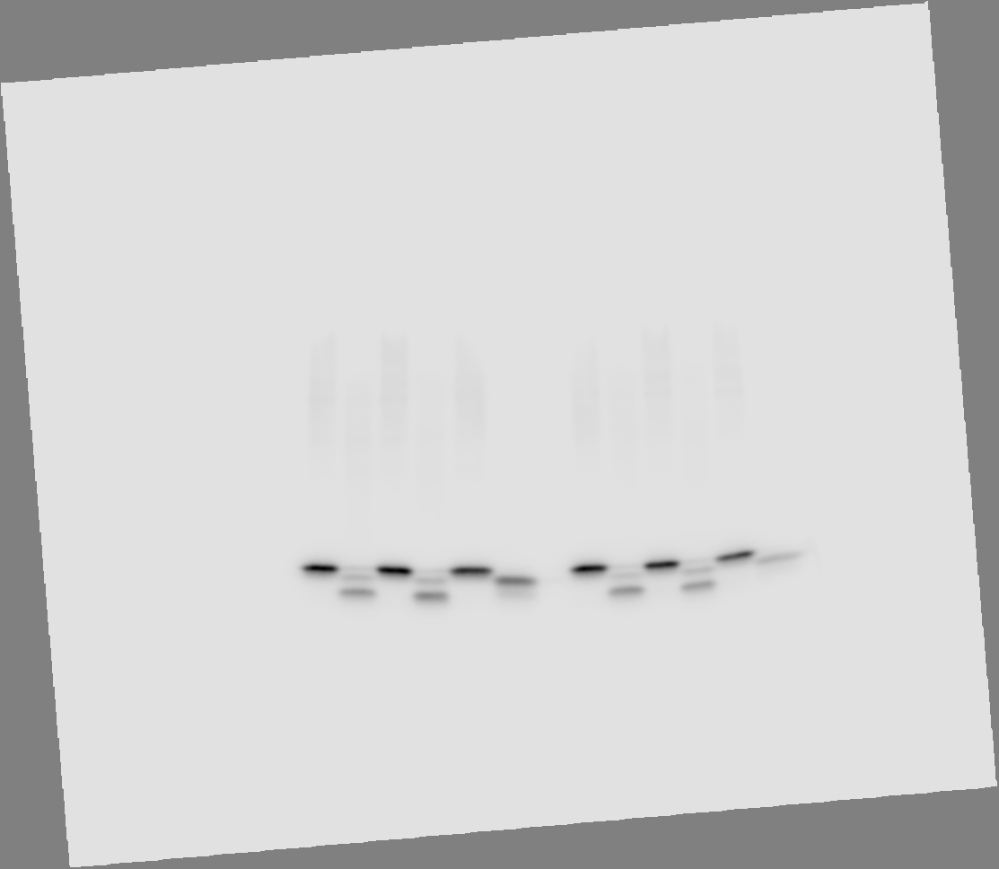

Supplement: Figure 5—source data 2. [file elife-84330-fig5-data2.zip › Figure 5-source data 1/5D Ant1 anti-Tom20.tif]

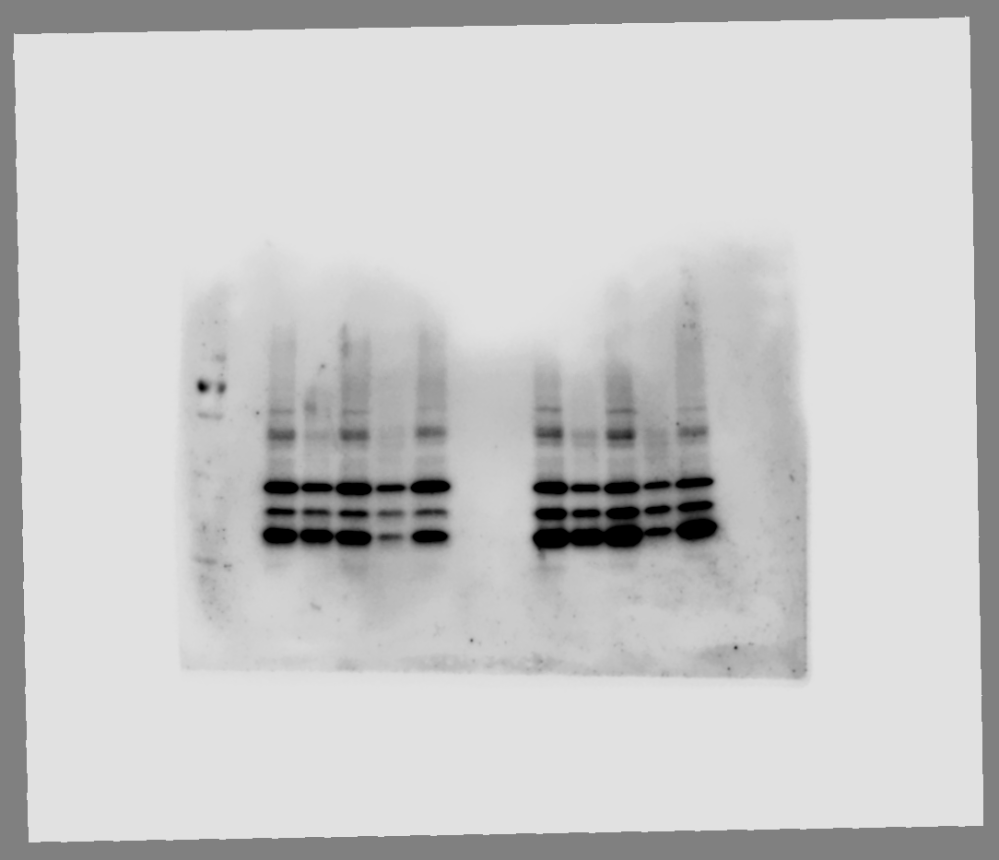

Supplement: Figure 5—source data 2. [file elife-84330-fig5-data2.zip › Figure 5-source data 1/5D Ant1_A114P anti-TFAM.tif]

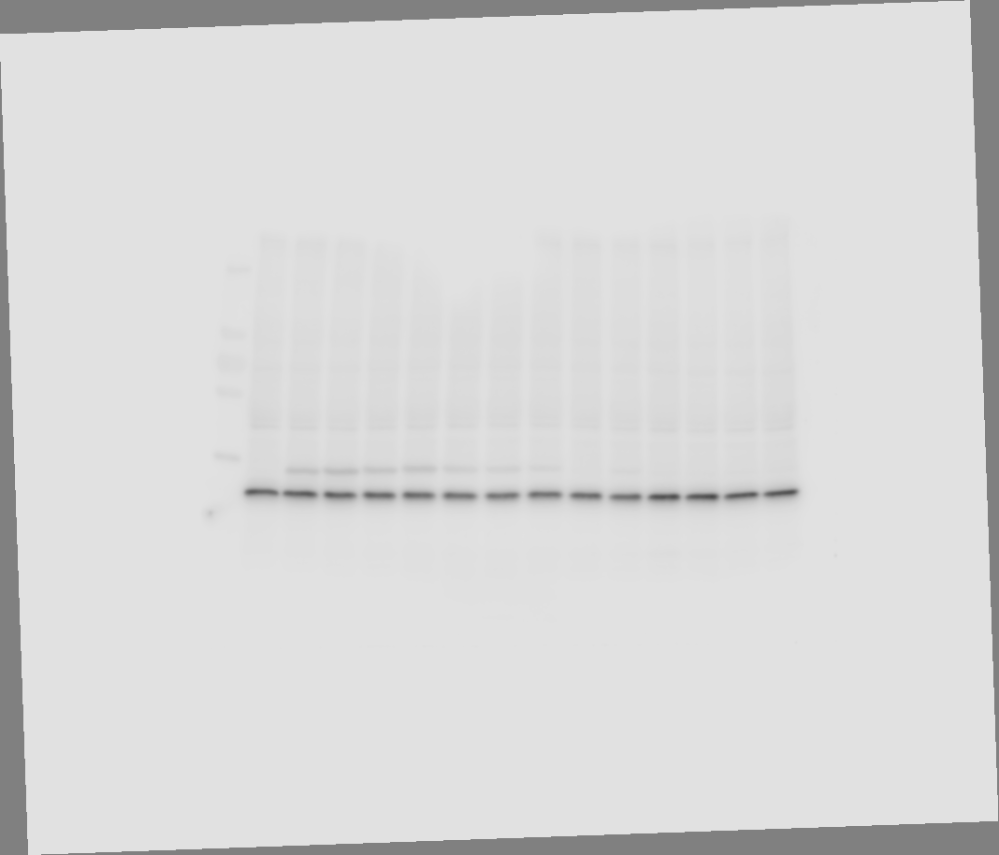

Supplement: Figure 5—source data 2. [file elife-84330-fig5-data2.zip › Figure 5-source data 1/5A TFAM.tif]

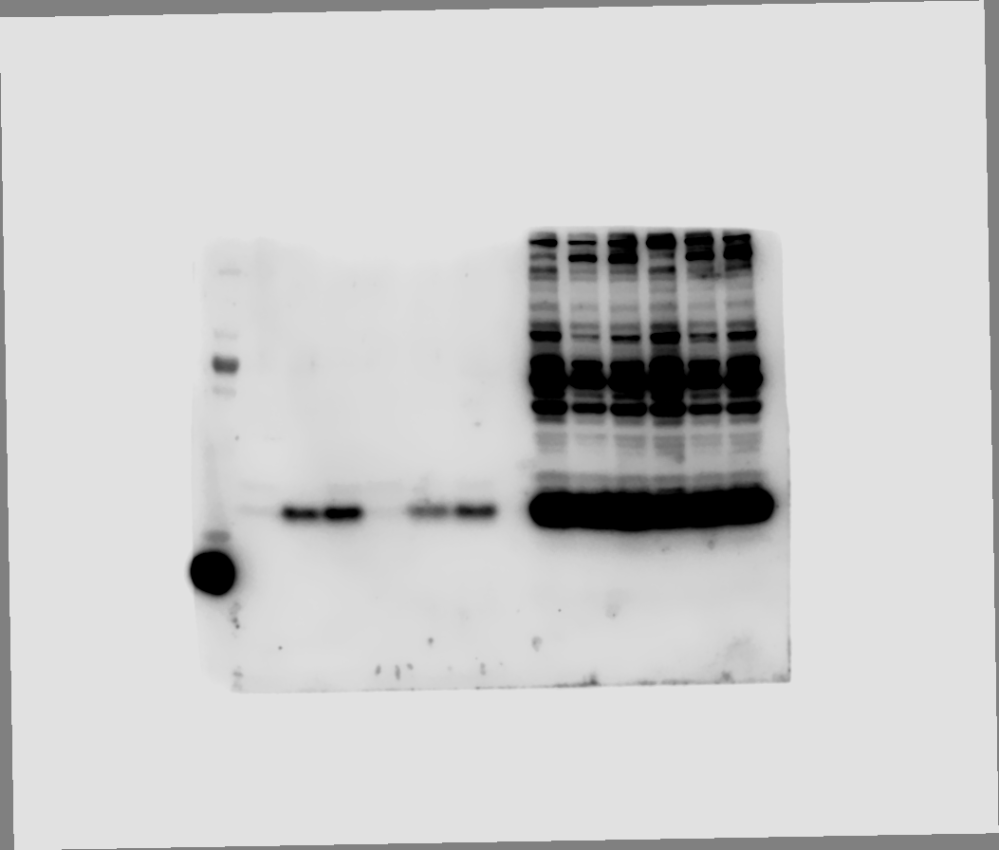

Supplement: Figure 5—source data 2. [file elife-84330-fig5-data2.zip › Figure 5-source data 1/5B IP Tim22.tif]

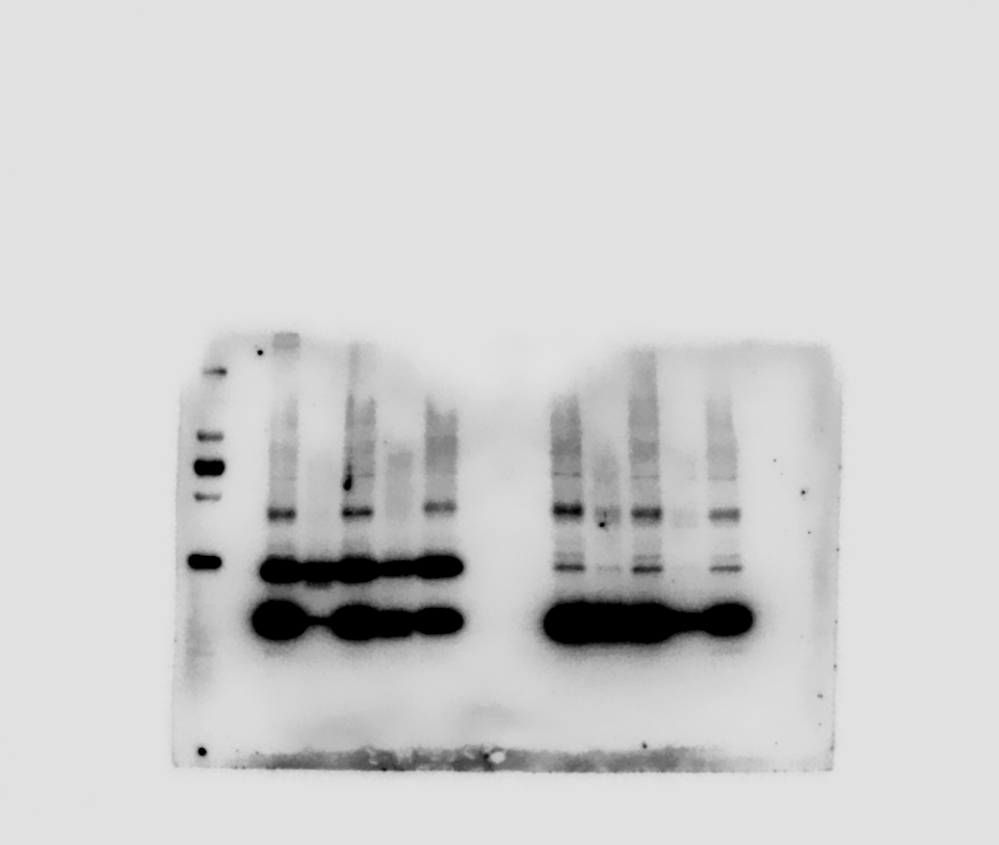

Supplement: Figure 5—source data 2. [file elife-84330-fig5-data2.zip › Figure 5-source data 1/5D Ant1_A114P_A123D anti-HA.tif]

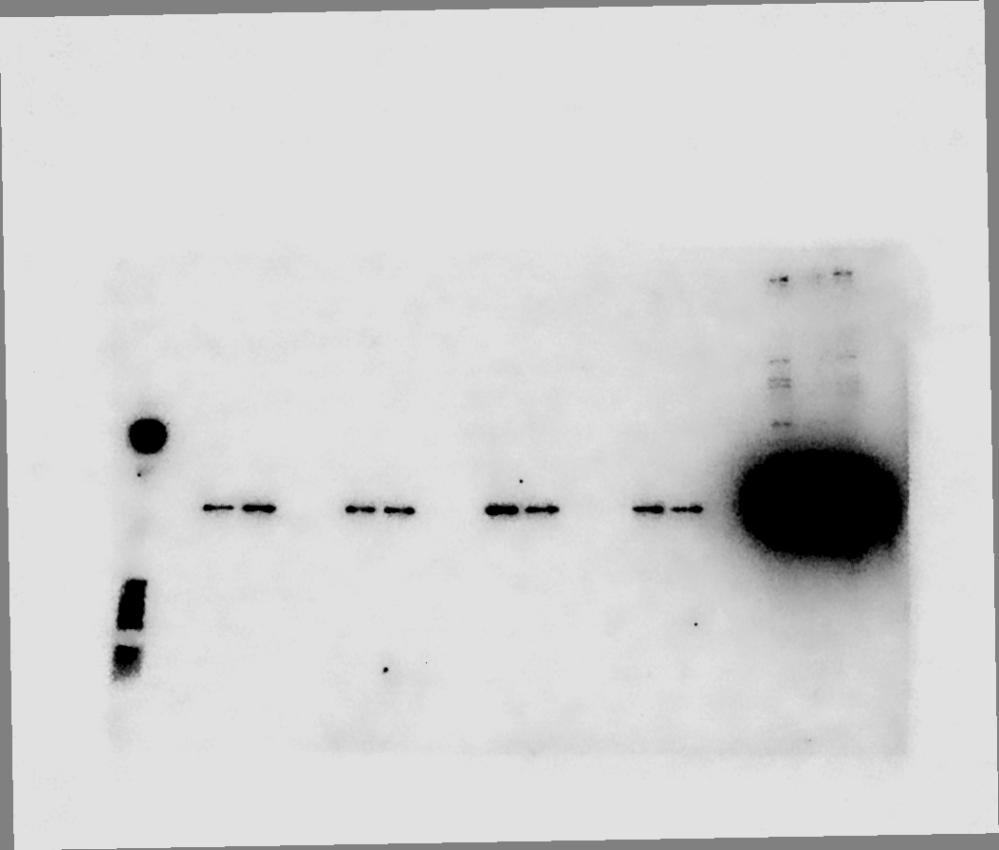

Supplement: Figure 5—source data 2. [file elife-84330-fig5-data2.zip › Figure 5-source data 1/5B IP Tom40.tif]

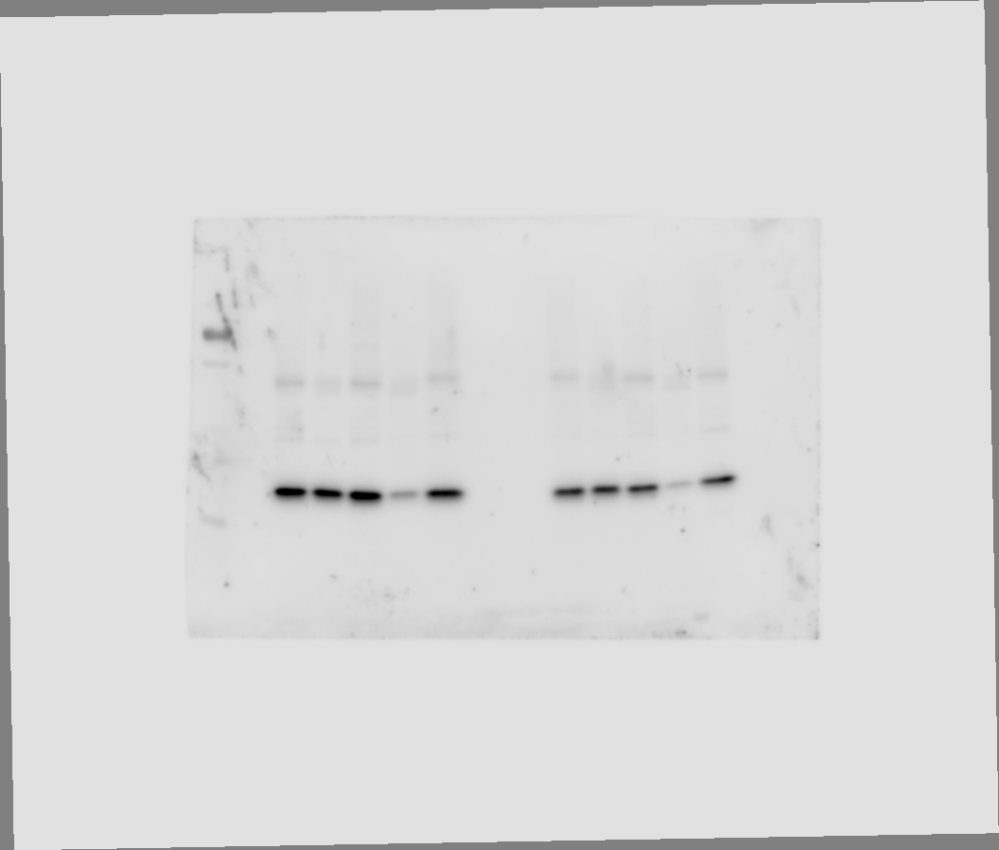

Supplement: Figure 5—source data 2. [file elife-84330-fig5-data2.zip › Figure 5-source data 1/5D Ant1 anti-Smac.tif]

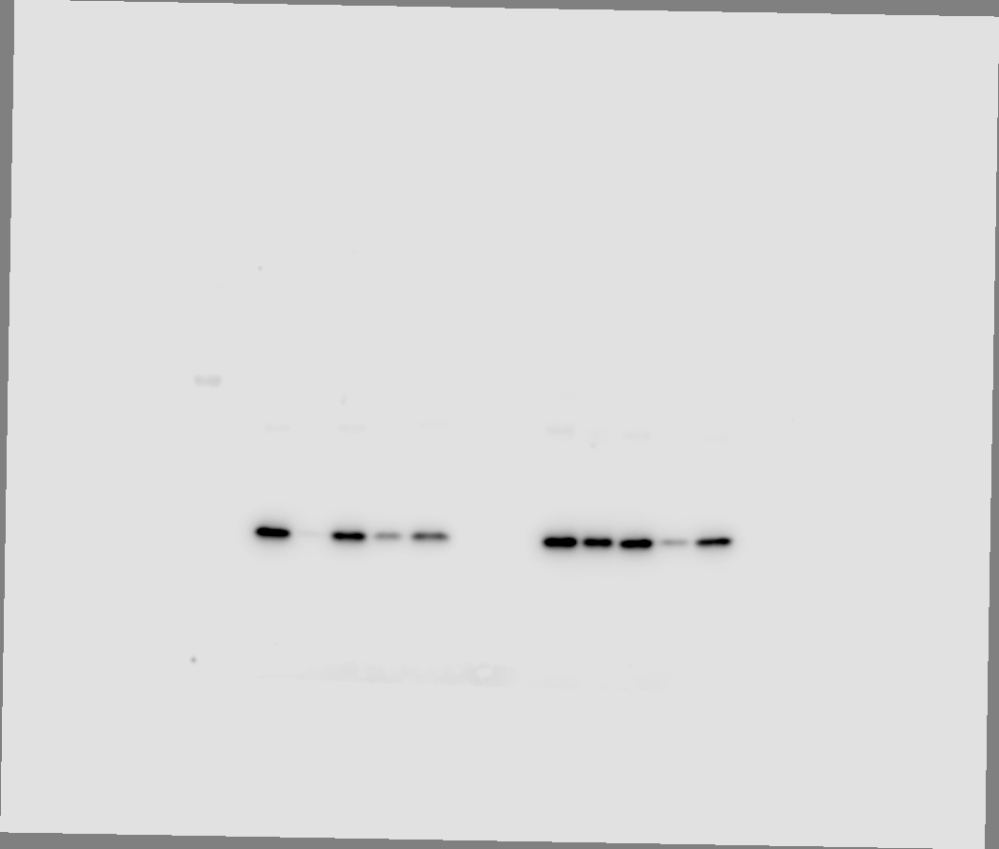

Supplement: Figure 5—source data 2. [file elife-84330-fig5-data2.zip › Figure 5-source data 1/5D Ant1_A114P_A123D anti-Smac.tif]

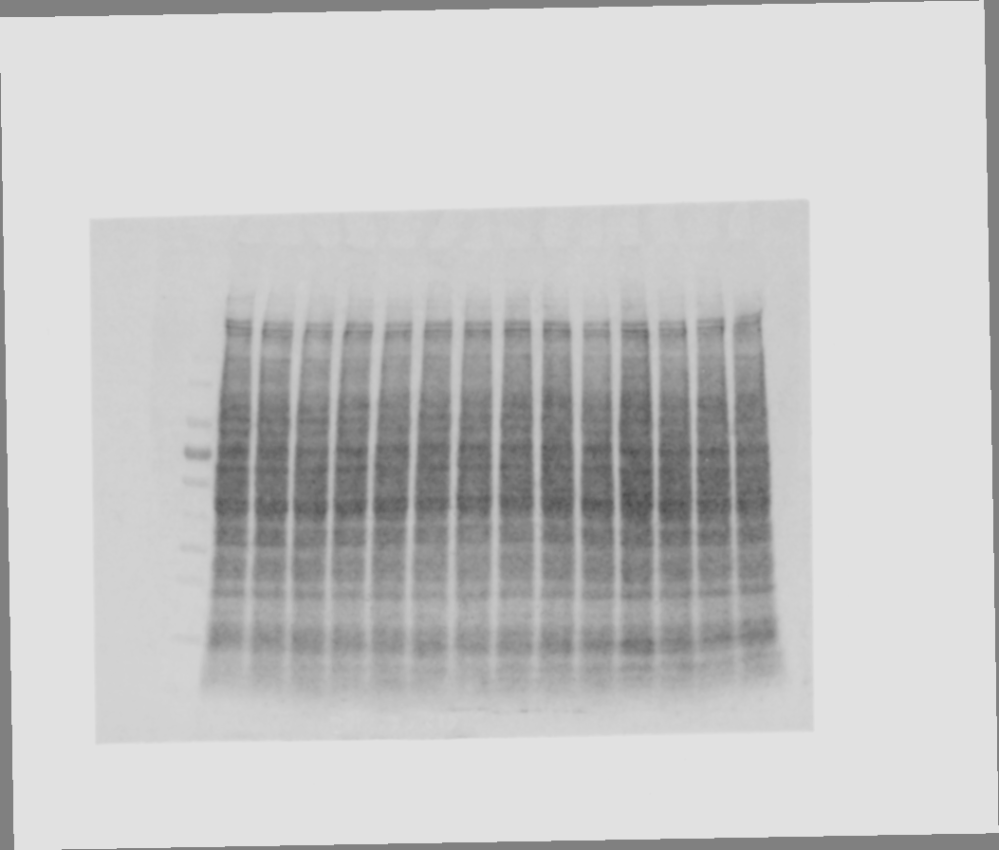

Supplement: Figure 5—source data 2. [file elife-84330-fig5-data2.zip › Figure 5-source data 1/5A TPS.tif]

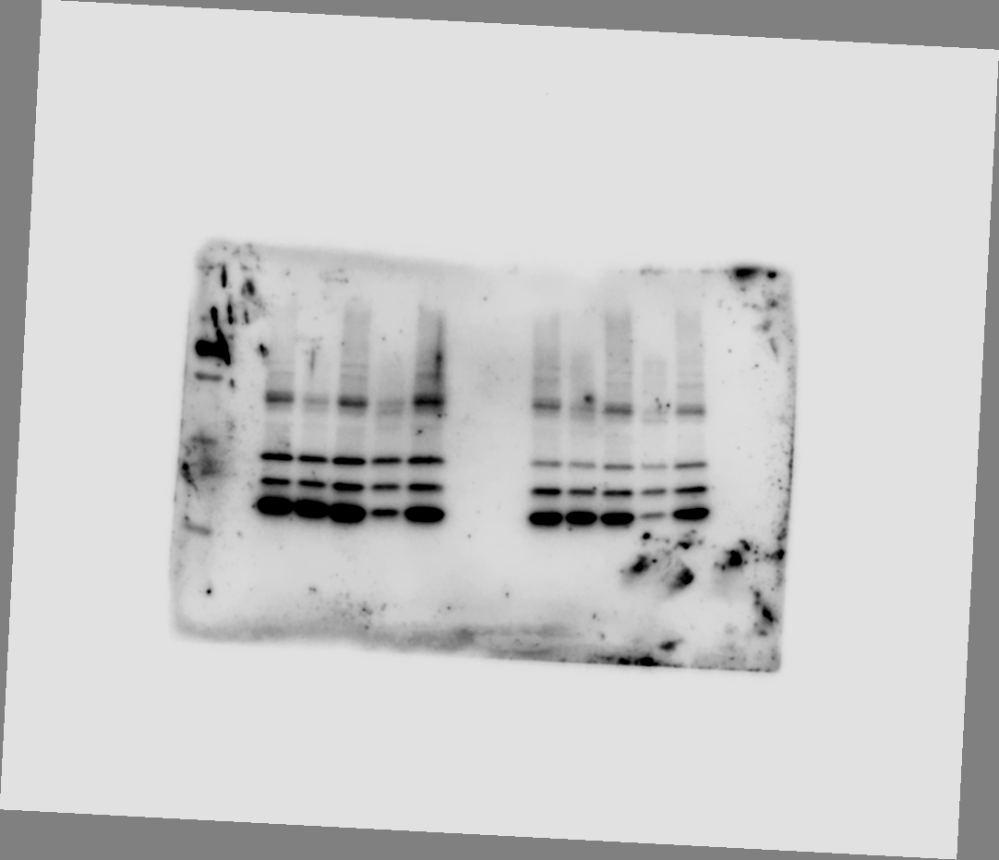

Supplement: Figure 5—source data 2. [file elife-84330-fig5-data2.zip › Figure 5-source data 1/5D Ant1_A123D anti-TFAM.tif]

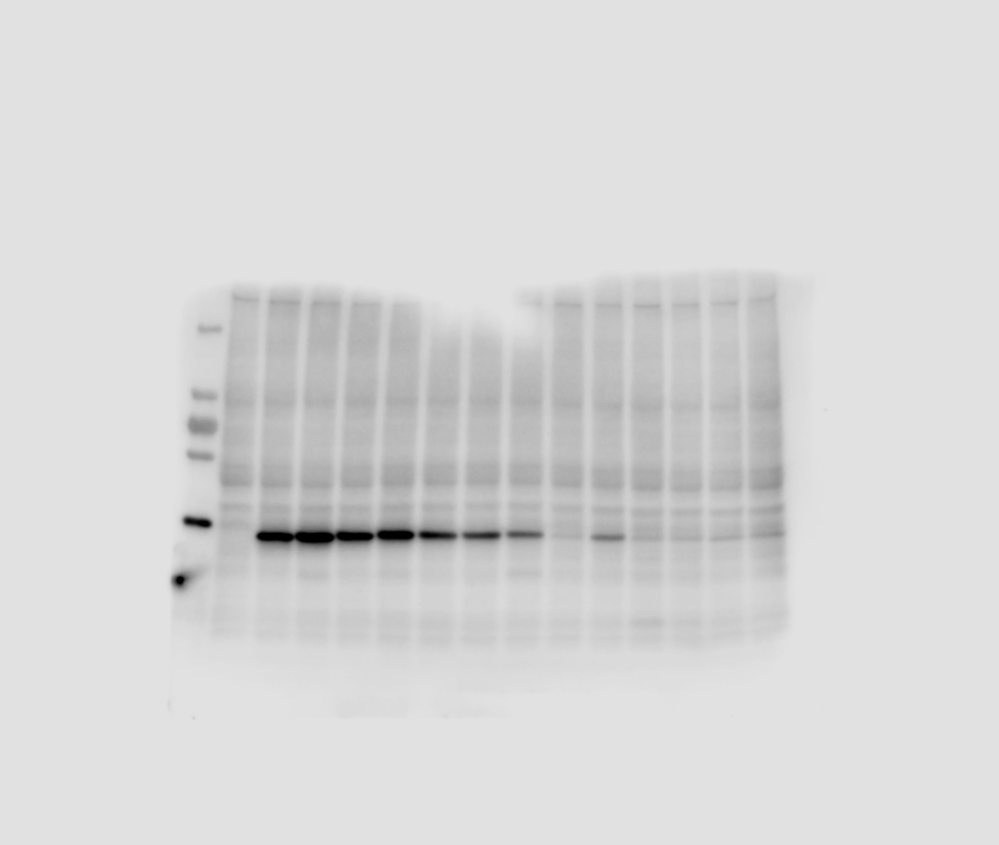

Supplement: Figure 5—source data 2. [file elife-84330-fig5-data2.zip › Figure 5-source data 1/5A HA.tif]

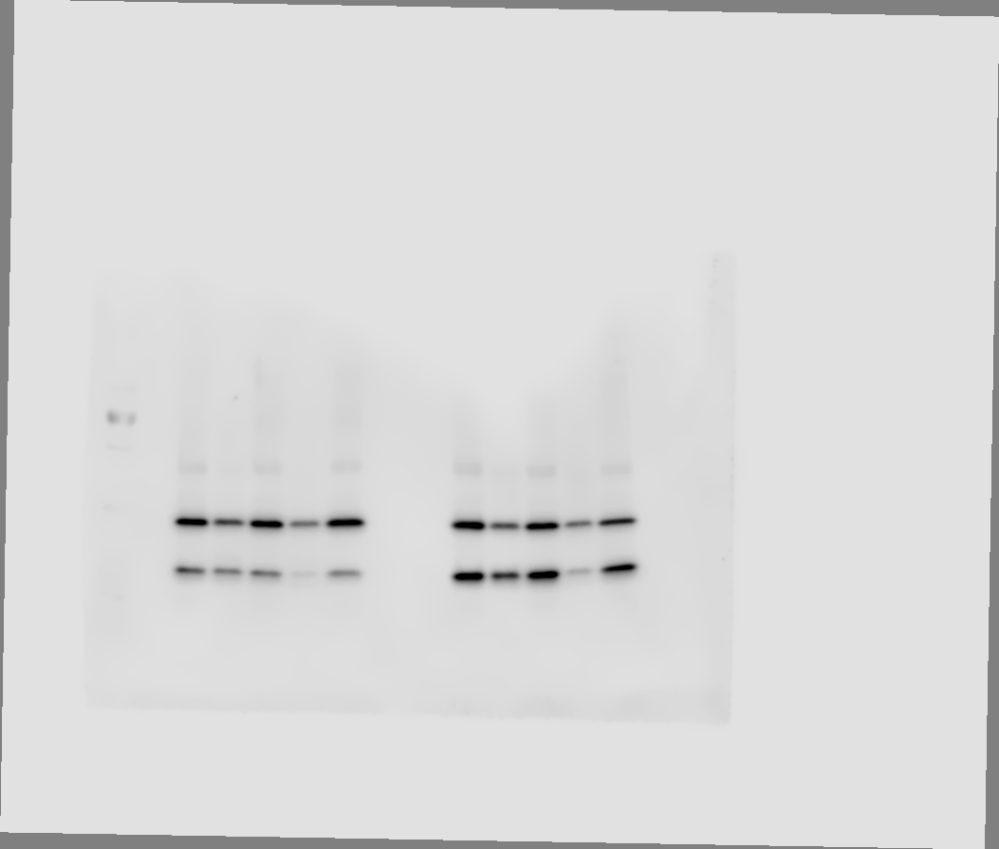

Supplement: Figure 5—source data 2. [file elife-84330-fig5-data2.zip › Figure 5-source data 1/5D Ant1_A114P anti-HA.tif]

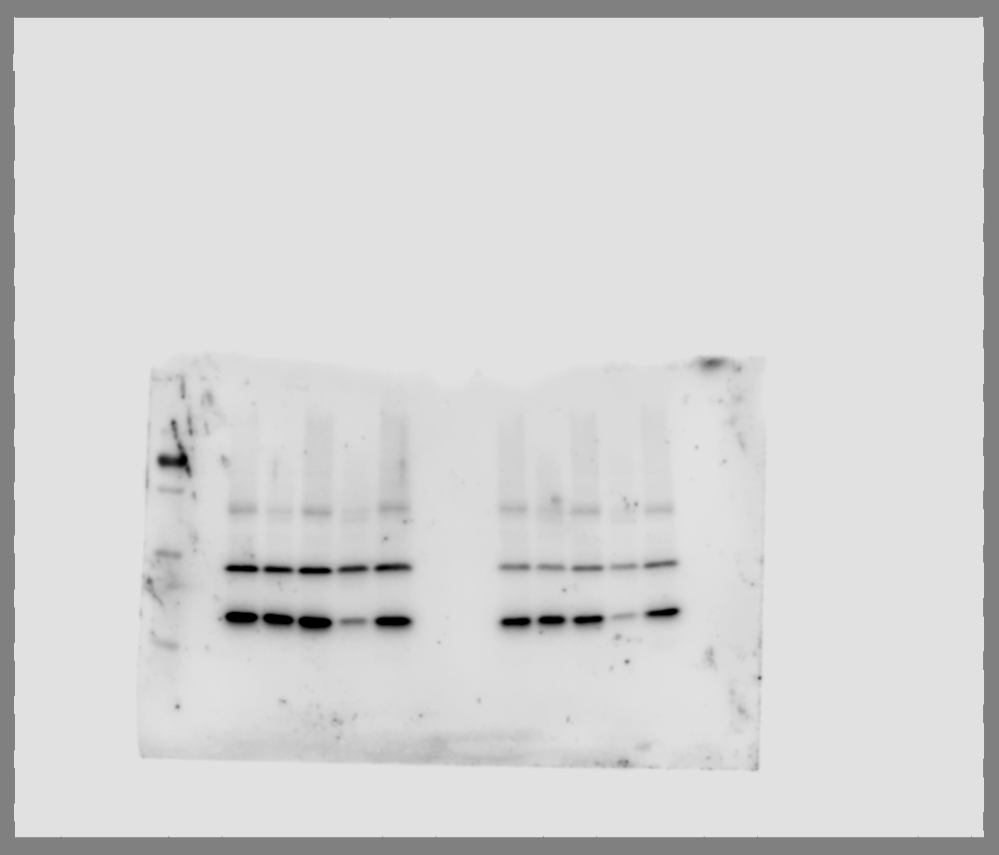

Supplement: Figure 5—source data 2. [file elife-84330-fig5-data2.zip › Figure 5-source data 1/5D Ant1 anti-HA.tif]

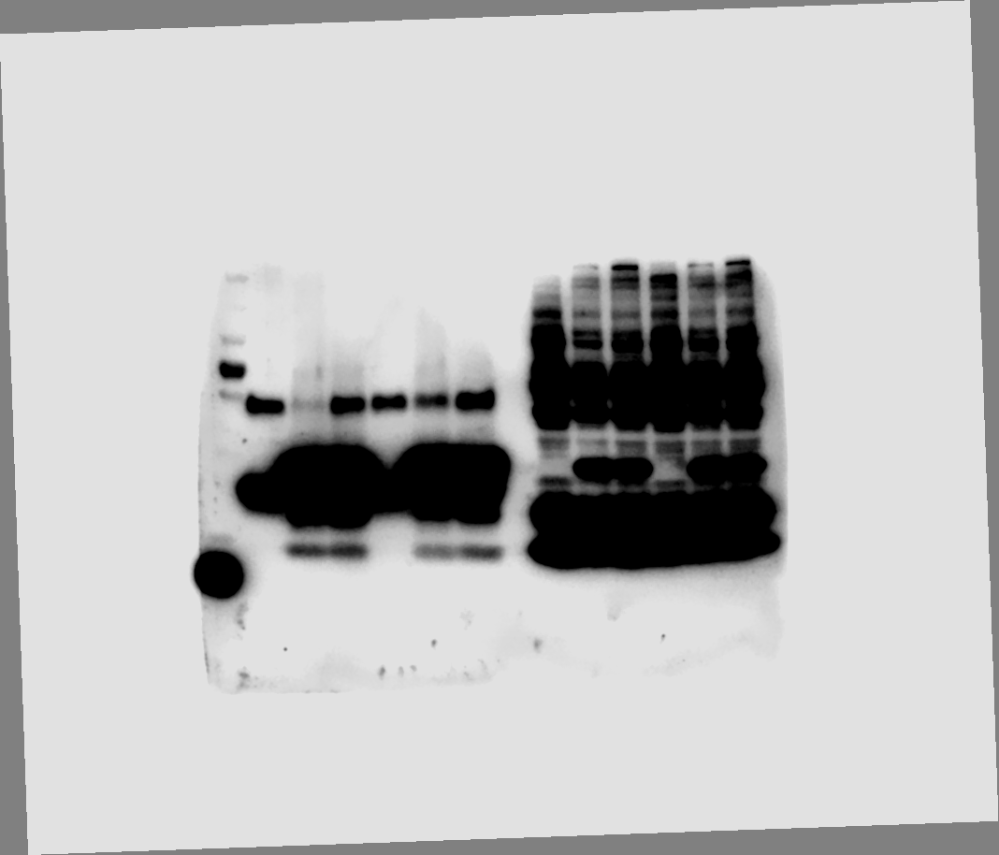

Supplement: Figure 5—source data 2. [file elife-84330-fig5-data2.zip › Figure 5-source data 1/5B IP Tom20.tif]

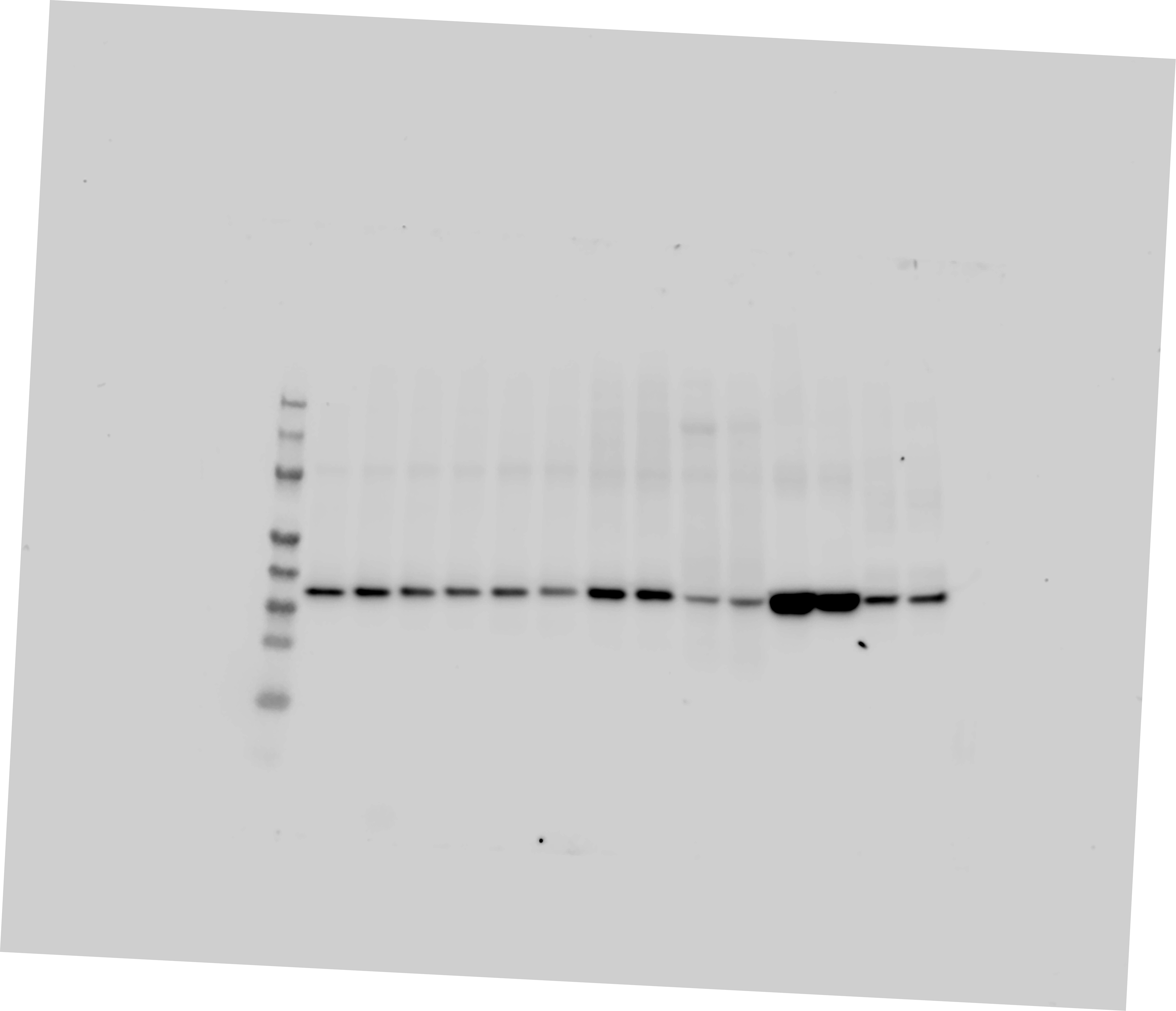

Supplement: Figure 6—source data 1. [file elife-84330-fig6-data1.zip › Figure 6-source data 1/Fig6F_GAPDH.tif]

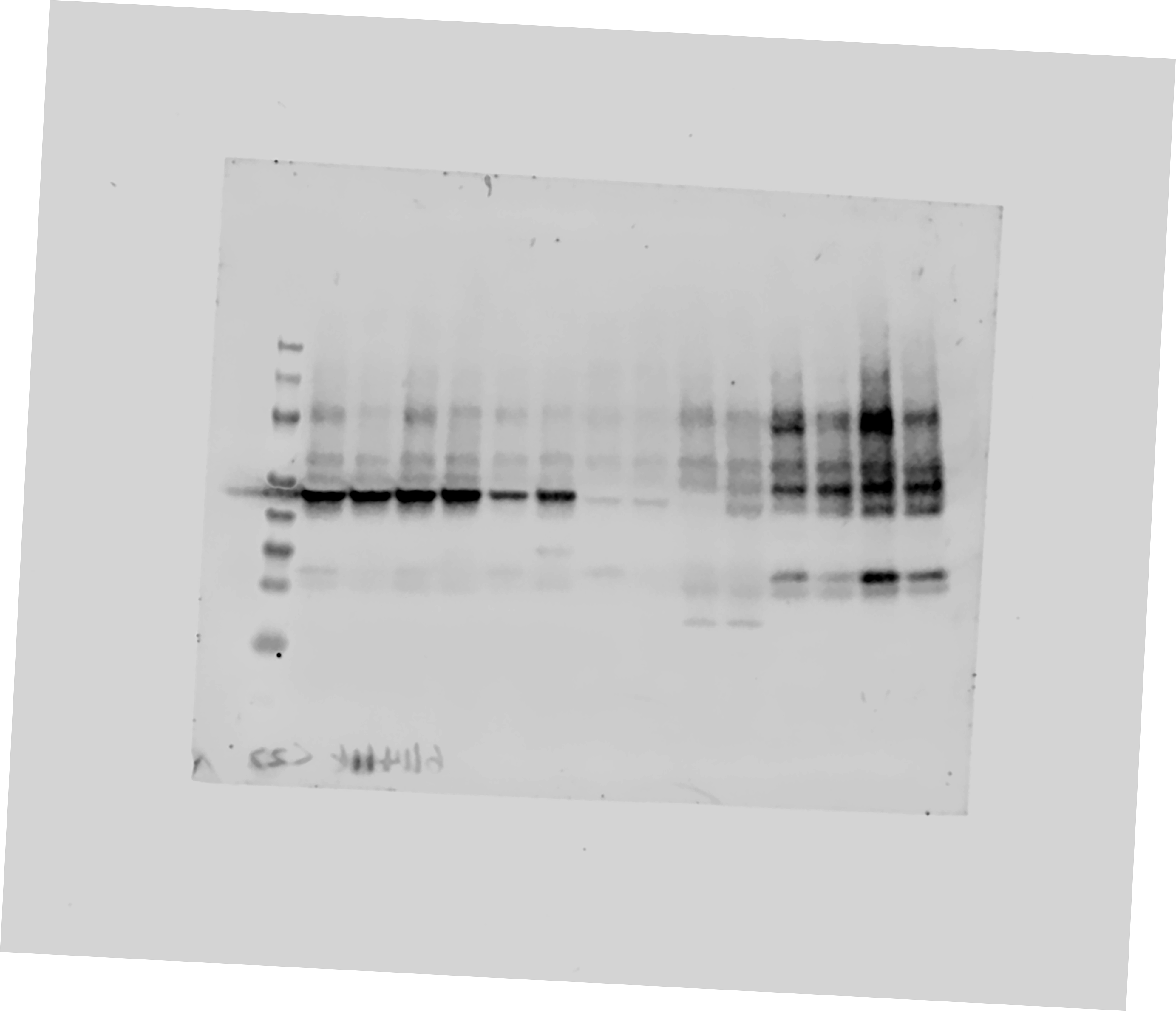

Supplement: Figure 6—source data 1. [file elife-84330-fig6-data1.zip › Figure 6-source data 1/Fig6F_GFAP.tif]

40 kDa

35 kDa

Cropped area for Figure 6F GAPDH

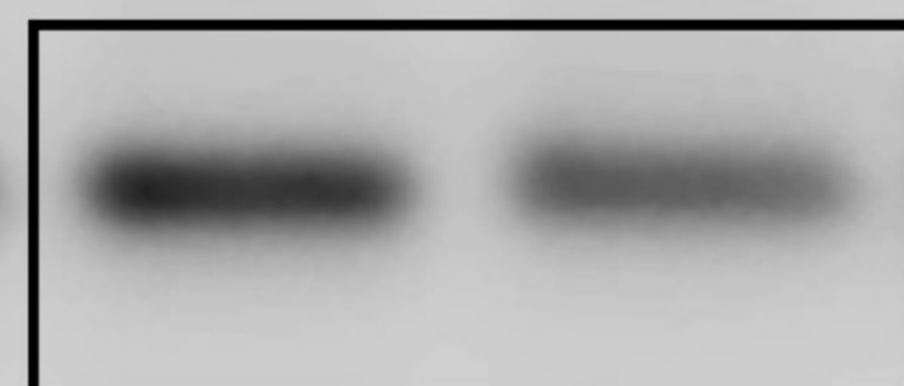

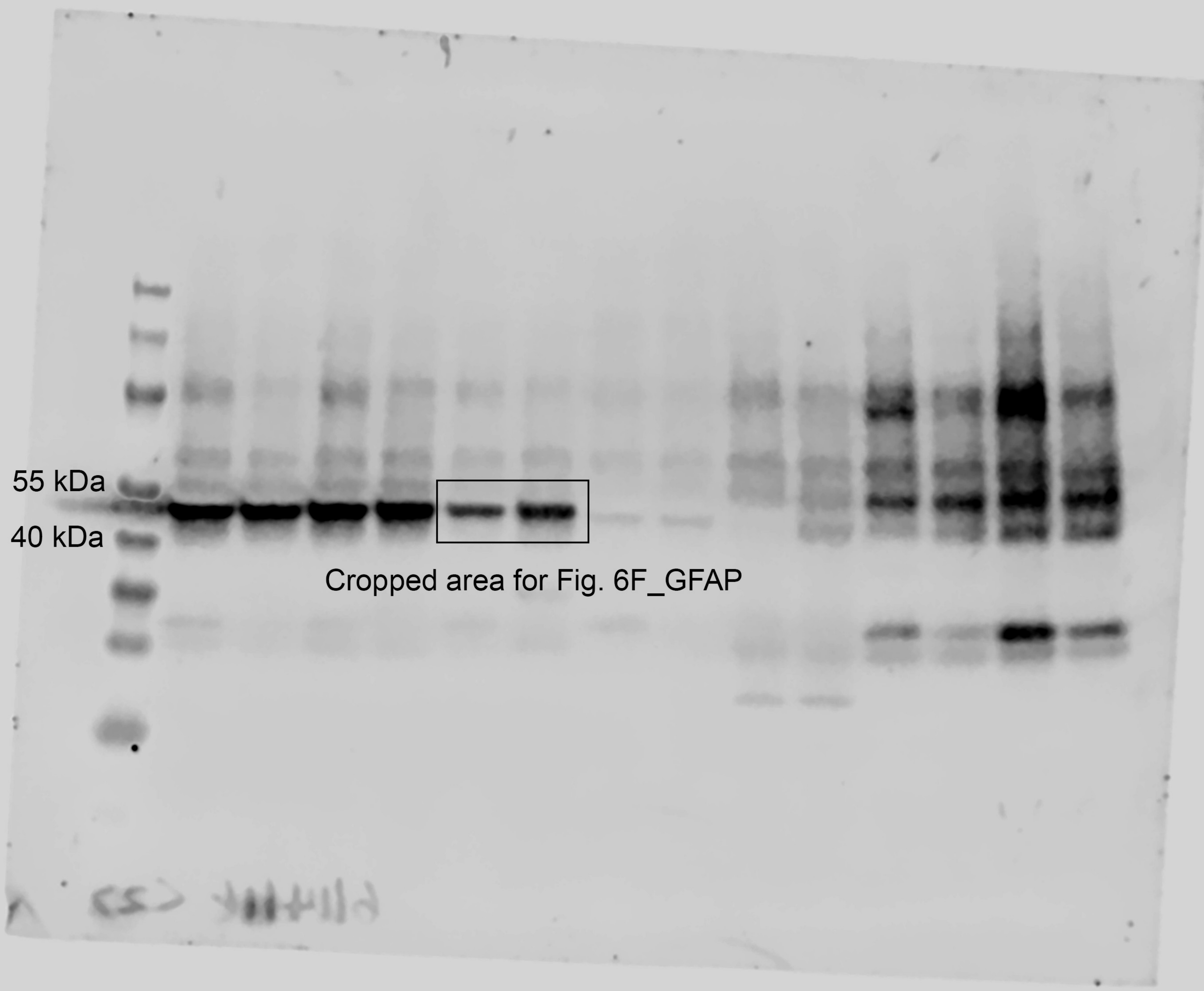

Supplement: Figure 6—source data 1. [file elife-84330-fig6-data1.zip › Figure 6-source data 1/Figure 6-source data_annotated.pdf]

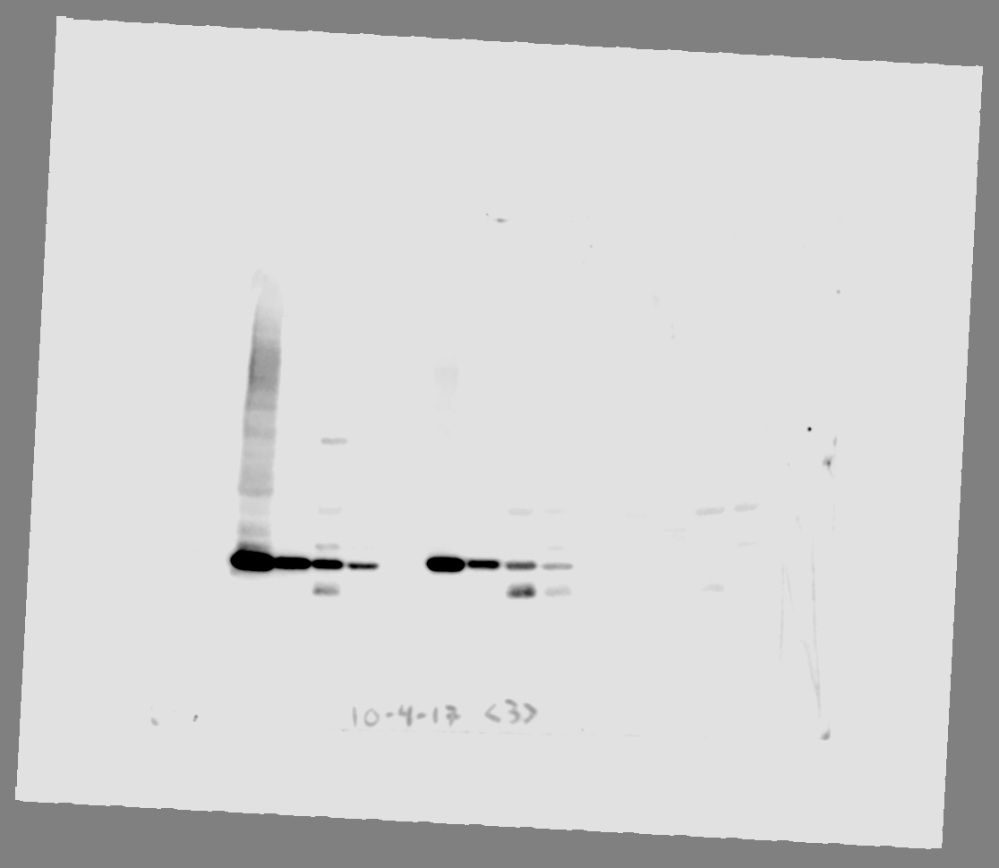

Supplement: Figure 8—source data 2. [file elife-84330-fig8-data2.zip › Figure 8-source data 1/8A Ant1.tif]

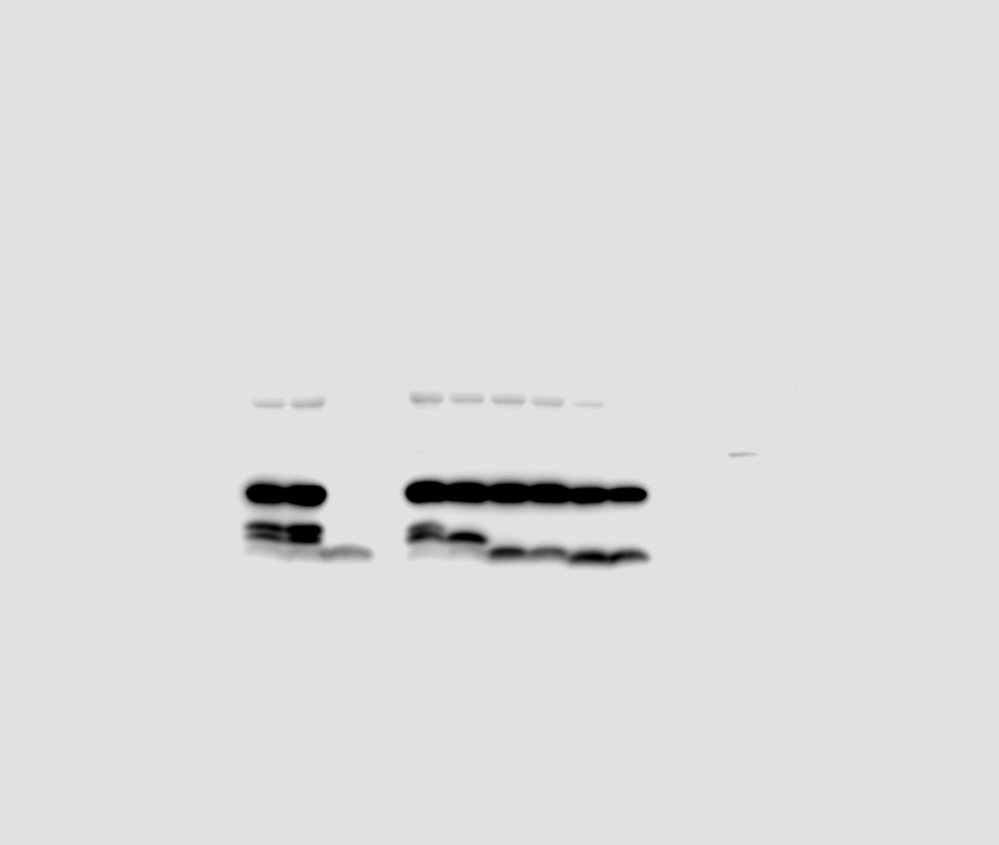

Supplement: Figure 8—source data 2. [file elife-84330-fig8-data2.zip › Figure 8-source data 1/8D Homozygous clogger mito Tom20.tif]

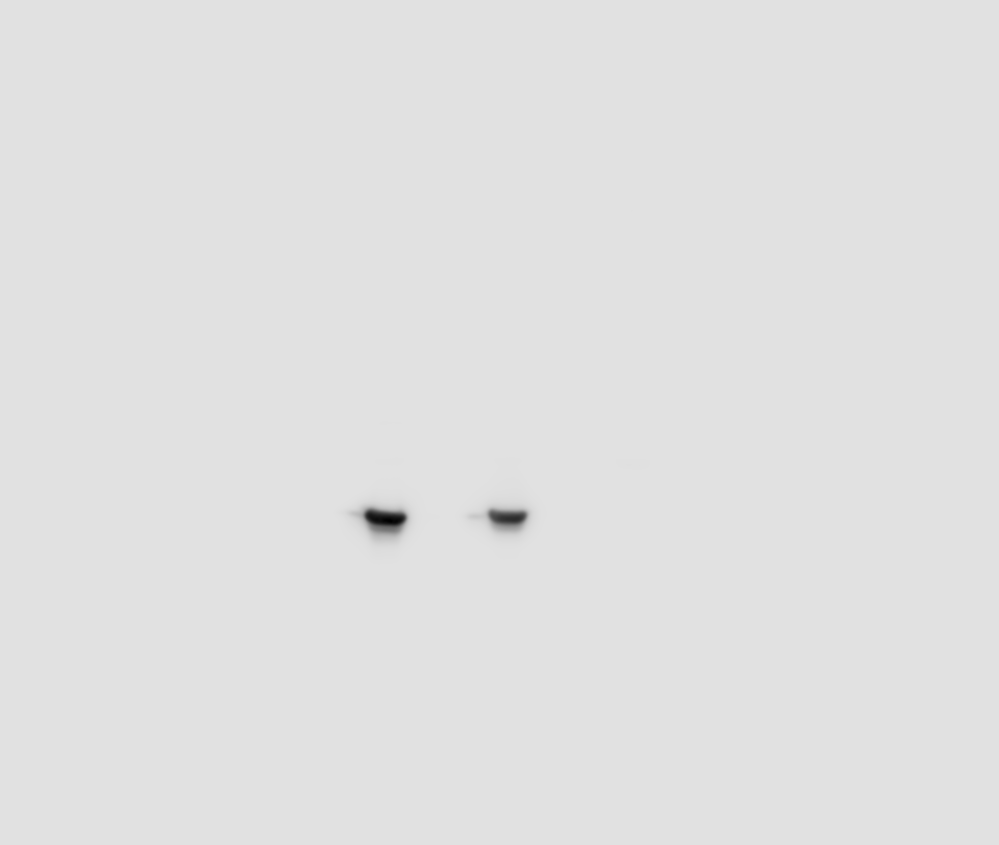

Supplement: Figure 8—source data 2. [file elife-84330-fig8-data2.zip › Figure 8-source data 1/8B Ant1 short.tif]

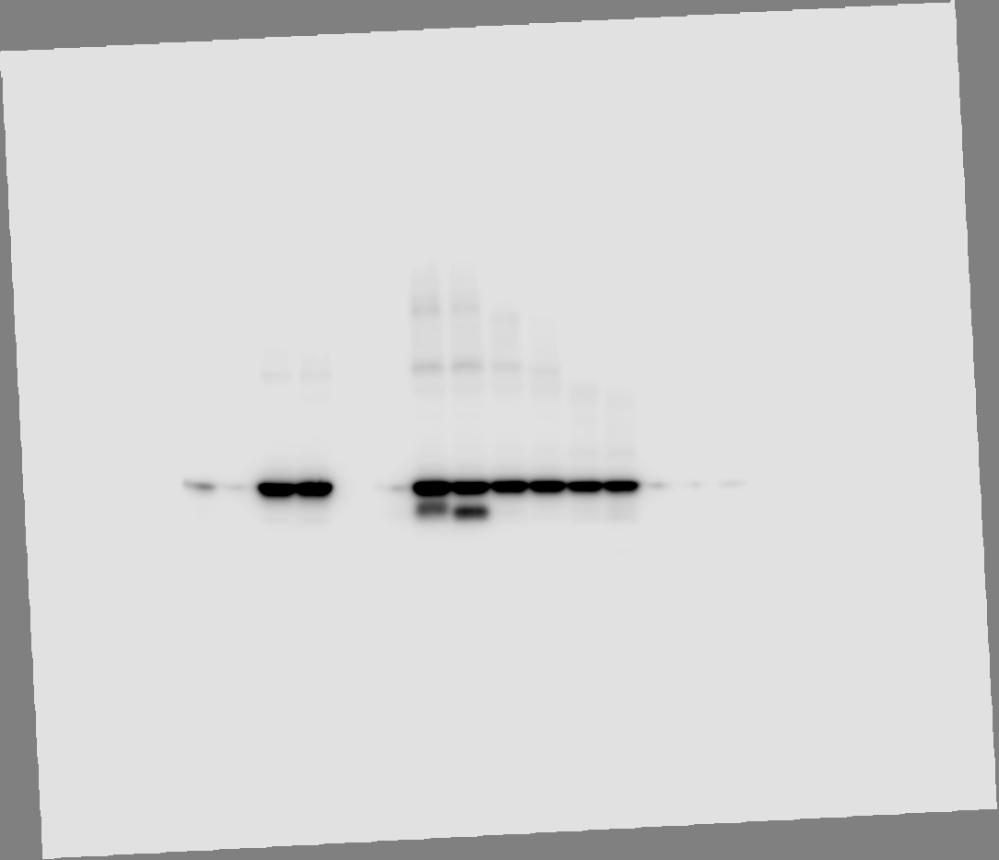

Supplement: Figure 8—source data 2. [file elife-84330-fig8-data2.zip › Figure 8-source data 1/8D Homozygous clogger mito Tim23.tif]

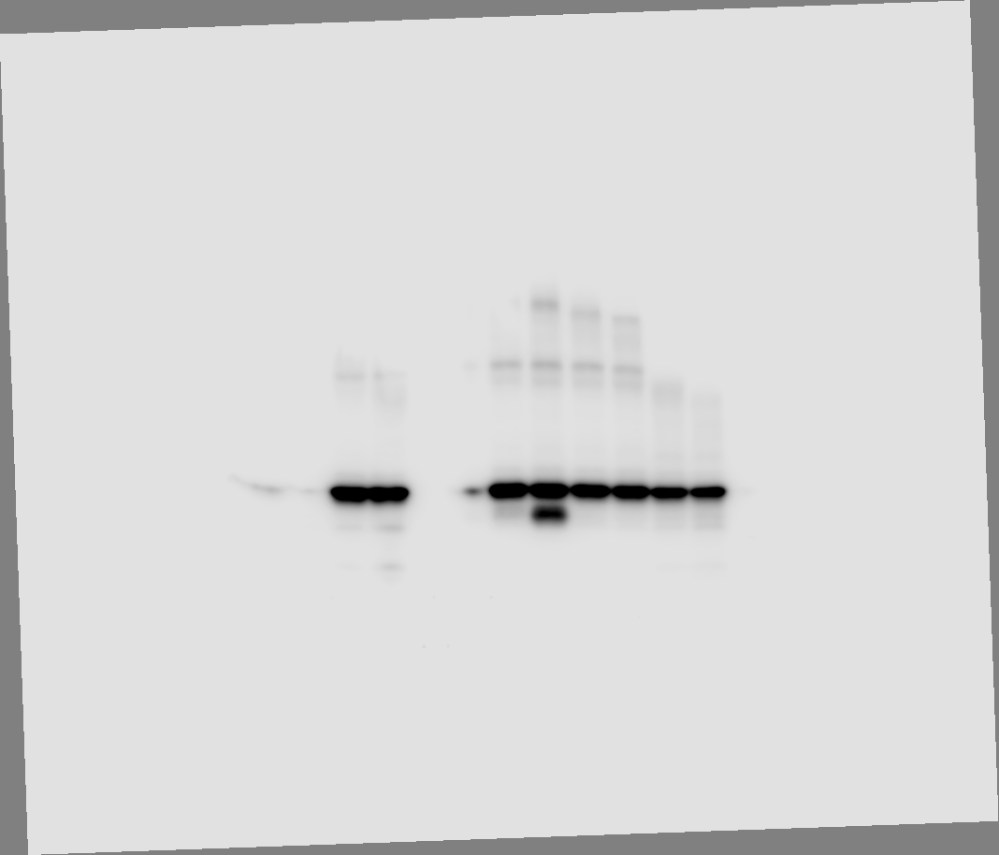

Supplement: Figure 8—source data 2. [file elife-84330-fig8-data2.zip › Figure 8-source data 1/8D WT mito Tim23.tif]

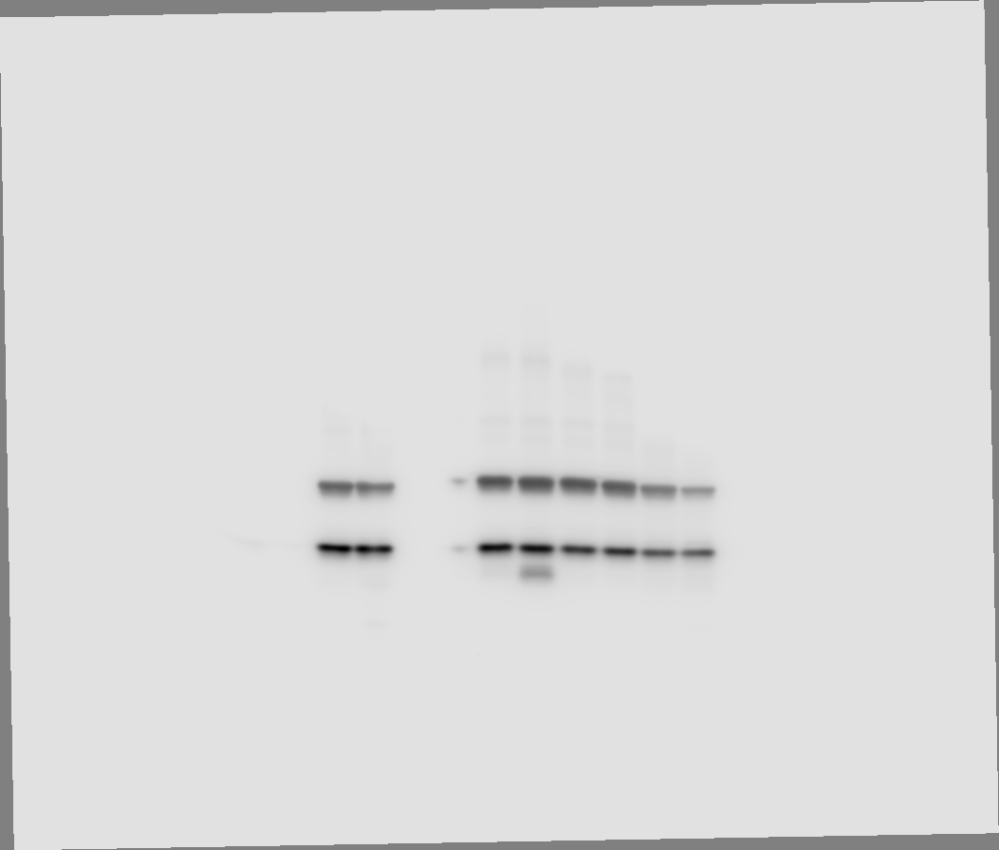

Supplement: Figure 8—source data 2. [file elife-84330-fig8-data2.zip › Figure 8-source data 1/8D WT mito MDH2.tif]

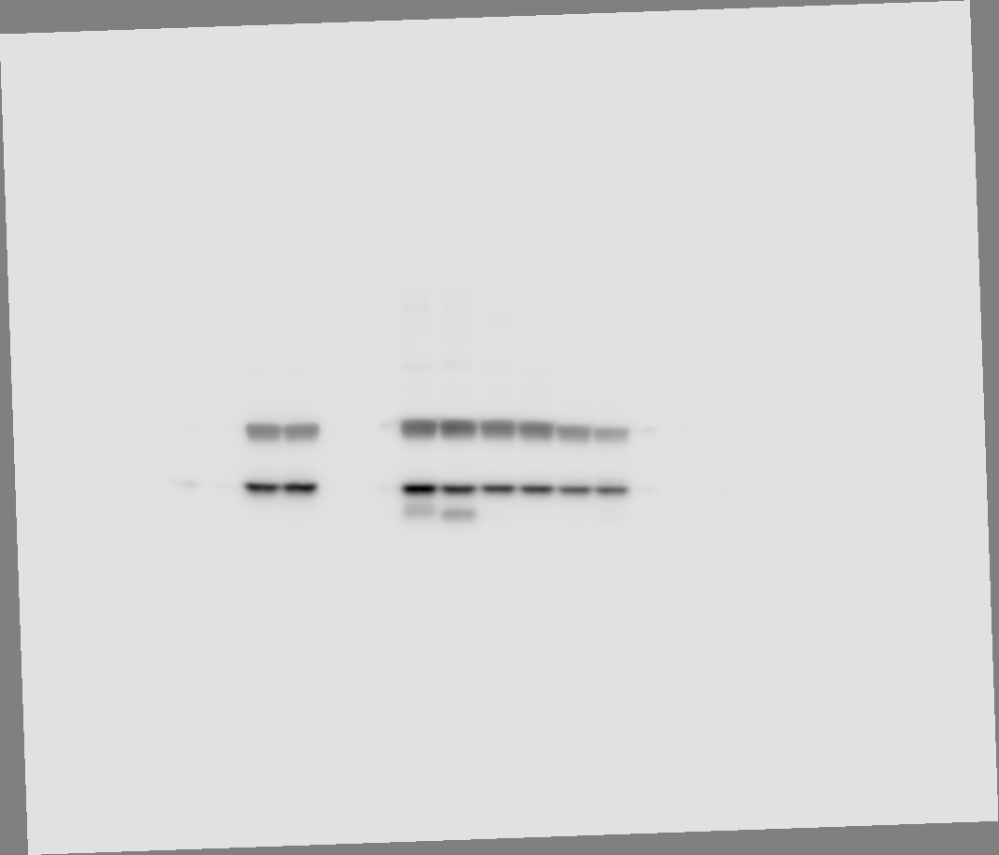

Supplement: Figure 8—source data 2. [file elife-84330-fig8-data2.zip › Figure 8-source data 1/8D Homozygous clogger mito Mdh2.tif]

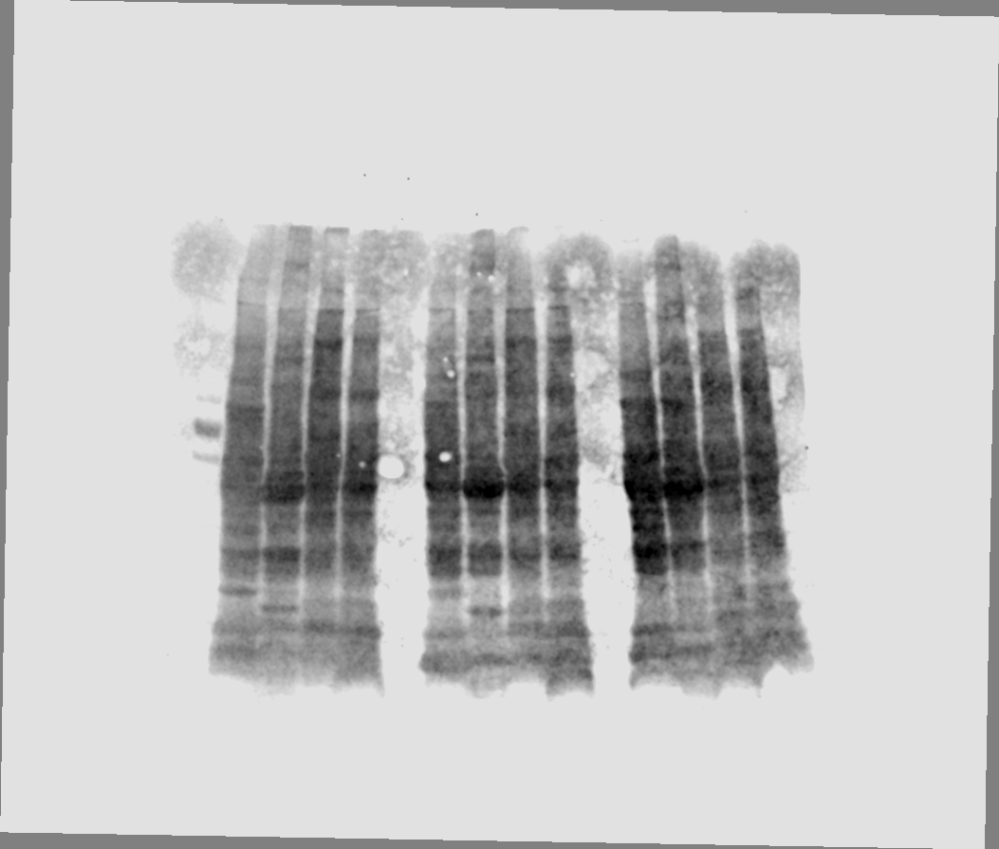

Supplement: Figure 8—source data 2. [file elife-84330-fig8-data2.zip › Figure 8-source data 1/8A TPS.tif]

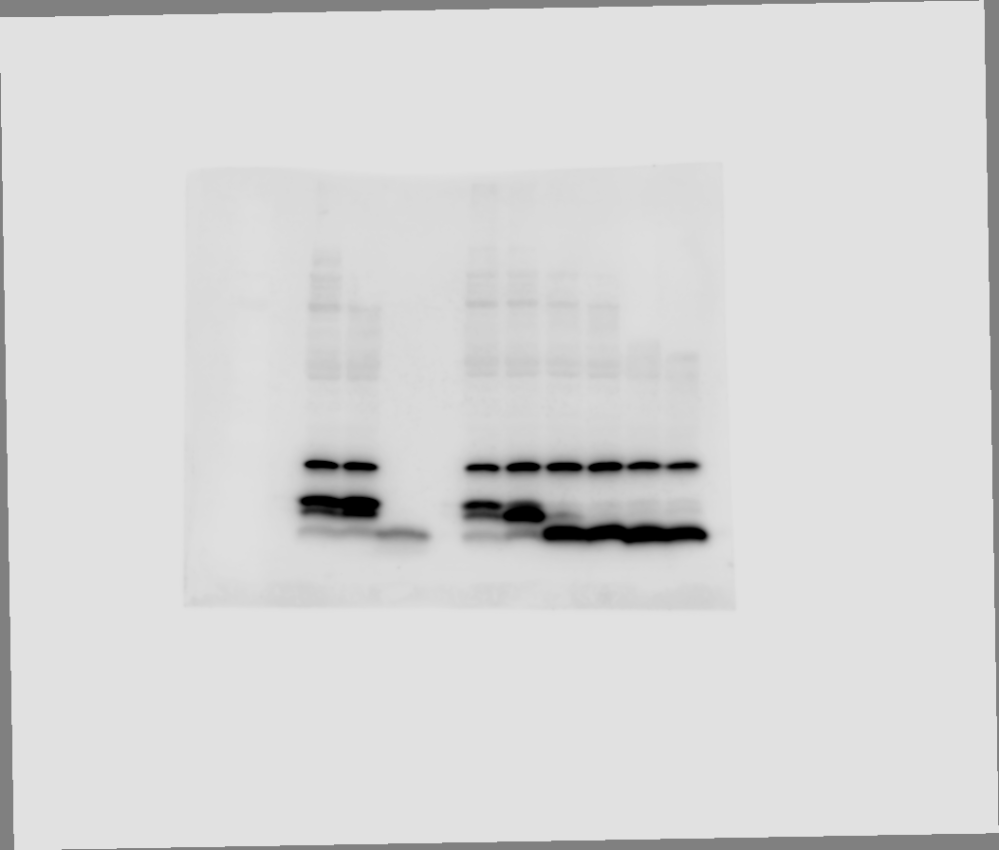

Supplement: Figure 8—source data 2. [file elife-84330-fig8-data2.zip › Figure 8-source data 1/8D WT mito Smac.tif]

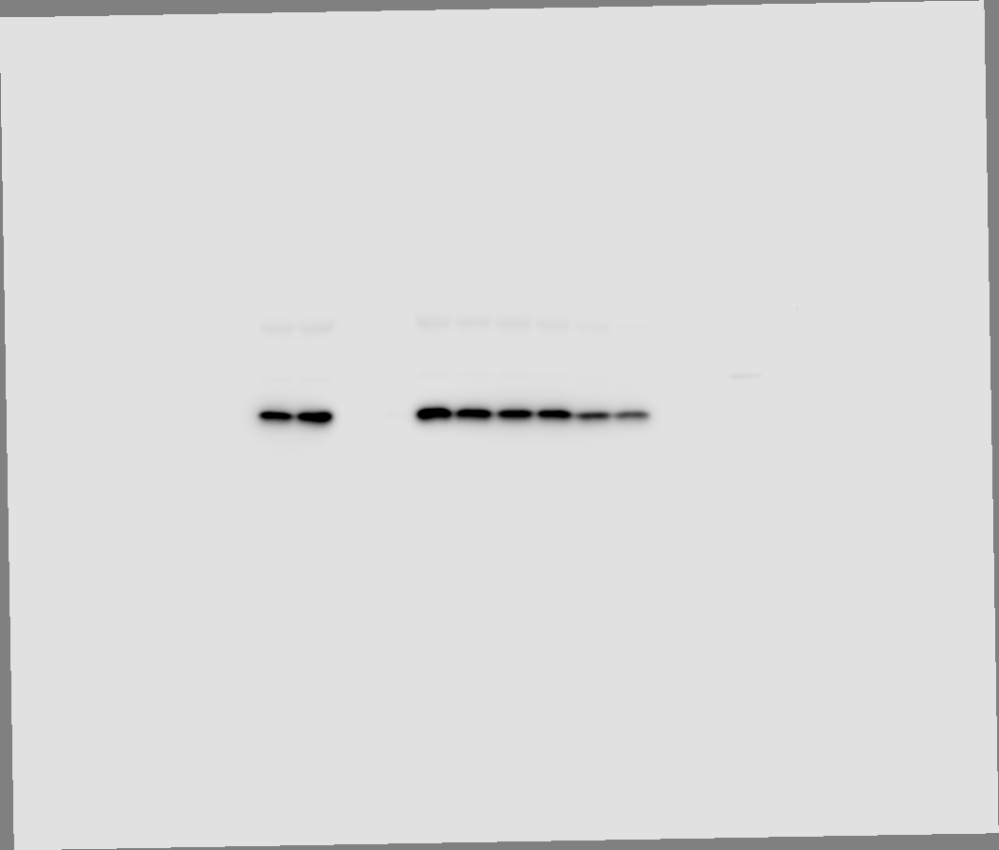

Supplement: Figure 8—source data 2. [file elife-84330-fig8-data2.zip › Figure 8-source data 1/8D Homozygous clogger mito SMAC.tif]

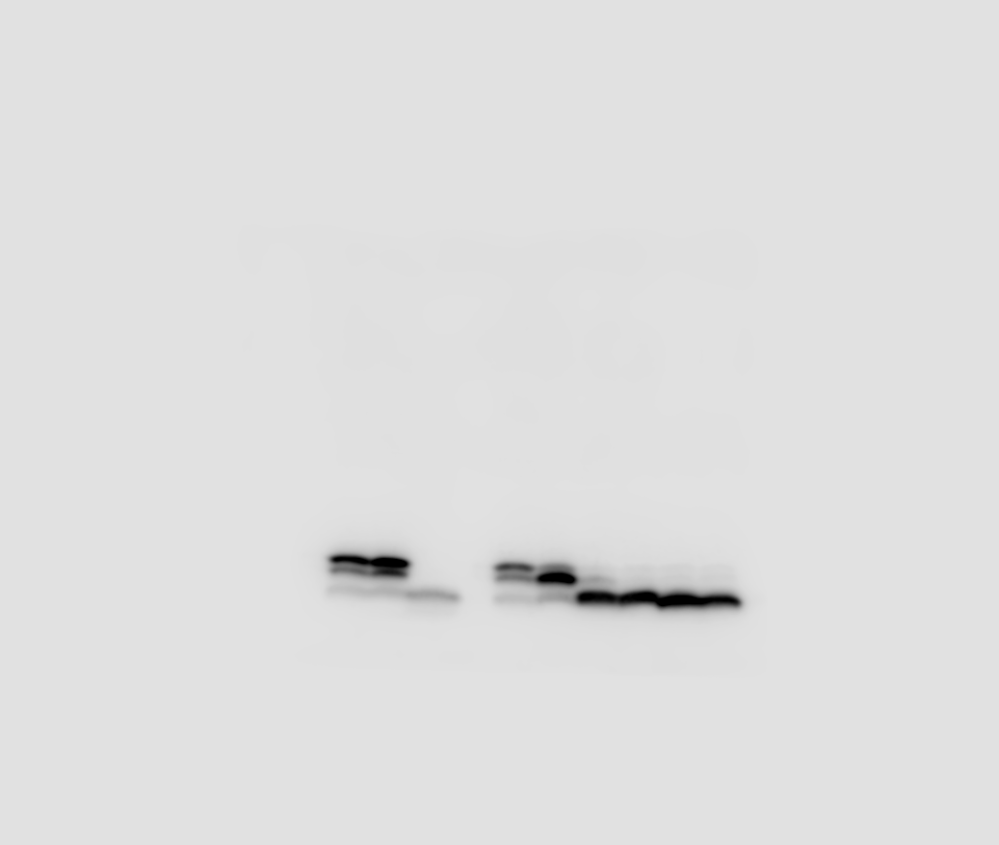

Supplement: Figure 8—source data 2. [file elife-84330-fig8-data2.zip › Figure 8-source data 1/8D WT mito Tom20.tif]

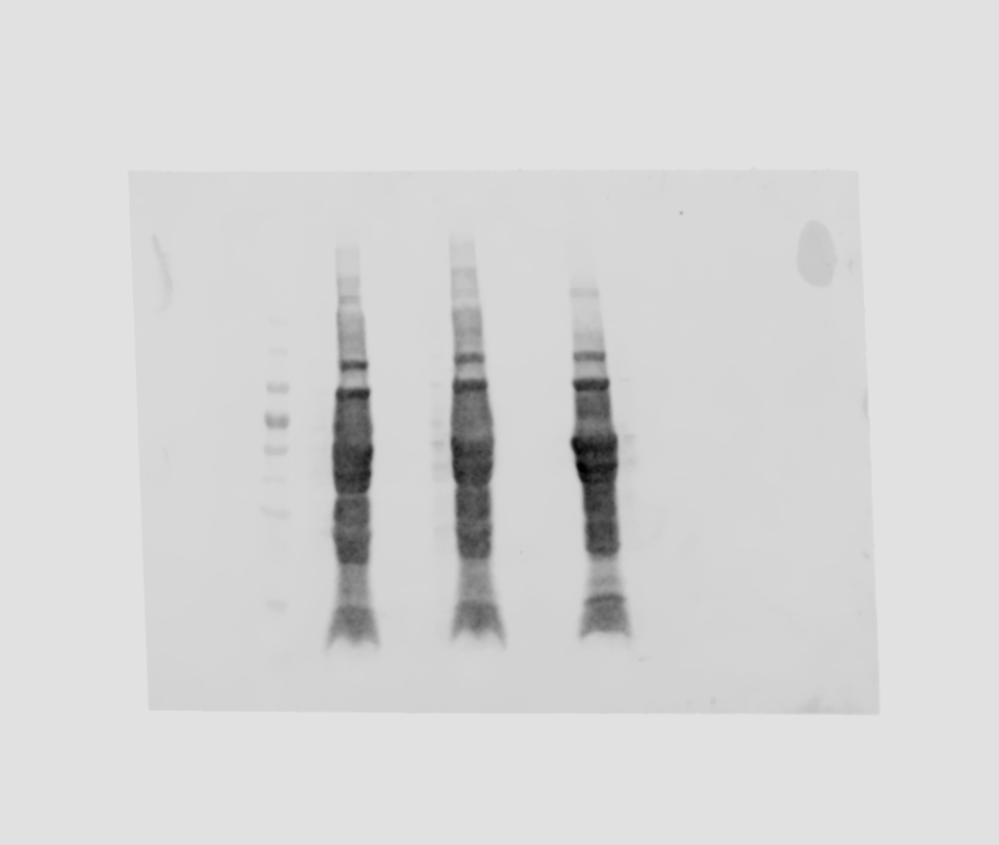

Supplement: Figure 8—source data 2. [file elife-84330-fig8-data2.zip › Figure 8-source data 1/8B TPS.tif]

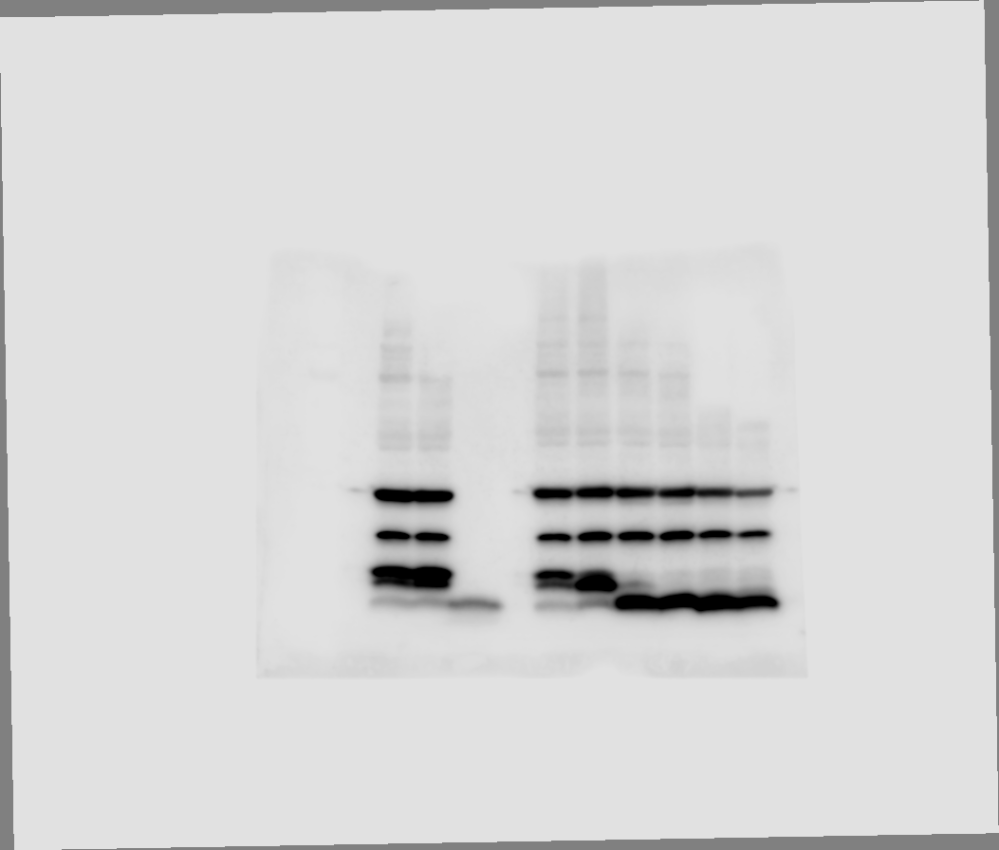

Supplement: Figure 8—source data 2. [file elife-84330-fig8-data2.zip › Figure 8-source data 1/8D WT mito Ant1.tif]

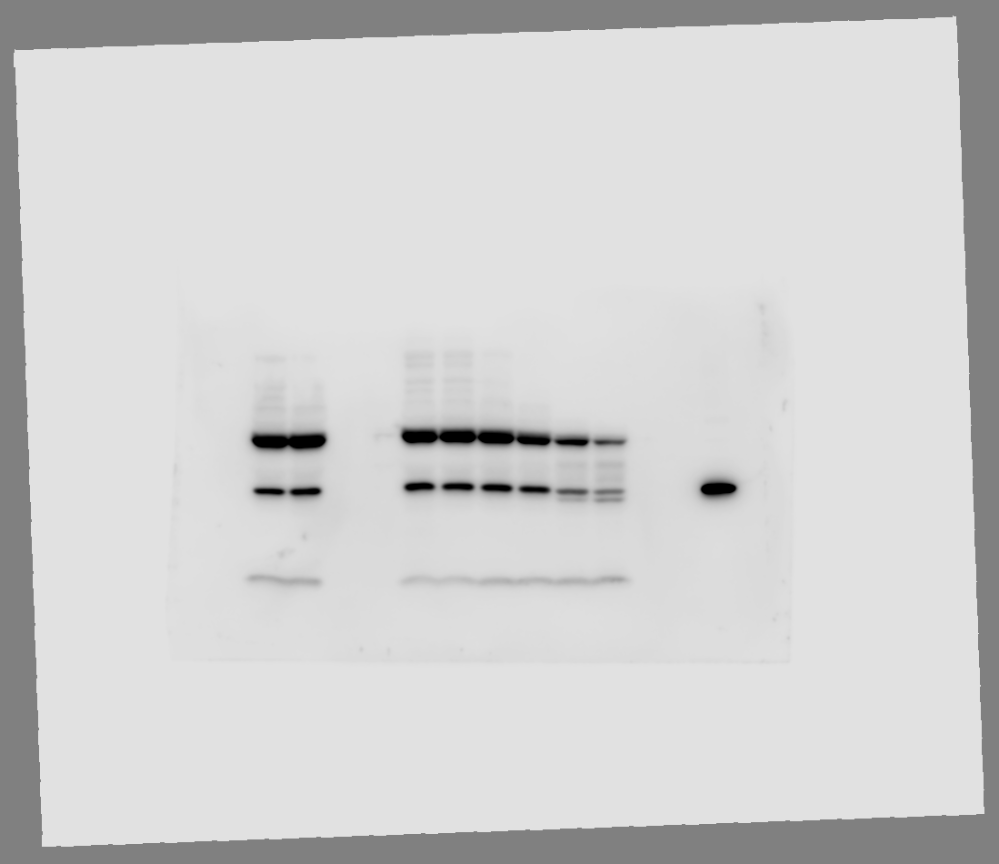

Supplement: Figure 8—source data 2. [file elife-84330-fig8-data2.zip › Figure 8-source data 1/8D Homozygous clogger mito Ant1.tif]

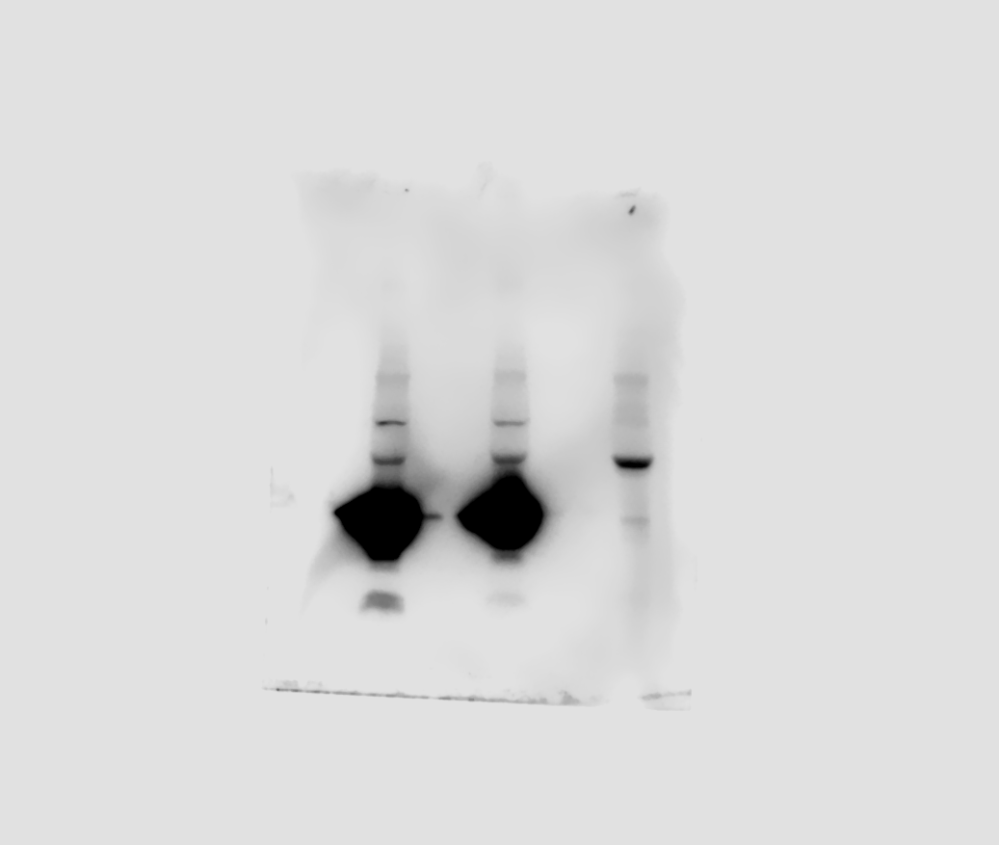

Supplement: Figure 8—source data 2. [file elife-84330-fig8-data2.zip › Figure 8-source data 1/8B Ant1 long.tif]

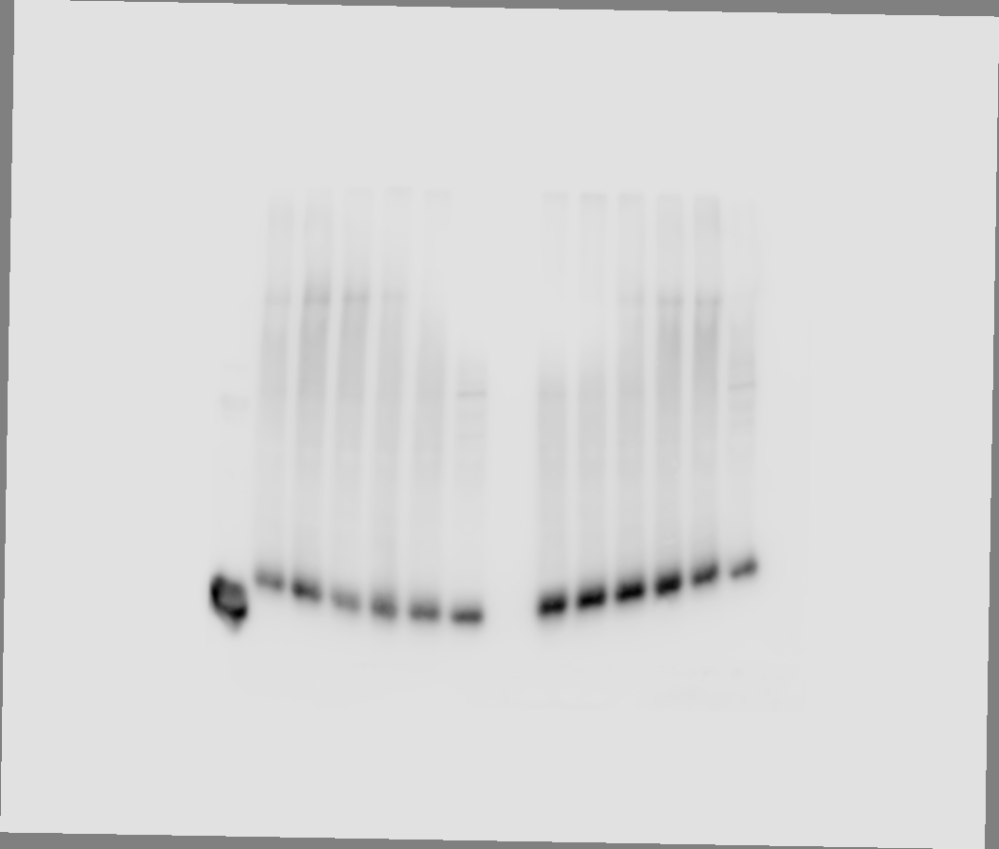

Supplement: Figure 8—figure supplement 1—source data 2. [file elife-84330-fig8-figsupp1-data2.zip › Figure 8-figure supplement 1-source data 1/Fig 8-fs1I Tim22.tif]

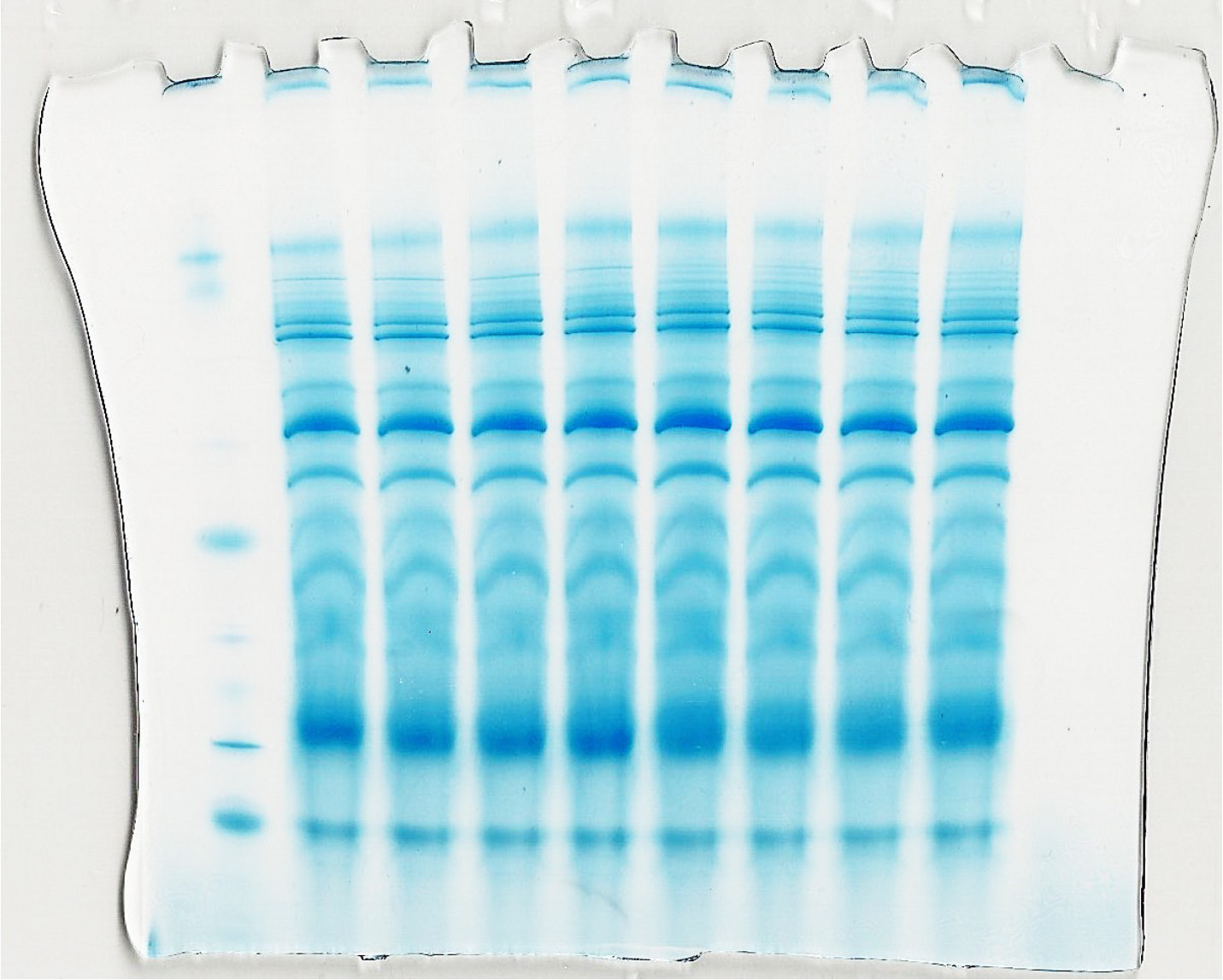

Supplement: Figure 8—figure supplement 1—source data 2. [file elife-84330-fig8-figsupp1-data2.zip › Figure 8-figure supplement 1-source data 1/Fig 8-fs1C BN-PAGE.jpg]

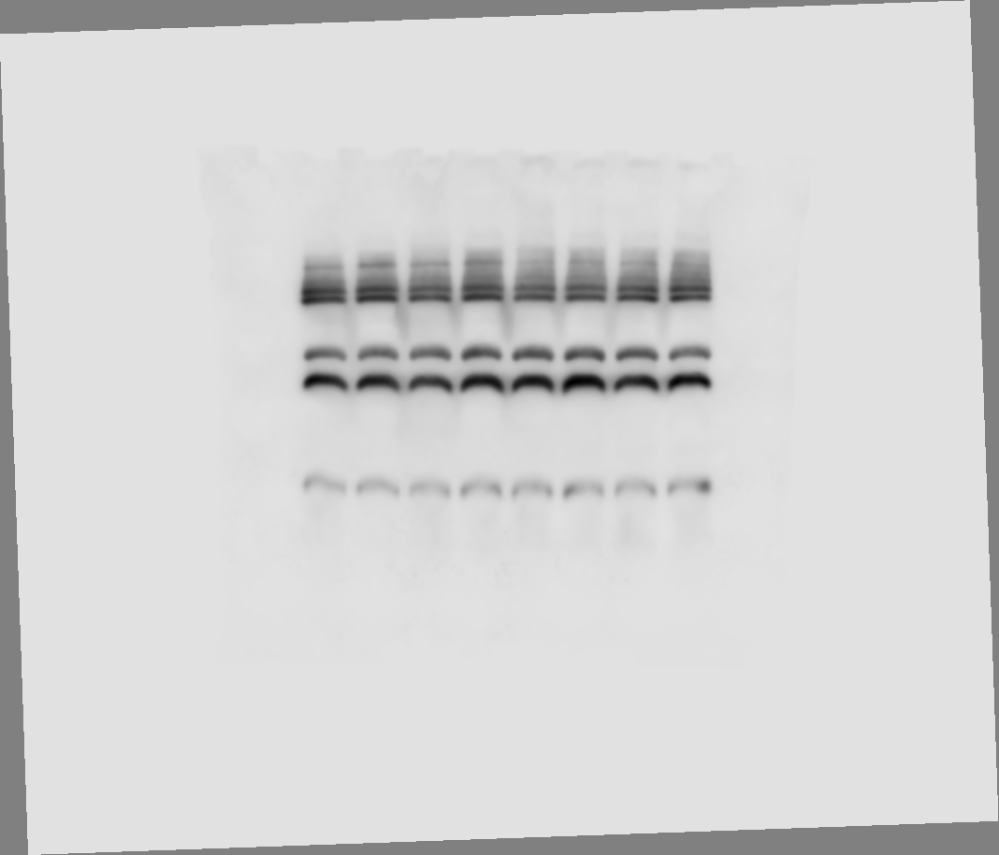

Supplement: Figure 8—figure supplement 1—source data 2. [file elife-84330-fig8-figsupp1-data2.zip › Figure 8-figure supplement 1-source data 1/Fig 8-fs1D BN-PAGE_OXPHOS cocktail.png]

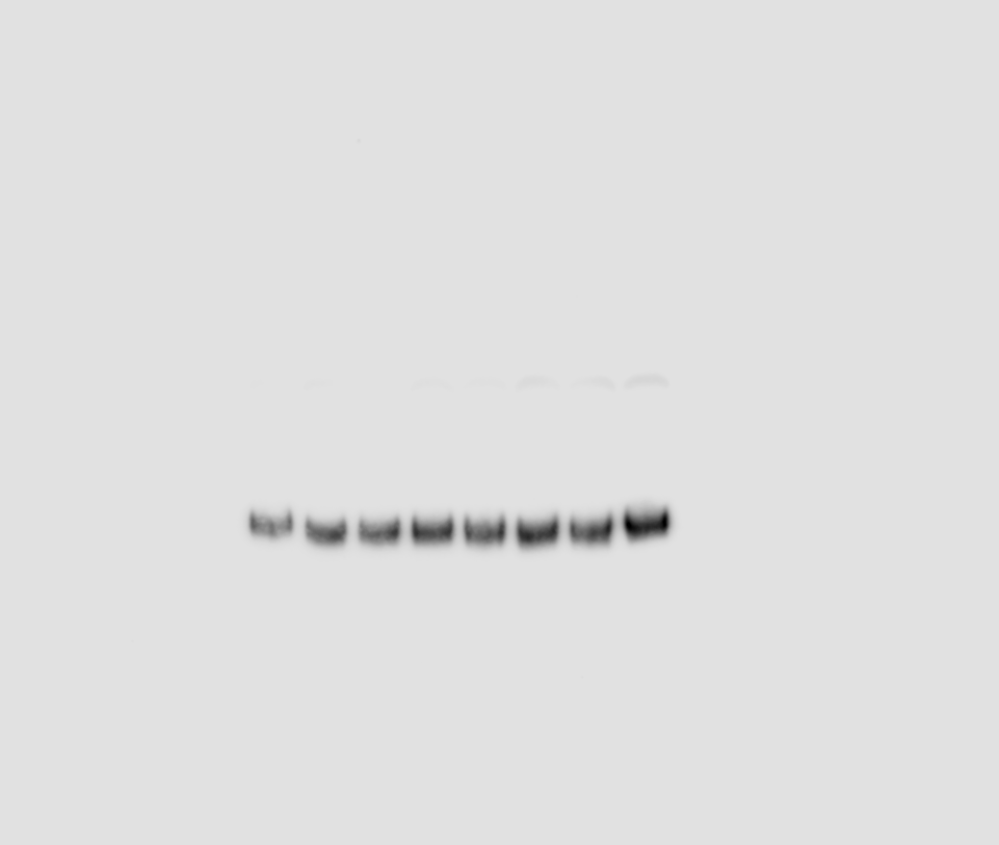

Supplement: Figure 8—figure supplement 1—source data 2. [file elife-84330-fig8-figsupp1-data2.zip › Figure 8-figure supplement 1-source data 1/Fig 8-fs1E BN-PAGE_Tim23.png]

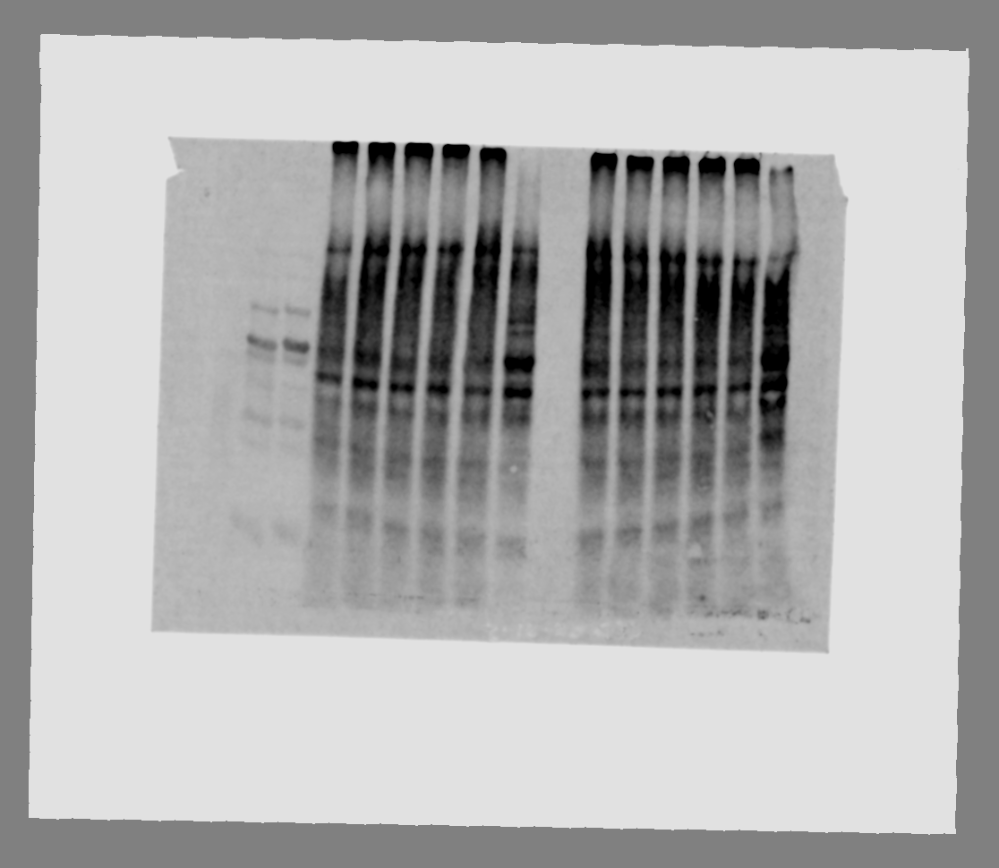

Supplement: Figure 8—figure supplement 1—source data 2. [file elife-84330-fig8-figsupp1-data2.zip › Figure 8-figure supplement 1-source data 1/Fig 8-fs1I TPS.tif]

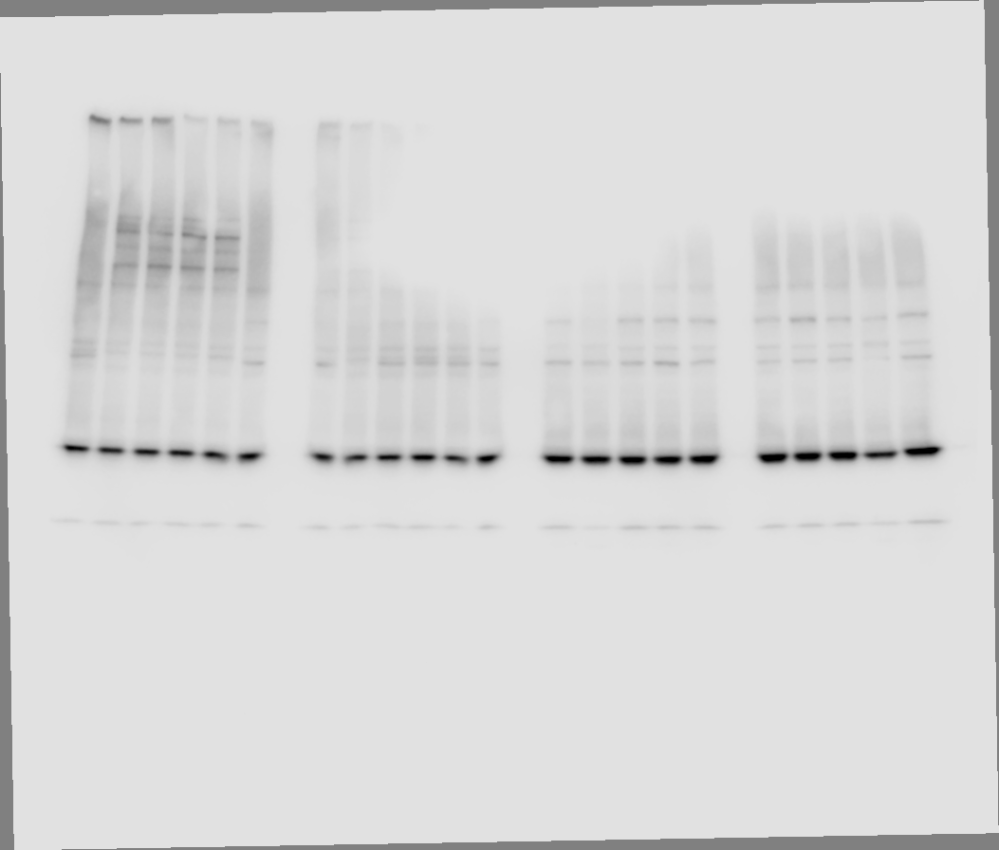

Supplement: Figure 8—figure supplement 2—source data 2. [file elife-84330-fig8-figsupp2-data2.zip › Figure 8-figure supplement 2-source data 1/Fig 8-fs2A eIF2a.png]

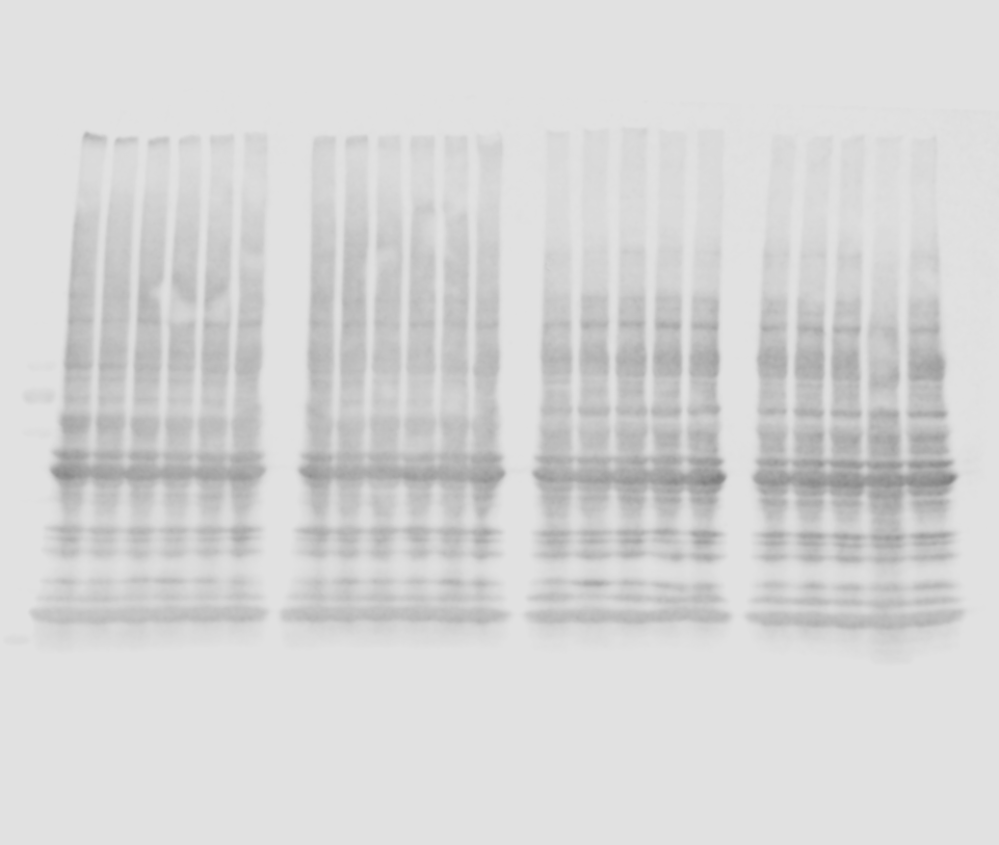

Supplement: Figure 8—figure supplement 2—source data 2. [file elife-84330-fig8-figsupp2-data2.zip › Figure 8-figure supplement 2-source data 1/Fig 8-fs2A TPS top.png]

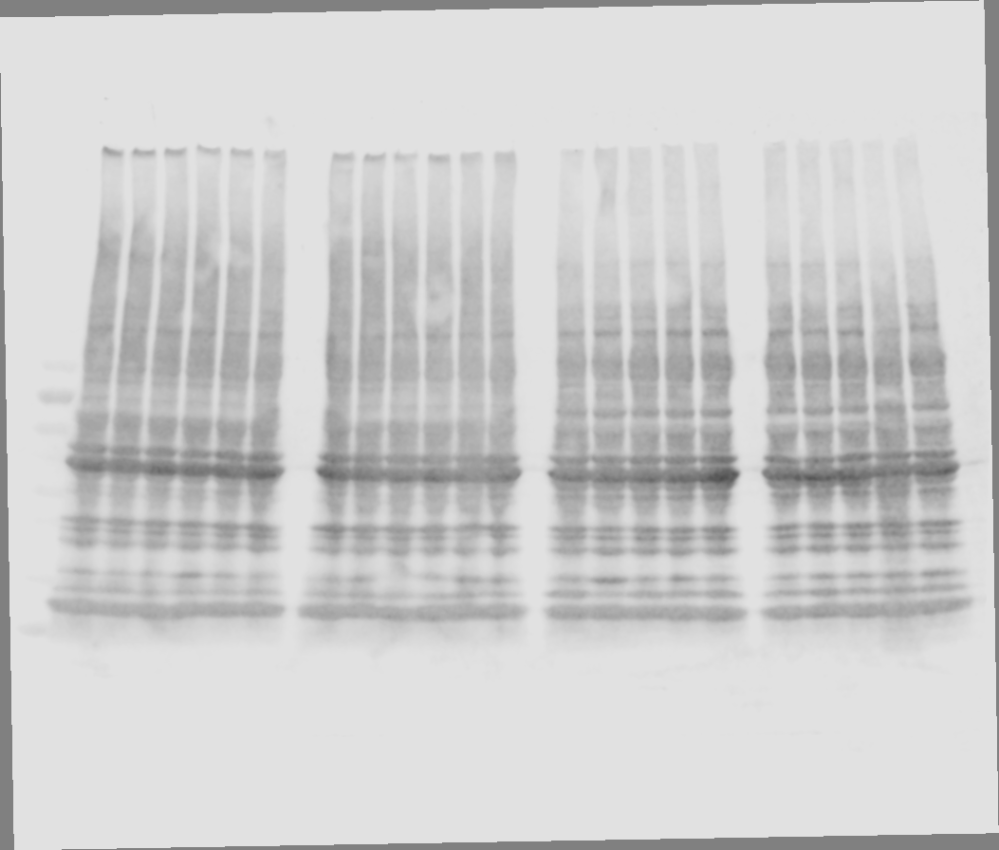

Supplement: Figure 8—figure supplement 2—source data 2. [file elife-84330-fig8-figsupp2-data2.zip › Figure 8-figure supplement 2-source data 1/Fig 8-fs2A TPS bottom.png]

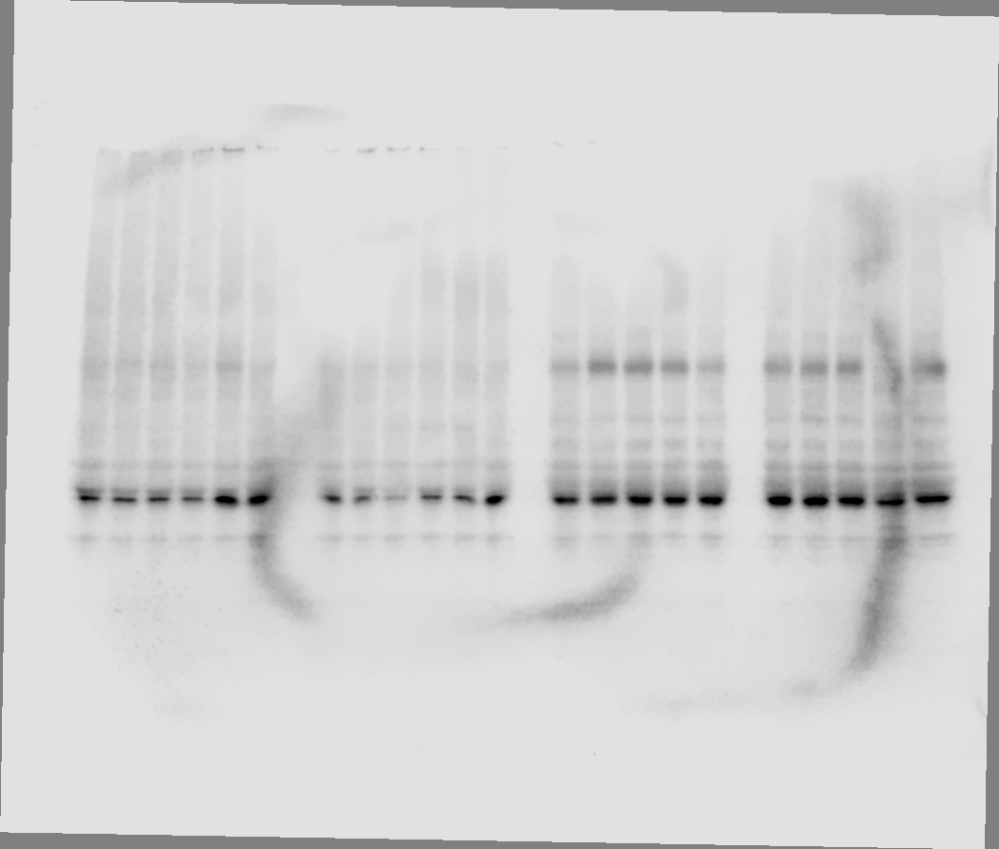

Supplement: Figure 8—figure supplement 2—source data 2. [file elife-84330-fig8-figsupp2-data2.zip › Figure 8-figure supplement 2-source data 1/Fig 8-fs2A phospho-eIF2a.png]
