# Supplementary material for: Mitochondrial protein import clogging as a mechanism of disease
Source: eLife. 2023 May 2;12:e84330. doi: 10.7554/eLife.84330 (PMC10208645; doi:10.7554/eLife.84330)
Supplement: Figure 1—figure supplement 1—source data 1. [file elife-84330-fig1-figsupp1-data1.zip › Figure 1-figure supplement 1/Figure 1-figure supplement 1-source data annotated.pdf]

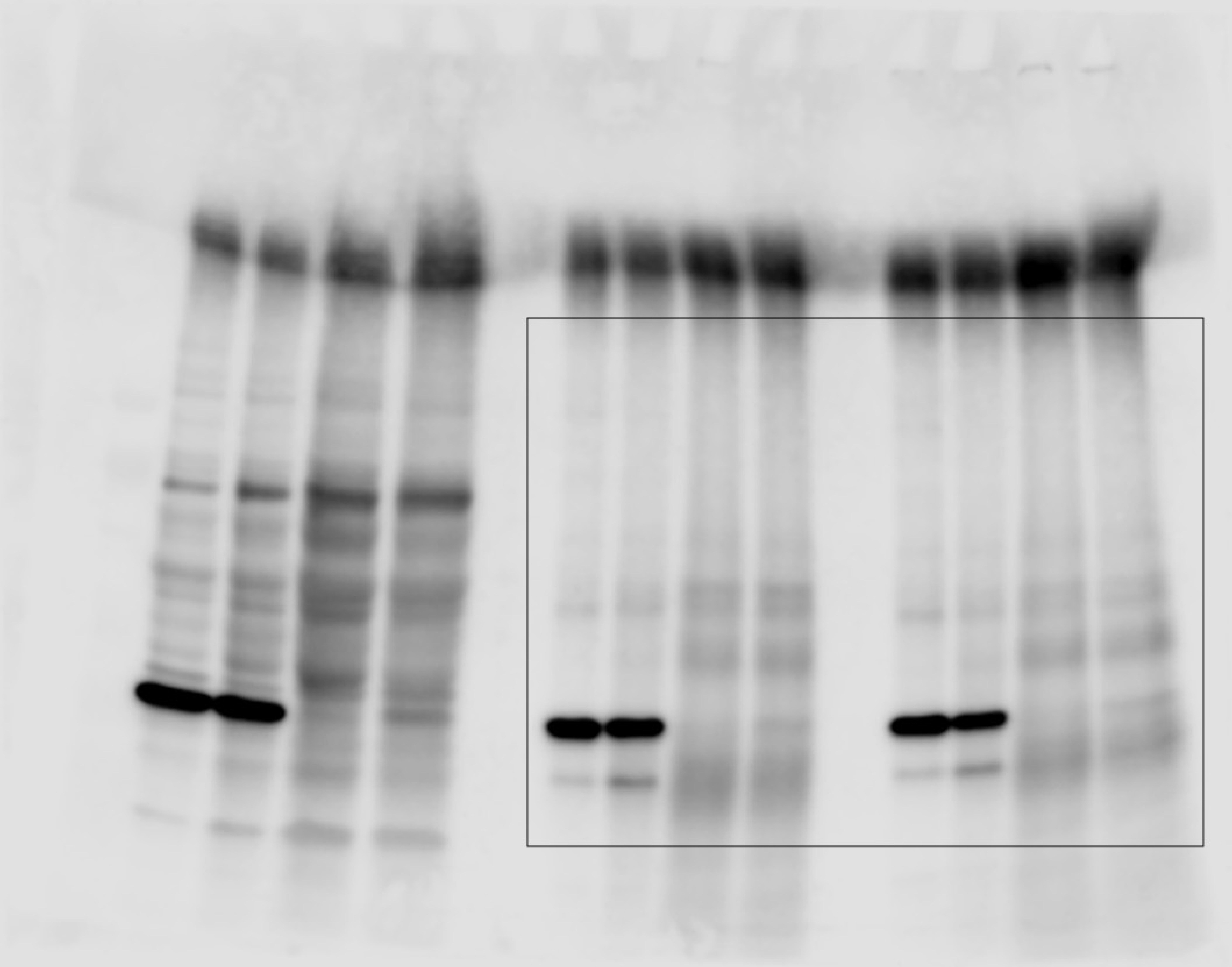

Cropped area for Figure S1B
AAC2

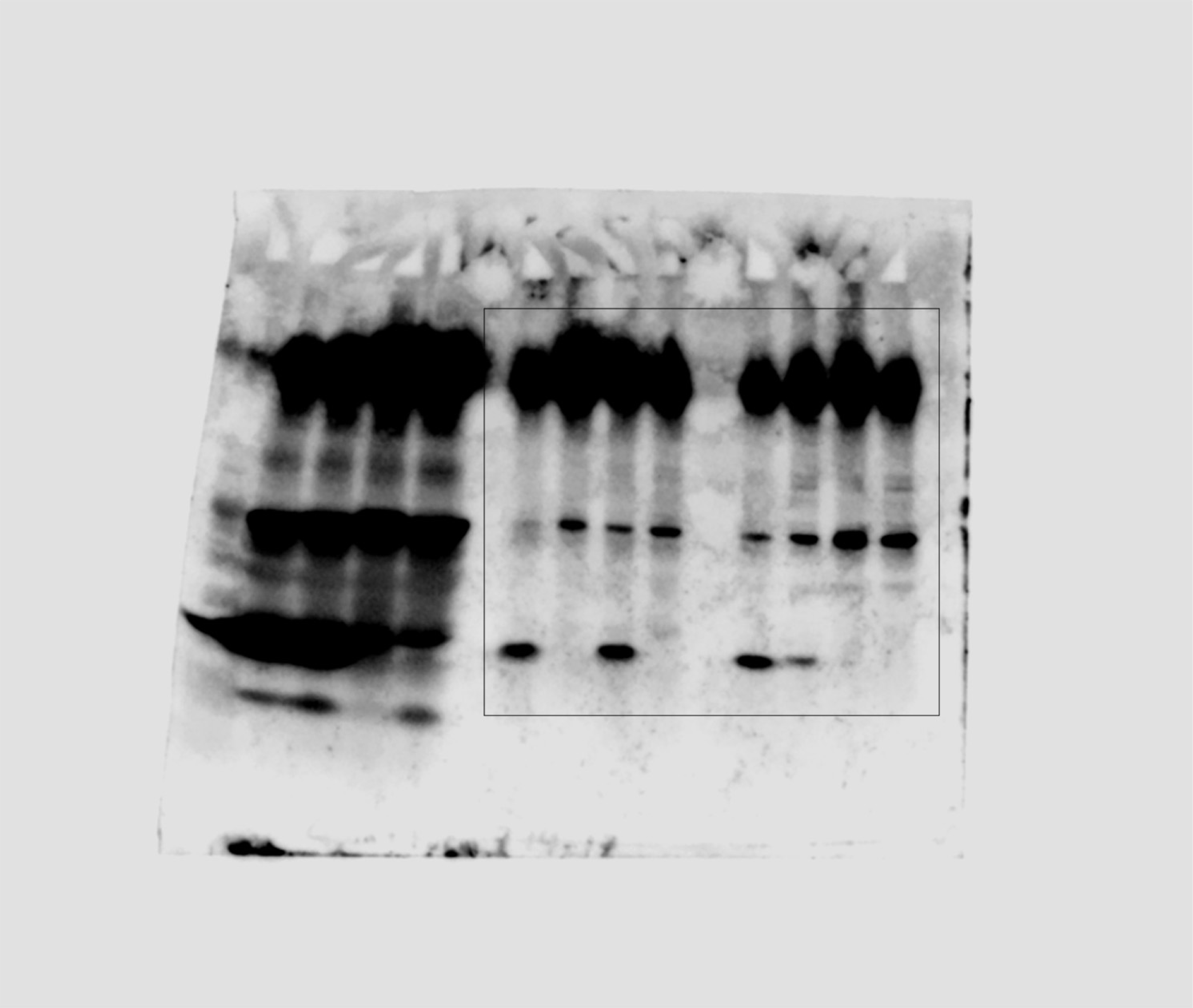

Cropped area for Figure S1C, left
AAC2

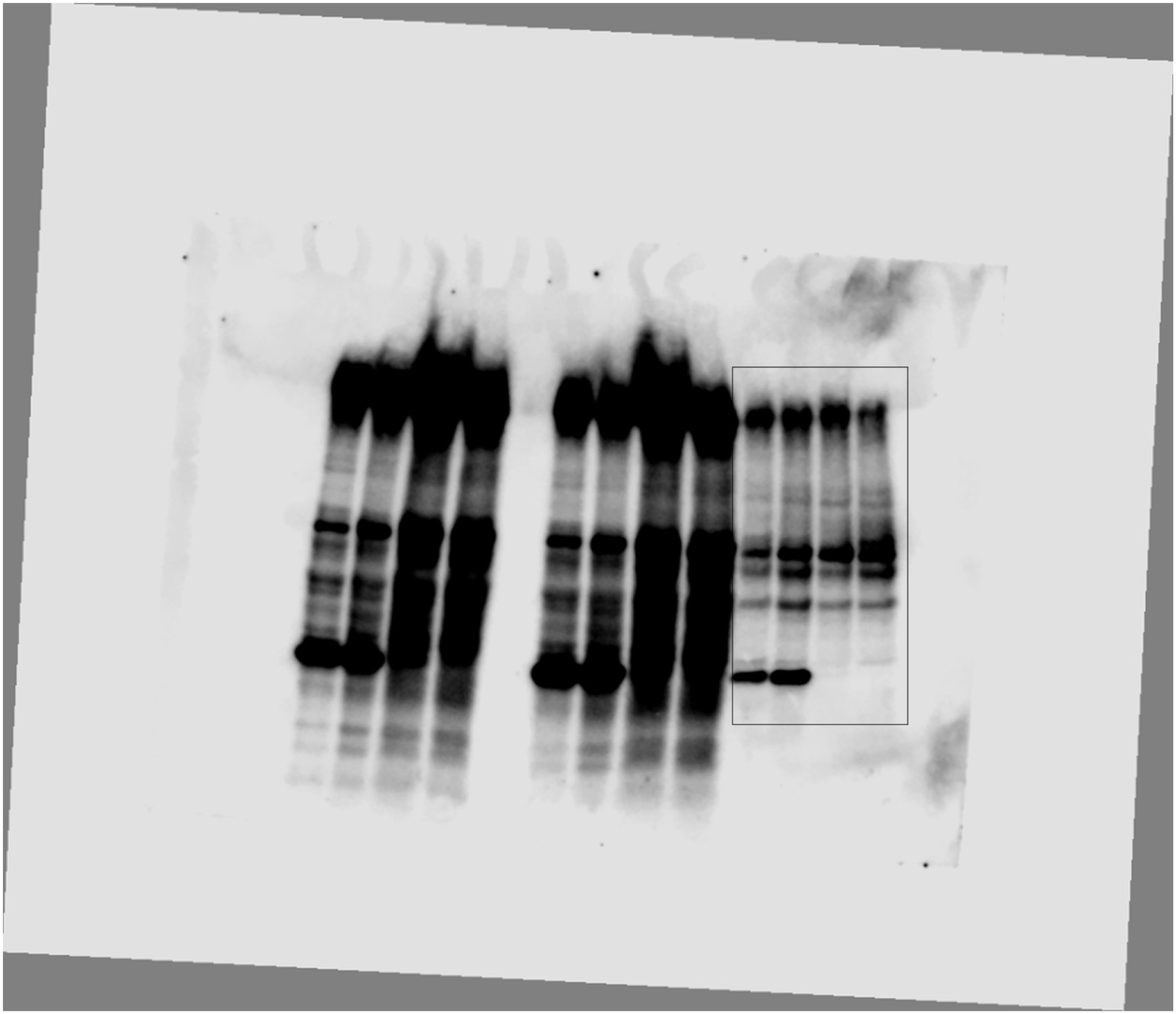

Cropped area for Figure S1C right
AAC2
