# Supplementary material for: Mitochondrial protein import clogging as a mechanism of disease
Source: eLife. 2023 May 2;12:e84330. doi: 10.7554/eLife.84330 (PMC10208645; doi:10.7554/eLife.84330)
Supplement: Figure 2—source data 1. [file elife-84330-fig2-data1.zip › Figure 2-source data 1/Figure 2-source data annotated.pdf]

Fig.2A

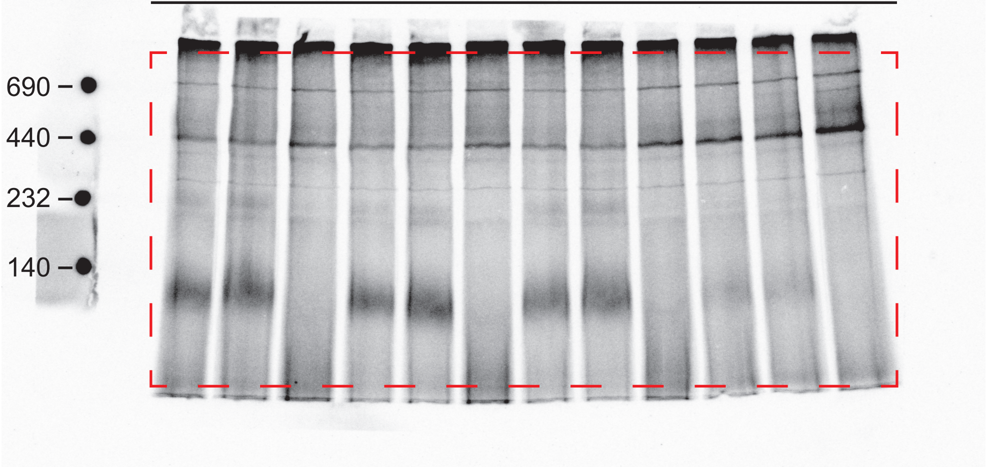

Fig.2C

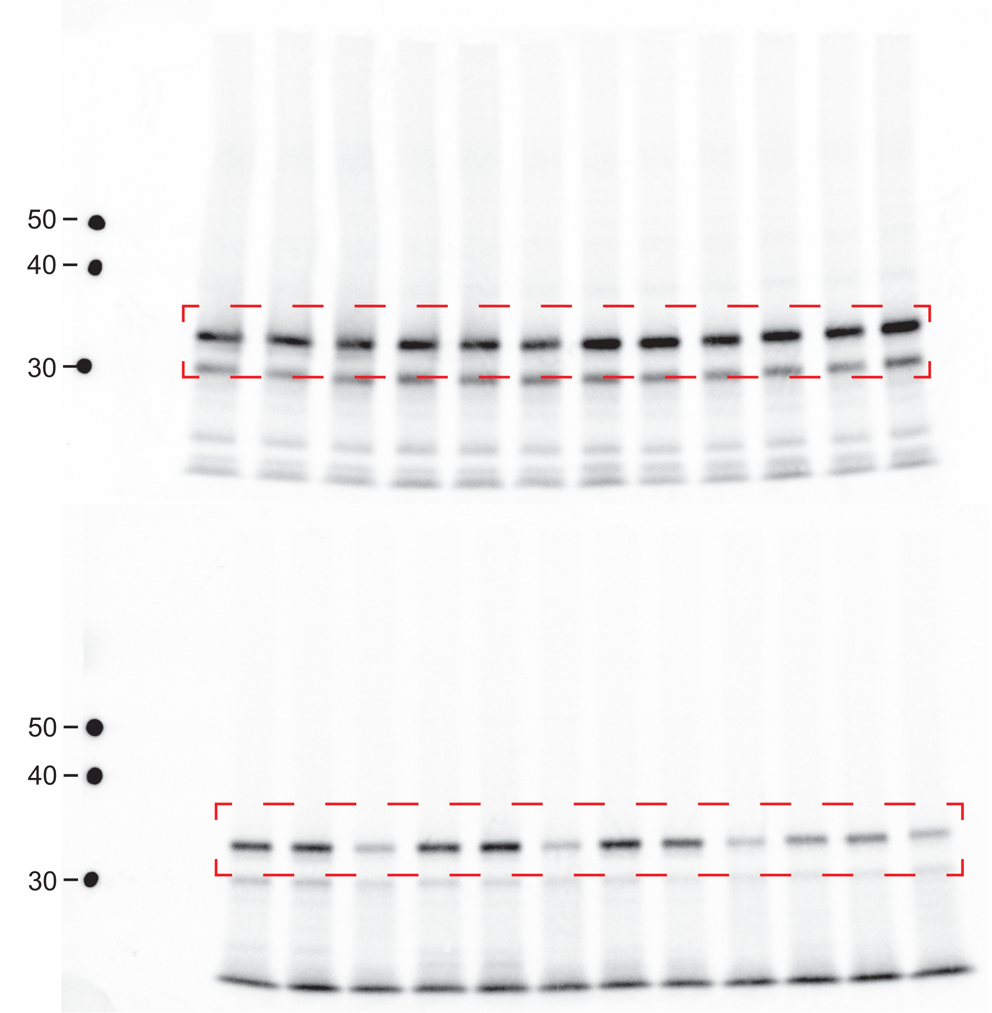

Fig.2E

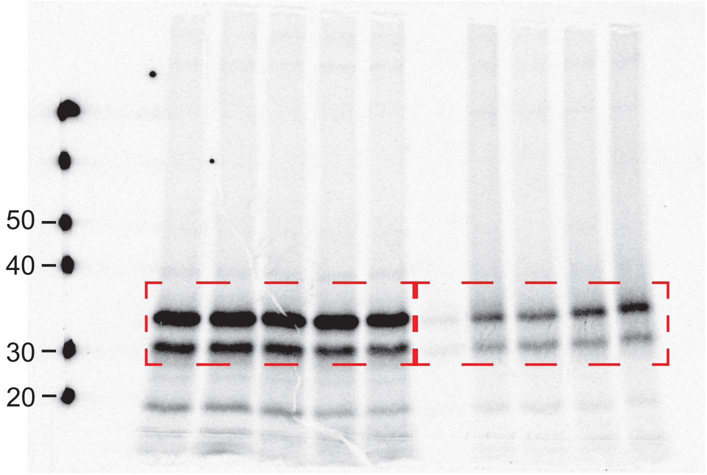

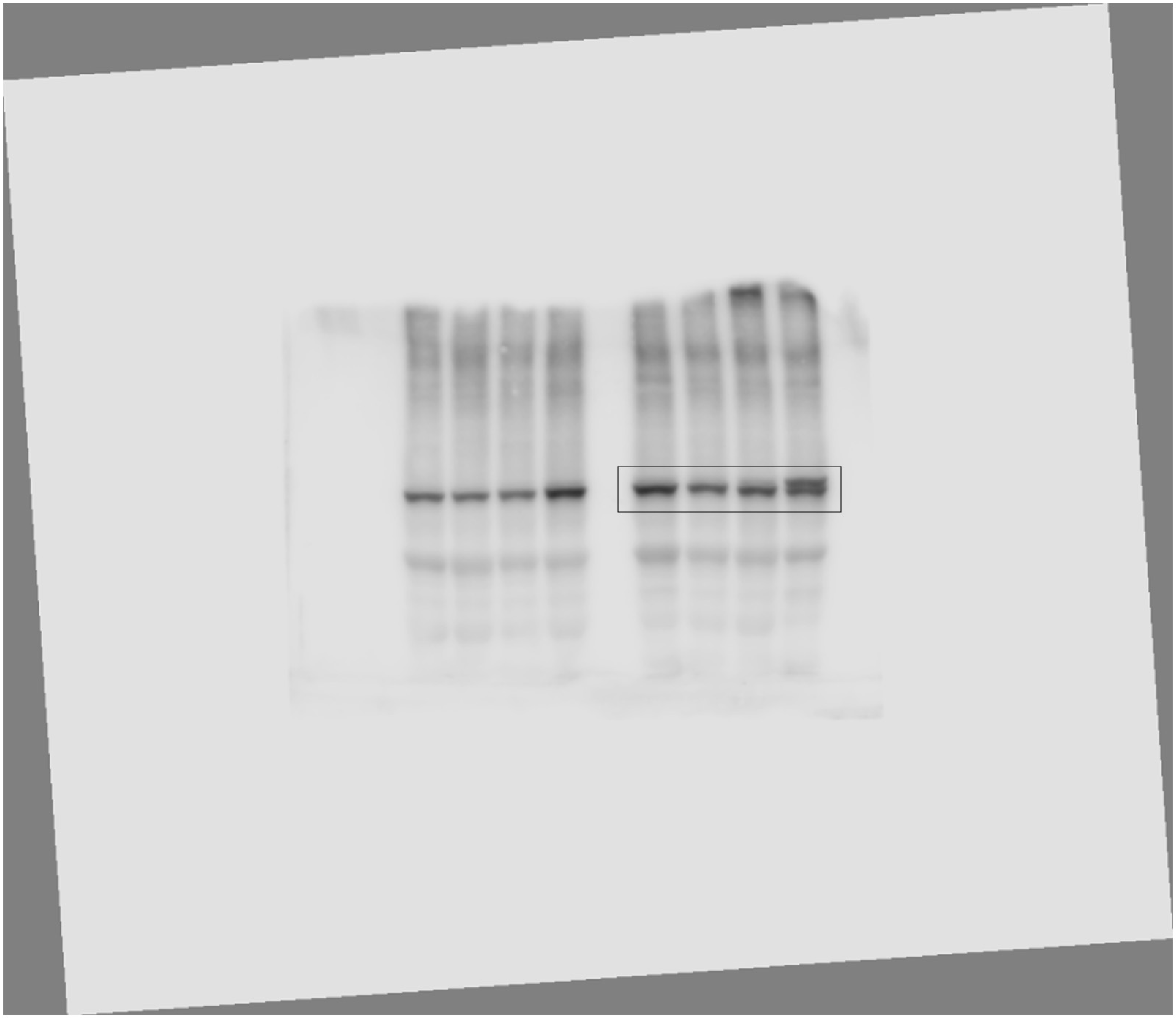

Cropped area for Figure 2G
Hsp60

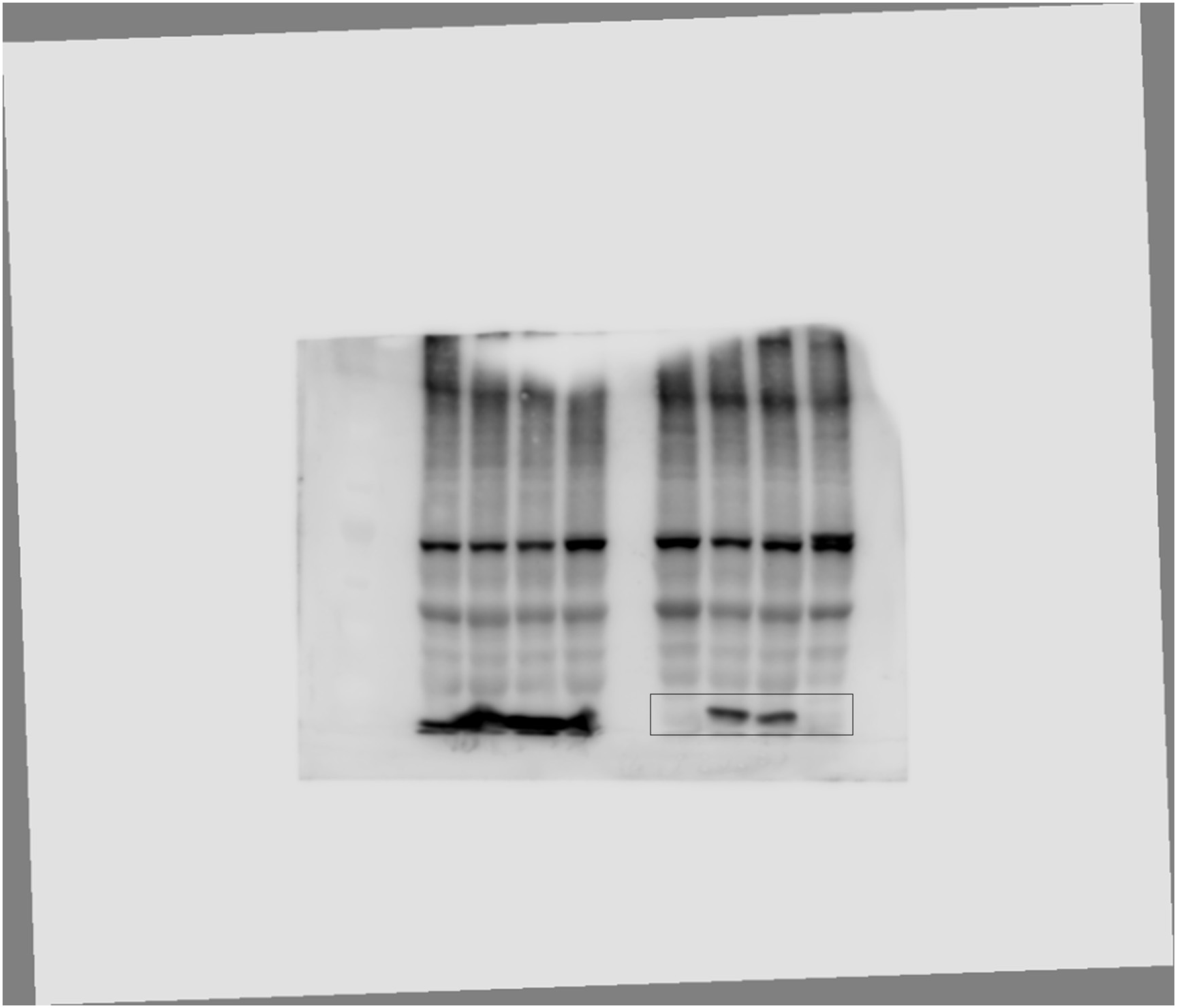

Cropped area for Figure 2G
Aac2

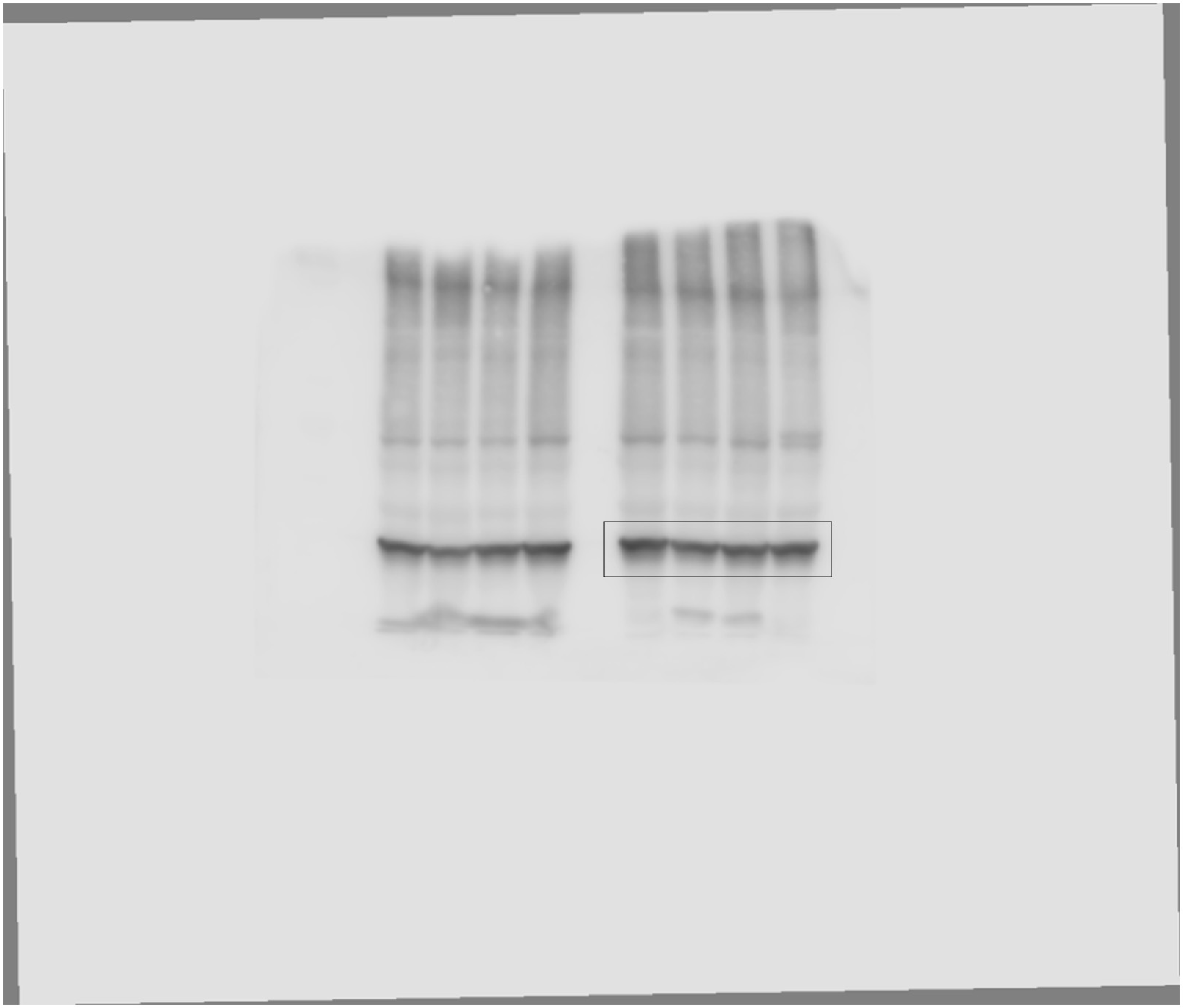

Cropped area for Figure 2G
Ilv5

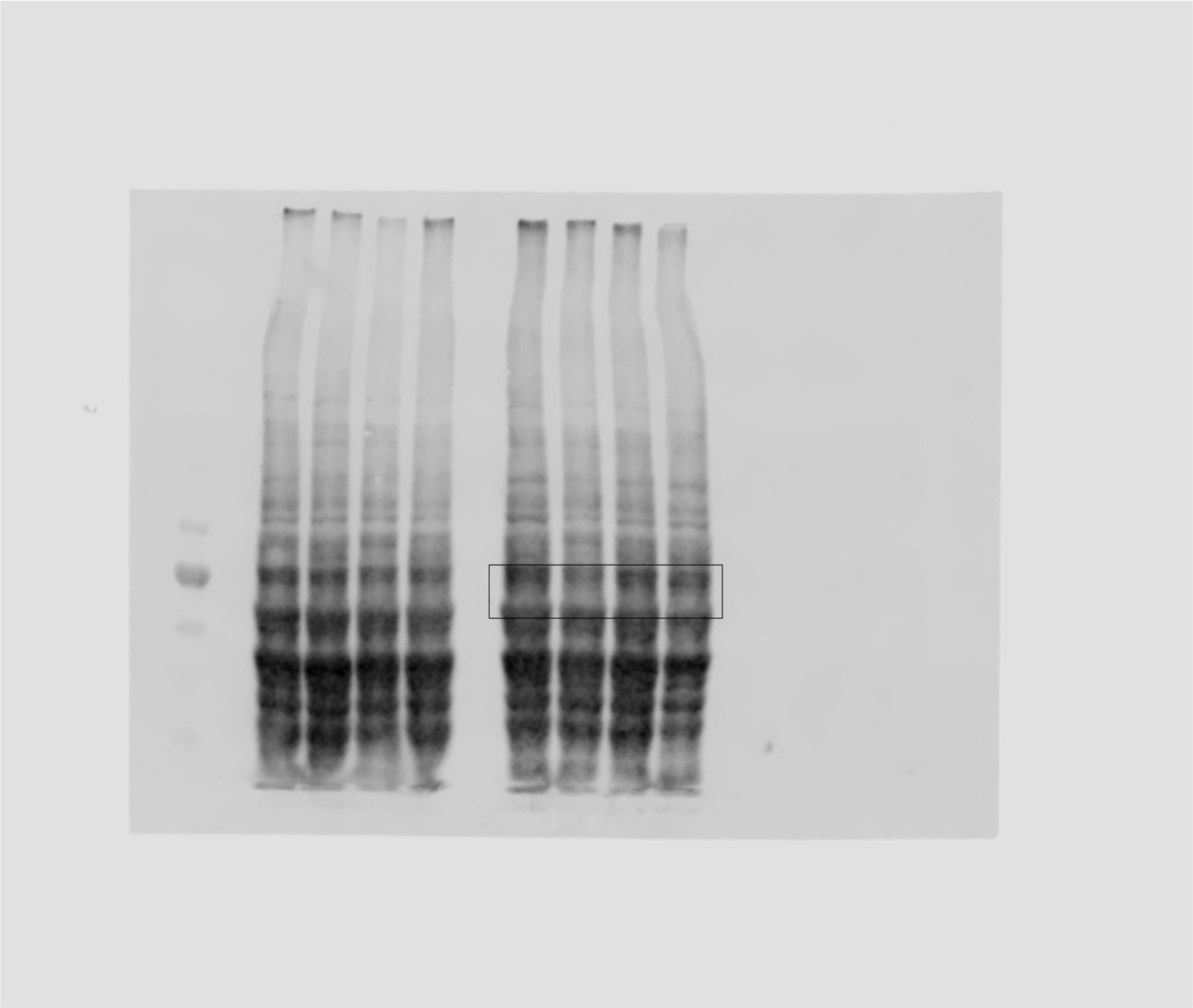

Cropped area for Figure 2G
Total Protein Stain

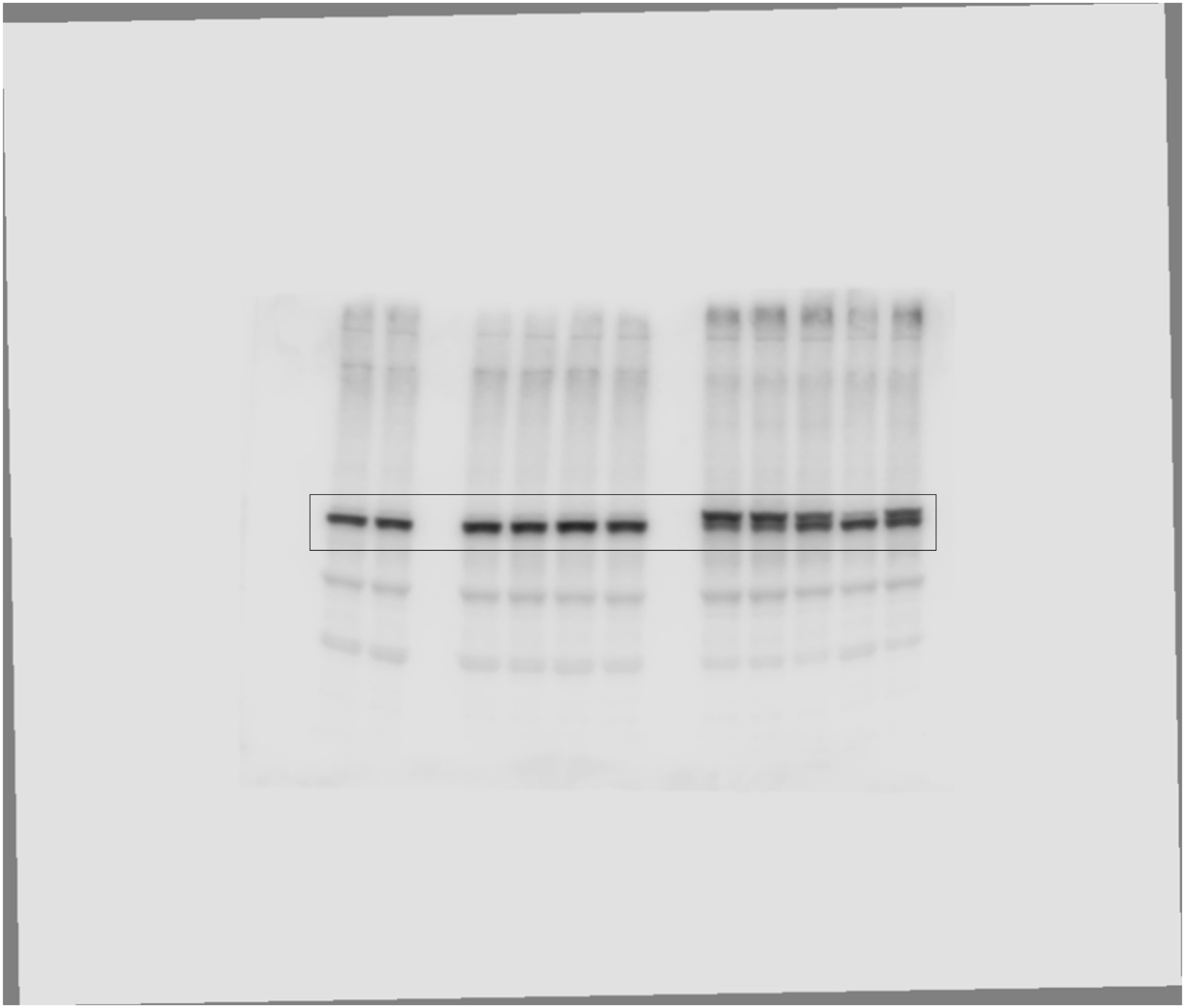

Cropped area for Figure 2H
Hsp60

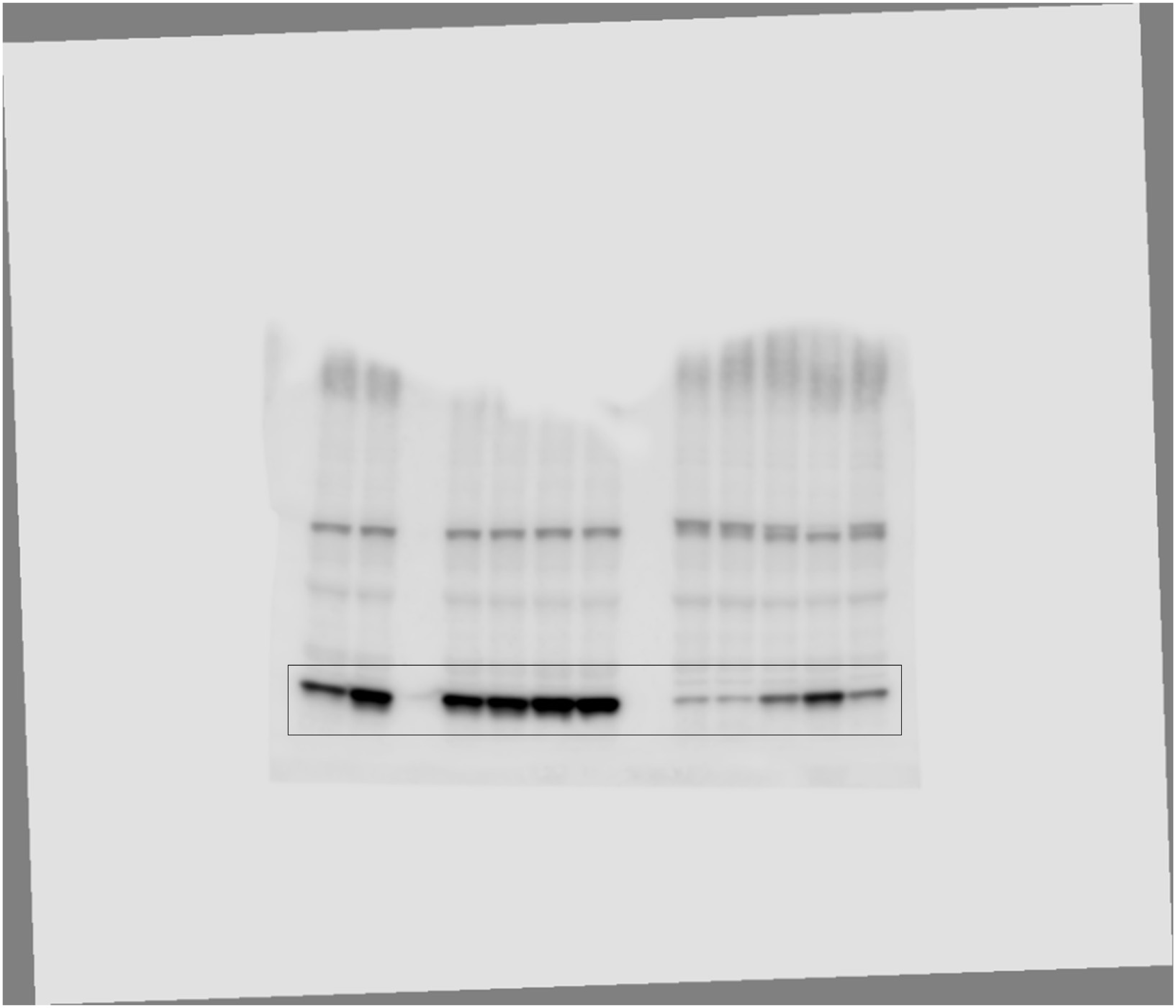

Cropped area for Figure 2H
Aac2

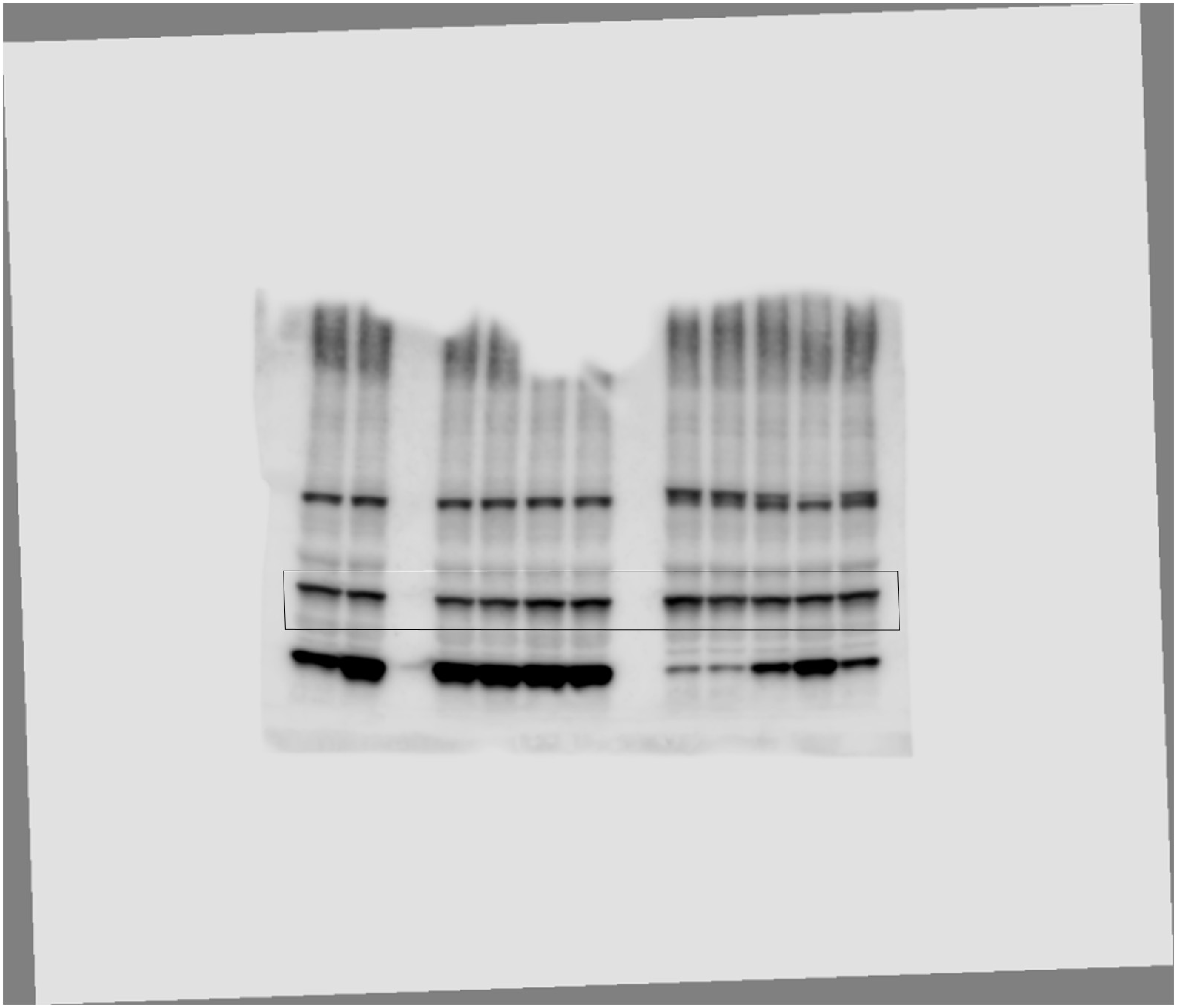

Cropped area for Figure 2H
Ilv5

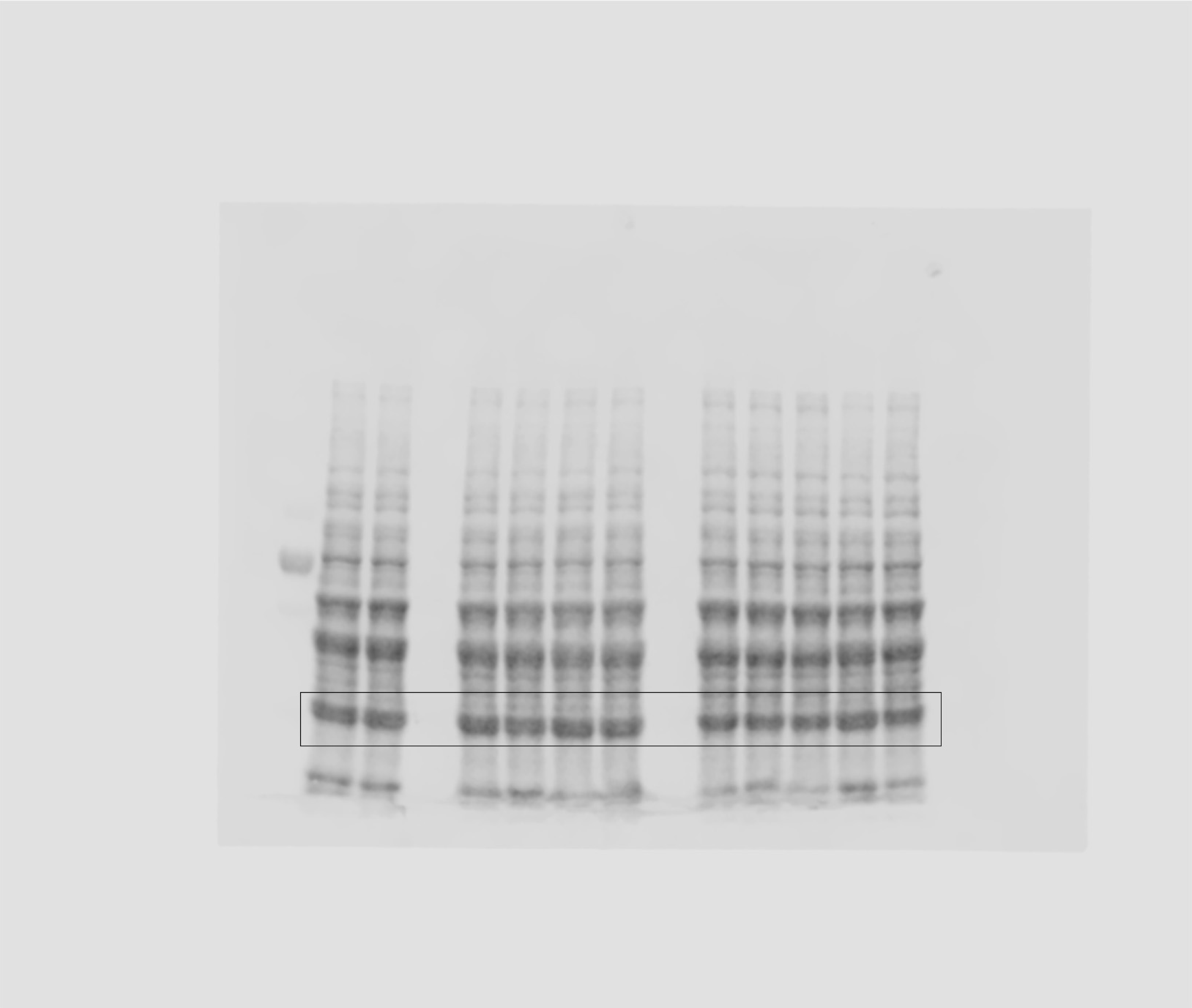

Cropped area for Figure 2H
Total Protein Stain
