# Supplementary material for: Mitochondrial protein import clogging as a mechanism of disease
Source: eLife. 2023 May 2;12:e84330. doi: 10.7554/eLife.84330 (PMC10208645; doi:10.7554/eLife.84330)
Supplement: Figure 2—figure supplement 1—source data 1. [file elife-84330-fig2-figsupp1-data1.zip › Figure 2-figure supplement 1-source data 1/Figure 2-figure supplement 1-source data 1_annotated.pdf]

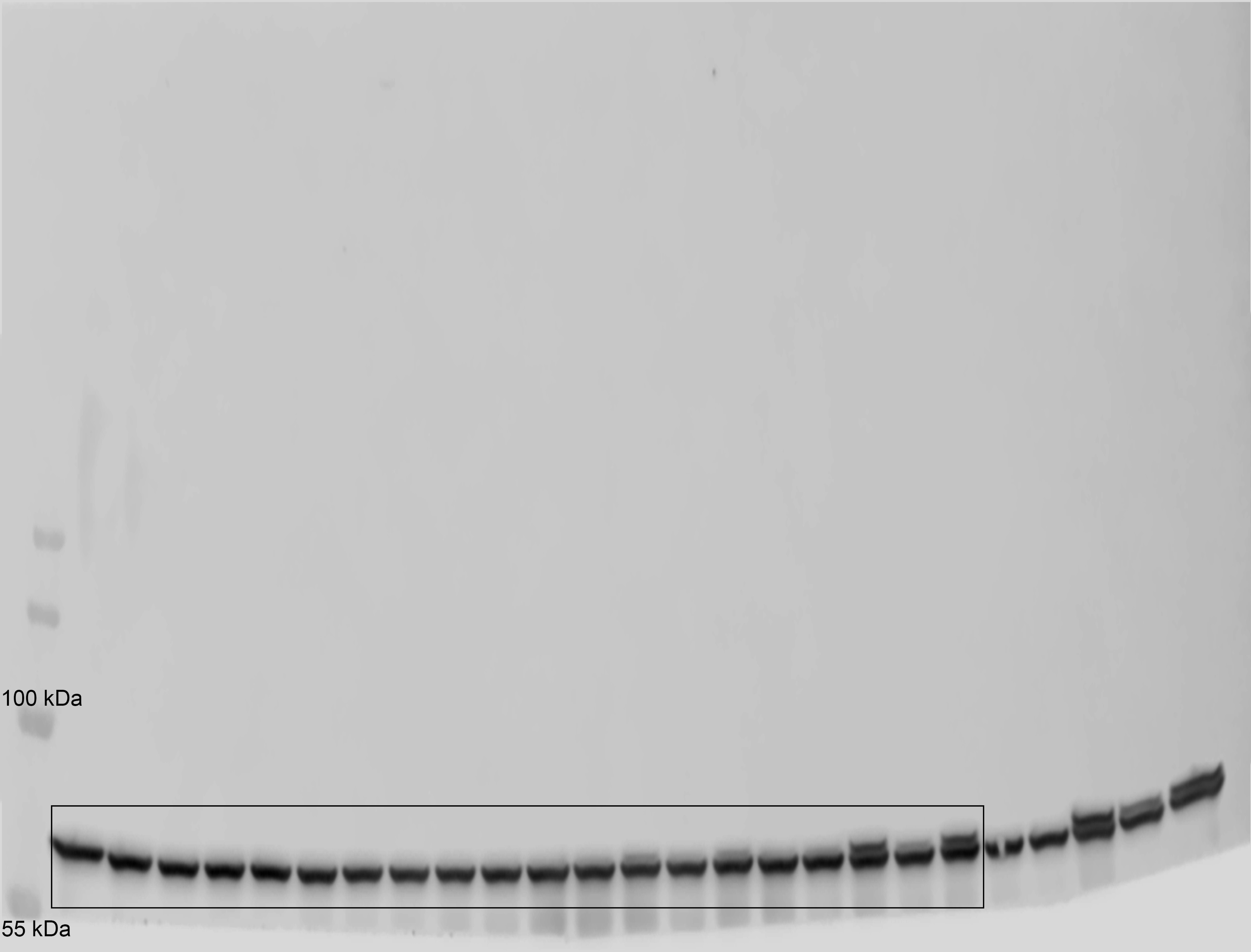

Cropped area for Figure 2-figure supplement 1B_Hsp60

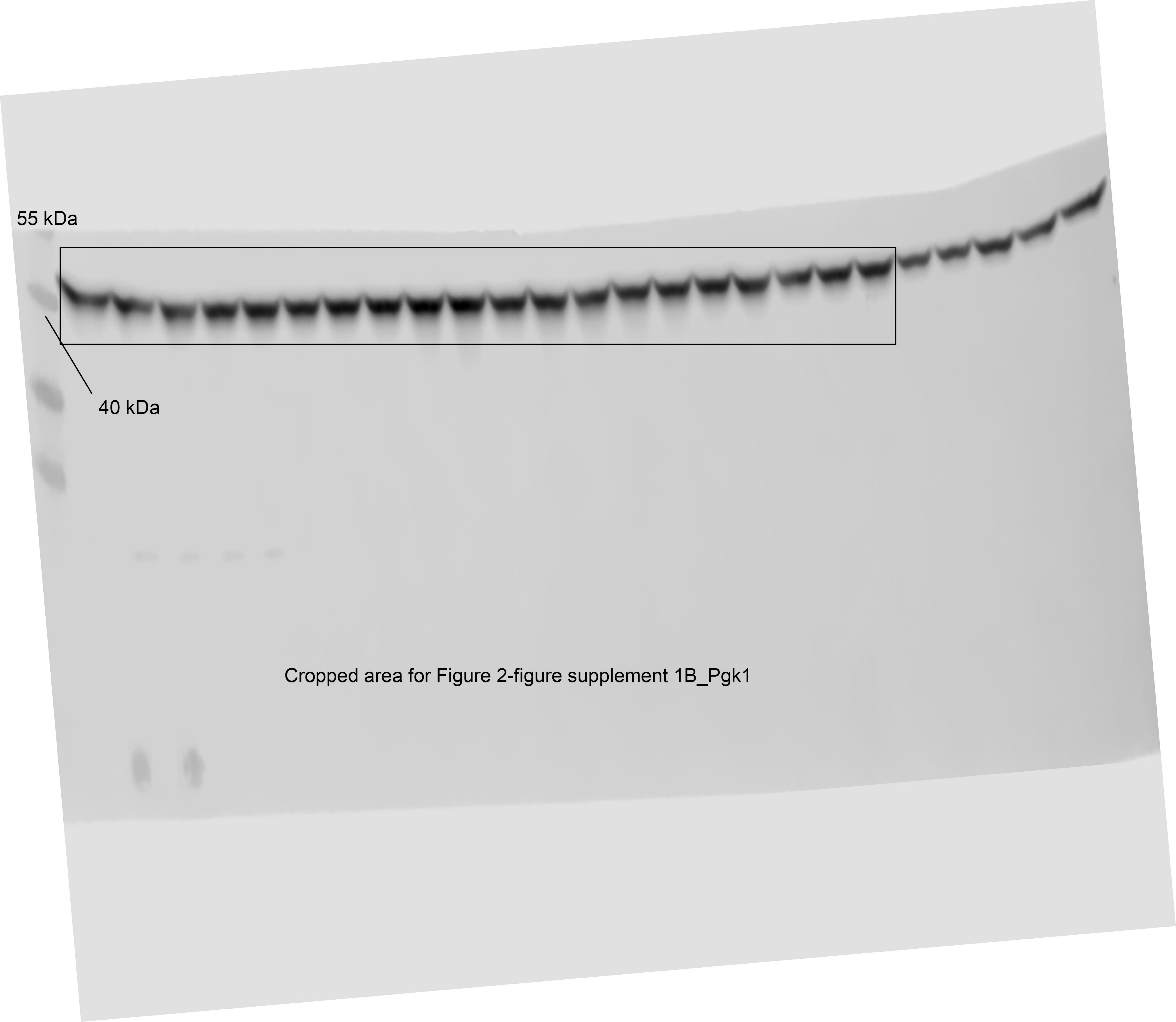
55 kDa
40 kDa
Cropped area for Figure 2-figure supplement 1B_Pgk1

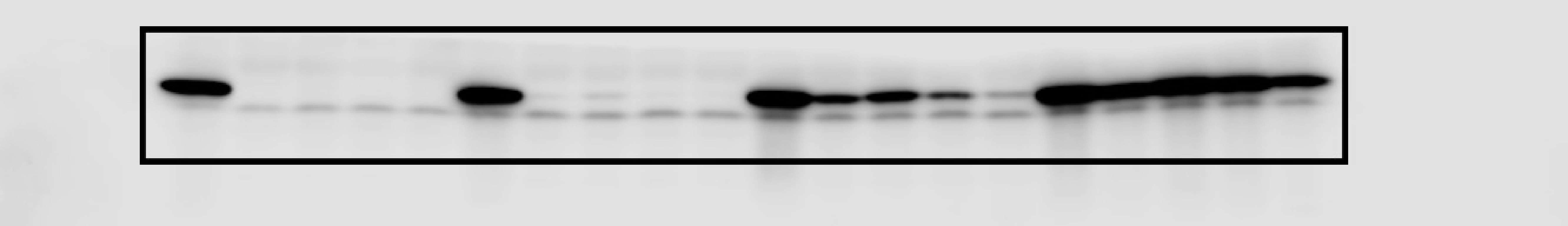

Cropped area for Figure 2-figure supplement 1C_Aac2

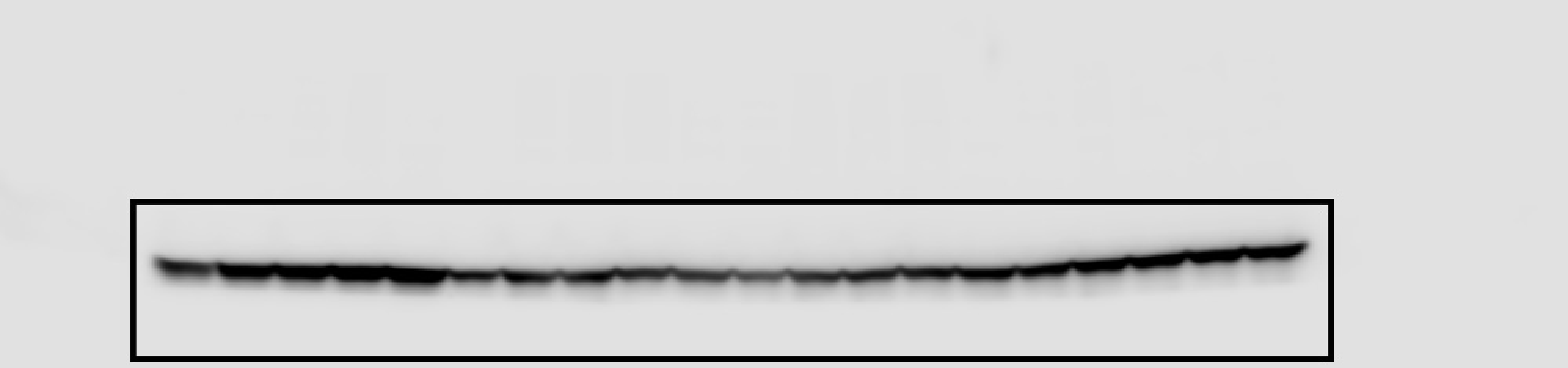

Cropped area for Figure 2-figure supplement 1C_Pgk1
