# Supplementary material for: Mitochondrial protein import clogging as a mechanism of disease
Source: eLife. 2023 May 2;12:e84330. doi: 10.7554/eLife.84330 (PMC10208645; doi:10.7554/eLife.84330)
Supplement: Figure 3—source data 1. [file elife-84330-fig3-data1.zip › Figure 3-source data 1/Figure 3-source data_annotated.pdf]

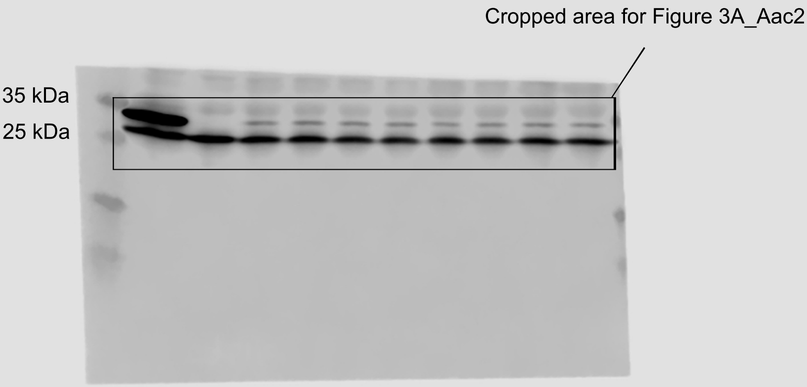
Cropped area for Figure 3A_Aac2
35 kDa
25 kDa

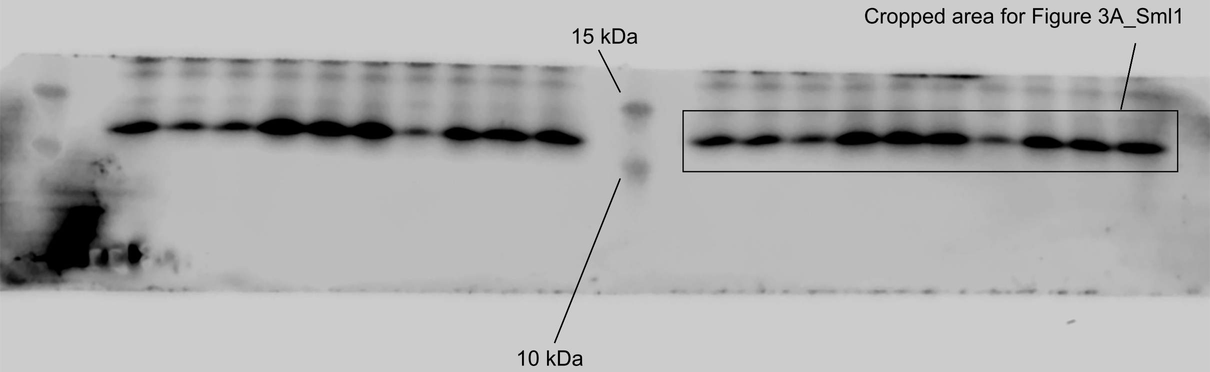
Cropped area for Figure 3A_Sml1
15 kDa
10 kDa

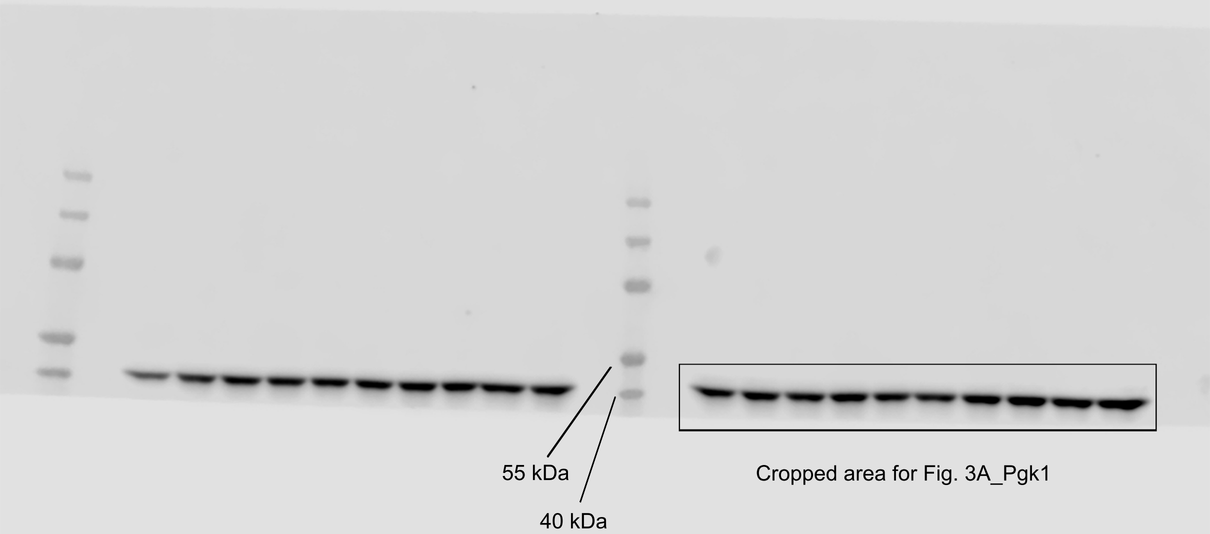

55 kDa
40 kDa
Cropped area for Fig. 3A_Pgk1

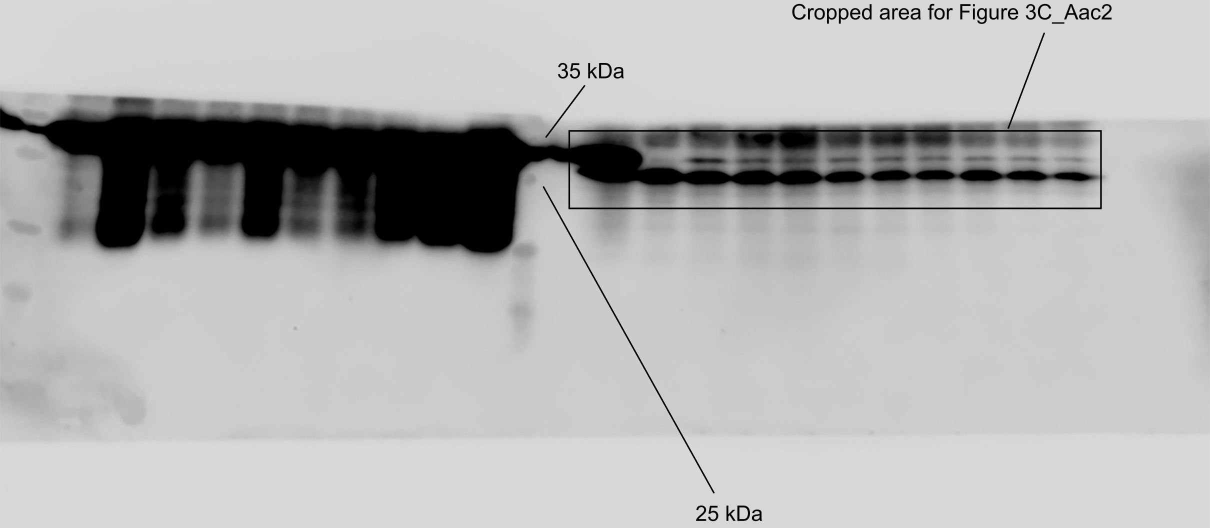
Cropped area for Figure 3C_Aac2
35 kDa
25 kDa

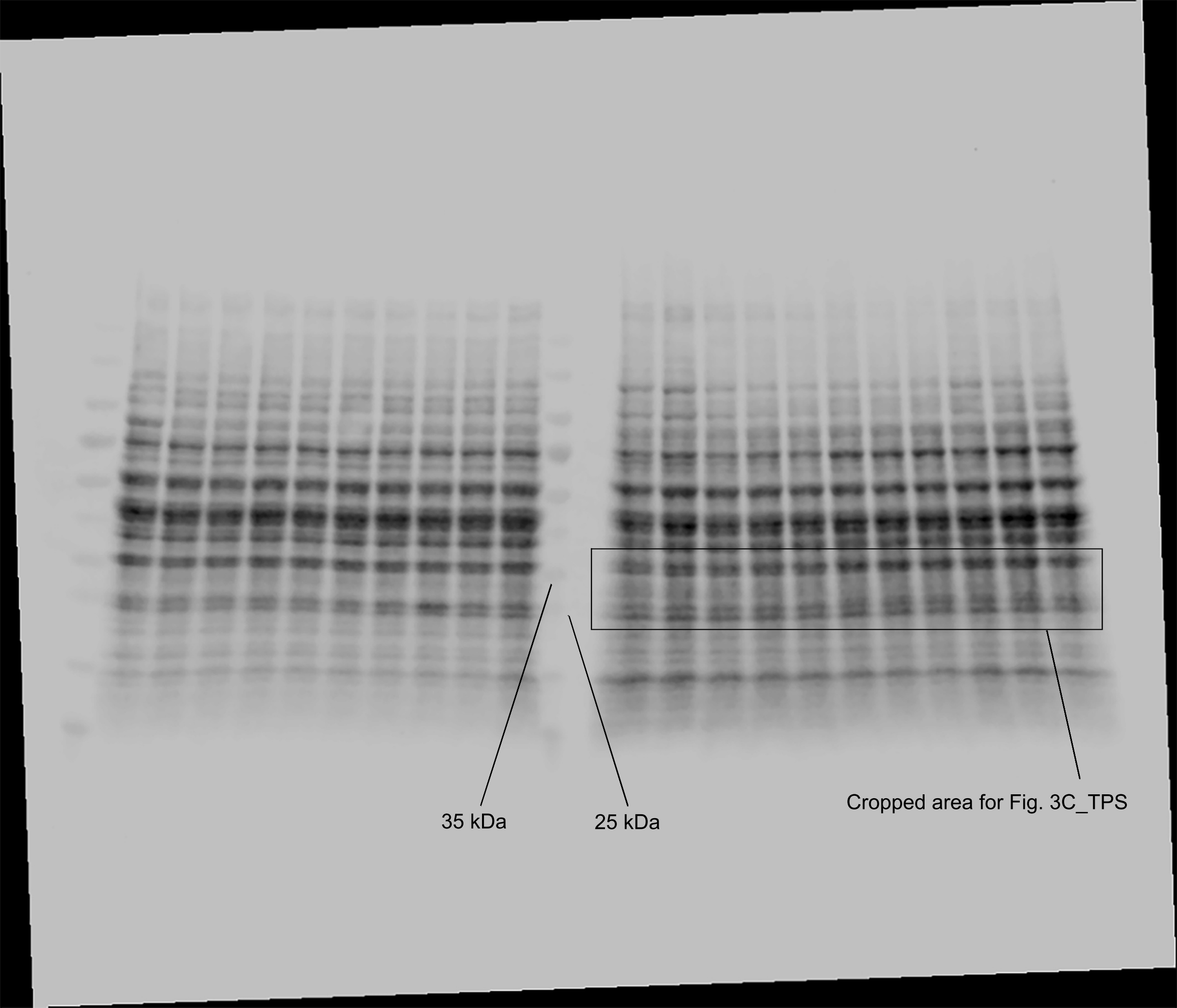
35 kDa
25 kDa
Cropped area for Fig. 3C_TPS

Cropped area for Figure 3D_Aac2

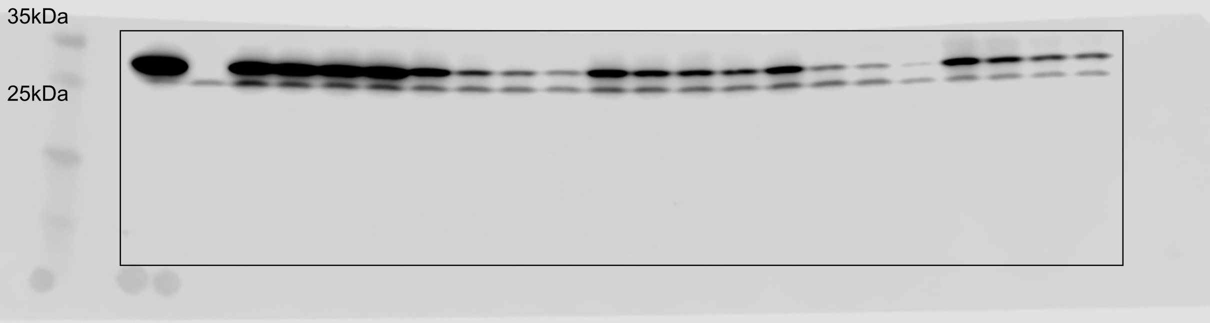

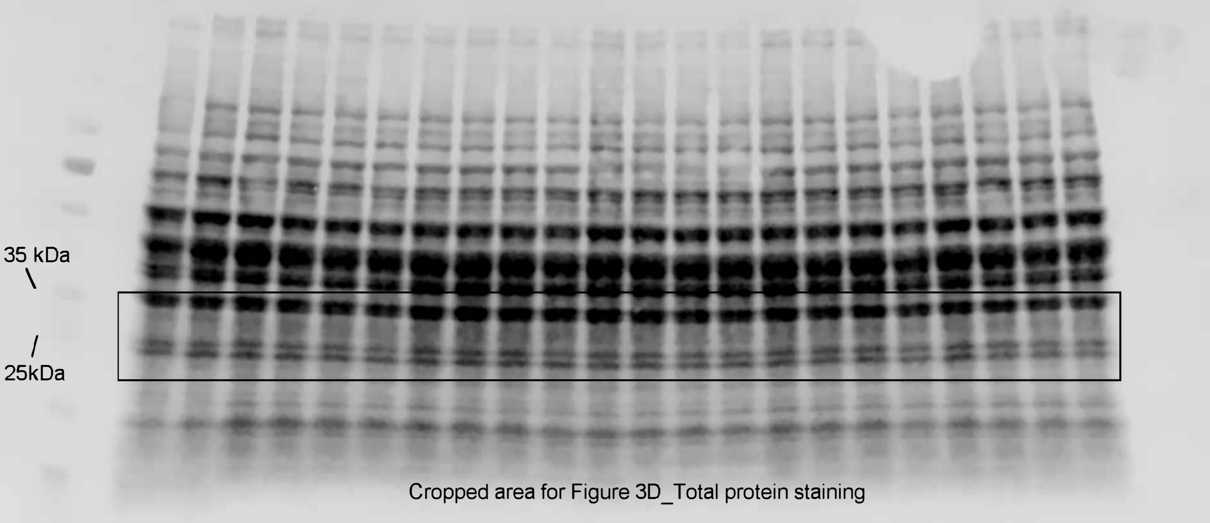

Cropped area for Figure 3D_Total protein staining
