# Supplementary material for: Mitochondrial protein import clogging as a mechanism of disease
Source: eLife. 2023 May 2;12:e84330. doi: 10.7554/eLife.84330 (PMC10208645; doi:10.7554/eLife.84330)
Supplement: Figure 4—figure supplement 1—source data 1. [file elife-84330-fig4-figsupp1-data1.zip › Figure 4-figure supplement 1-source data 1/Figure 4-figure supplement 1_annotated.pdf]

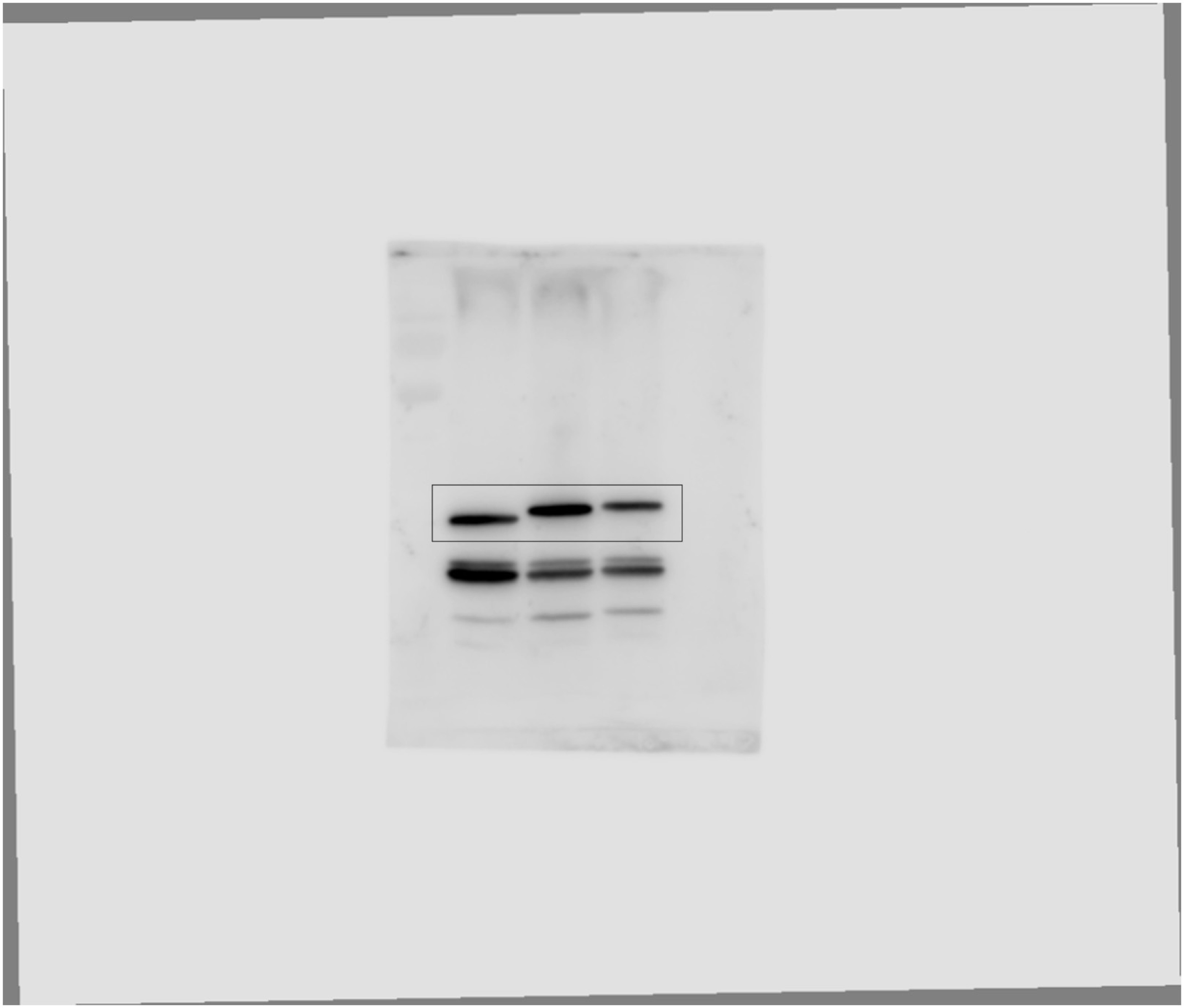

Cropped are for Figure 4-figure supplement 1A
Aac2

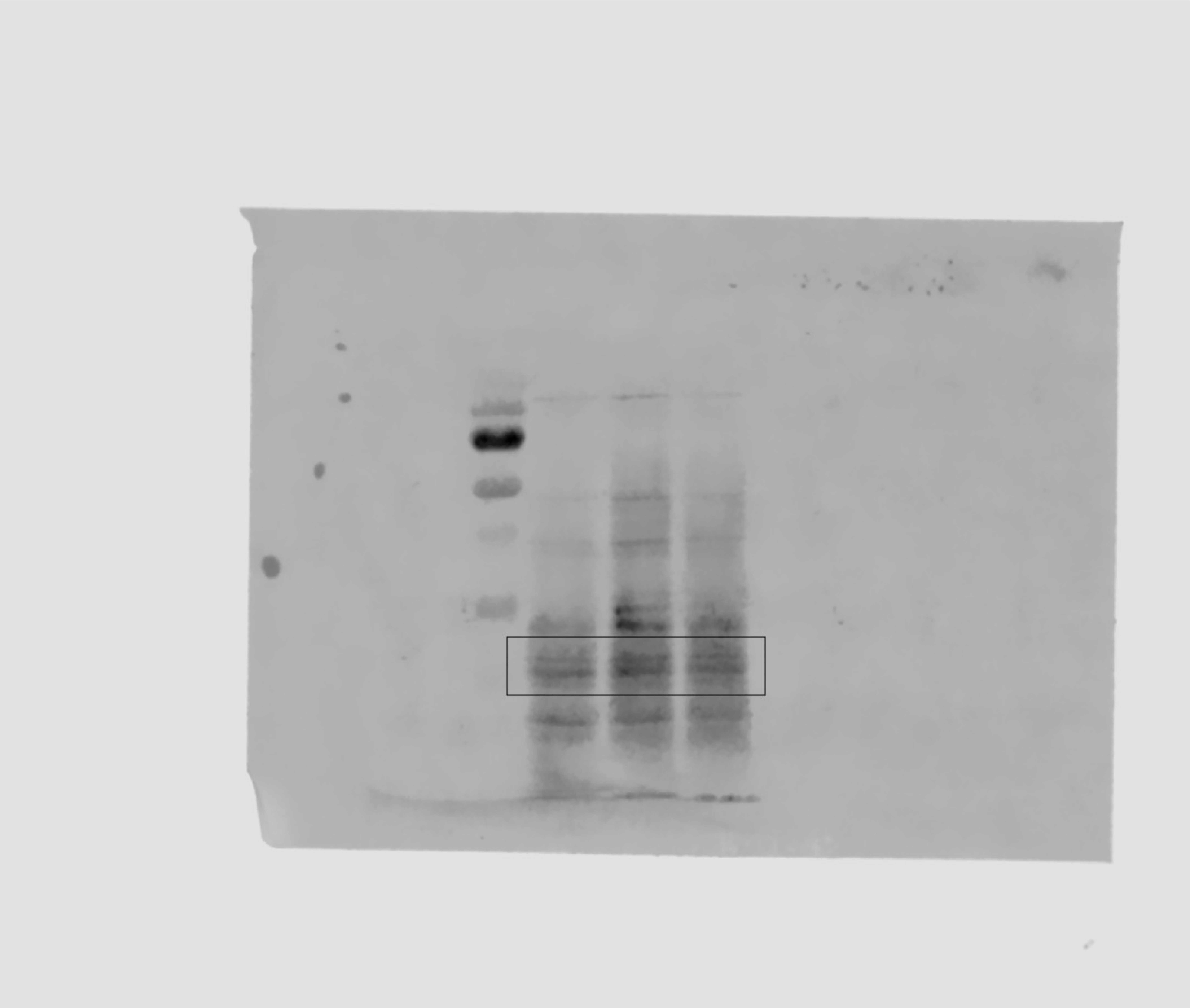

Cropped area for Figure 4-figure supplement 1A
Total Protein Stain

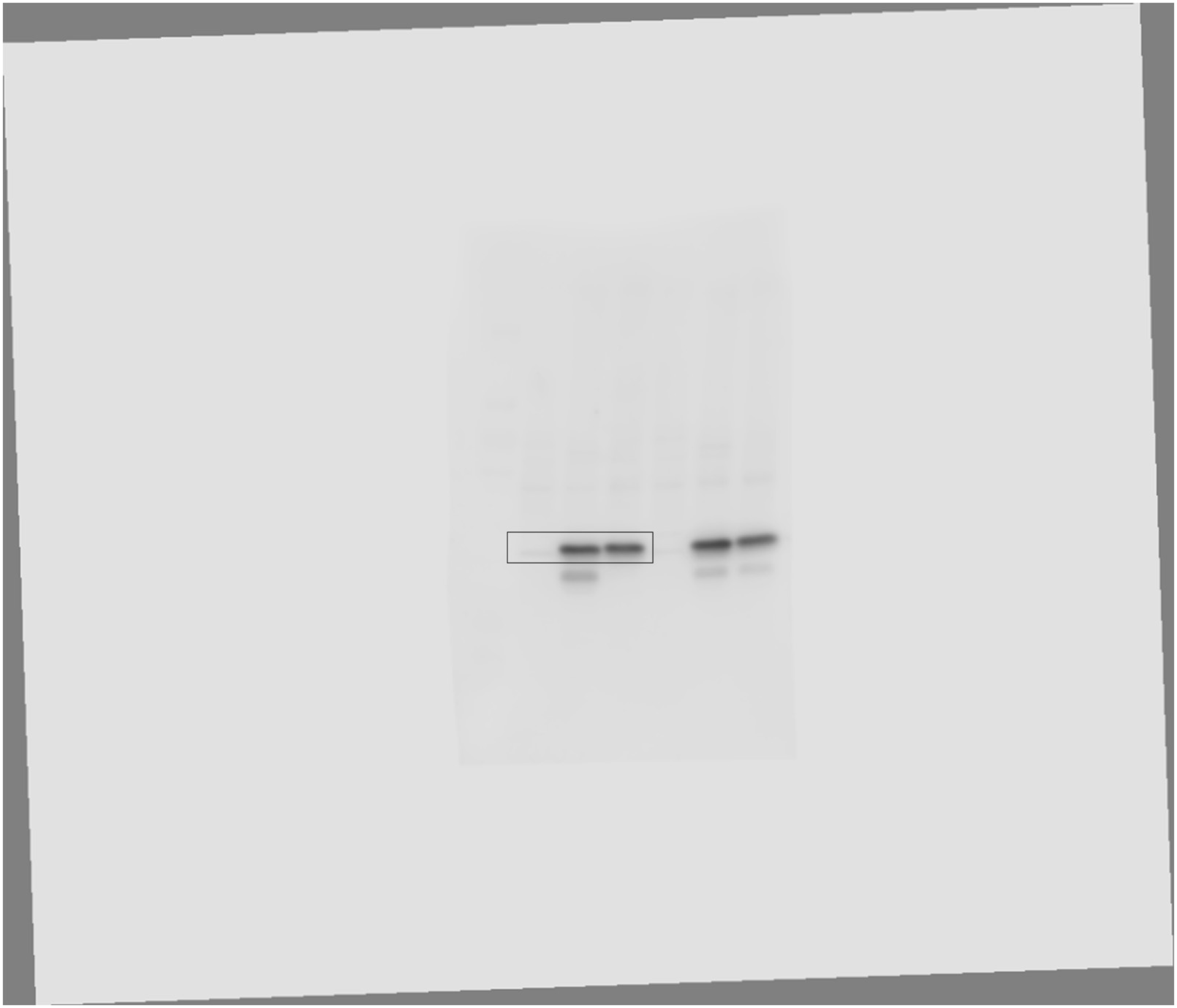

Cropped area for Figure 4-figure supplement1G
Eluate, Aac2

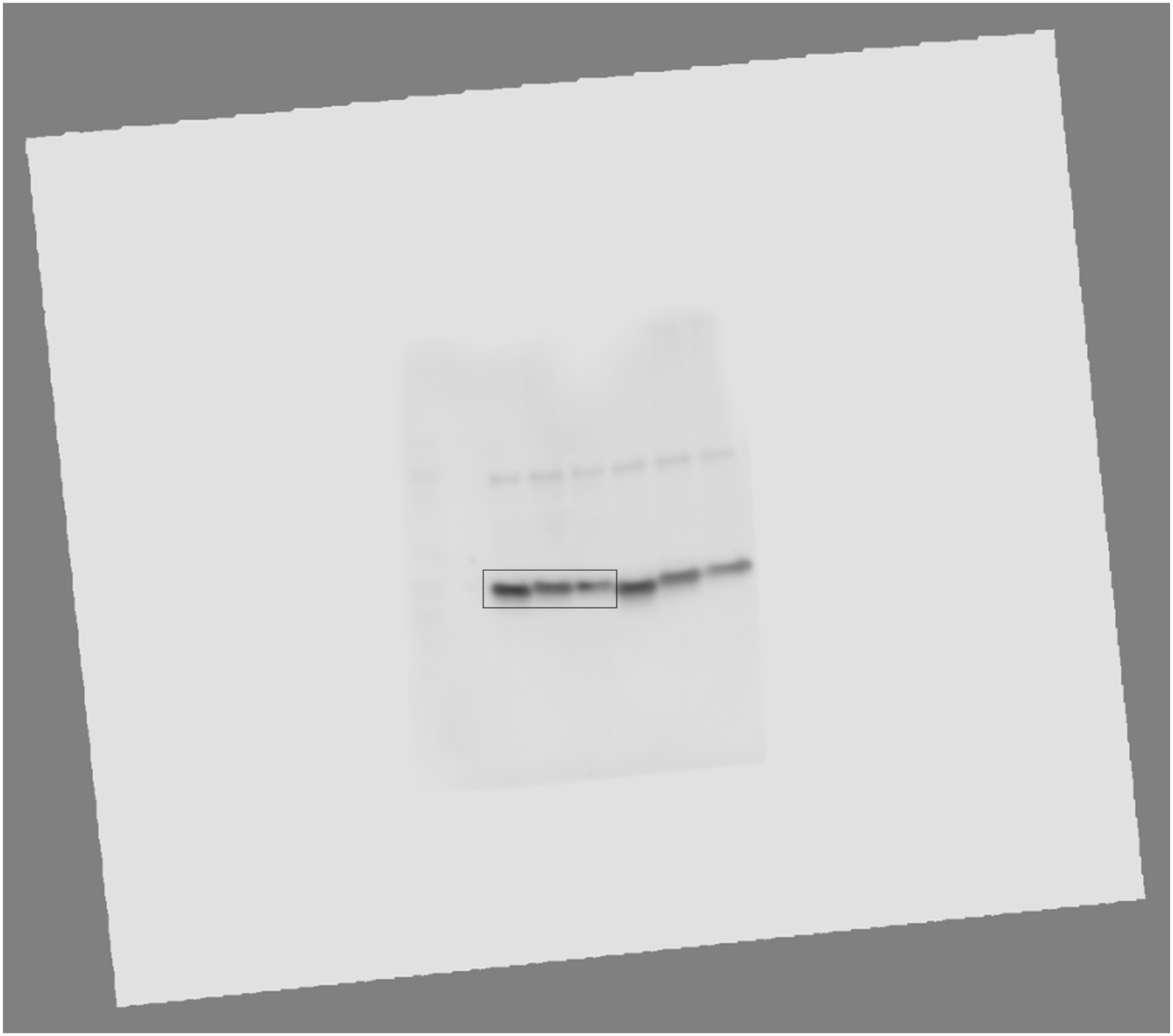

Cropped area for Figure 4-figure supplement1G
Input, Aac2

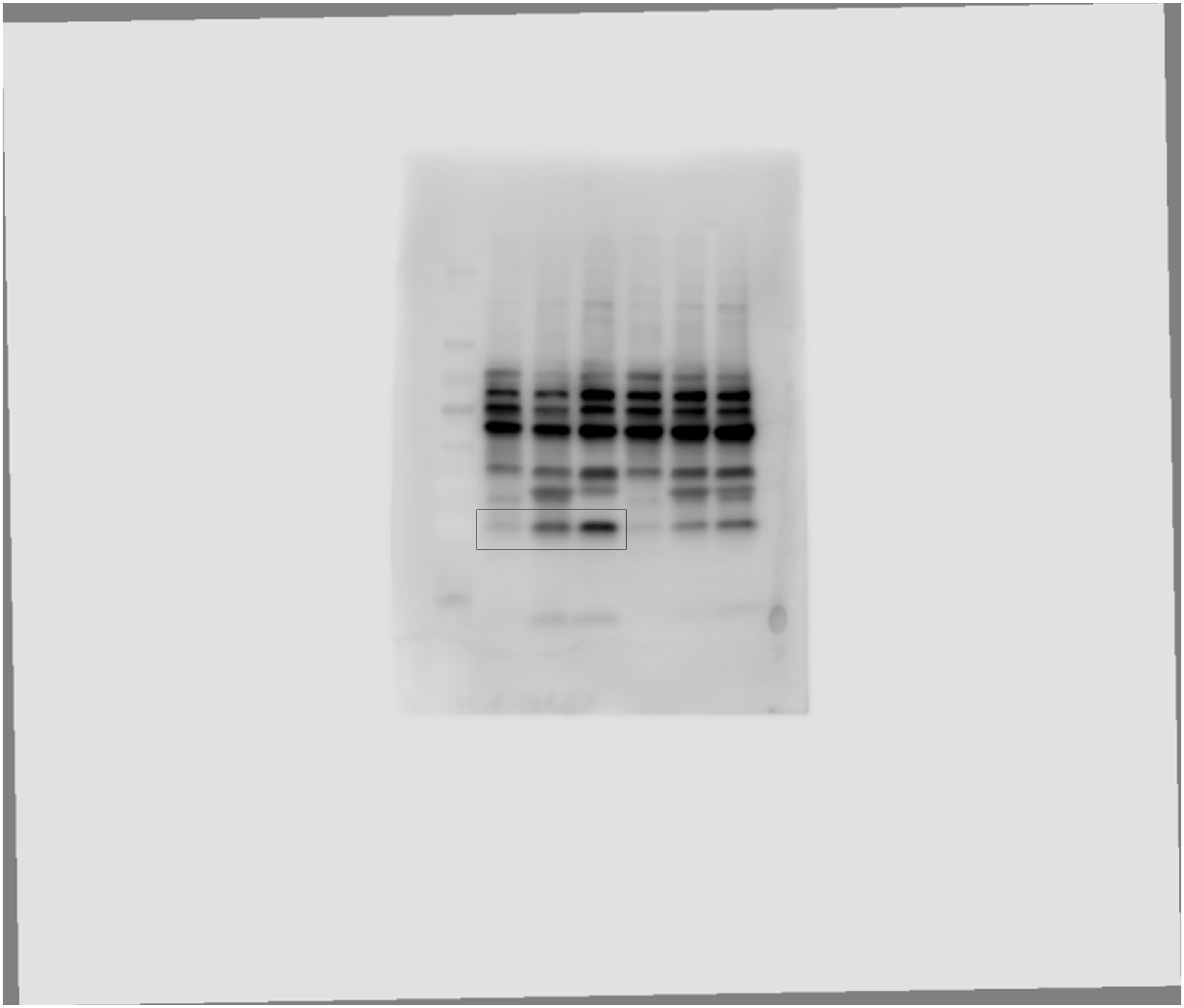

Cropped area for Figure 4-figure supplement1G
Eluate, Tim22

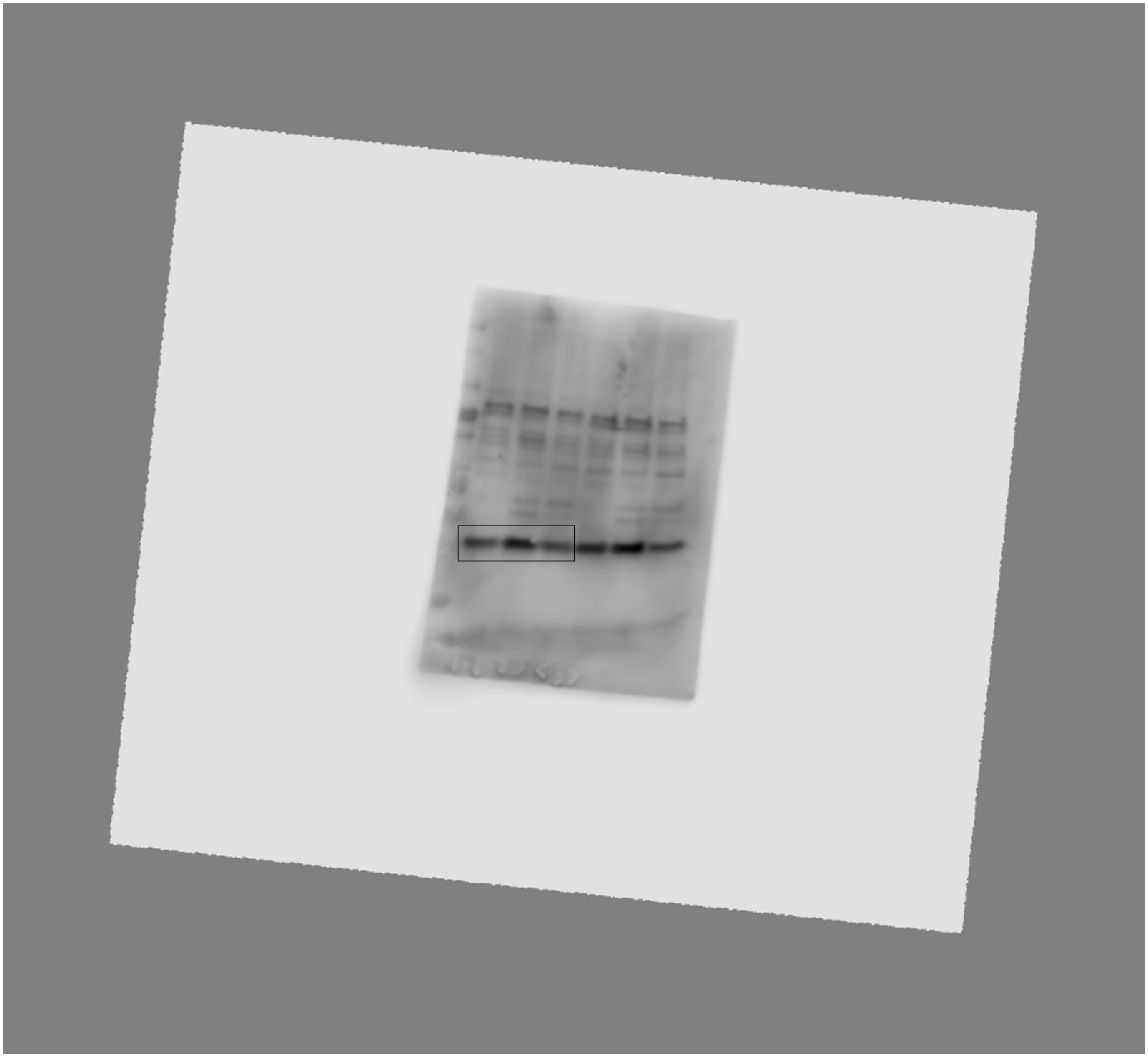

Cropped area for Figure 4-figure supplement1G
Input, Tim22
