# Supplementary material for: Mitochondrial protein import clogging as a mechanism of disease
Source: eLife. 2023 May 2;12:e84330. doi: 10.7554/eLife.84330 (PMC10208645; doi:10.7554/eLife.84330)
Supplement: Figure 4—figure supplement 2—source data 2. [file elife-84330-fig4-figsupp2-data2.zip › Figure 4-figure supplement 2-source datat 1/Figure 4-figure supplement 2-source data_annotated.pdf]

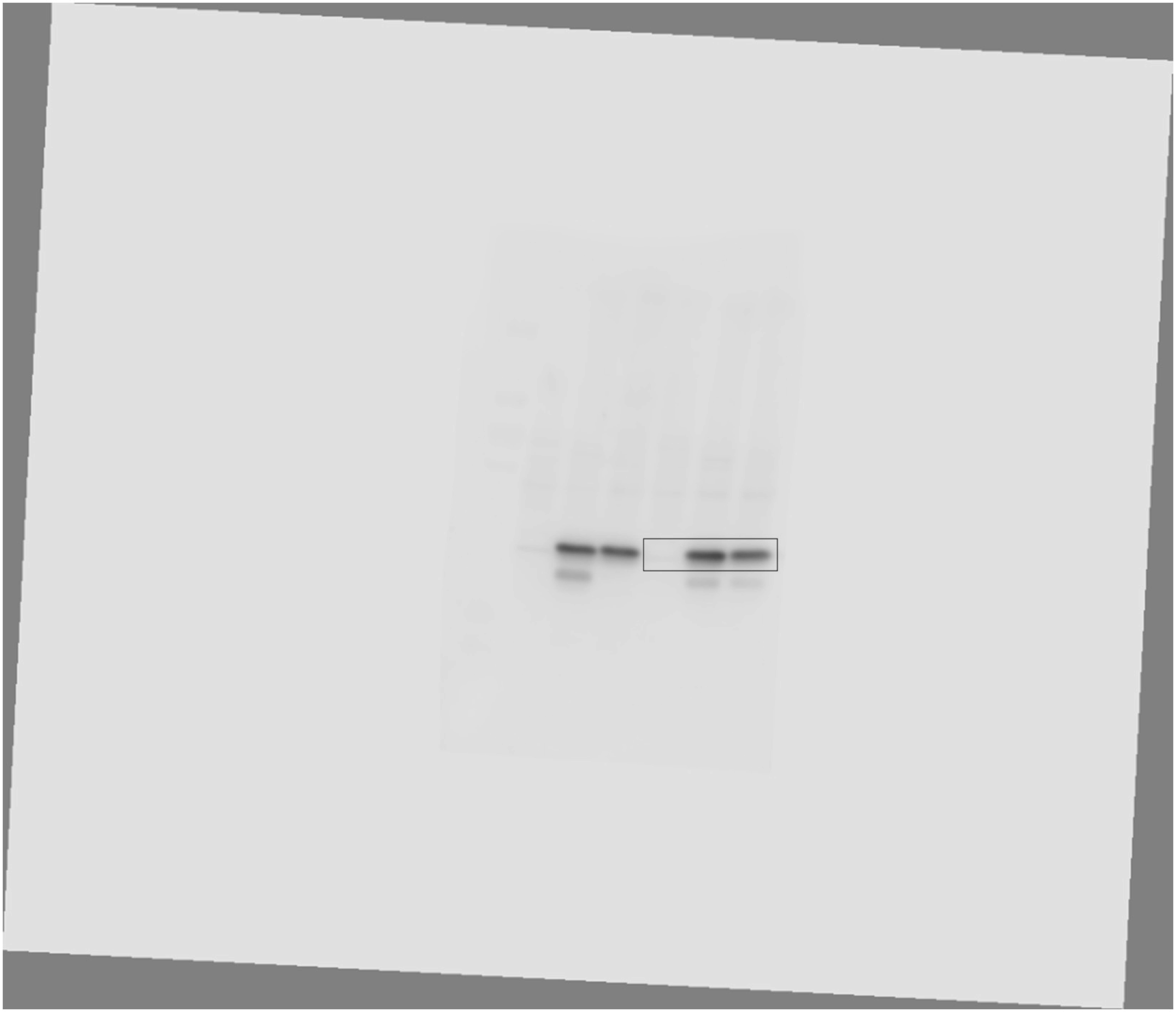

Cropped area for Figure 4-figure supplenent 2C
Eluate, Aac2

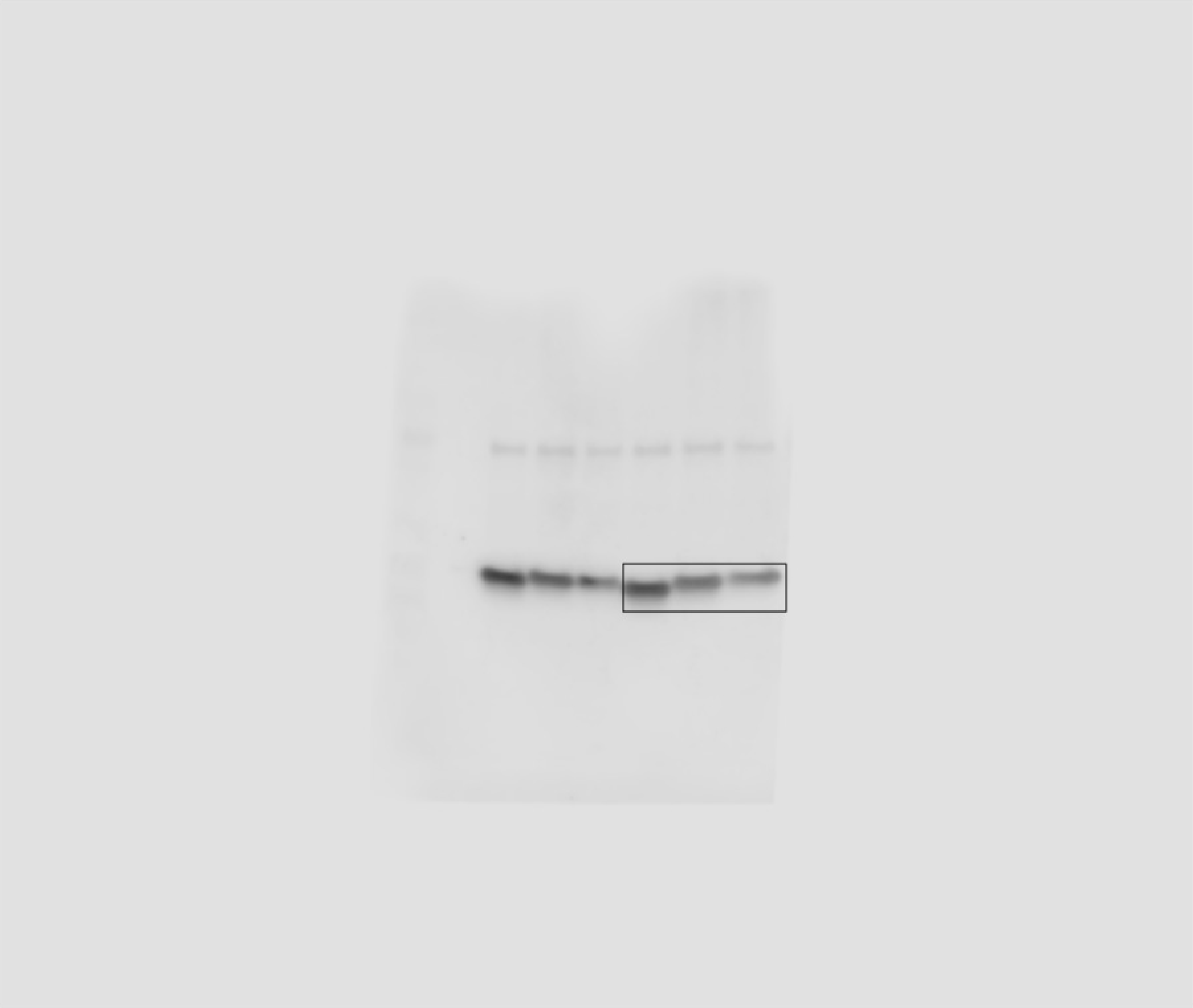

Cropped area for Figure 4-figure supplenent 2C
Input, Aac2

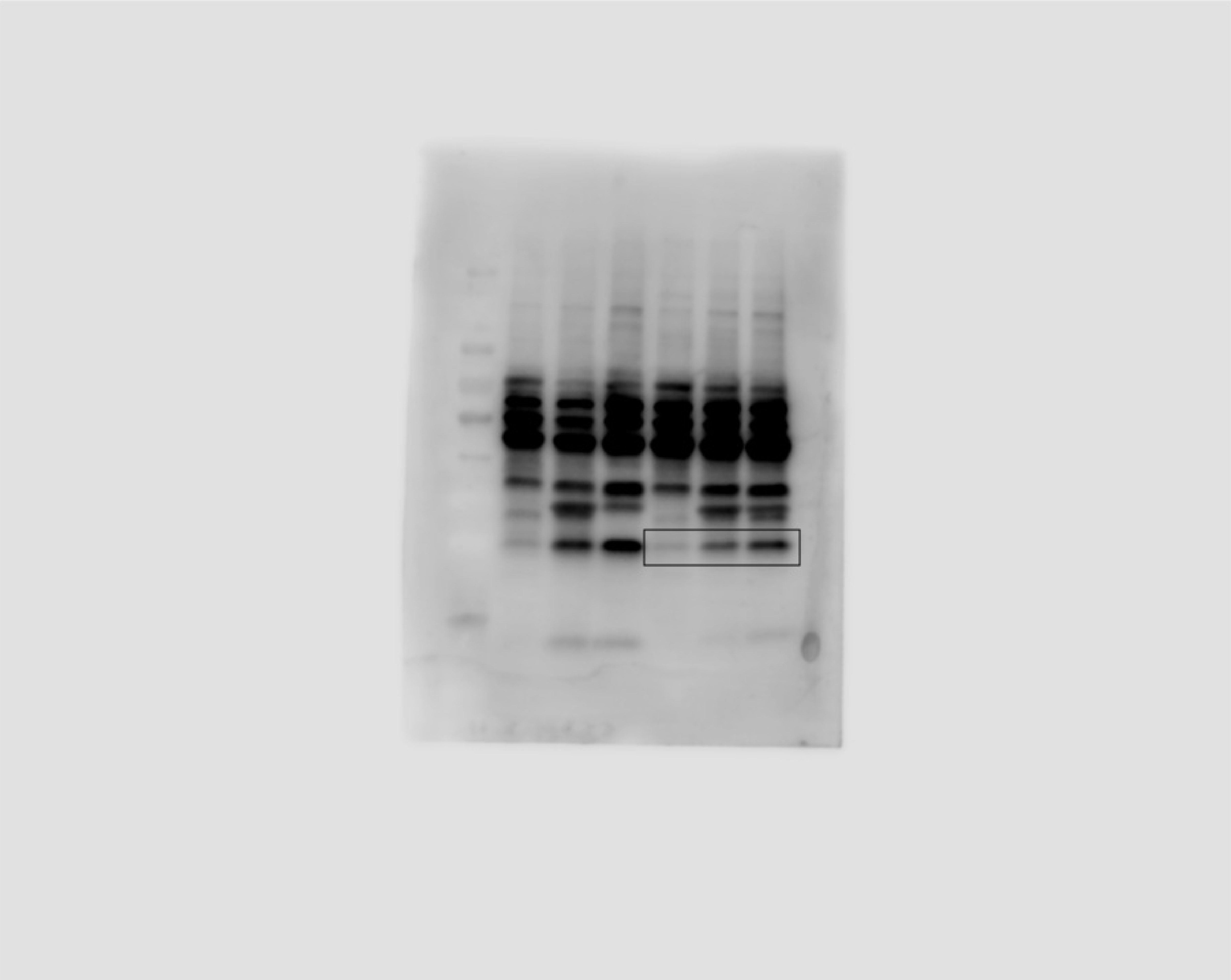

Cropped area for Figure 4-figure supplenent 2C
Eluate, Tim22

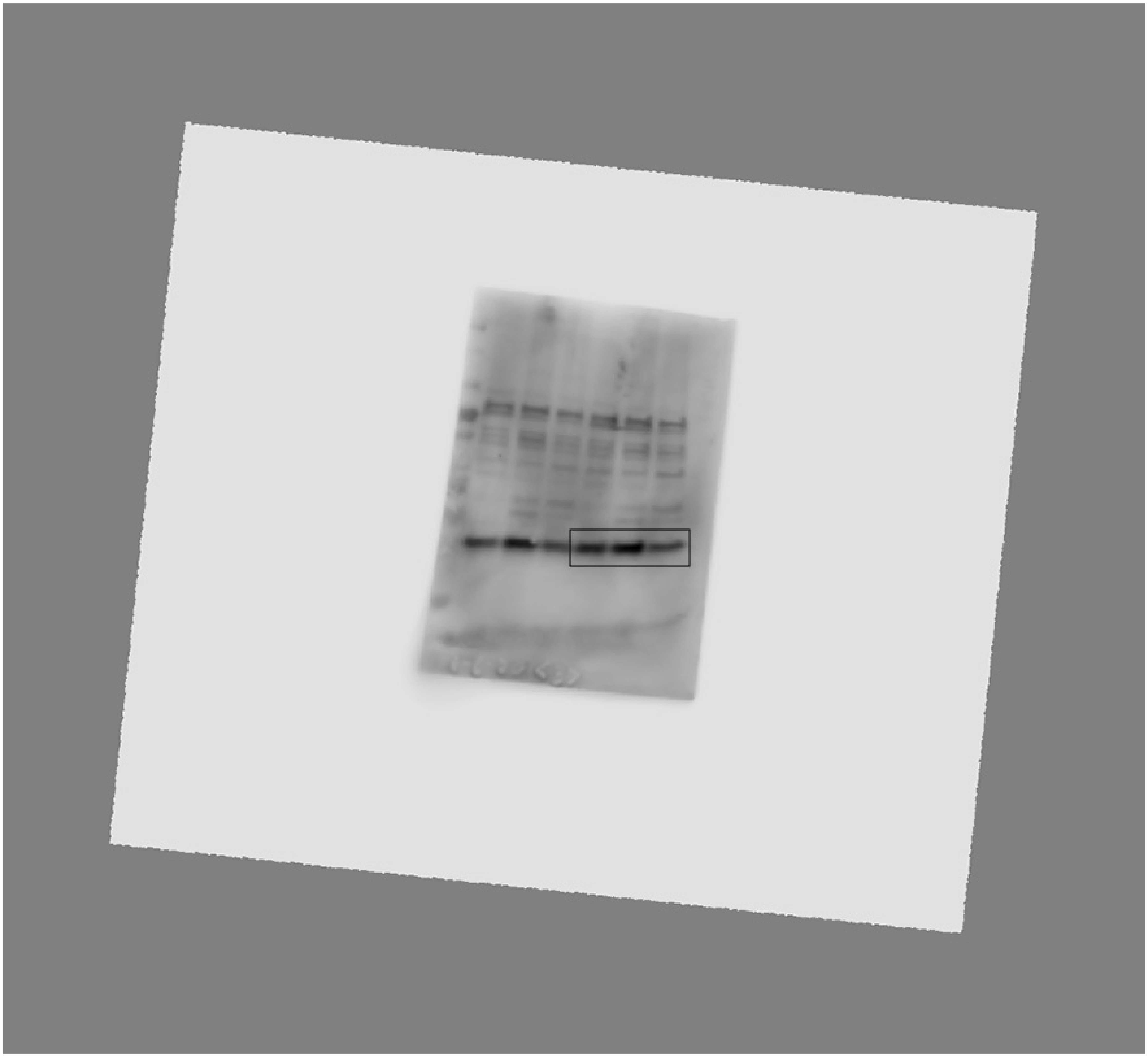

Cropped area for Figure 4-figure supplenent 2C
Input, Tim22
