# Supplementary material for: Mitochondrial protein import clogging as a mechanism of disease
Source: eLife. 2023 May 2;12:e84330. doi: 10.7554/eLife.84330 (PMC10208645; doi:10.7554/eLife.84330)
Supplement: Figure 5—source data 2. [file elife-84330-fig5-data2.zip › Figure 5-source data 1/Figure 5-source data_annotated.pdf]

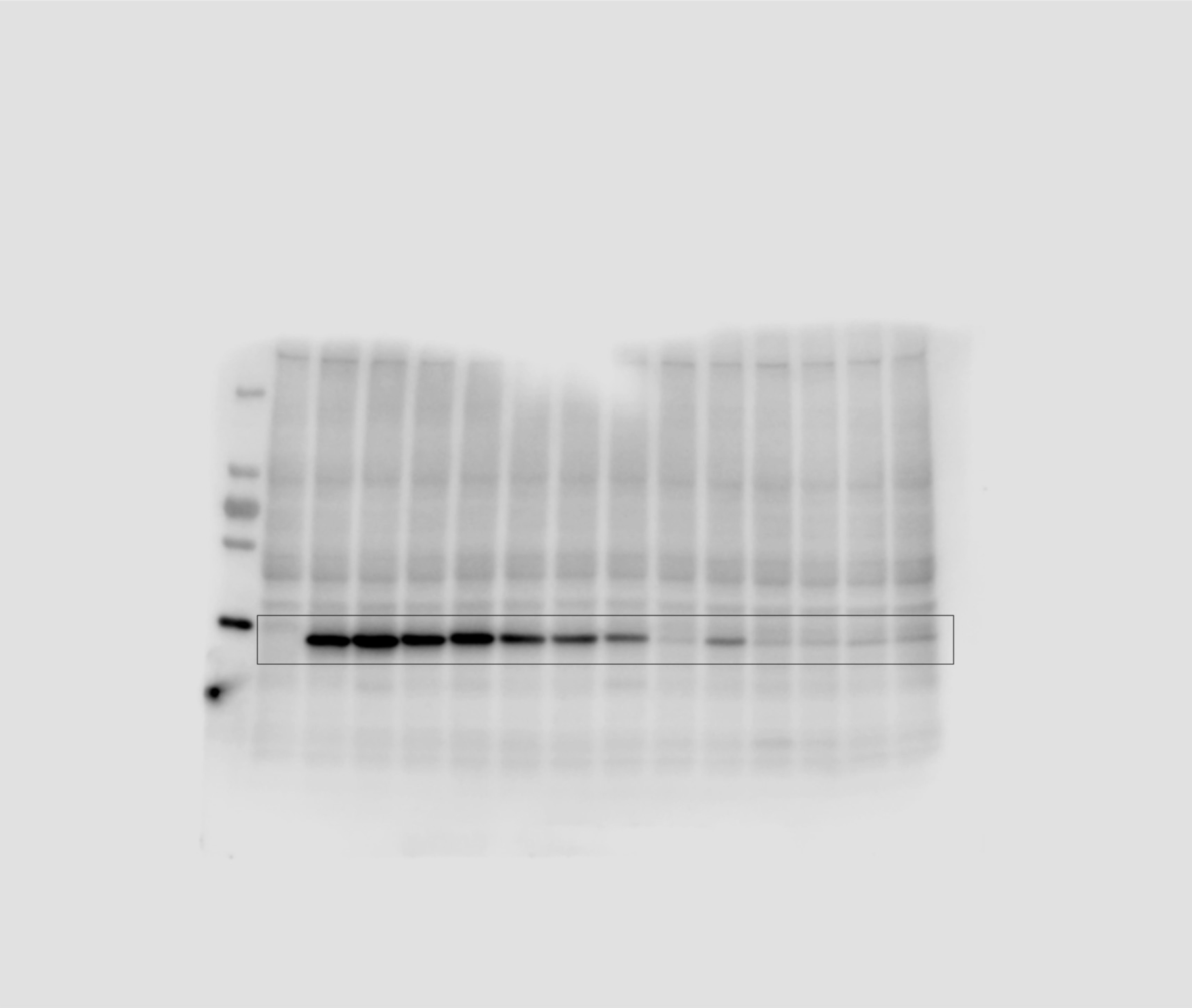

Cropped area for Figure 5A
HA

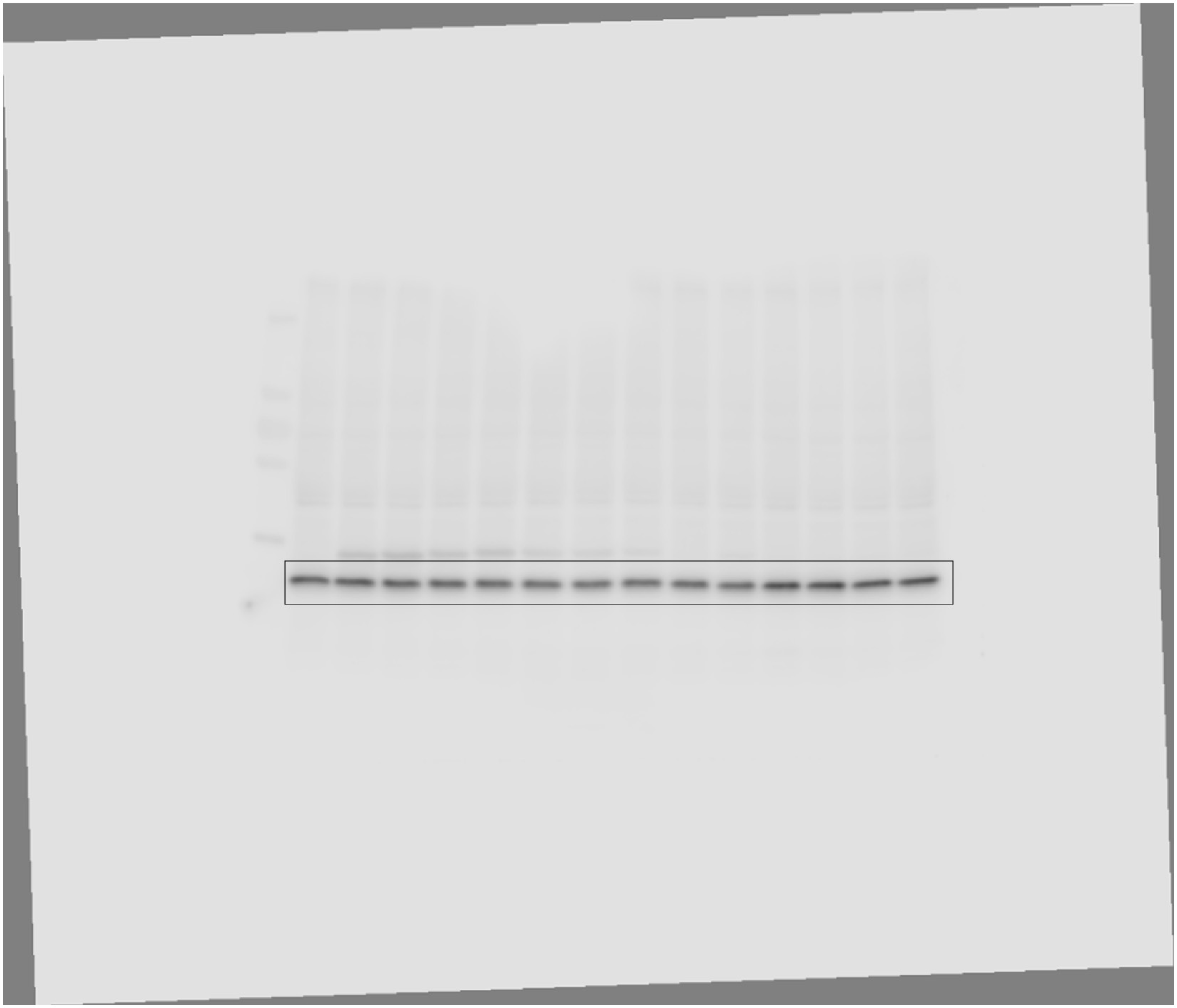

Cropped area for Figure 5A
TFAM

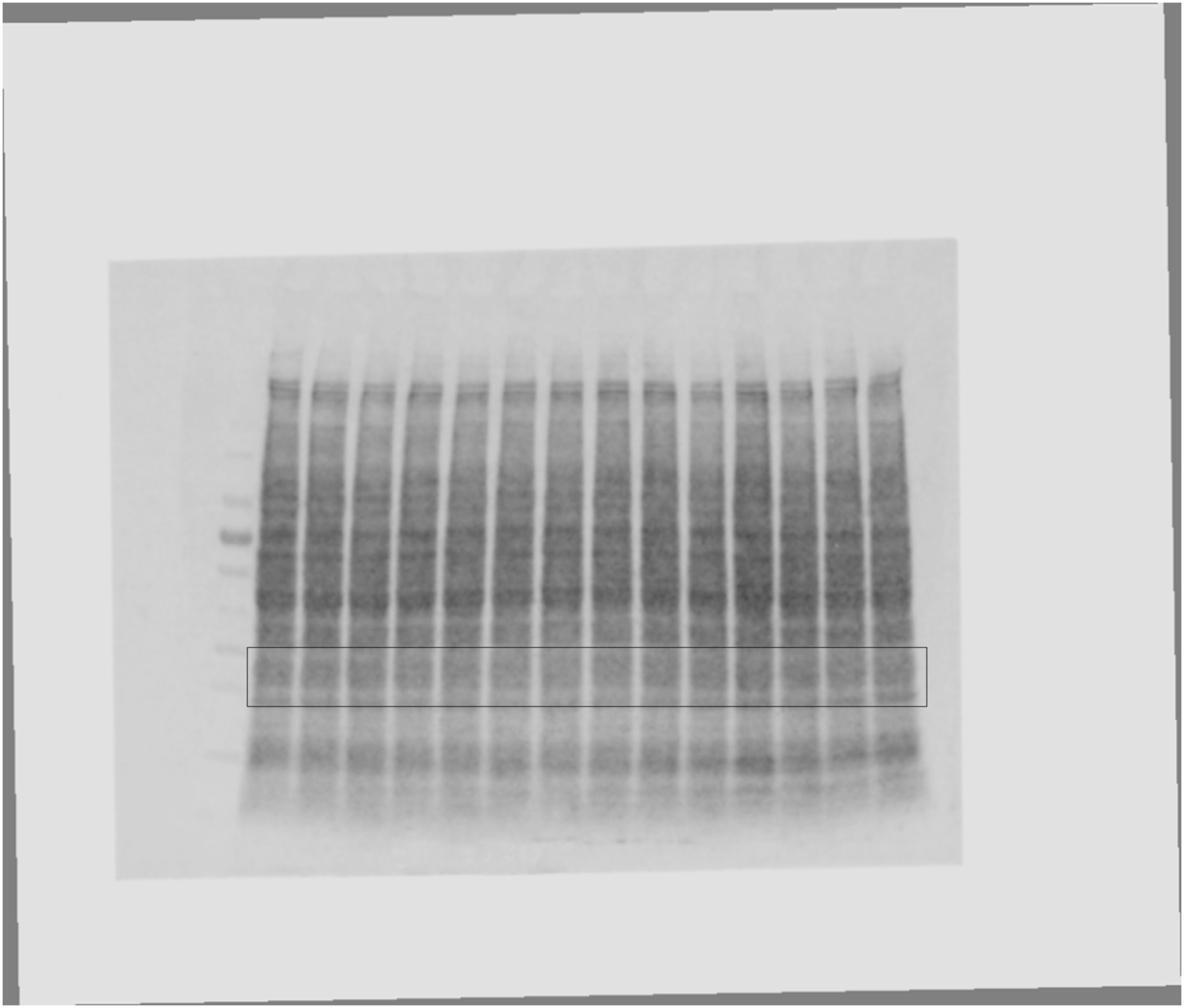

Cropped area for Figure 5A
Total Protein Stain

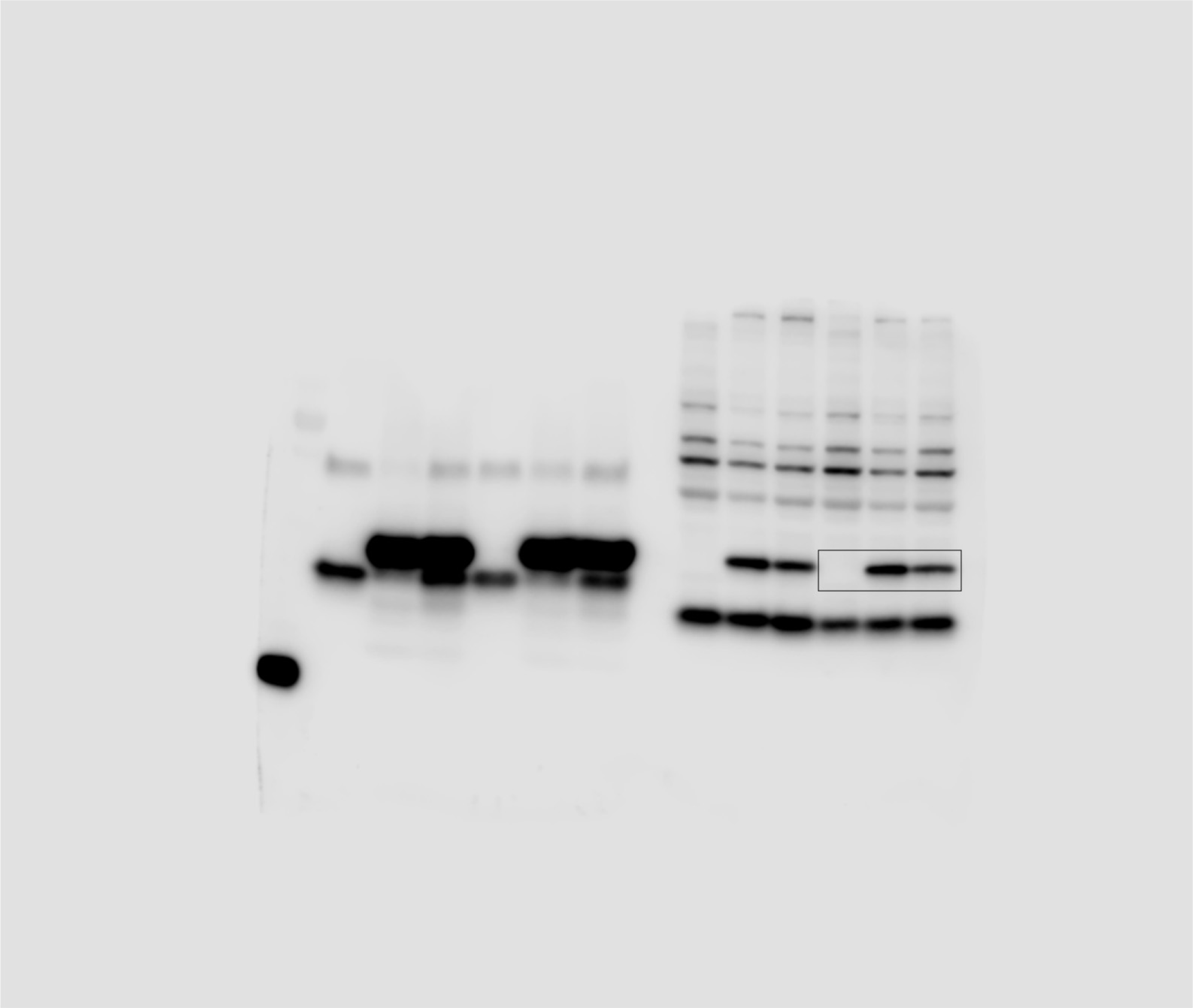

Cropped area for Figure 5B
Input, anti-HA

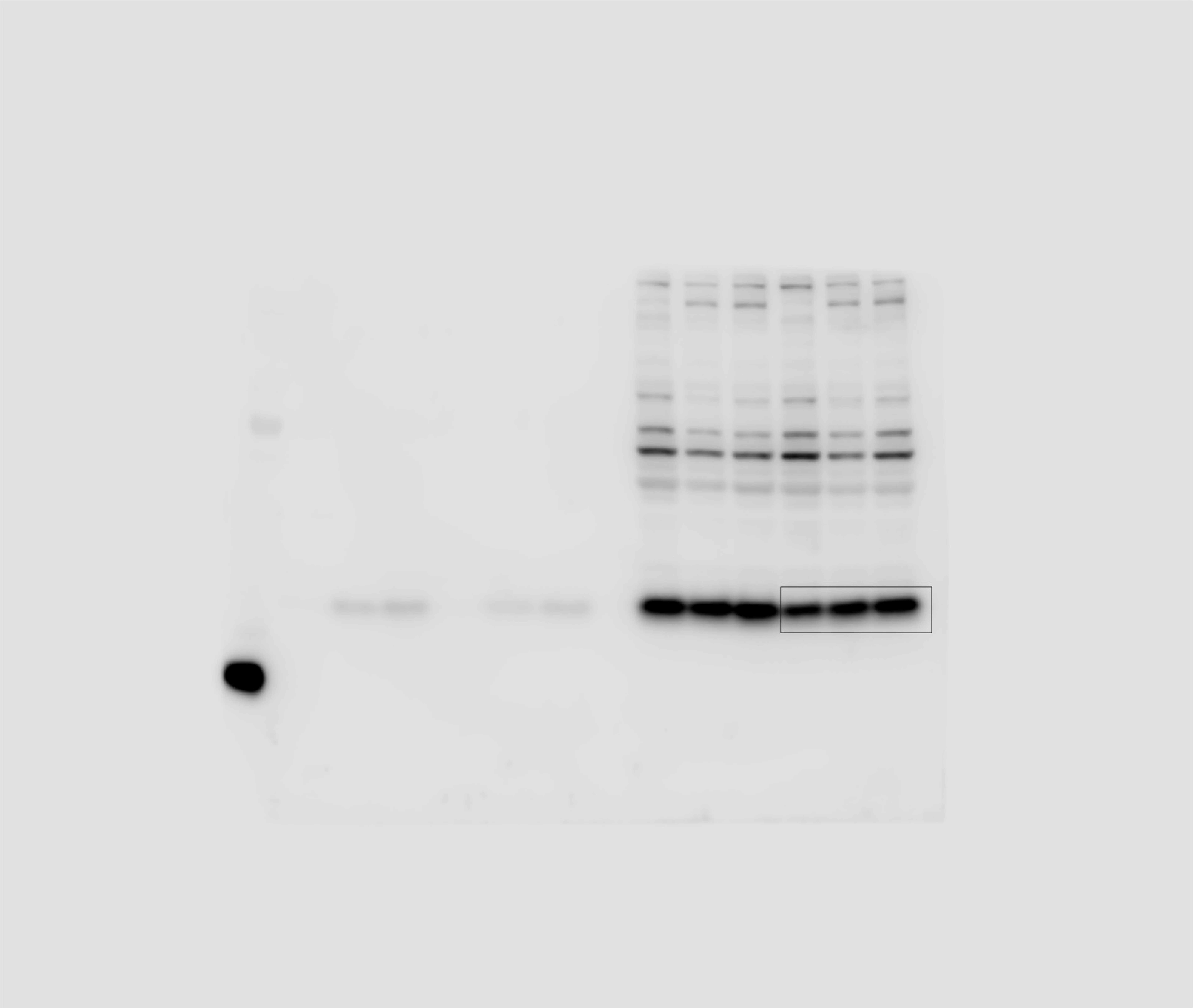

Cropped area for Figure 5B
Input, Tim22

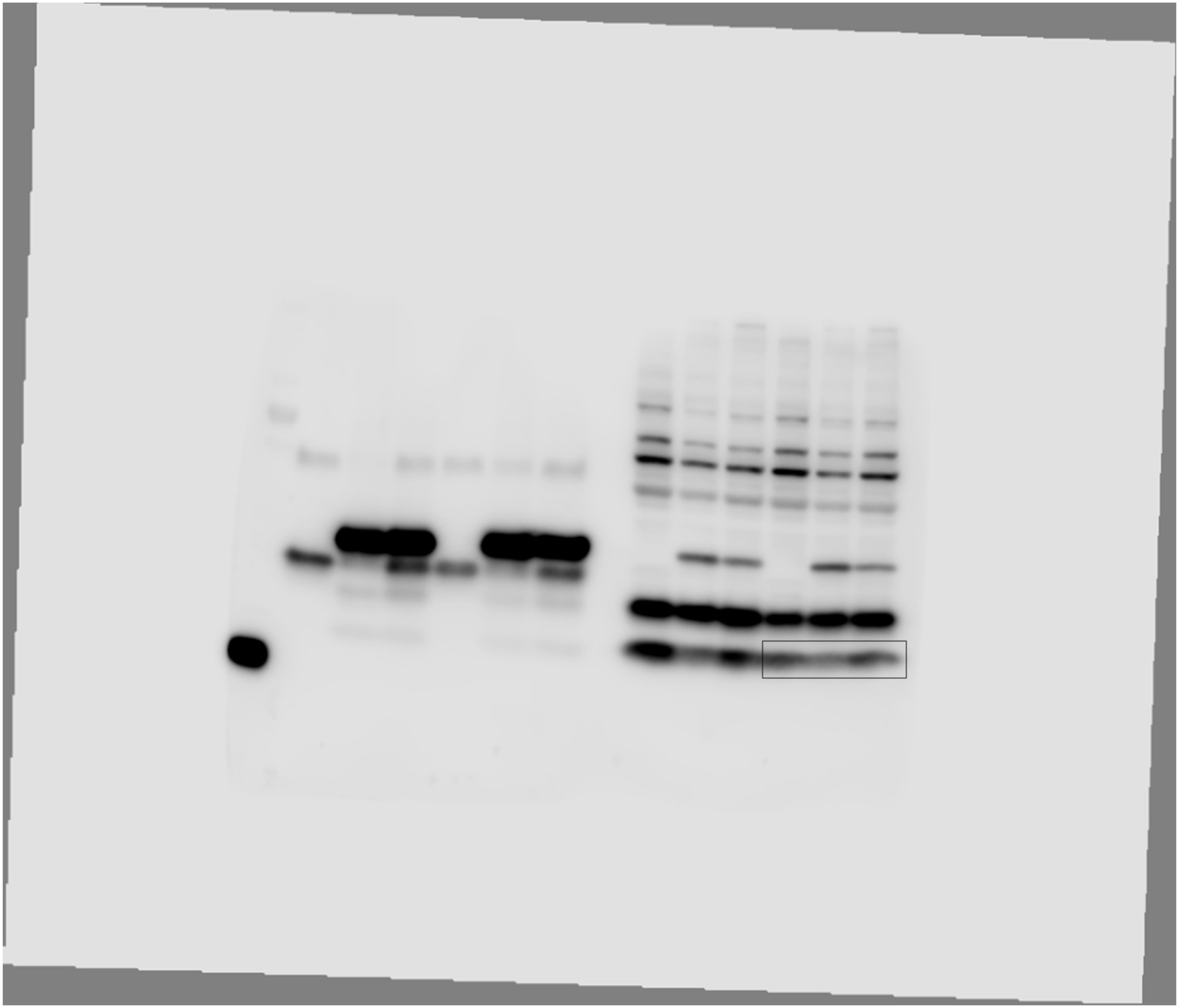

Cropped area for Figure 5B
Input, Tom20

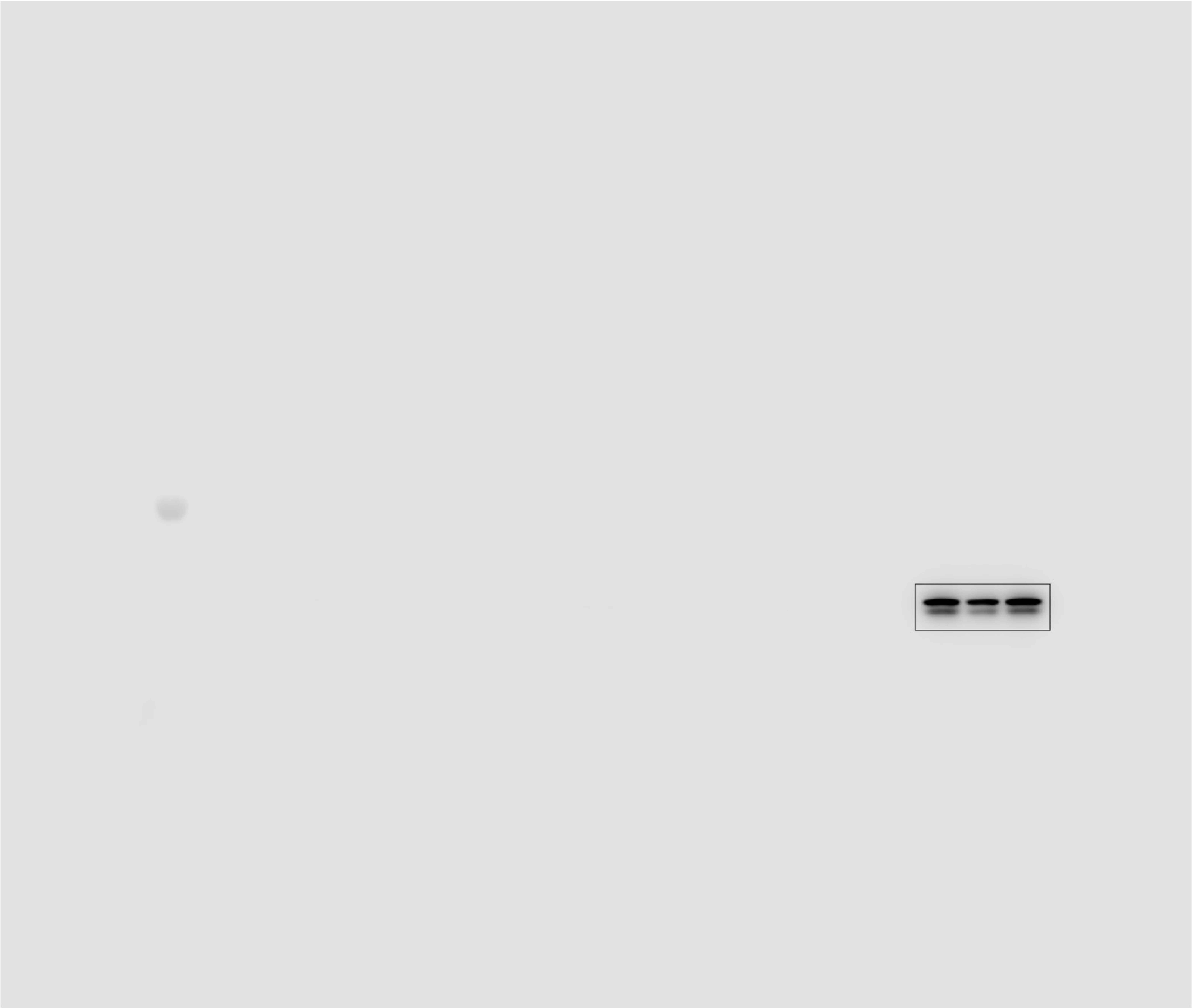

Cropped area for Figure 5B
Input, Tom40

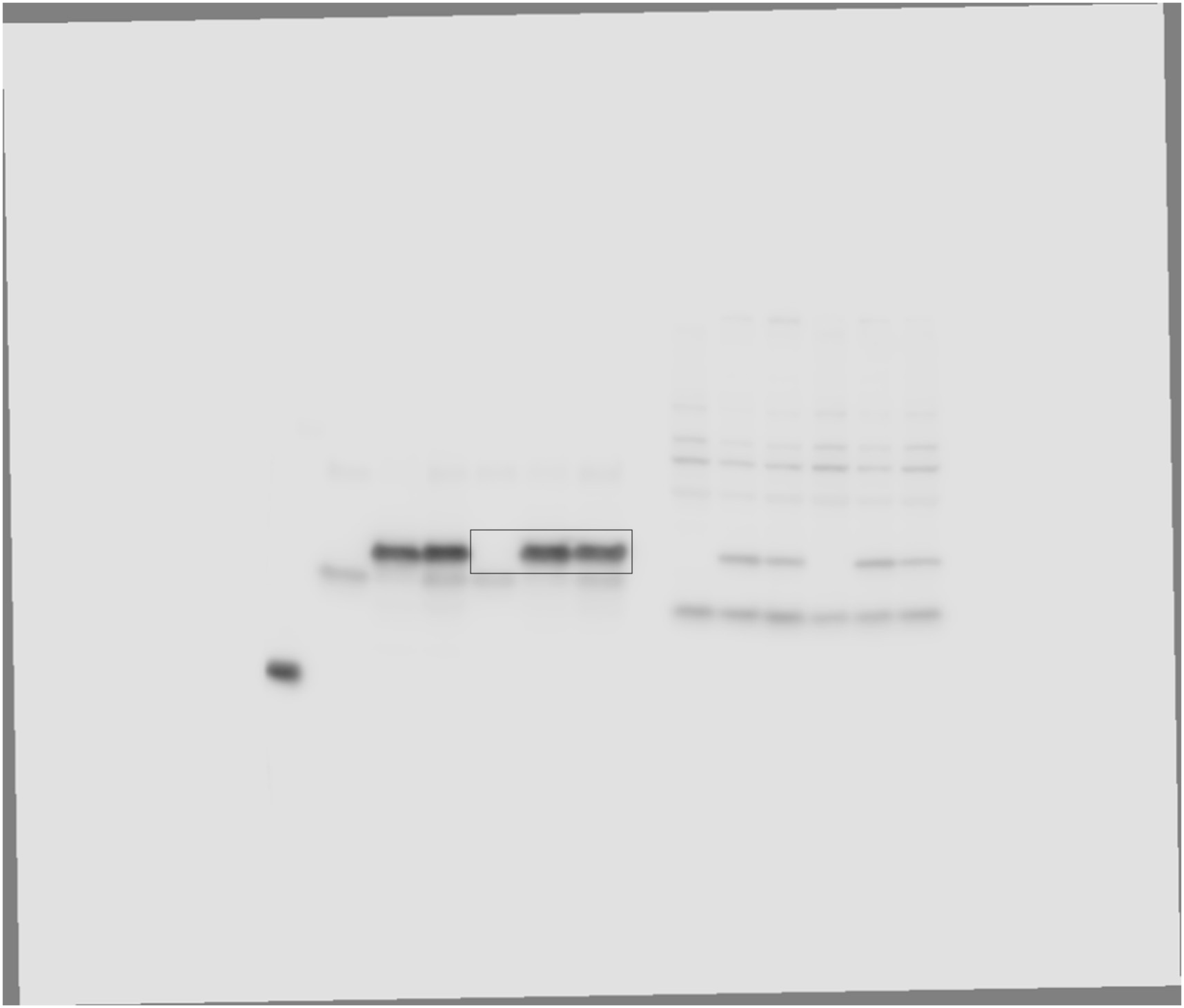

Cropped area for Figure 5B
IP:HA, anti-HA

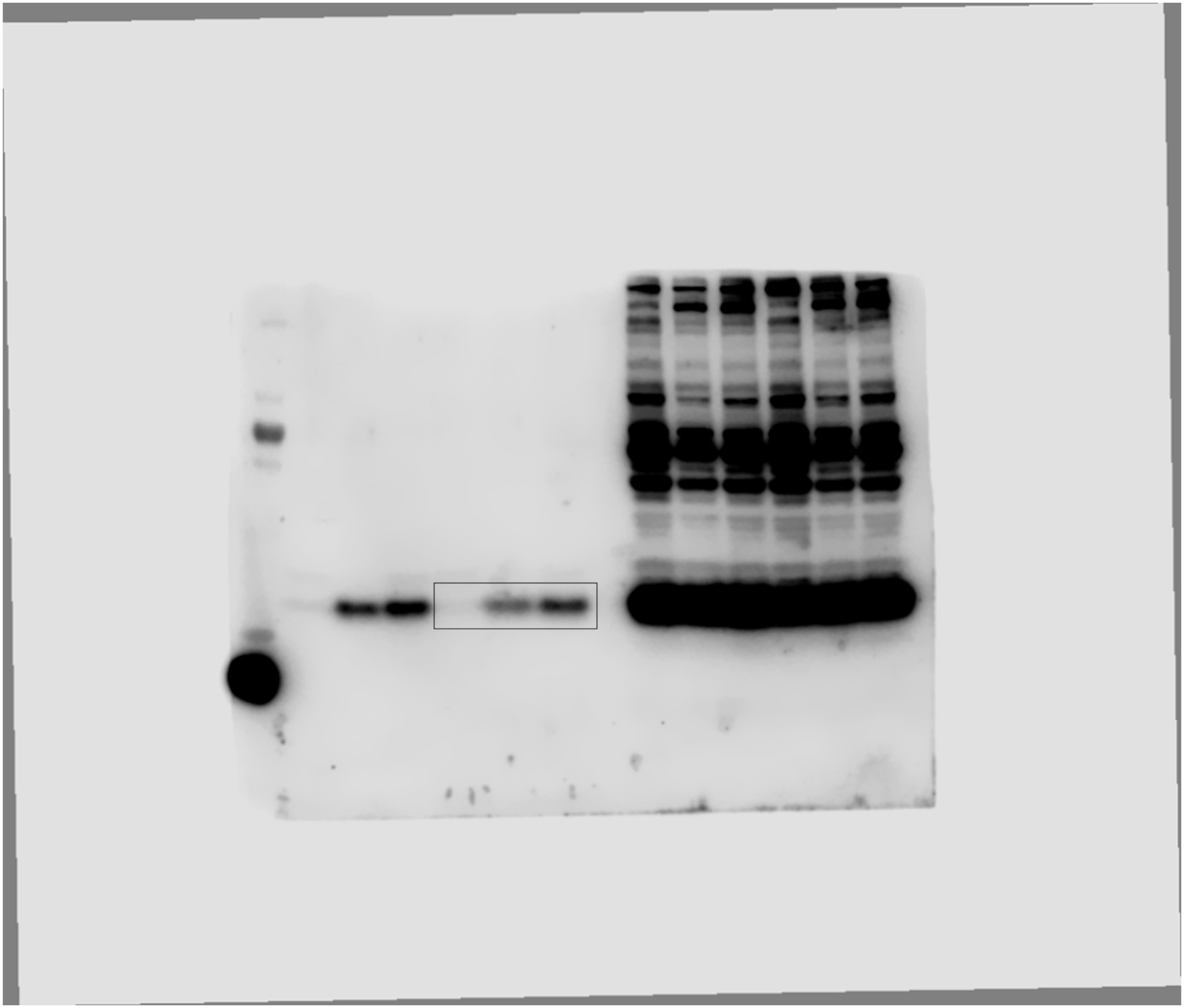

Cropped area for Figure 5B
IP: HA, Tim22

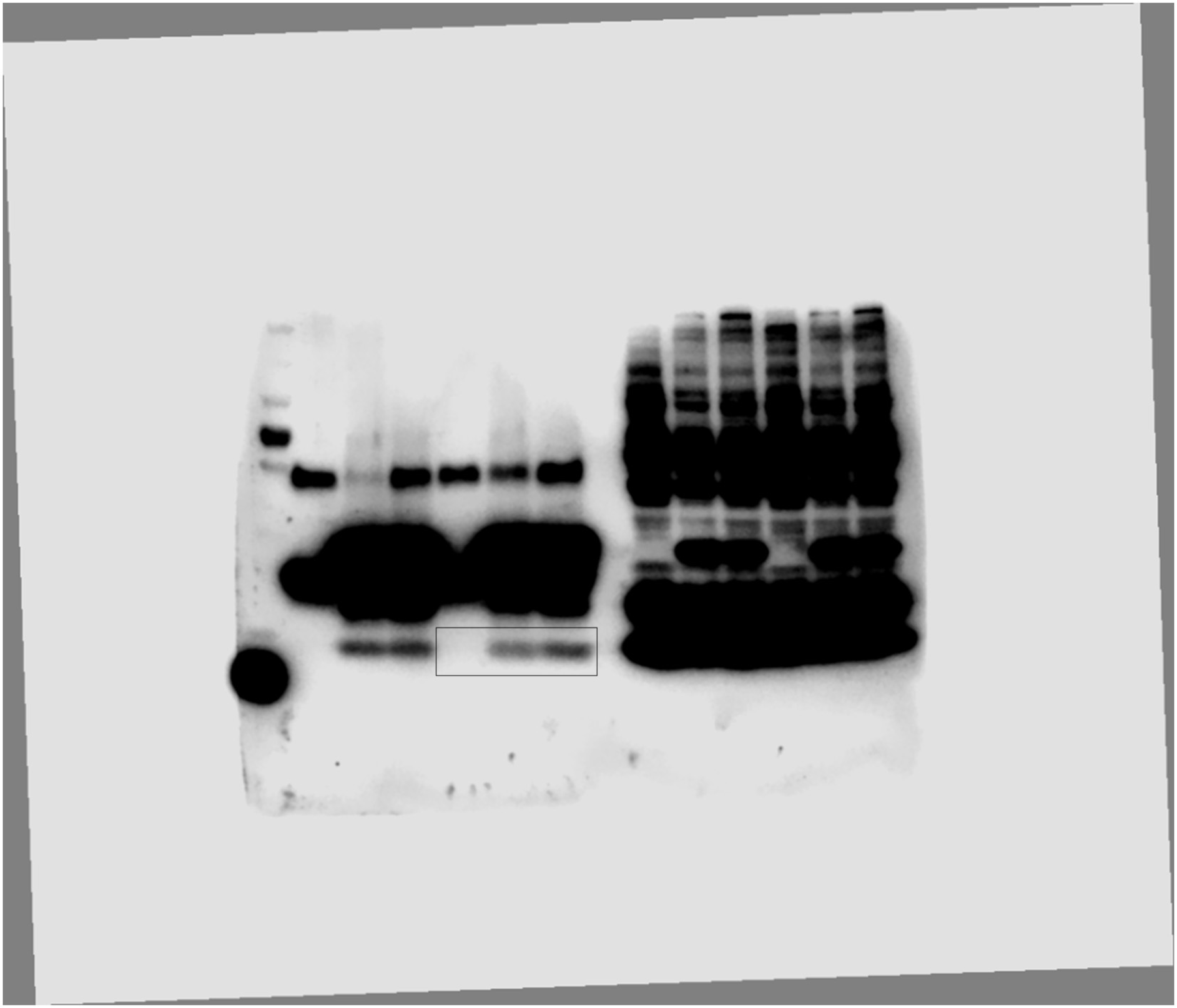

Cropped area for Figure 5B
IP: HA, Tom20

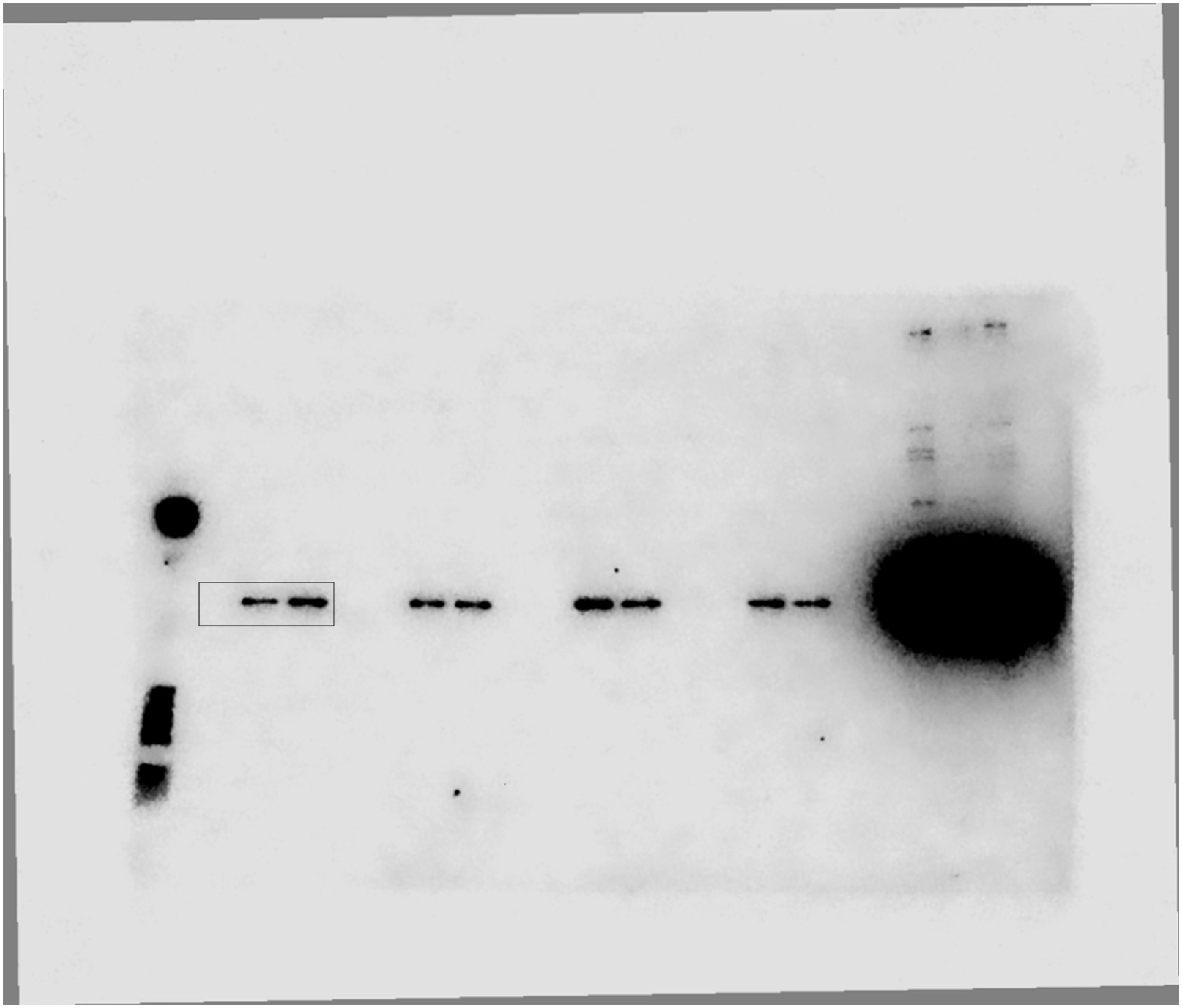

Cropped area for Figure 5B
IP: HA, Tom40

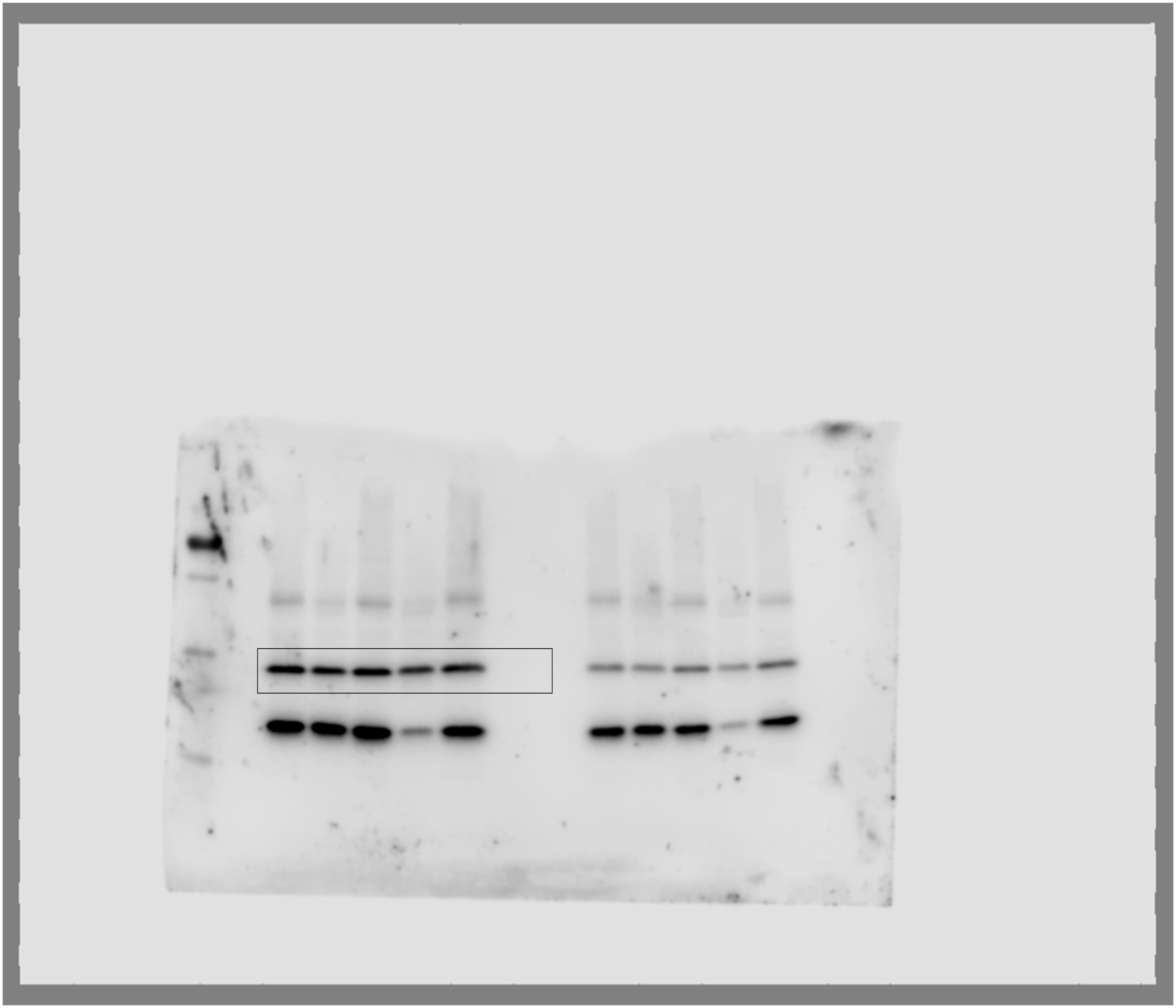

Cropped area for Figure 5D
Ant1-HA, anti-HA

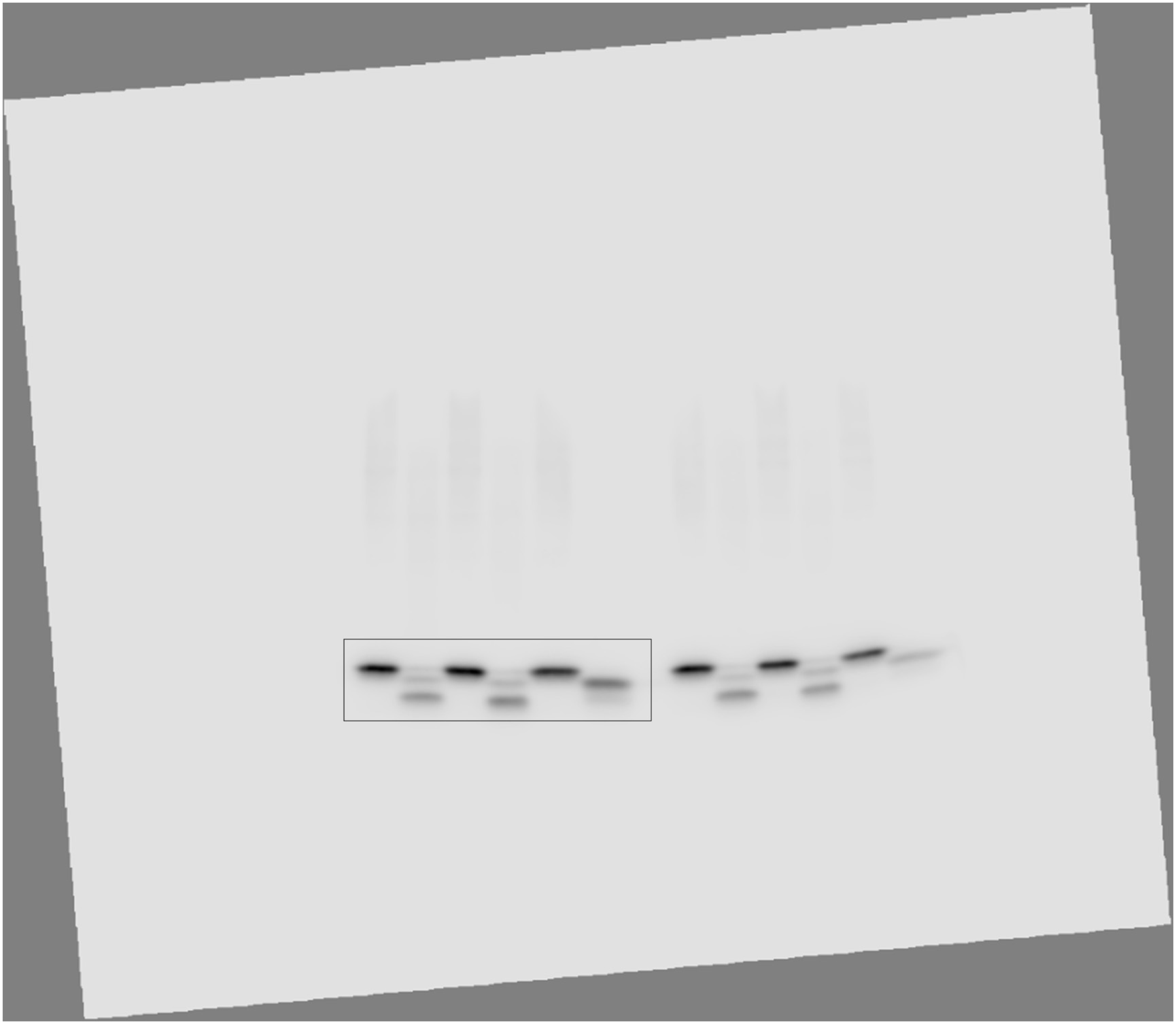

Cropped area for Figure 5D
Ant1-HA, anti-Tom20

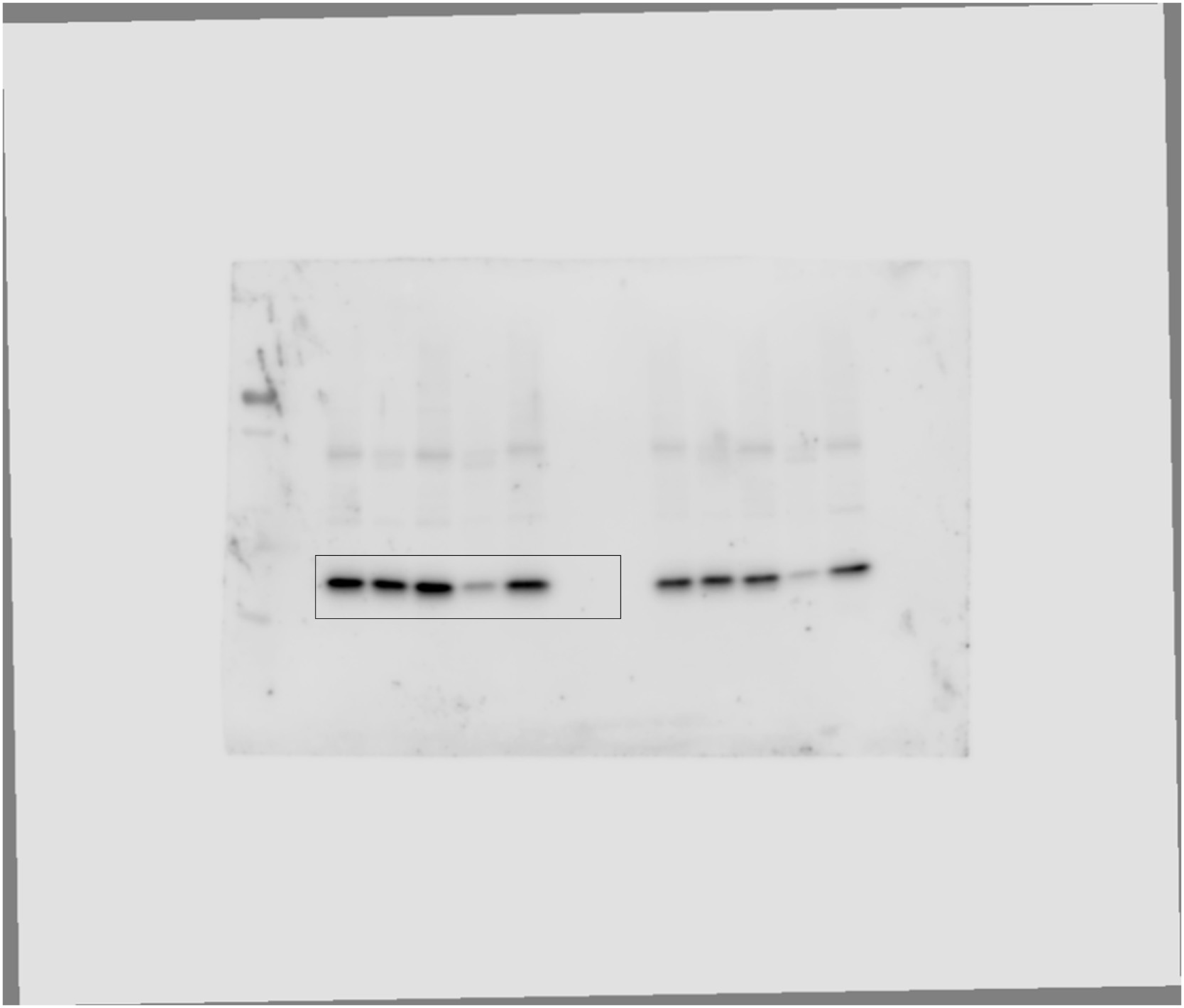

Cropped area for Figure 5D
Ant1-HA, anti-Smac

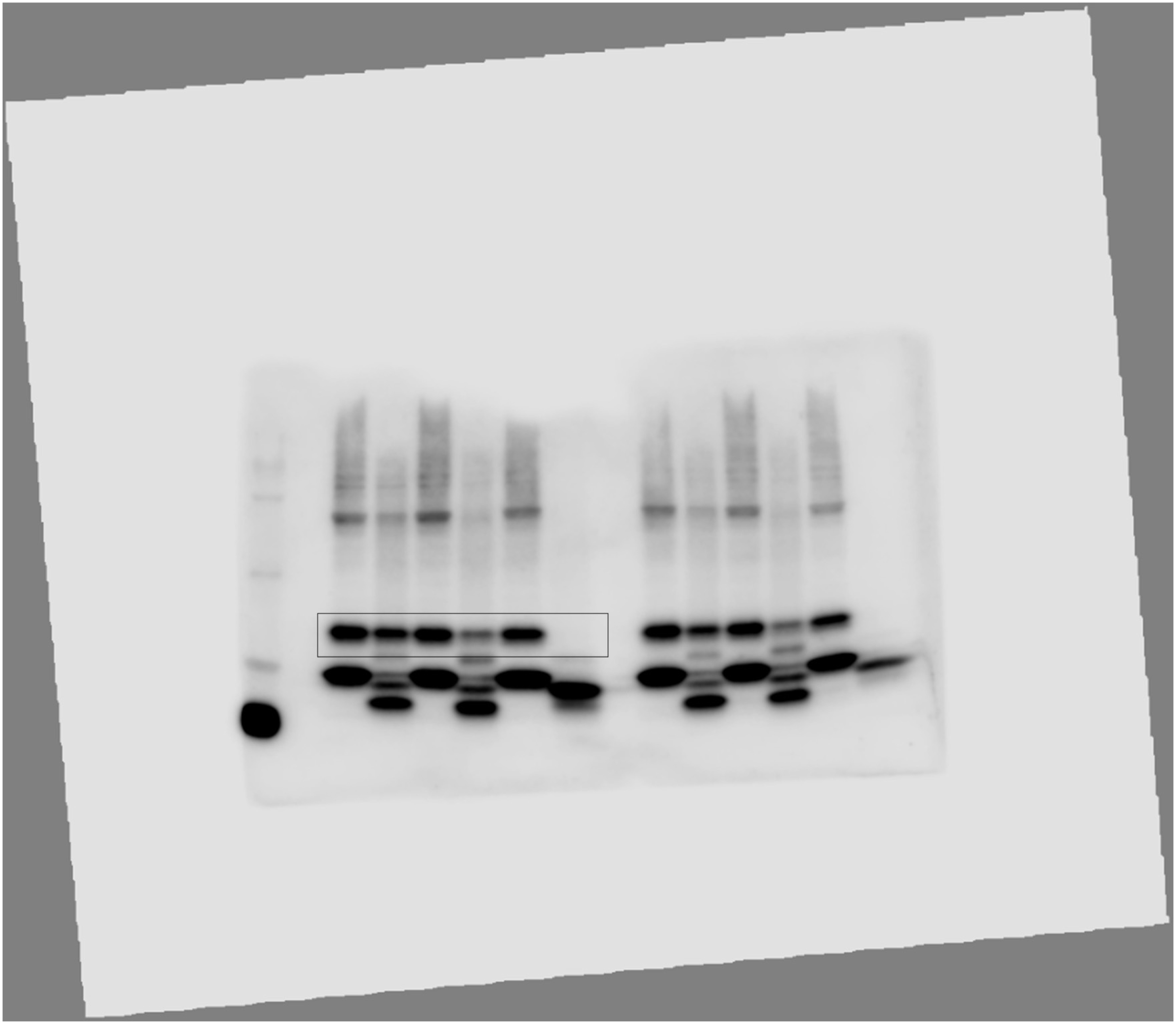

Cropped area for Figure 5D
Ant1-HA, anti-Tim22

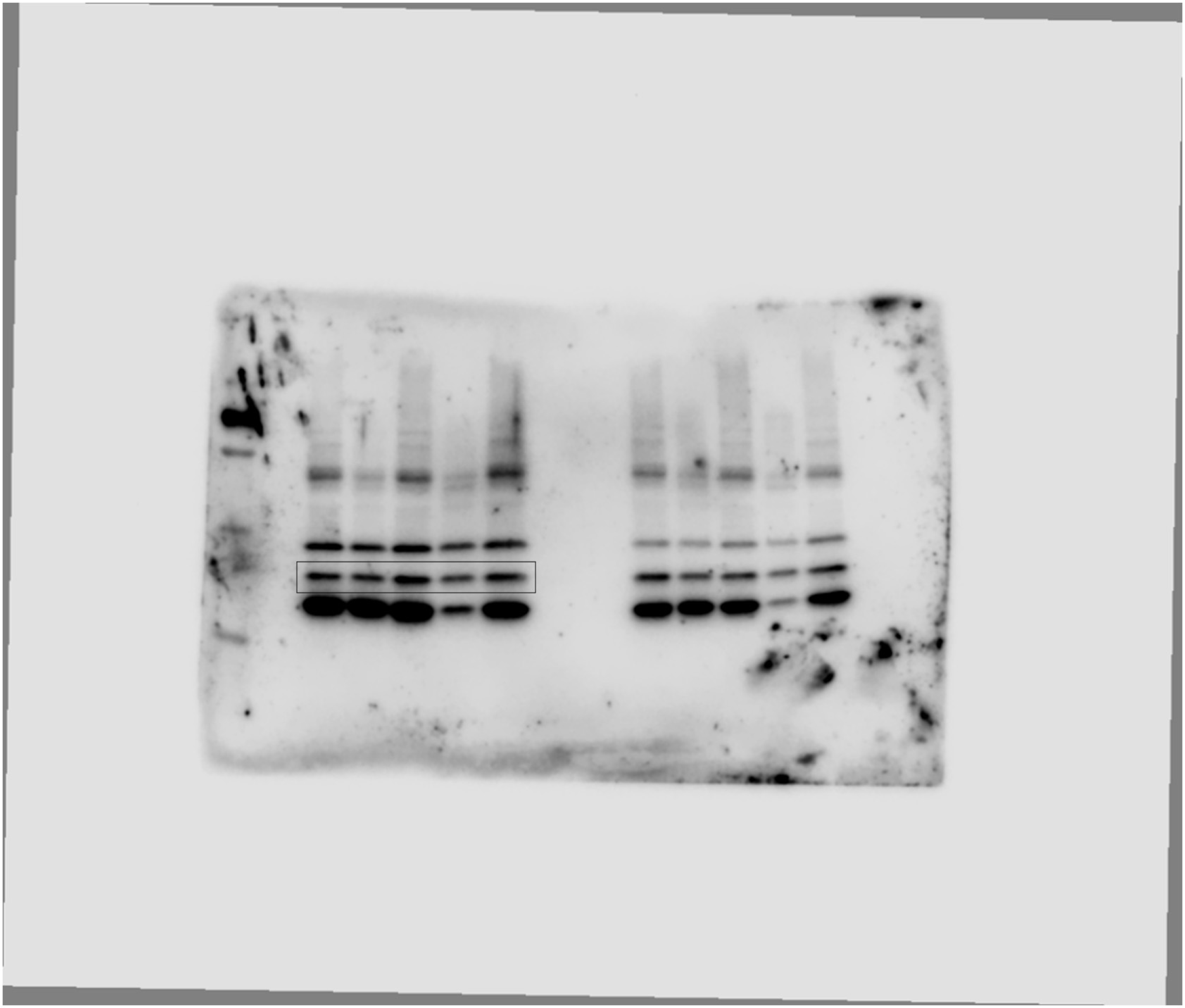

Cropped area for Figure 5D
Ant1-HA, anti-TFAM

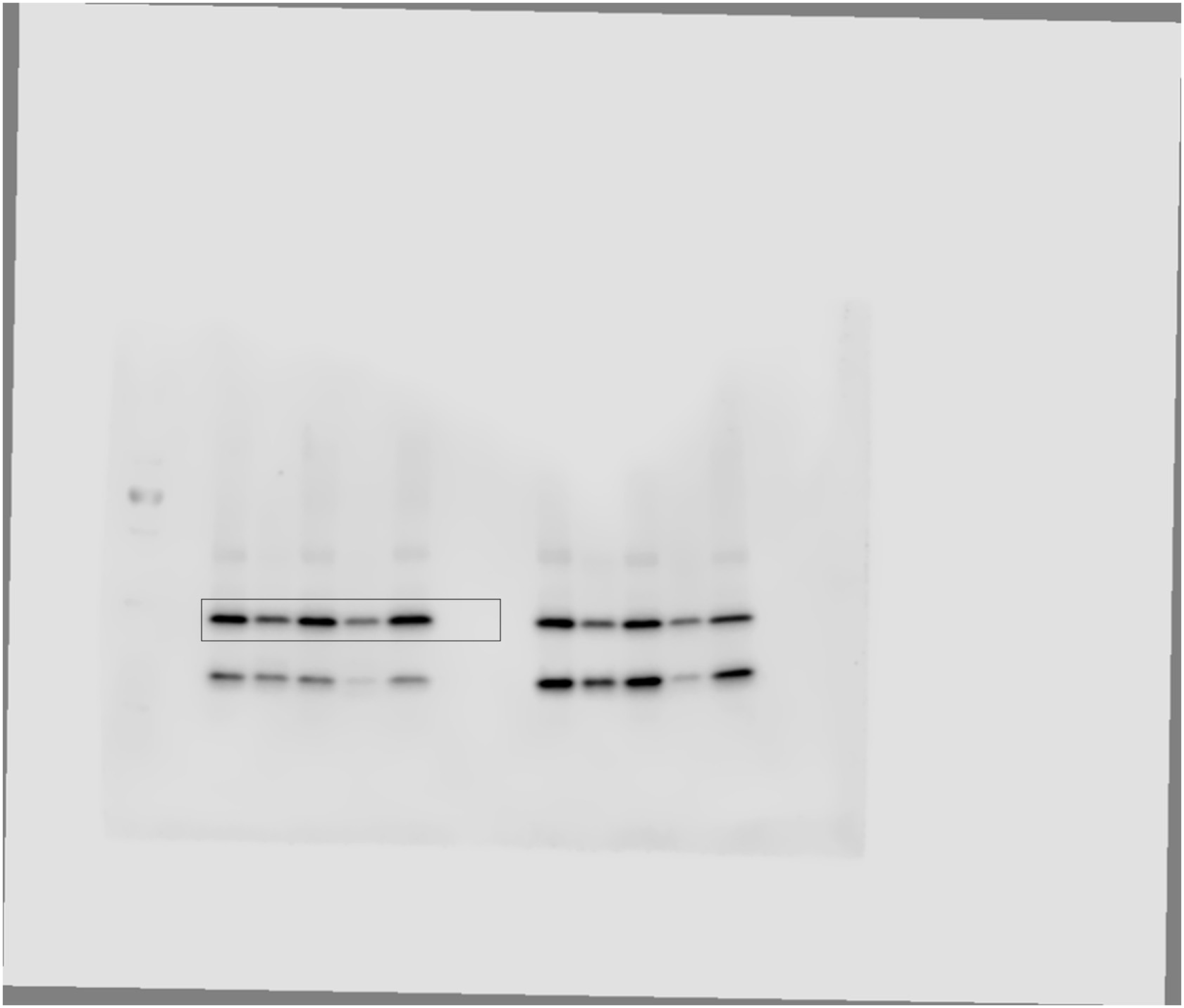

Croppped area for Figure 5D
Ant1(A114P)-HA, anti-HA

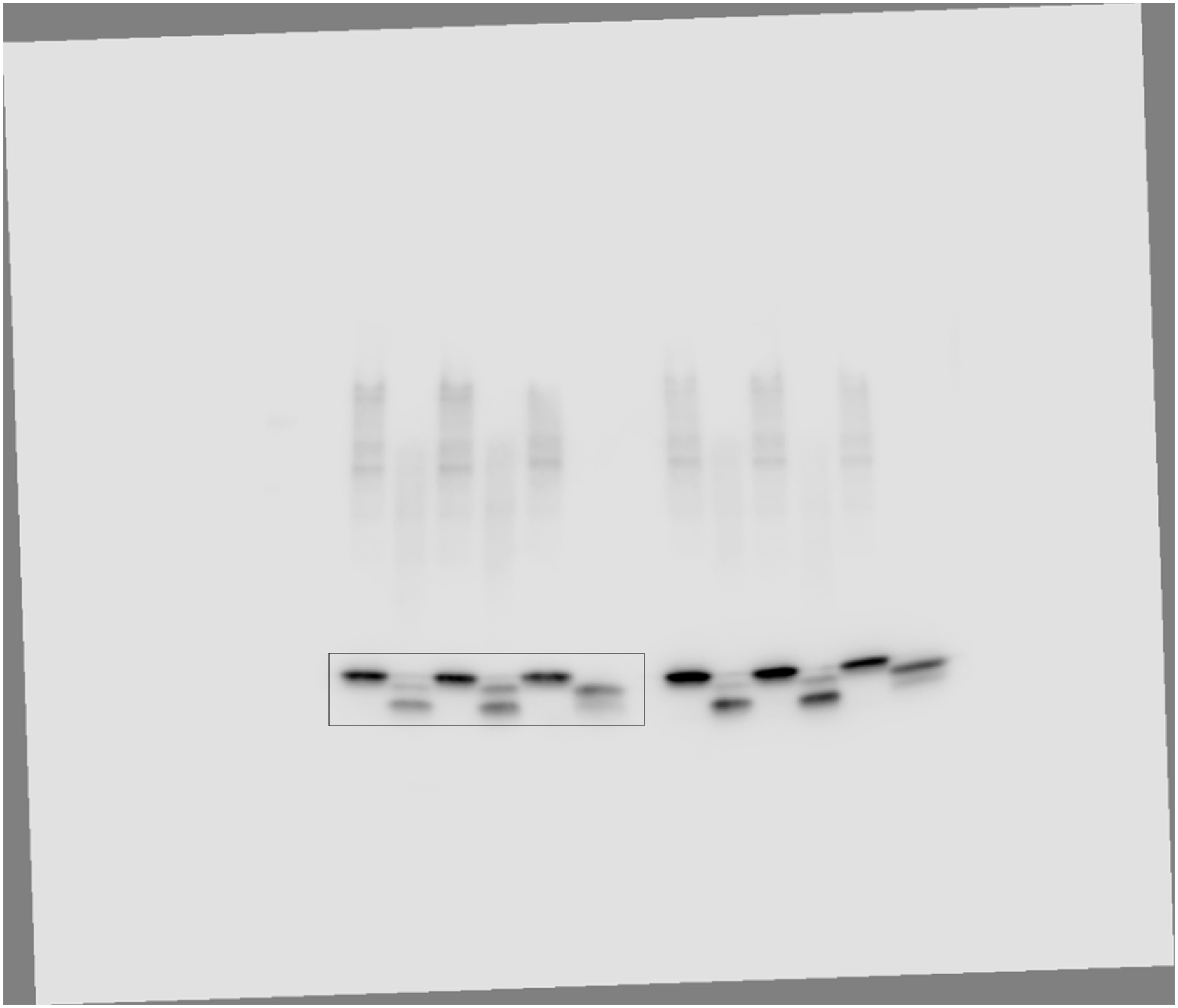

Cropped area for Figure 5D
Ant1(A114P)-HA, anti-Tom20

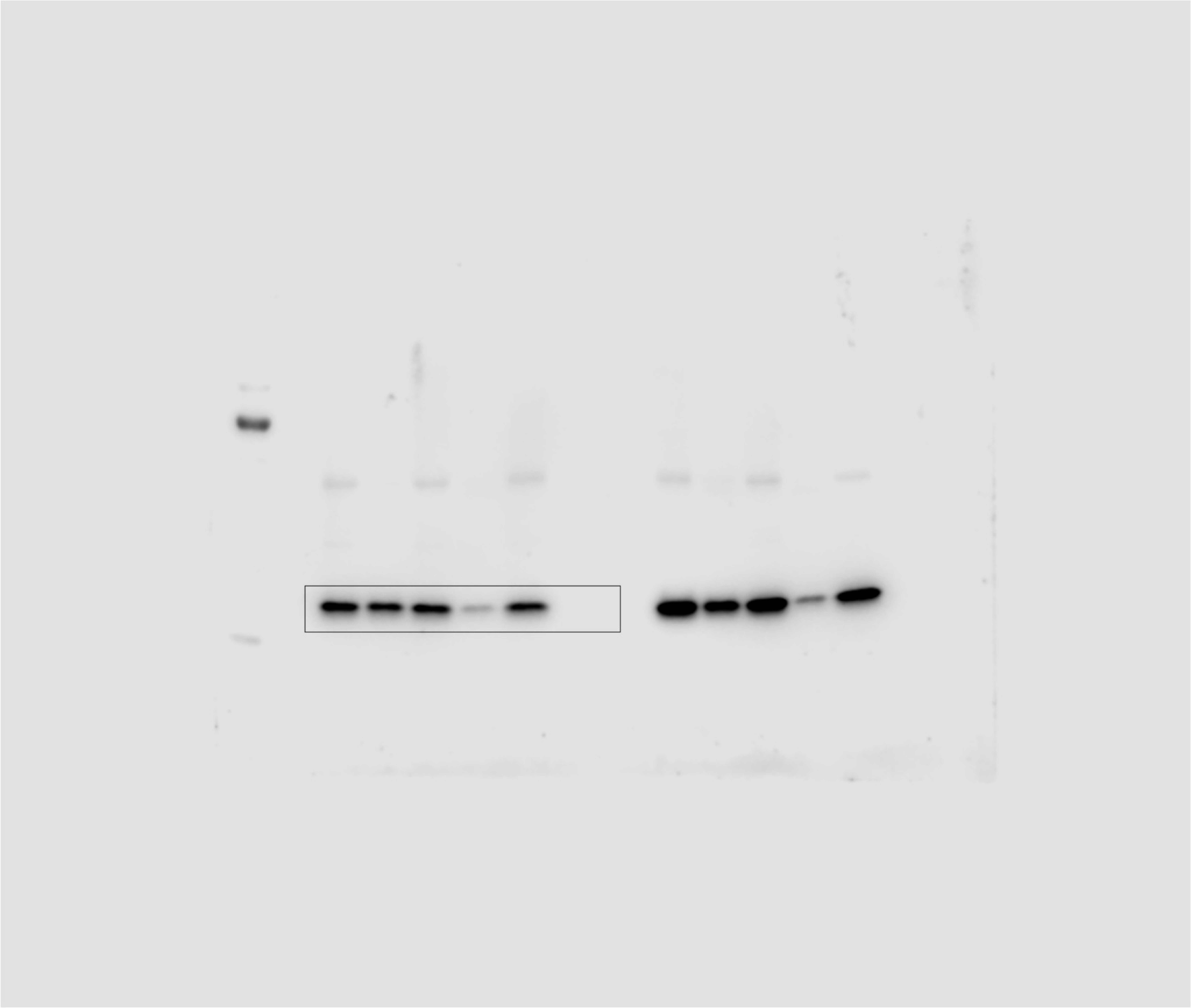

Cropped area for Figure 5D
Ant1(A114P)-HA, anti-Smac

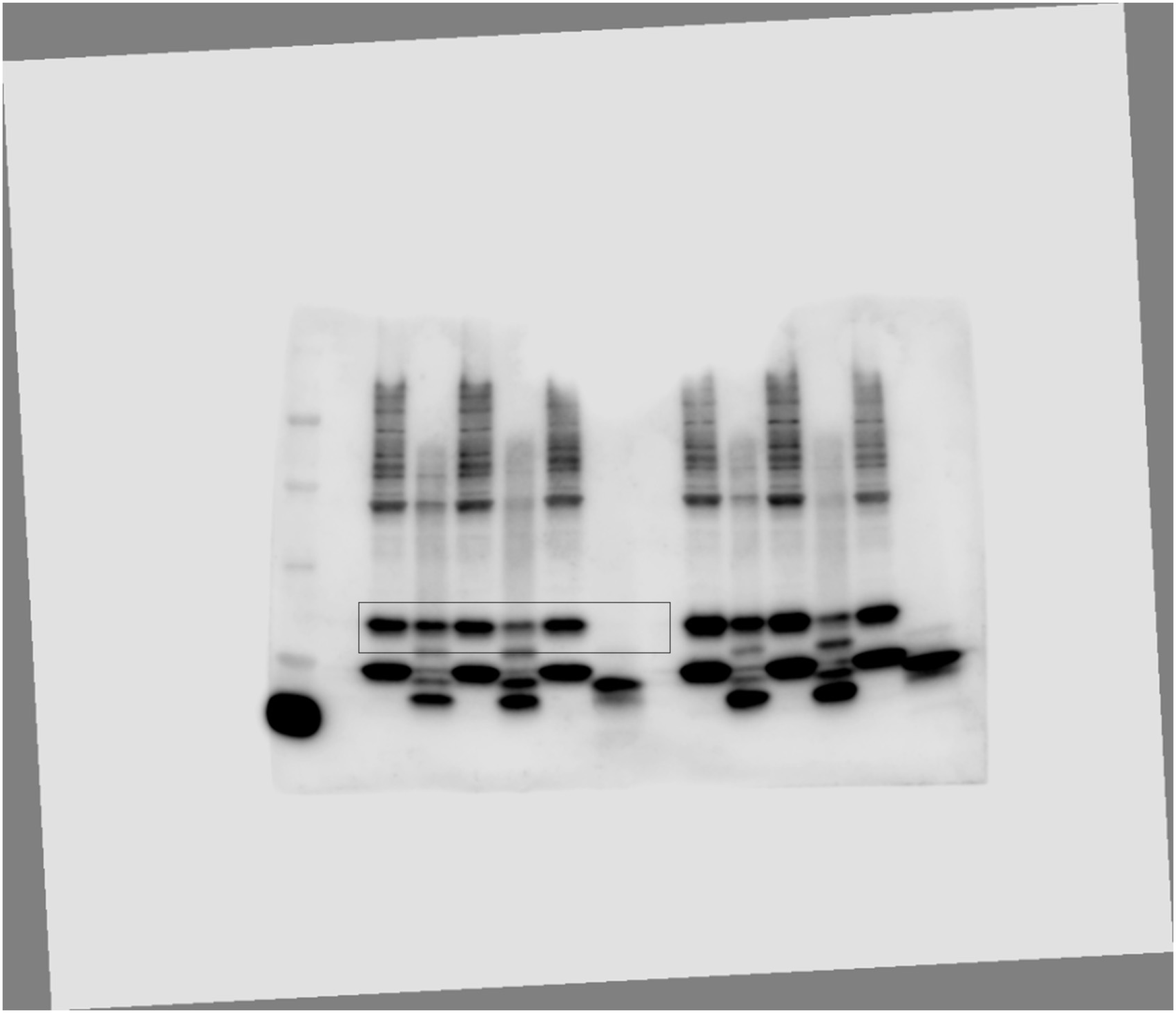

Cropped area for Figure 5D
Ant1(A114P)-HA, anti-Tim22

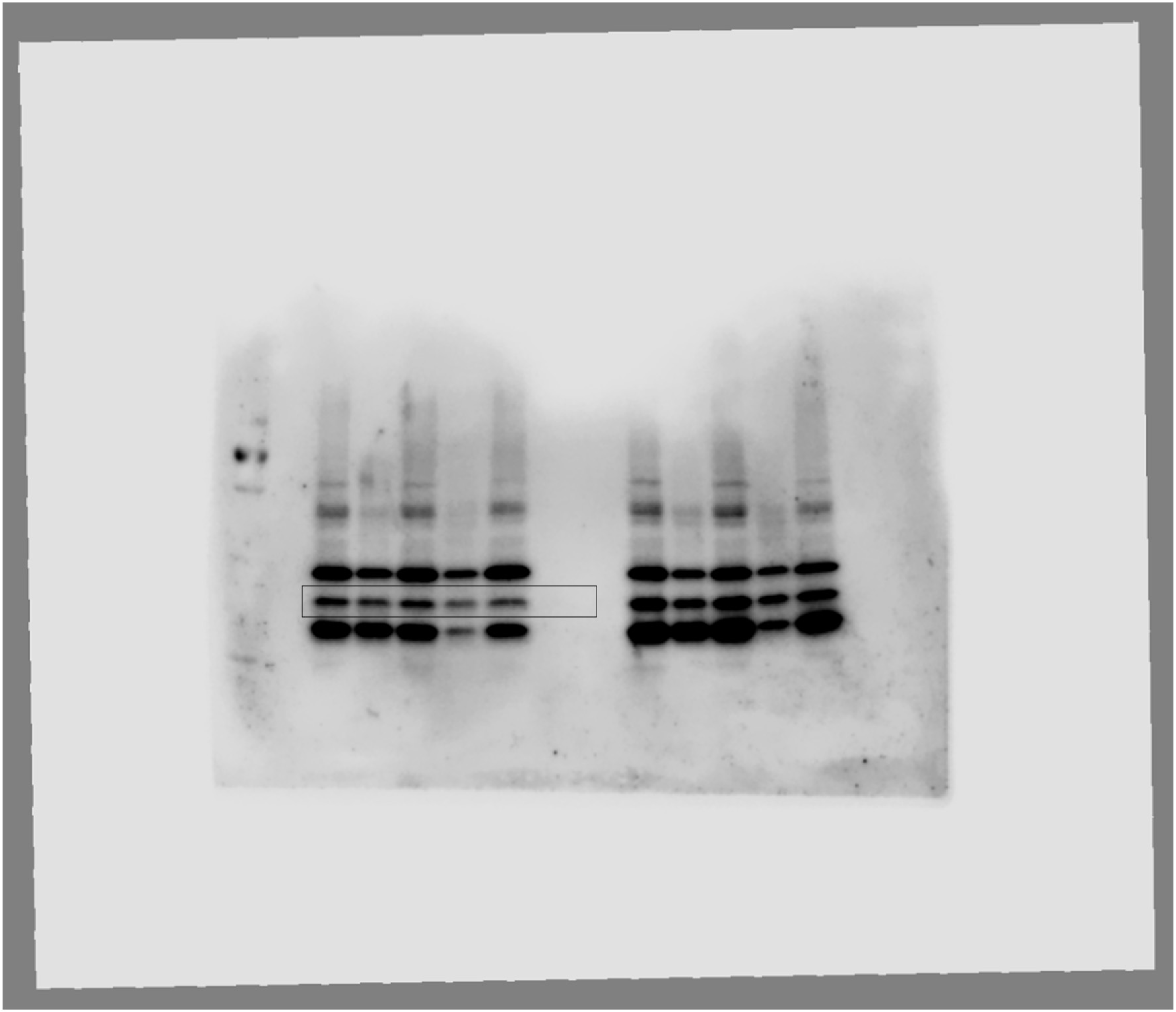

Cropped area for Figure 5D
Ant1(A114P)-HA, anti-TFAM

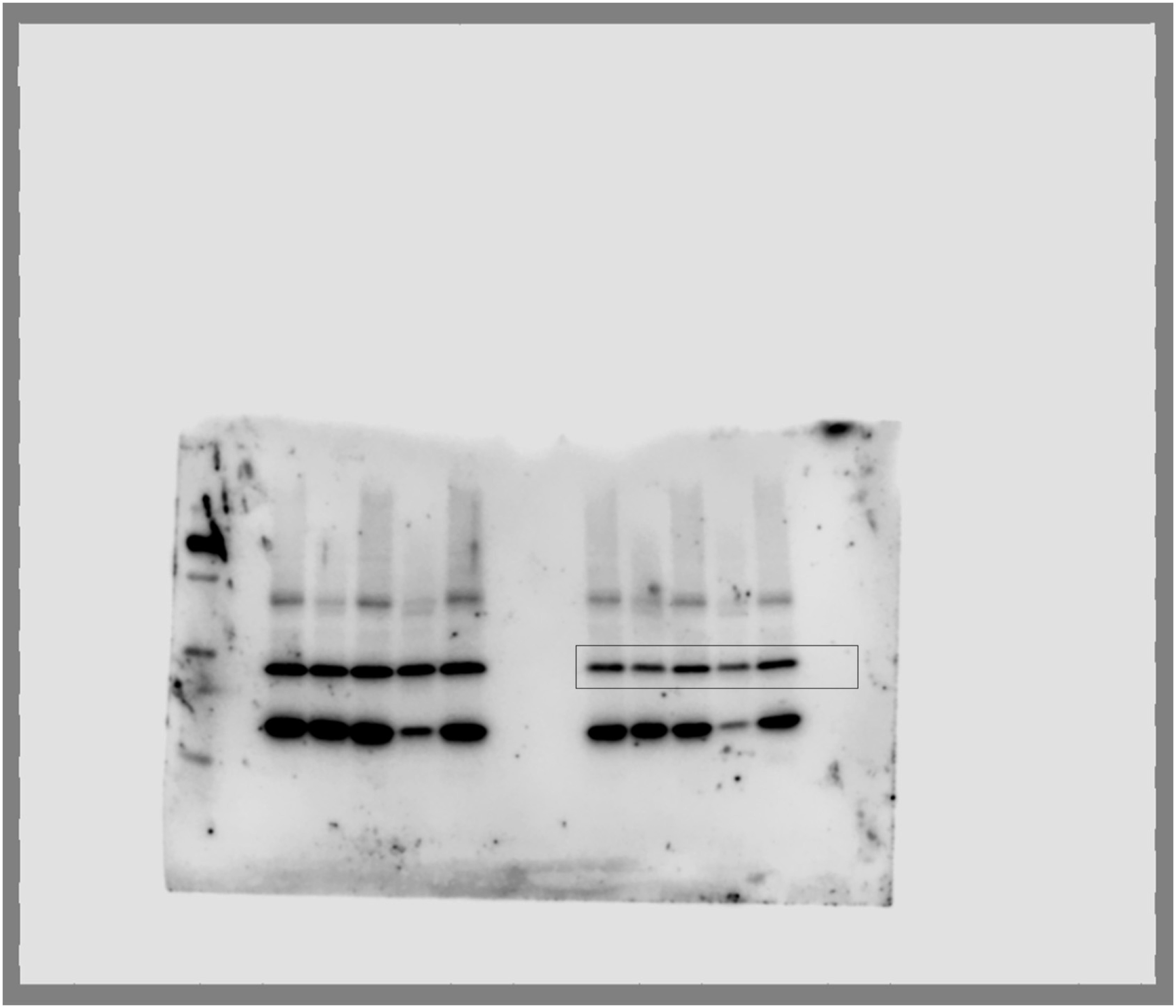

Cropped area for Figure 5D
Ant1(A123D)-HA, anti-HA

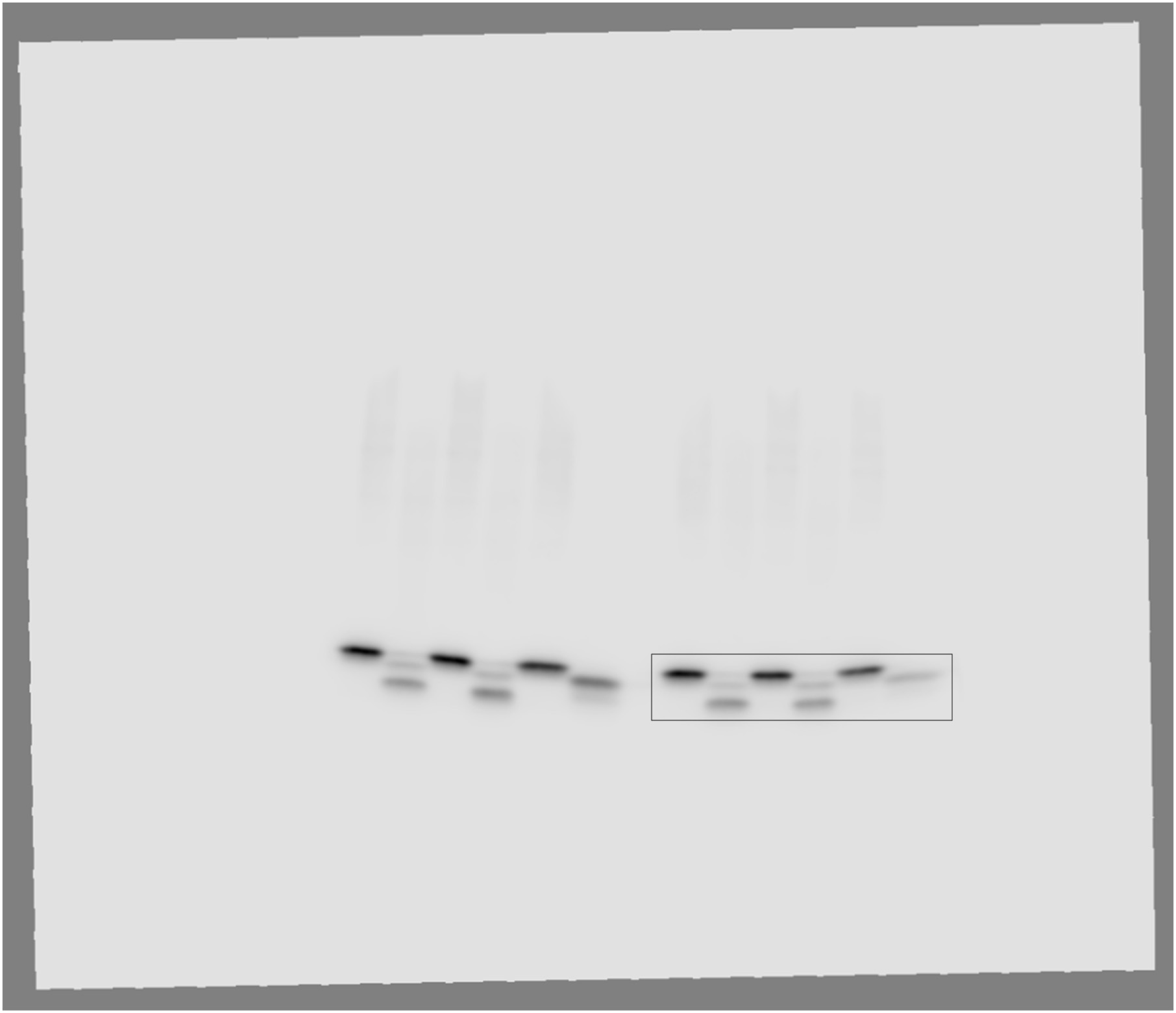

Cropped area for Figure 5D
Ant1(A123D)-HA, anti-Tom20

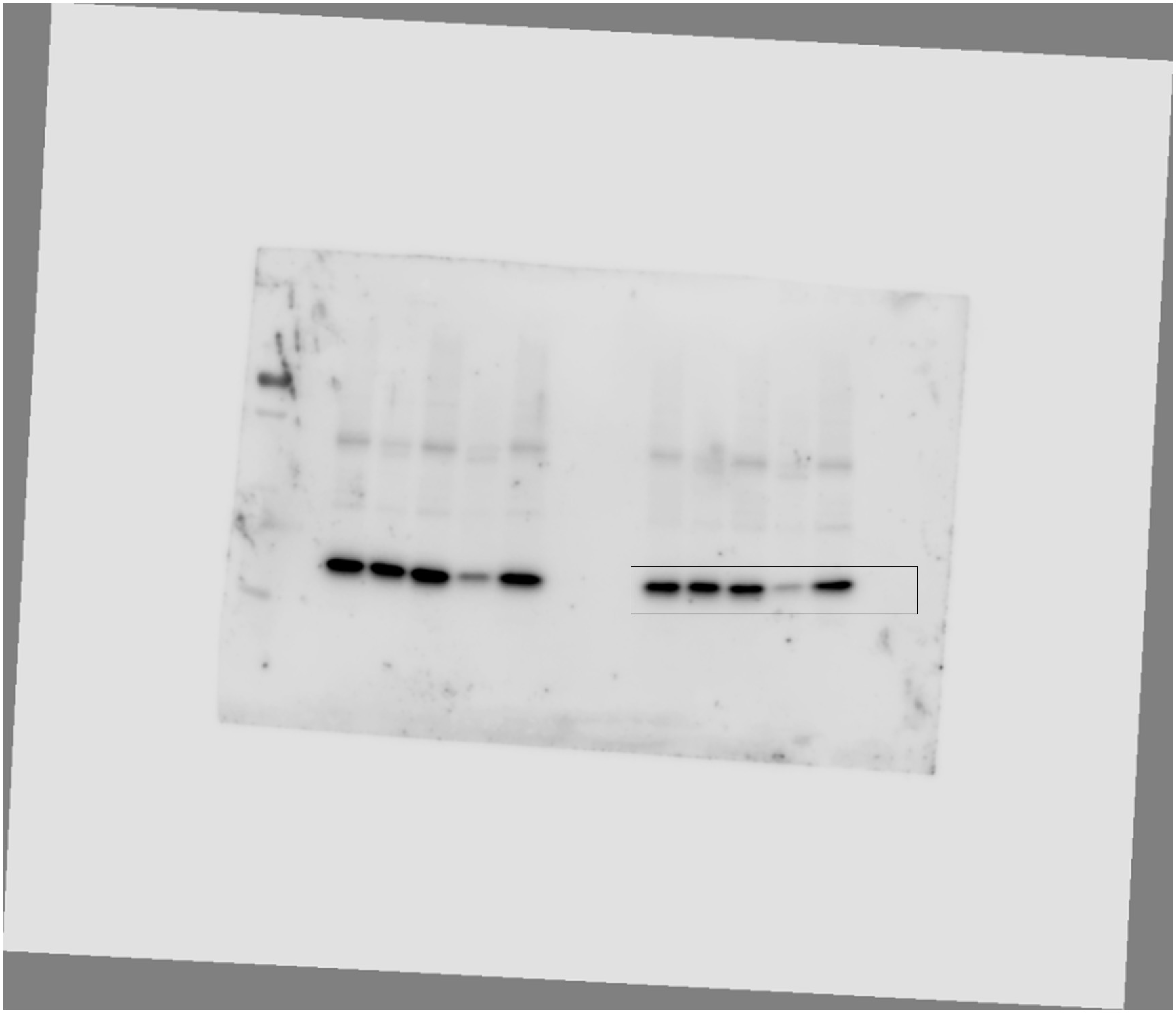

Cropped area for Figure 5D
Ant1(A123D)-HA, anti-Smac

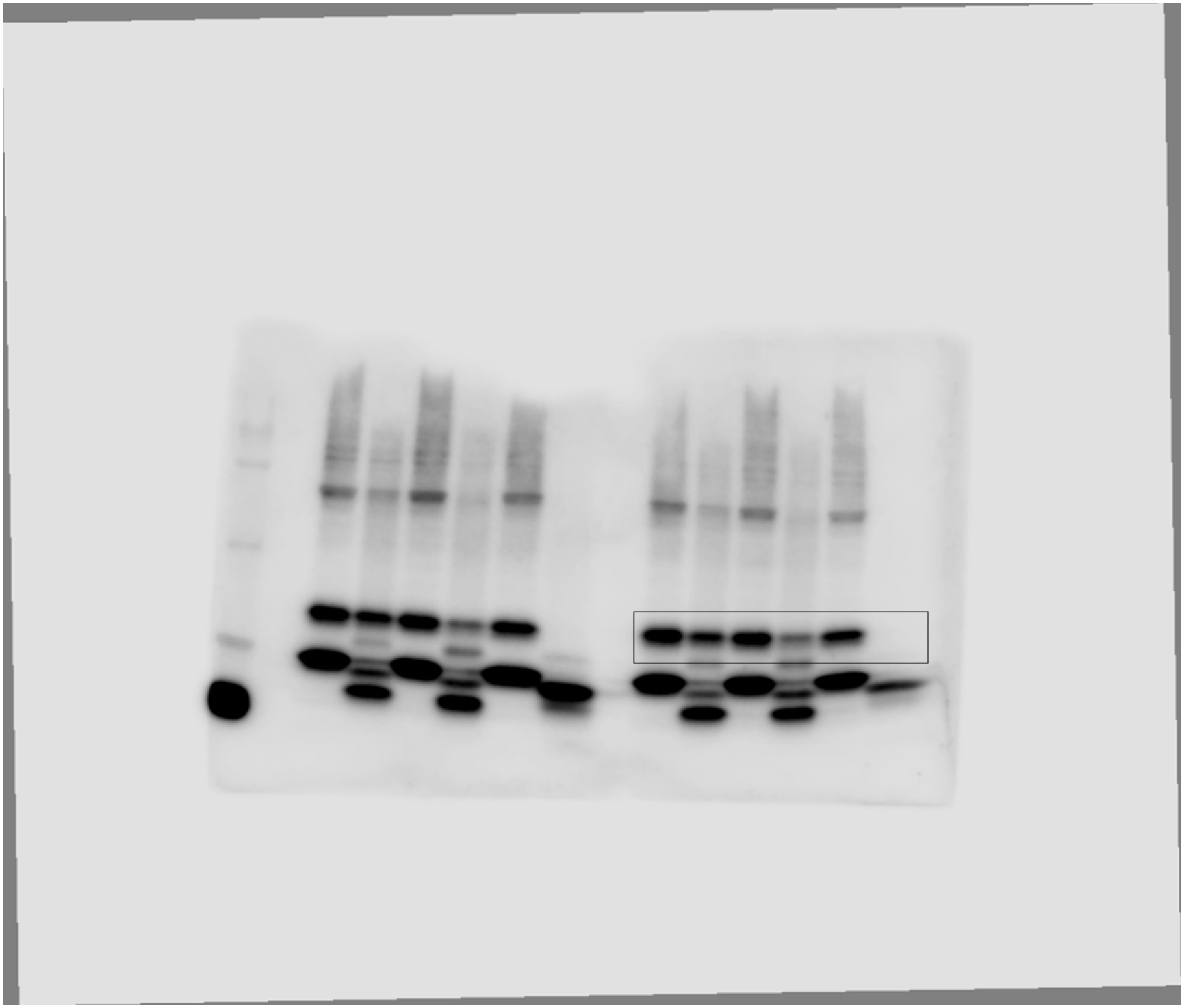

Cropped area for Figure 5D
Ant1(A123D)-HA, anti-Tim22

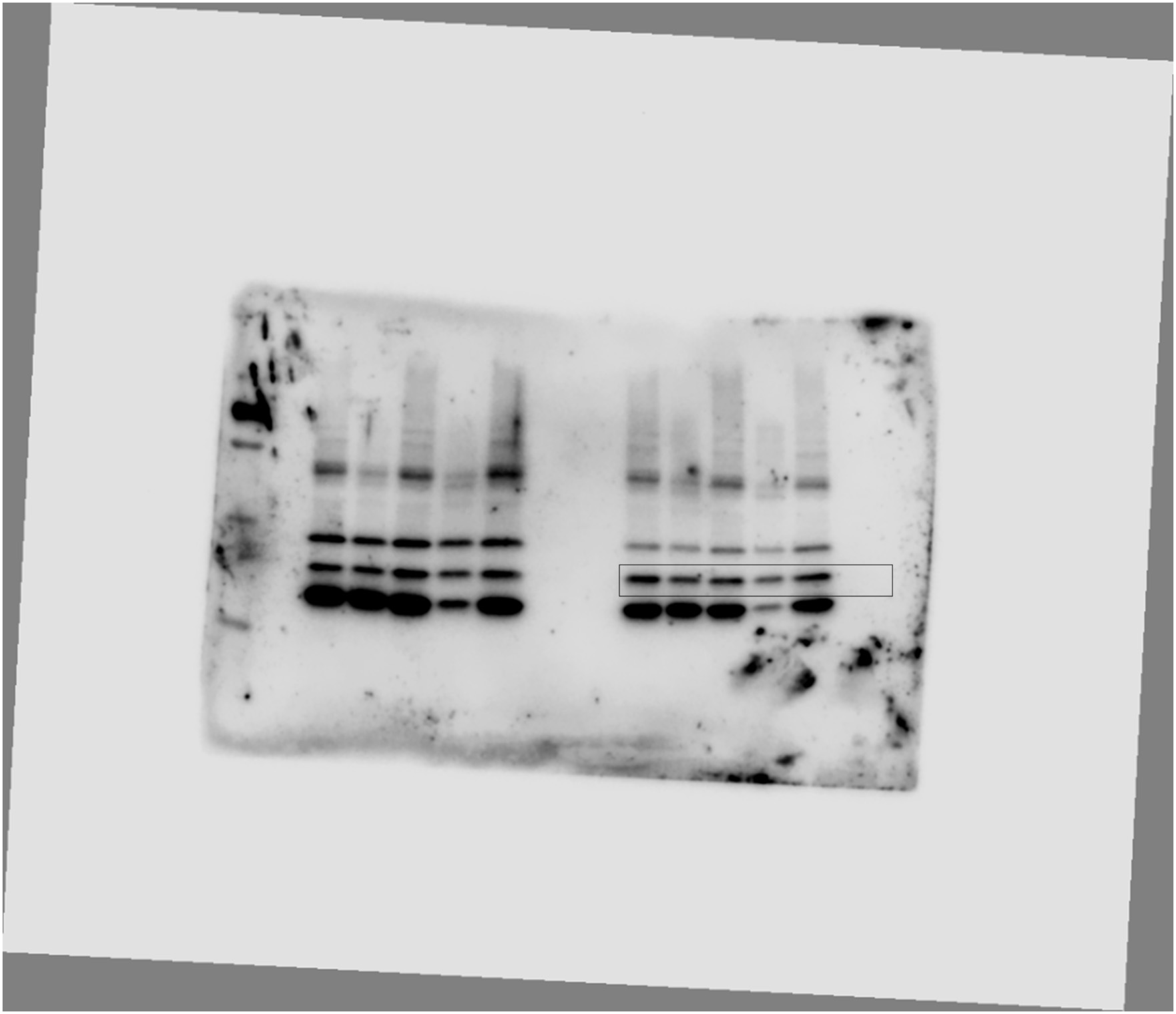

Cropped area for FIgure 5D
Ant1(A123D)-HA, anti-TFAM

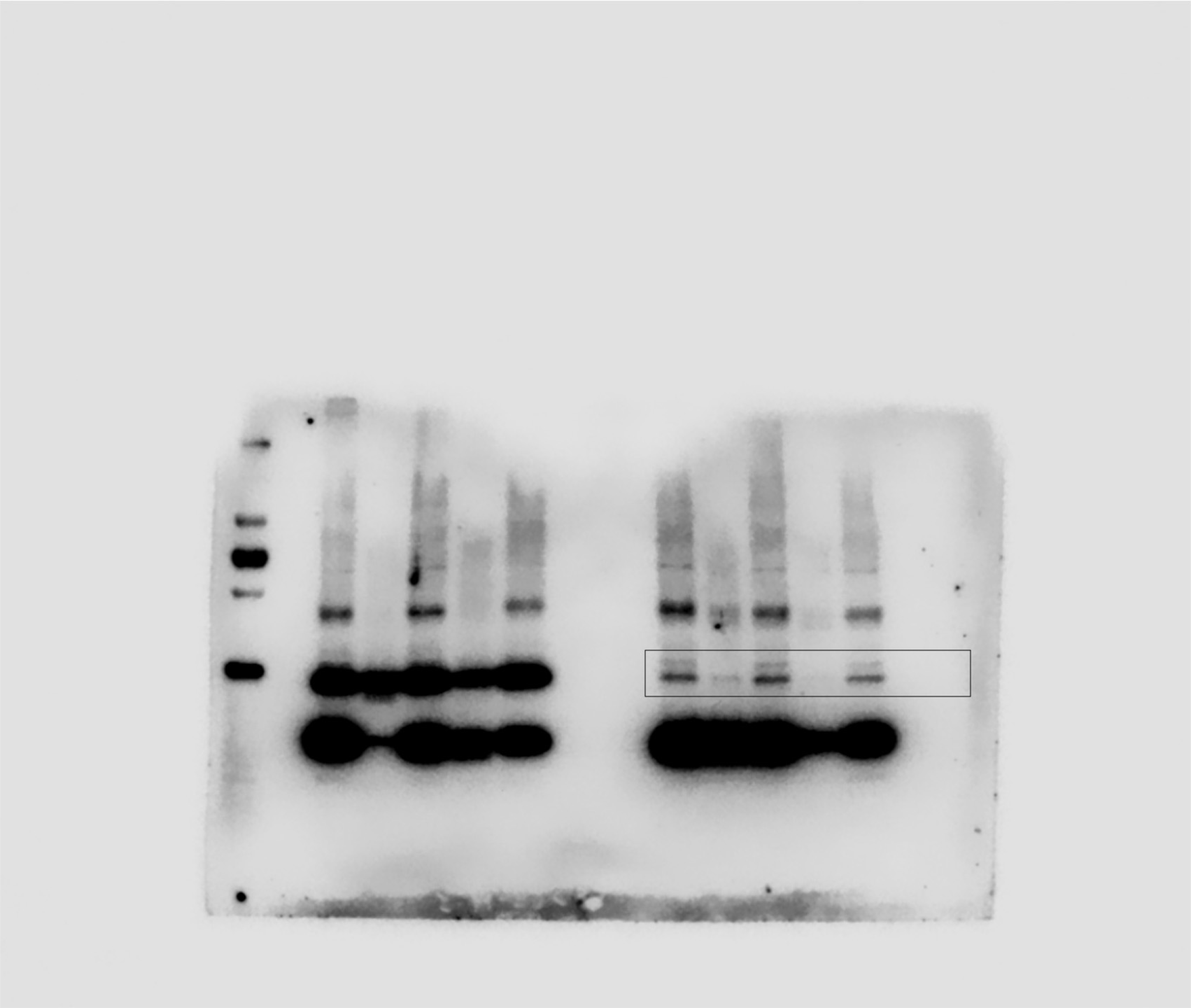

Cropped area for FIgure 5D
Ant1(A114P,A123D)-HA, anti-HA

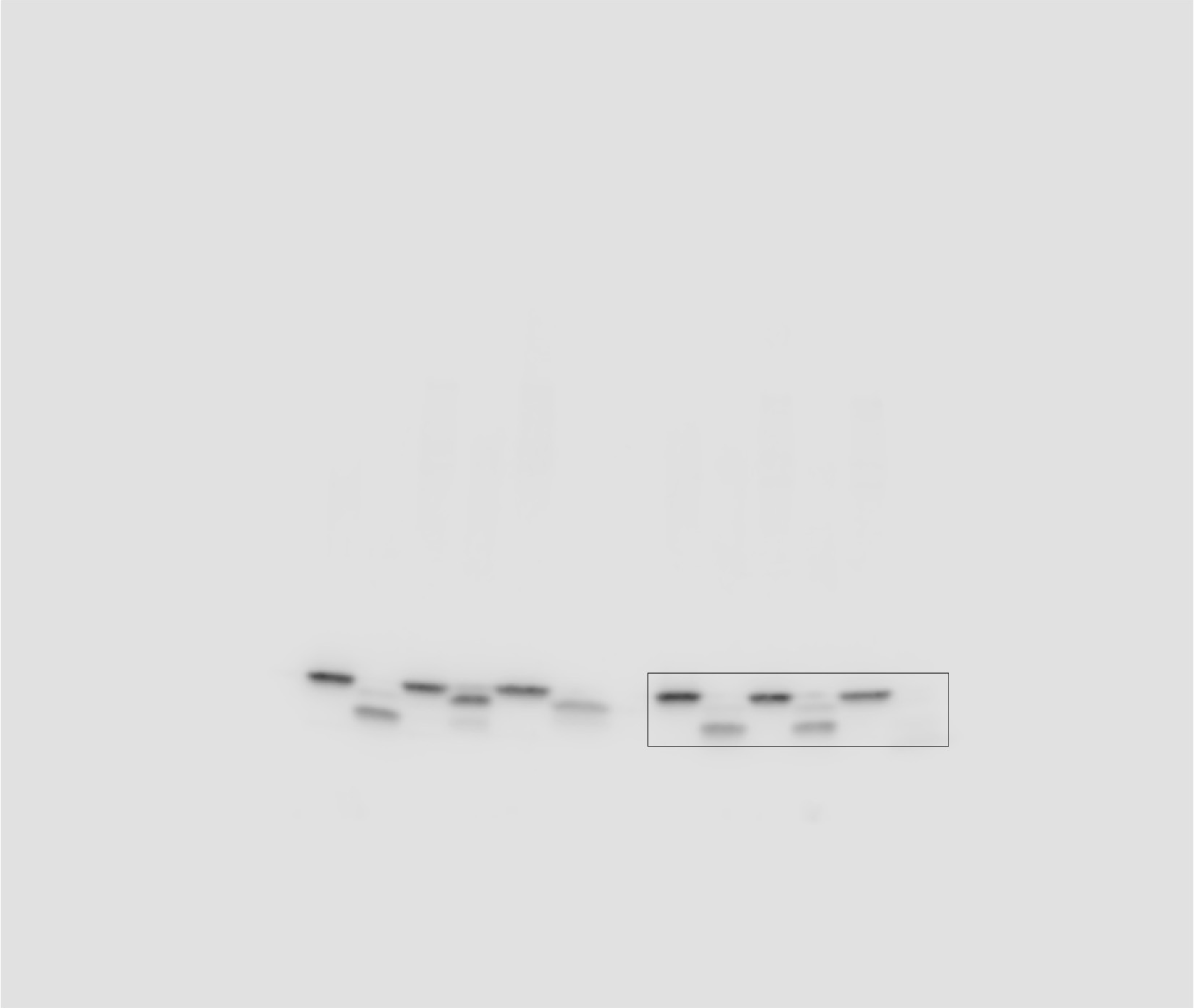

Cropped area for Figure 5D
Ant1(A114P,A123D)-HA, anti-Tom20

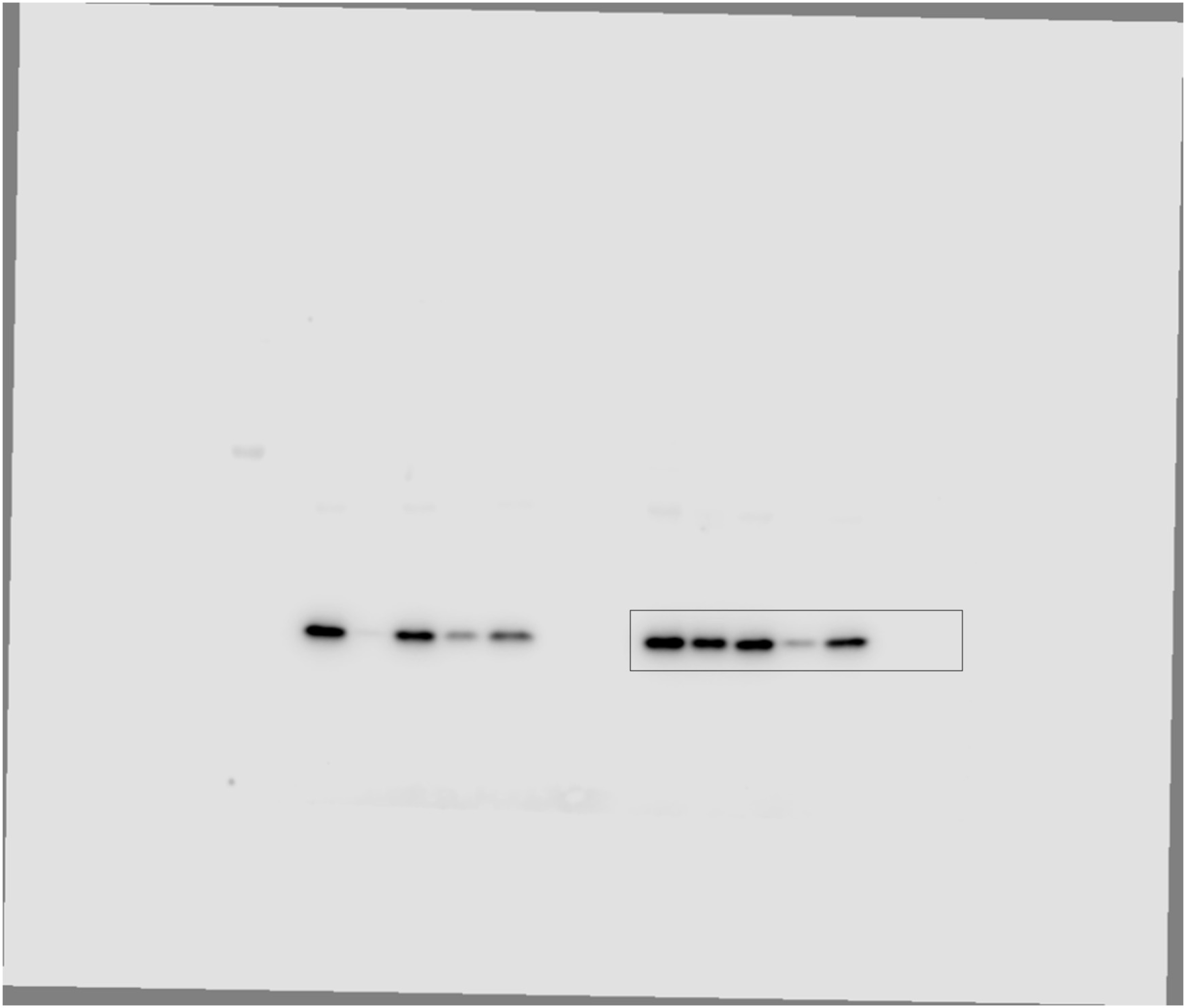

Cropped area for Figure 5D
Ant1(A114P,A123D)-HA, anti-Smac

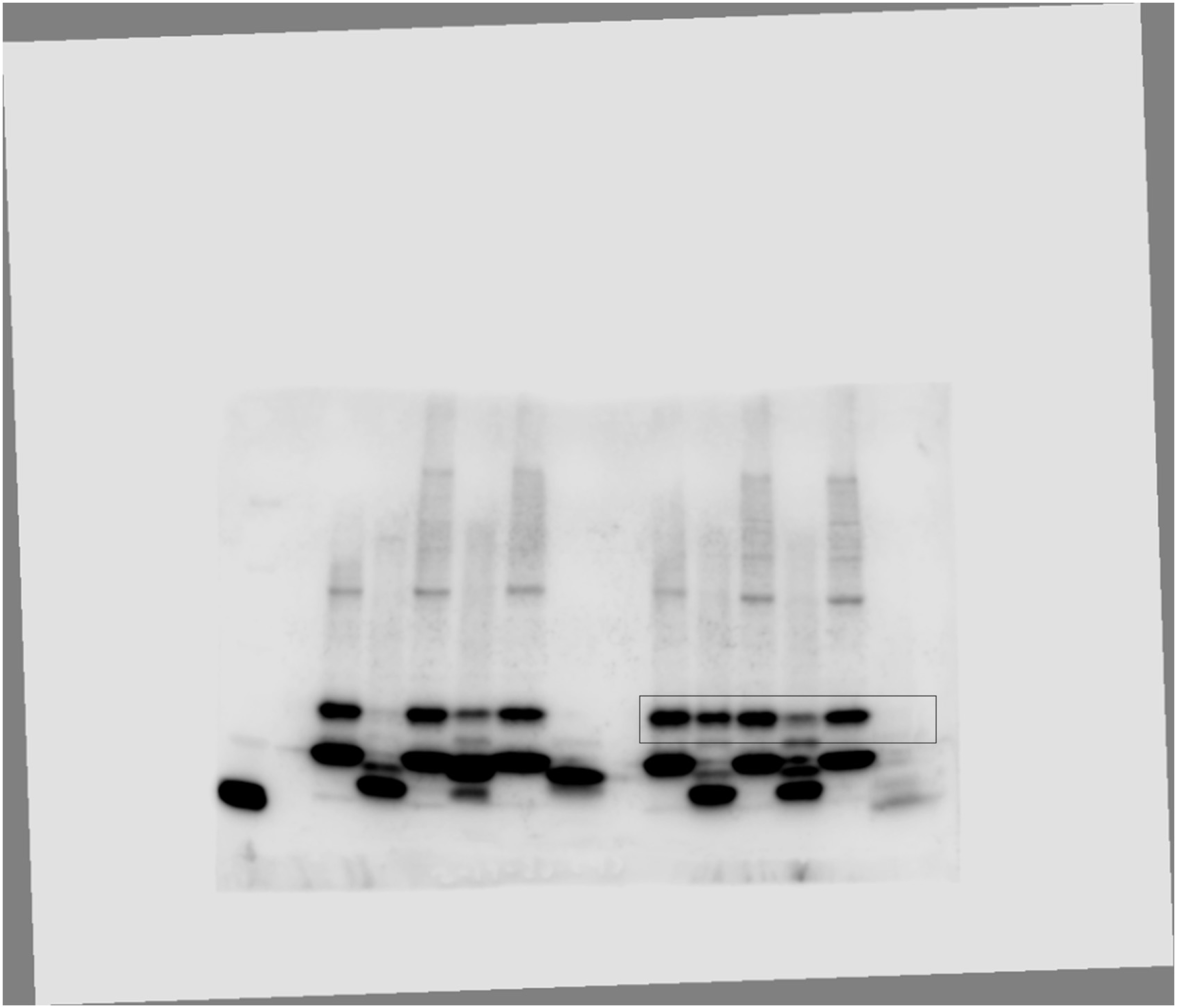

Cropped area for Figure 5D
Ant1(A114P,A123D)-HA, anti-Tim22

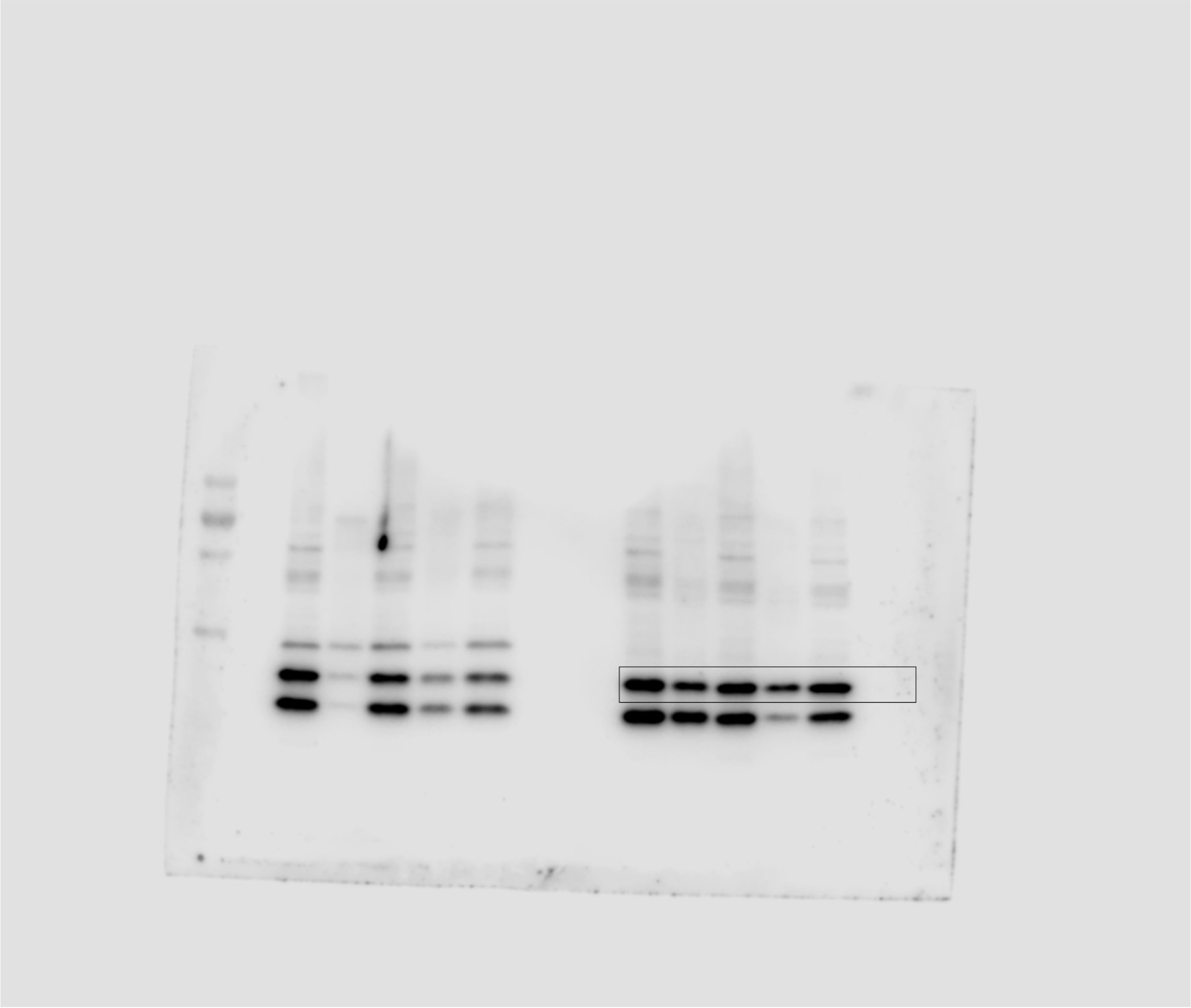

Cropped area for Figure 5D
Ant1(A114P,A123D)-HA, anti-TFAM
