# Supplementary material for: Mitochondrial protein import clogging as a mechanism of disease
Source: eLife. 2023 May 2;12:e84330. doi: 10.7554/eLife.84330 (PMC10208645; doi:10.7554/eLife.84330)
Supplement: Figure 8—source data 2. [file elife-84330-fig8-data2.zip › Figure 8-source data 1/Figure 8-source data_annotated.pdf]

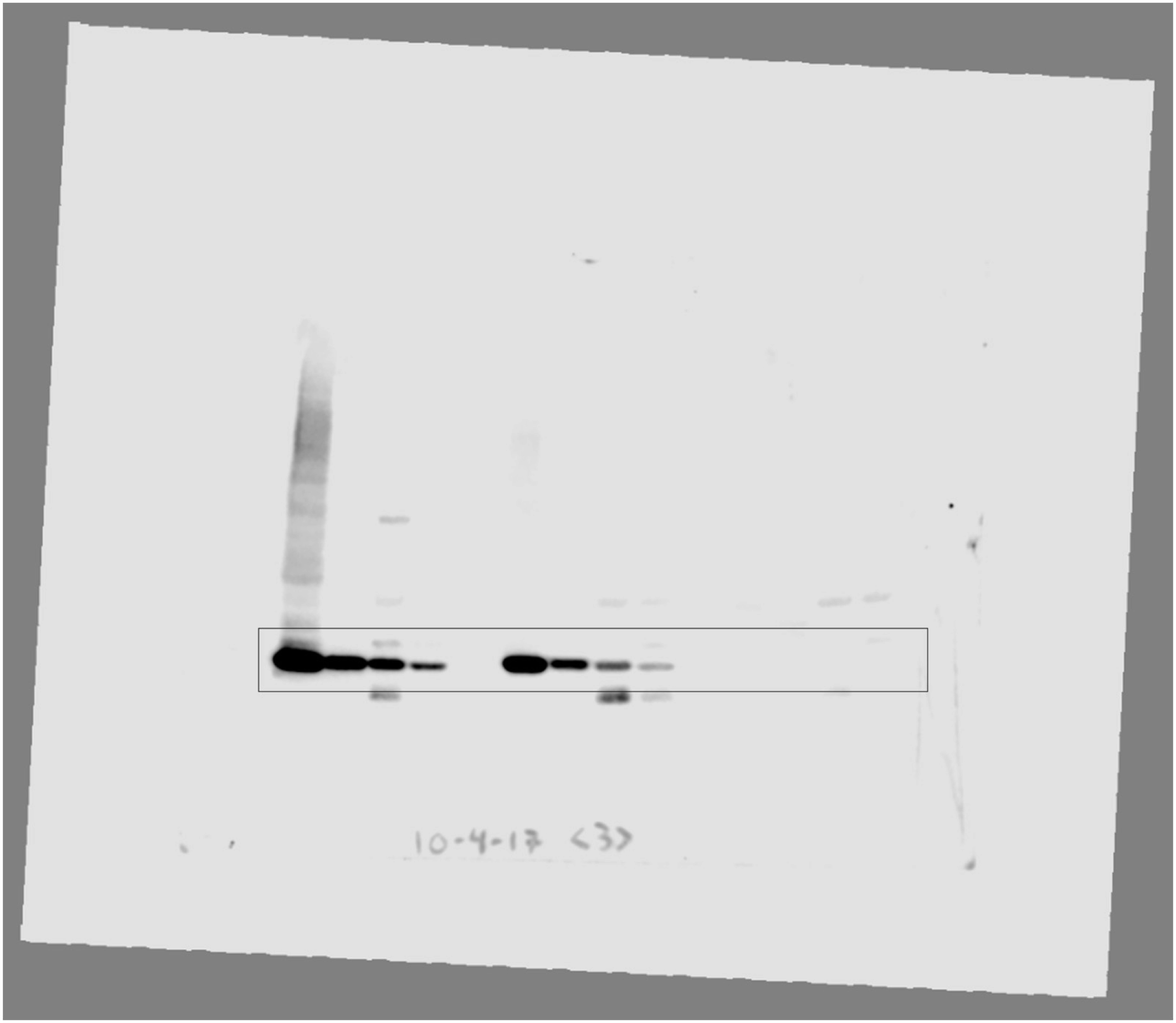

Cropped area for Figure 8A
Ant1

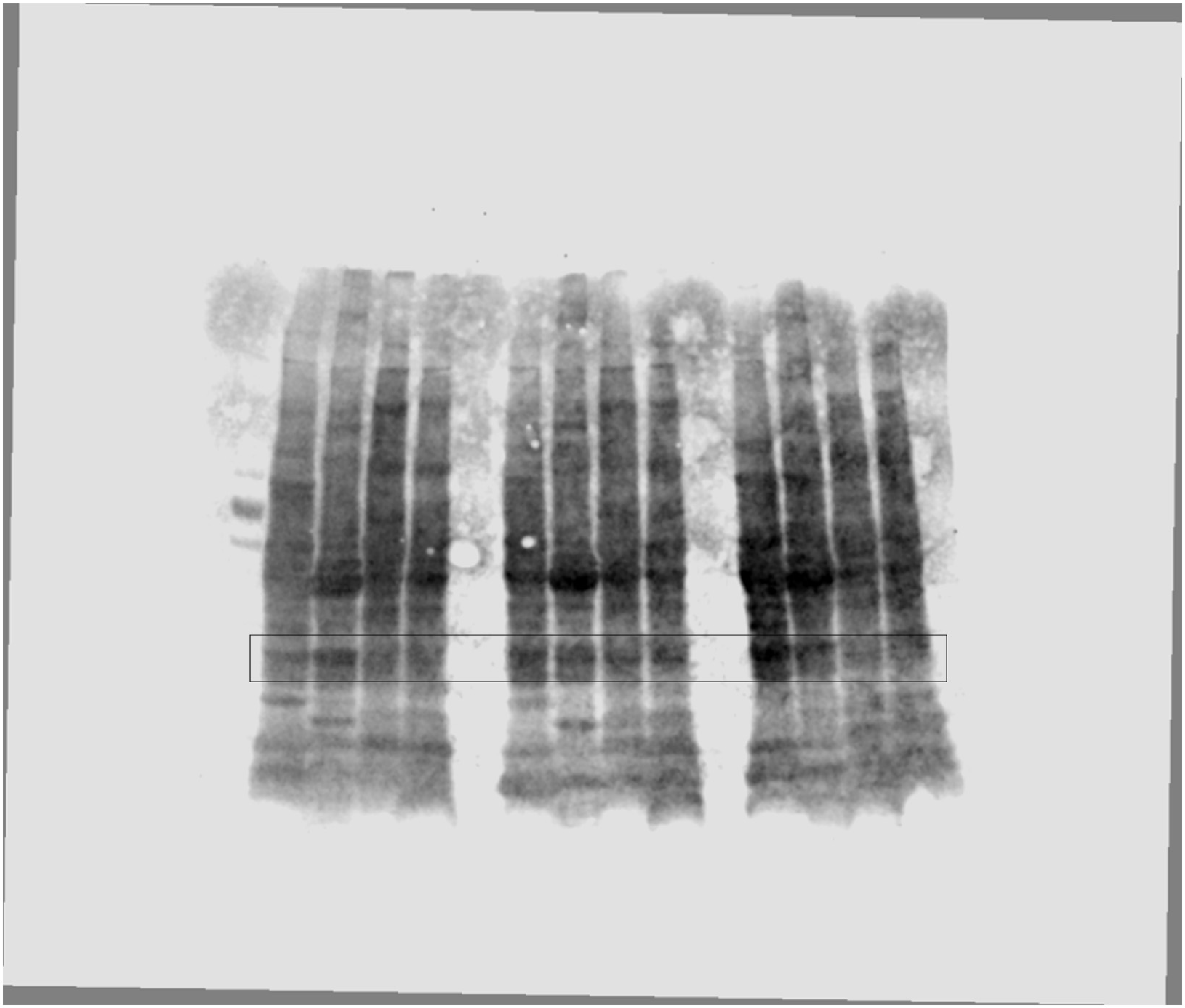

Cropped area for Figure 8A
Total Protein Stain

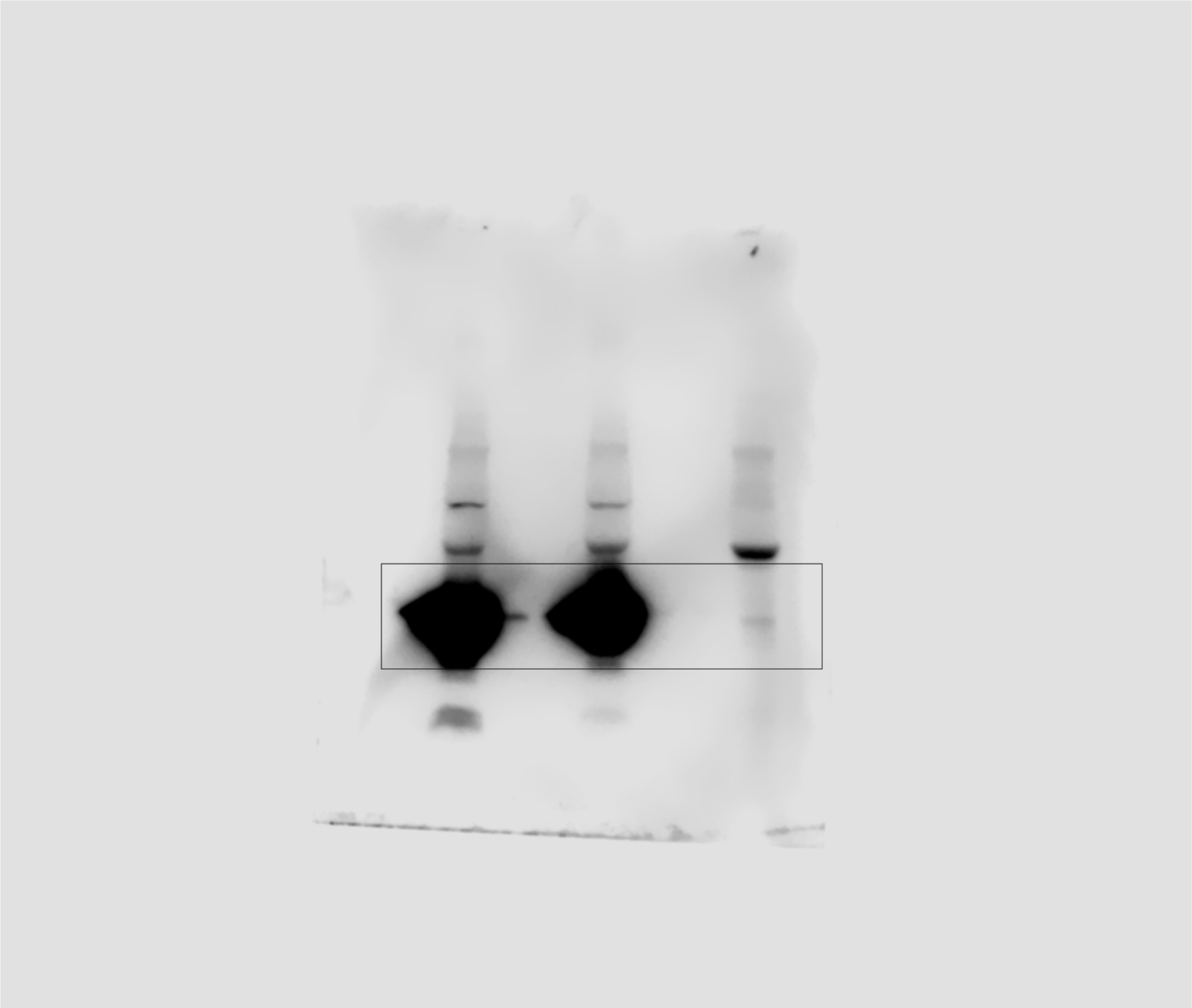

Cropped area for Figure 8B
Ant1 long

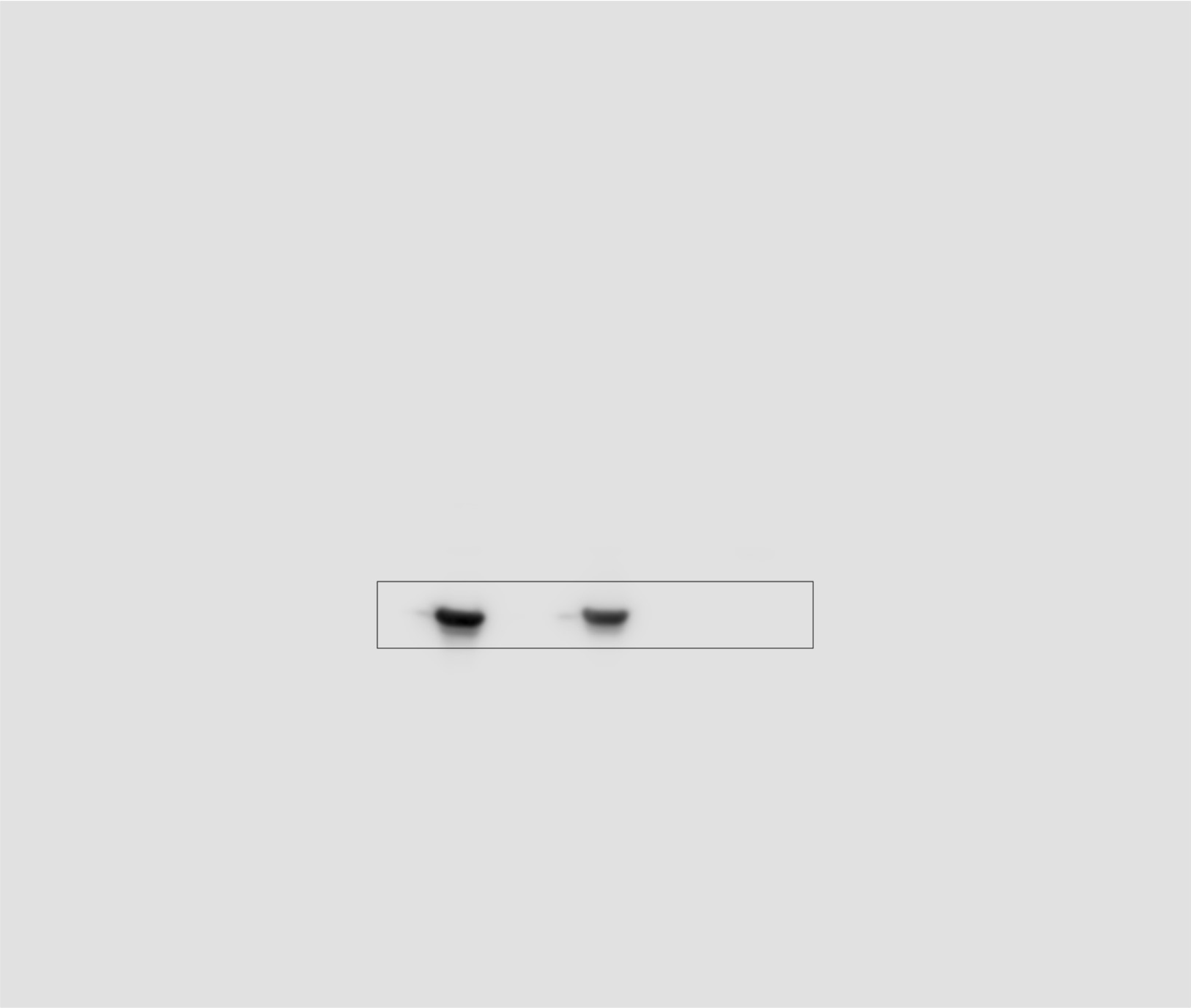

Cropped area for Figure 8B
Ant1 short

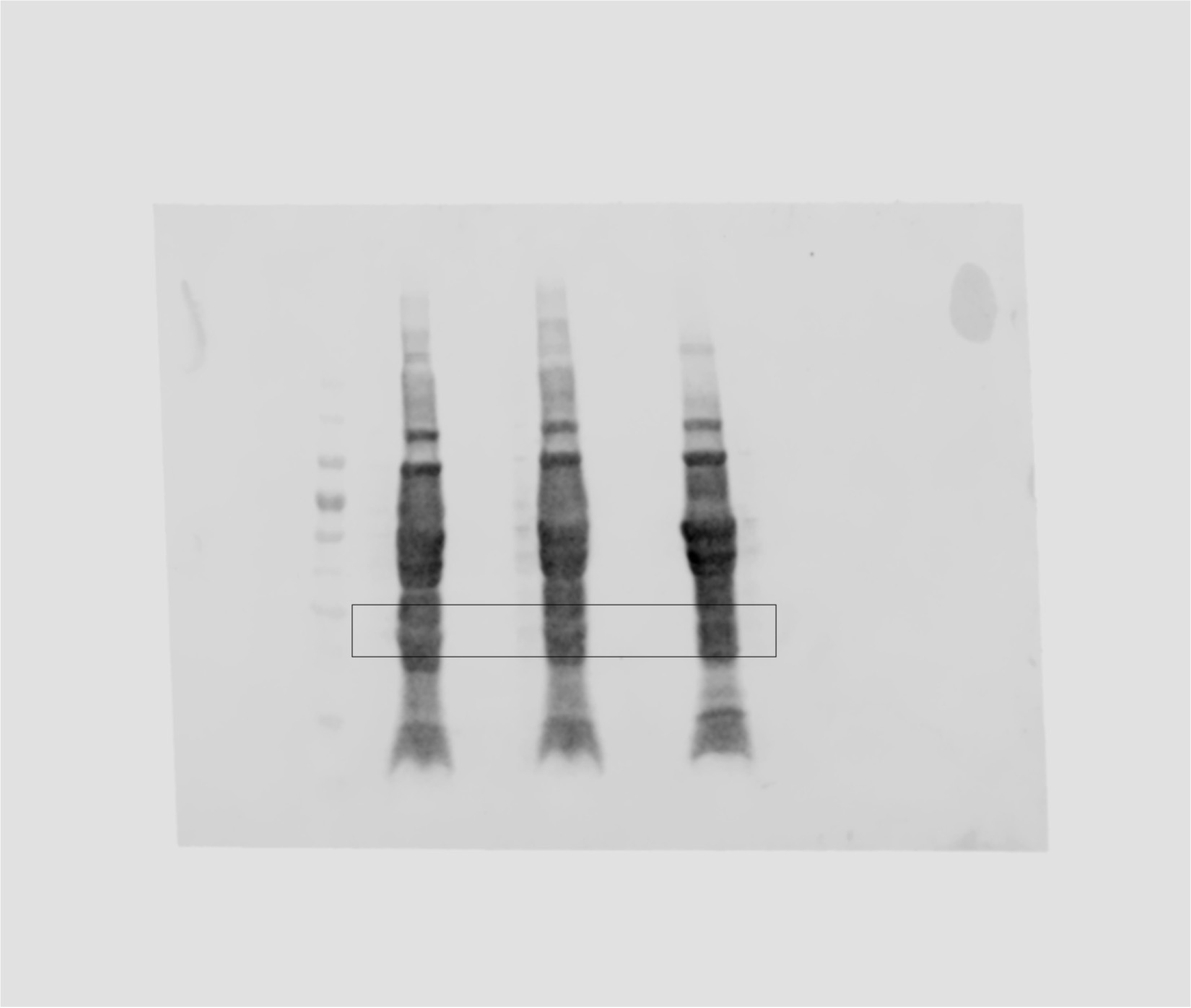

Cropped area for Figure 8B
Total Protein Stain

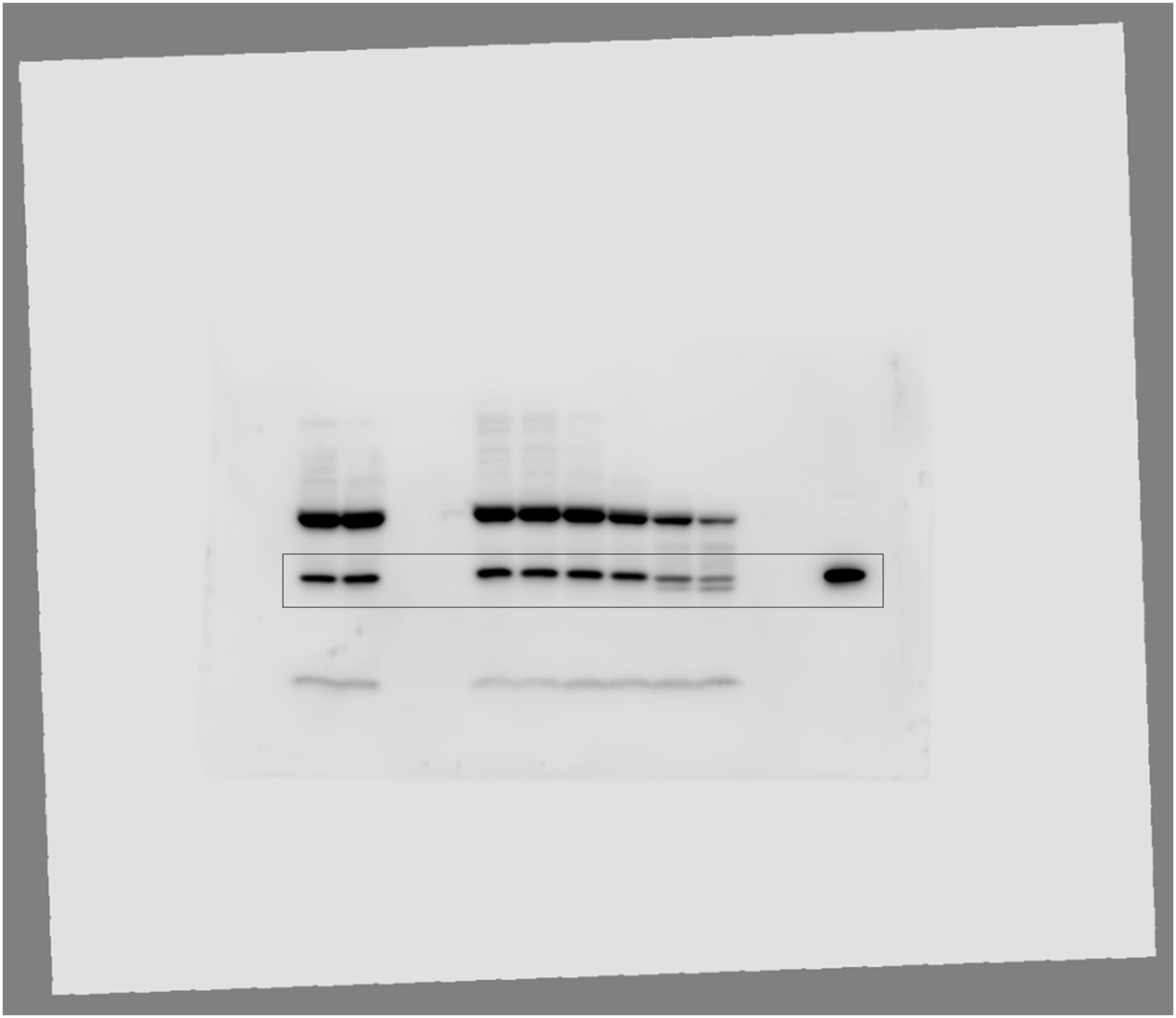

Cropped area for Figure 8D
$Ant1^{A114P,A123D}/Ant1^{A114P,A123D}$ mito, Ant1

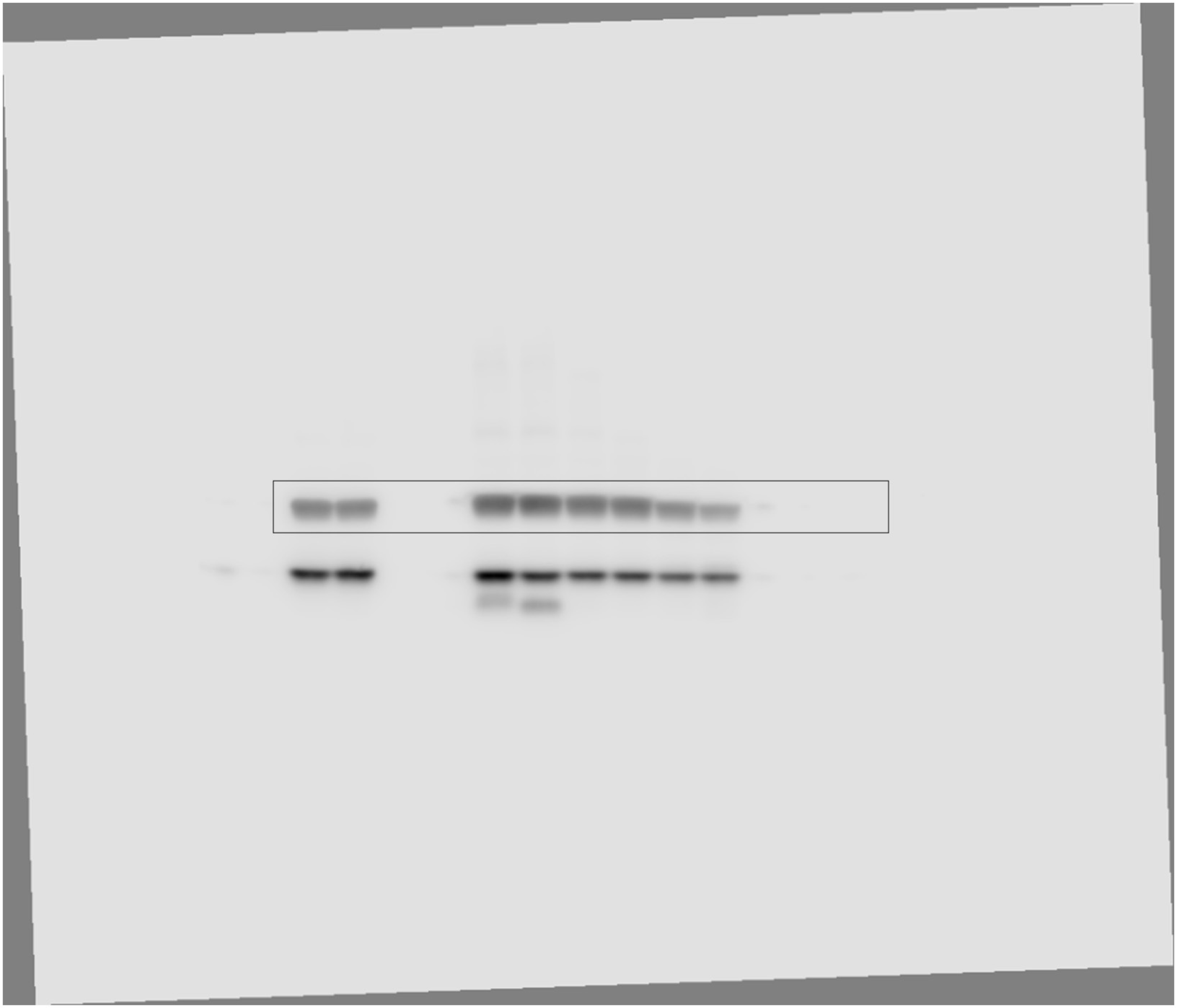

Cropped area for Figure 8D
Ant1^{A114P,A123D}/Ant1^{A114P,A123D}, Mdh2

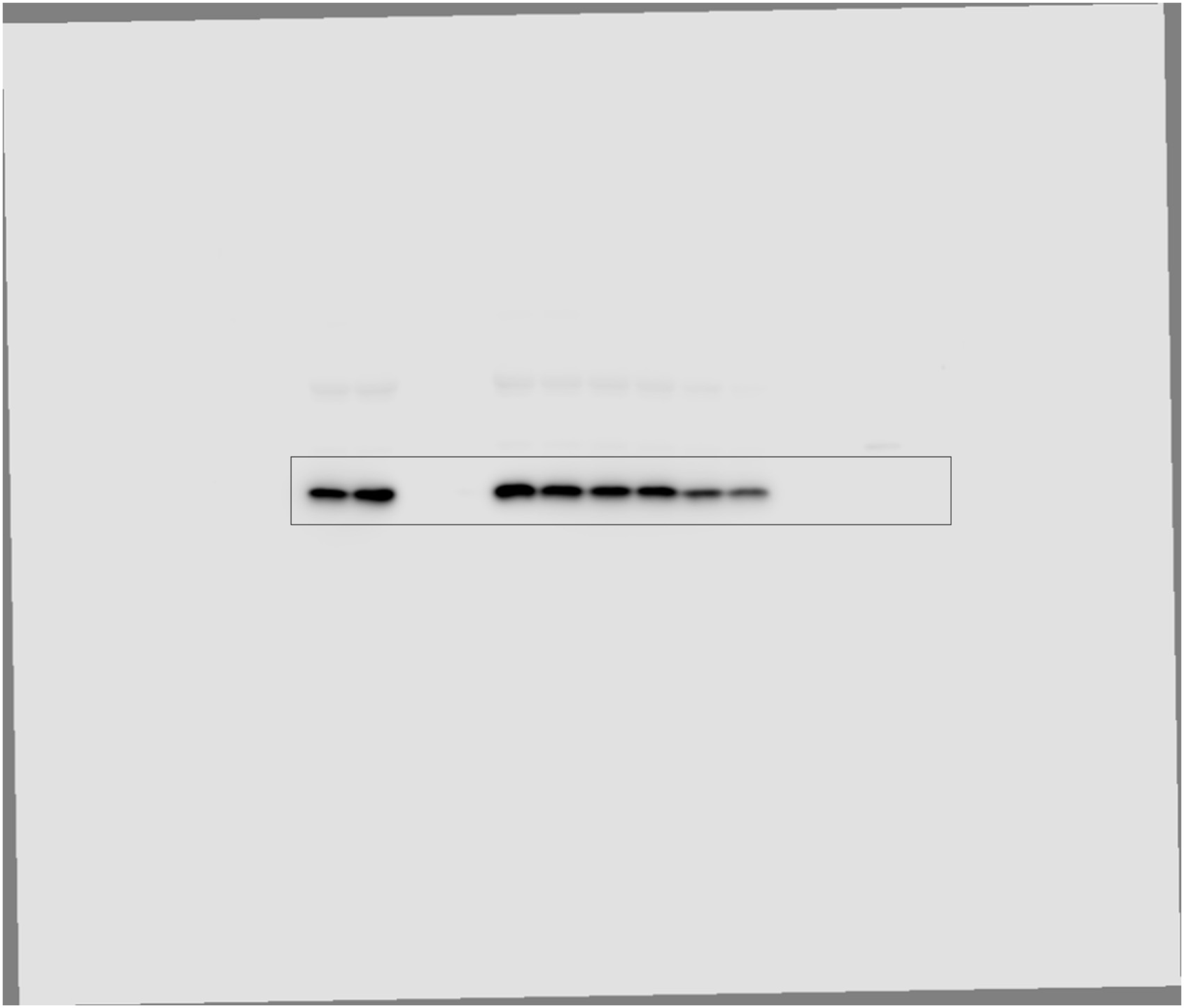

Cropped area for Figure 8D

$Ant1^{A114P,A123D}/Ant1^{A114P,A123D}$ mito, SMAC

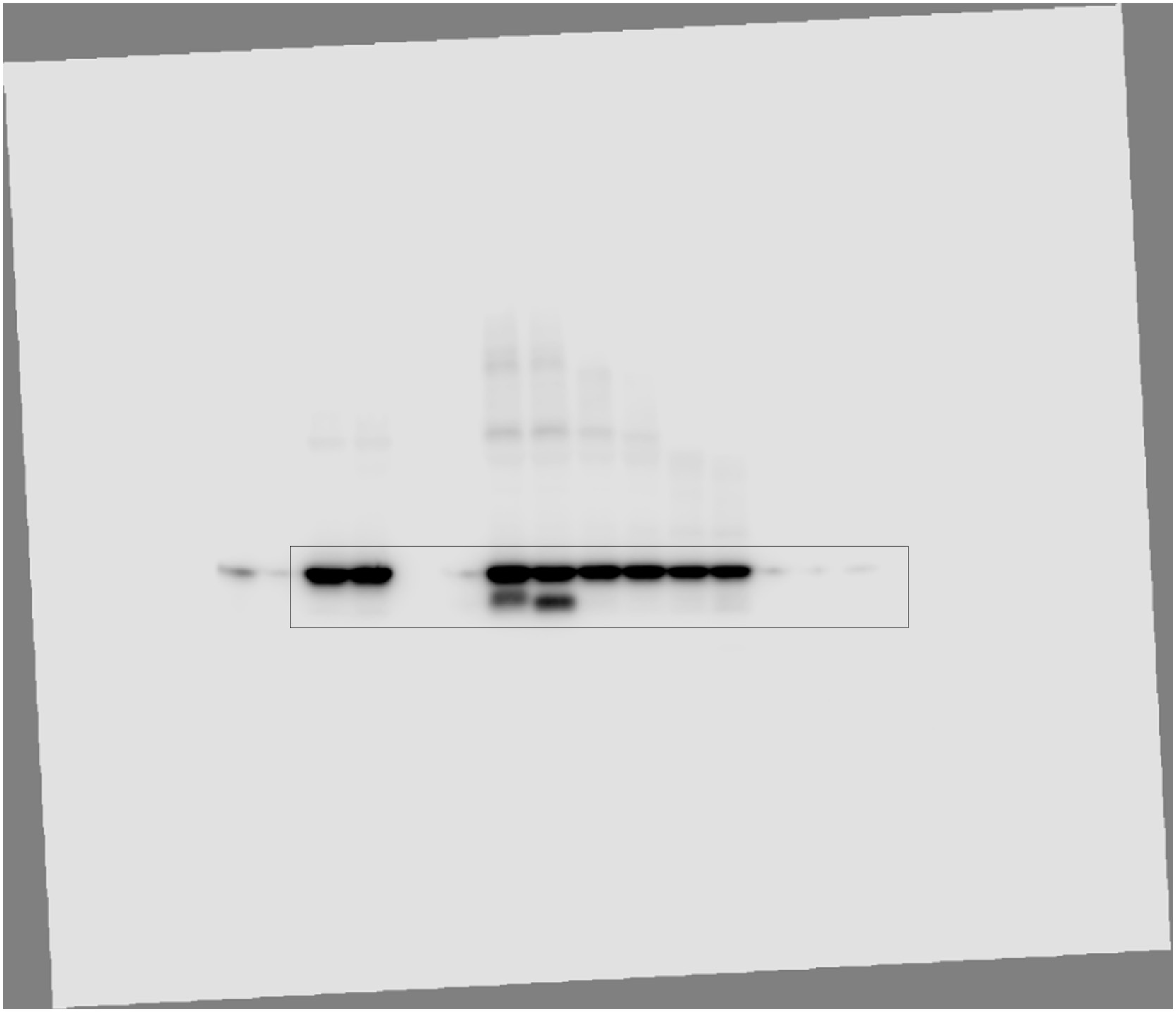

Cropped area for Figure 8D

$Ant1^{A114P,A123D}/Ant1^{A114P,A123D}$ mito, TIM23

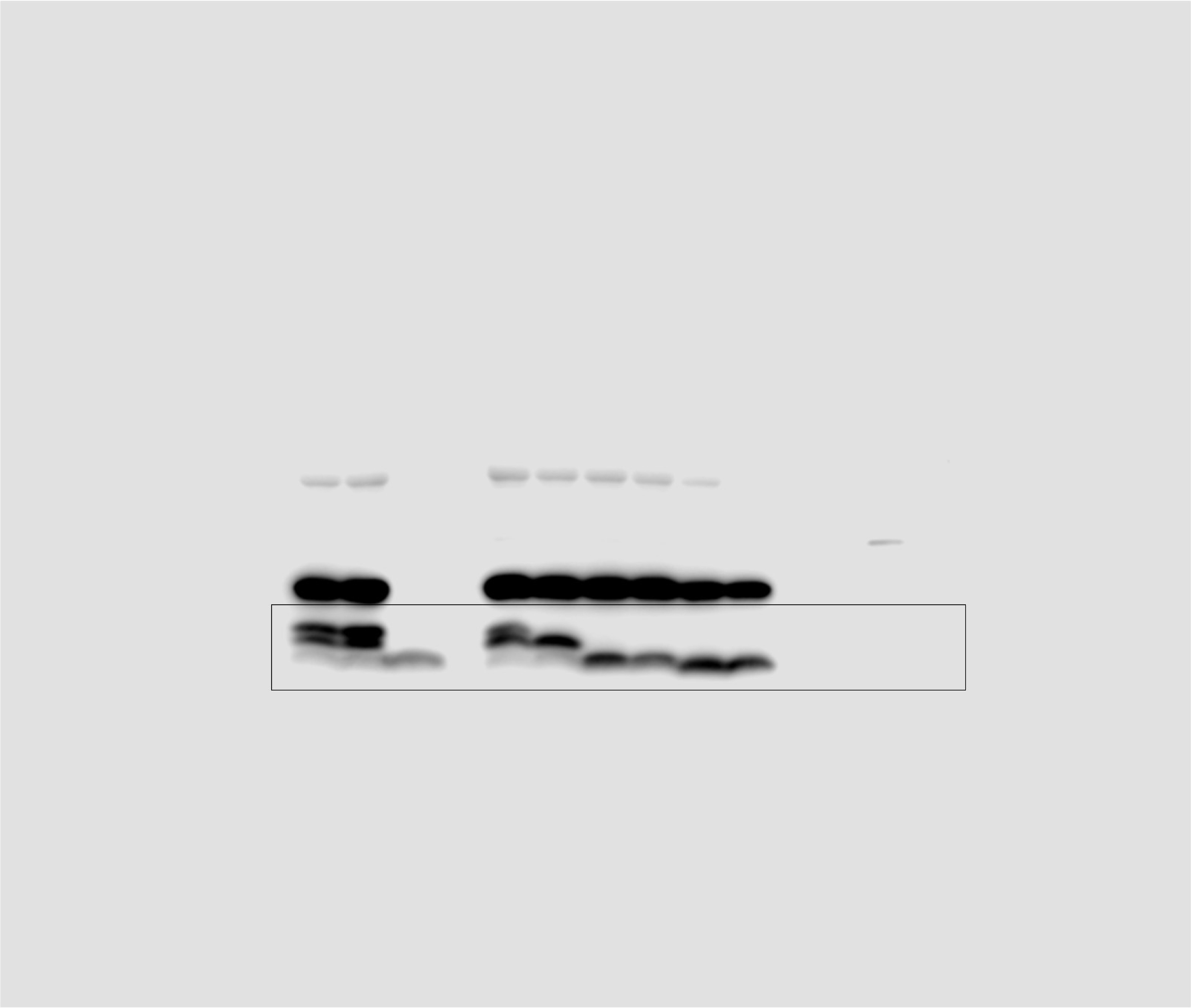

Cropped area for Figure 8D
$Ant1^{A114P,A123D}/Ant1^{A114P,A123D}$ mito, Tom20

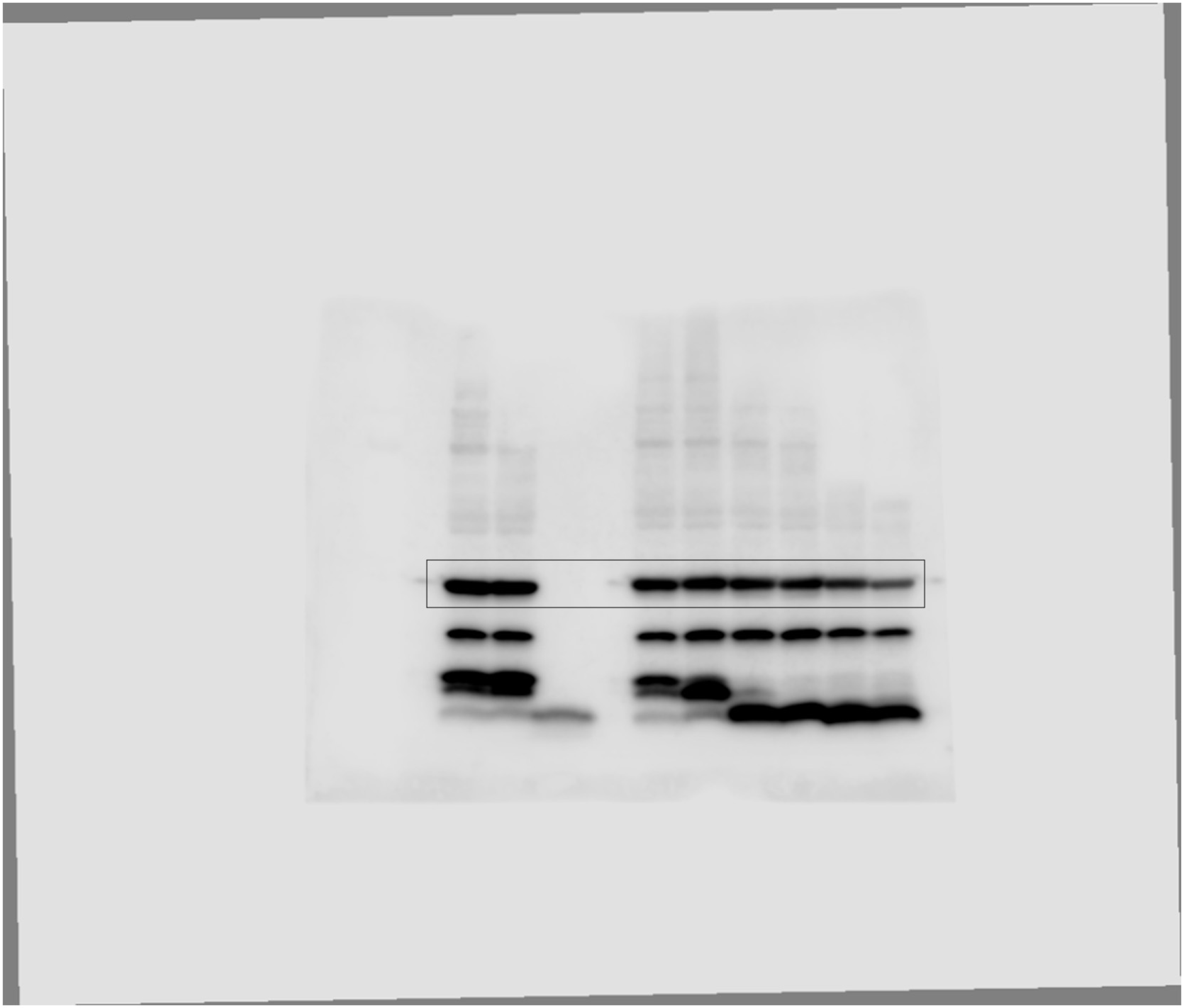

Cropped area for Figure 8D
Wild-type mito, Ant1

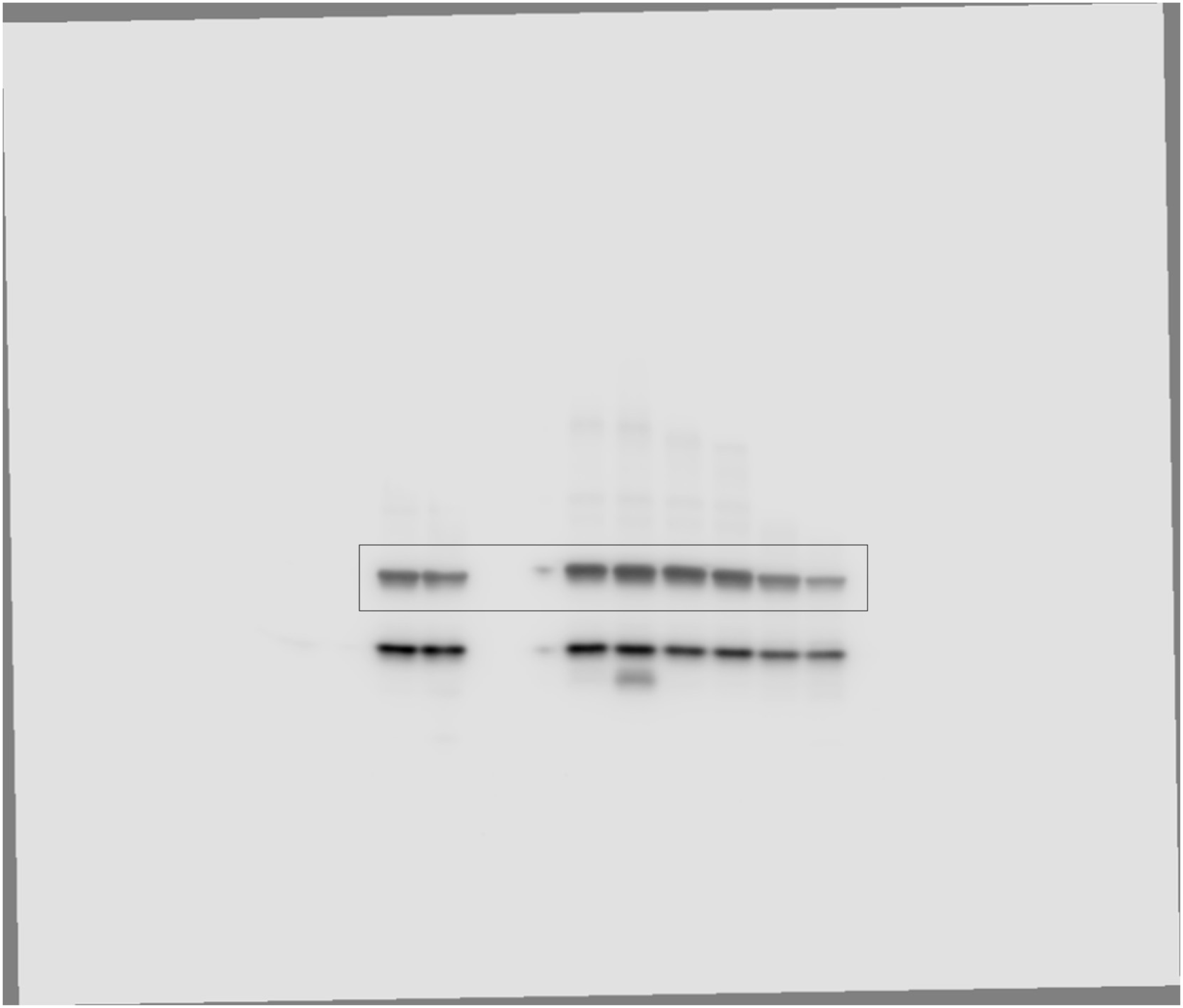

Cropped area for Figure 8D
Wild-type mito, MDH2

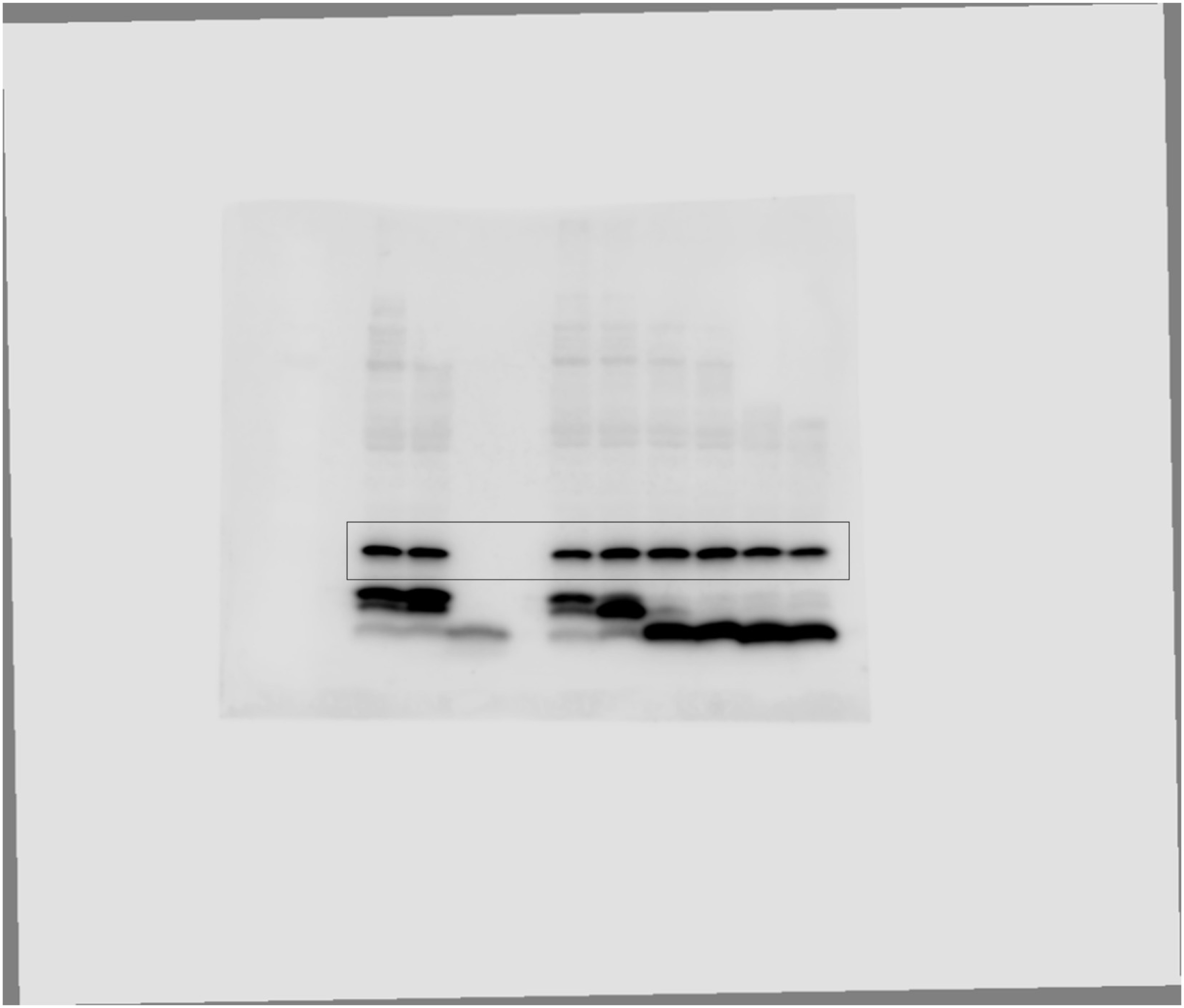

Area cropped for Figure 8D
Wild-type mito, SMAC

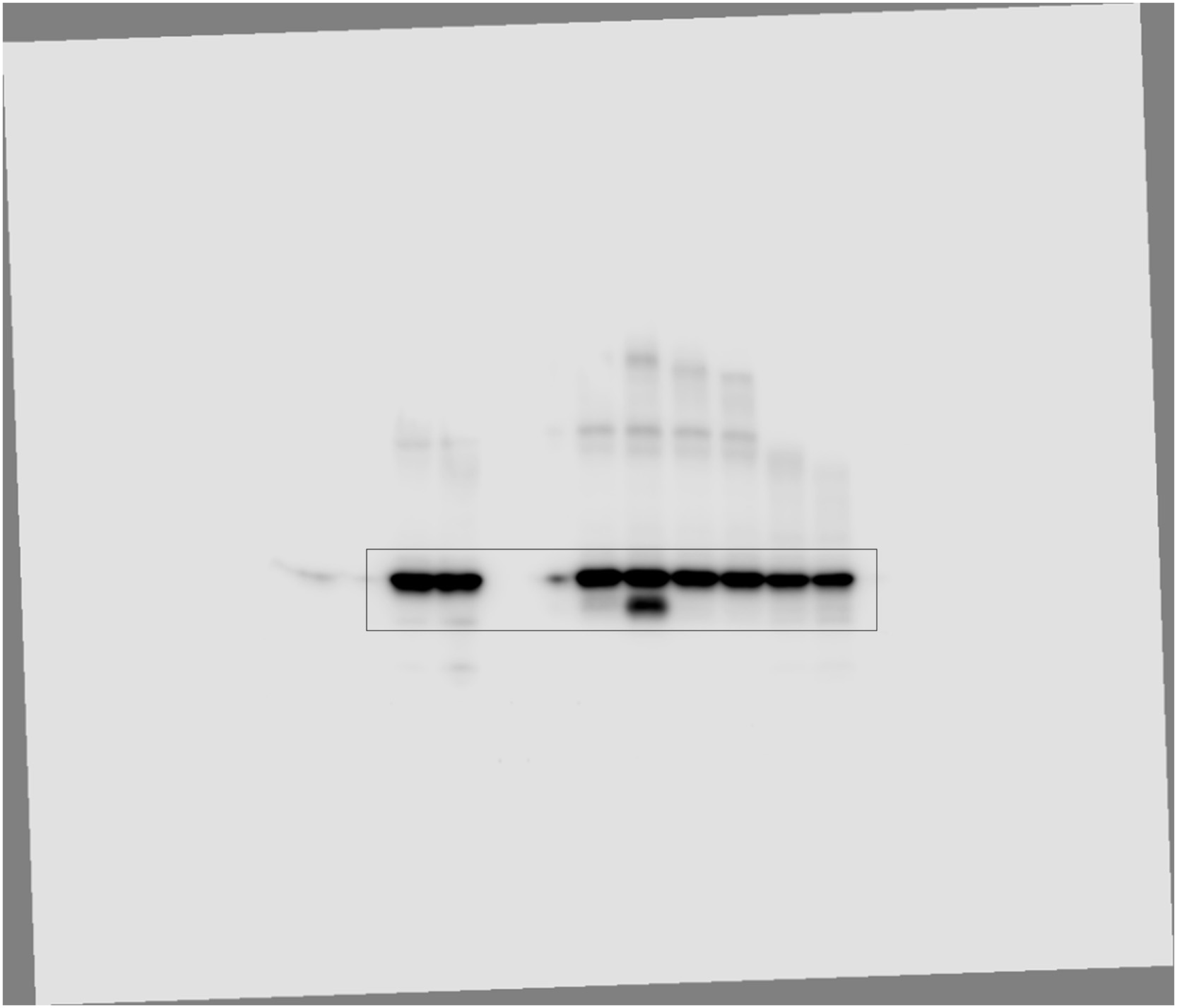

Cropped area for Figure 8D
Wild-type mito, Tim23

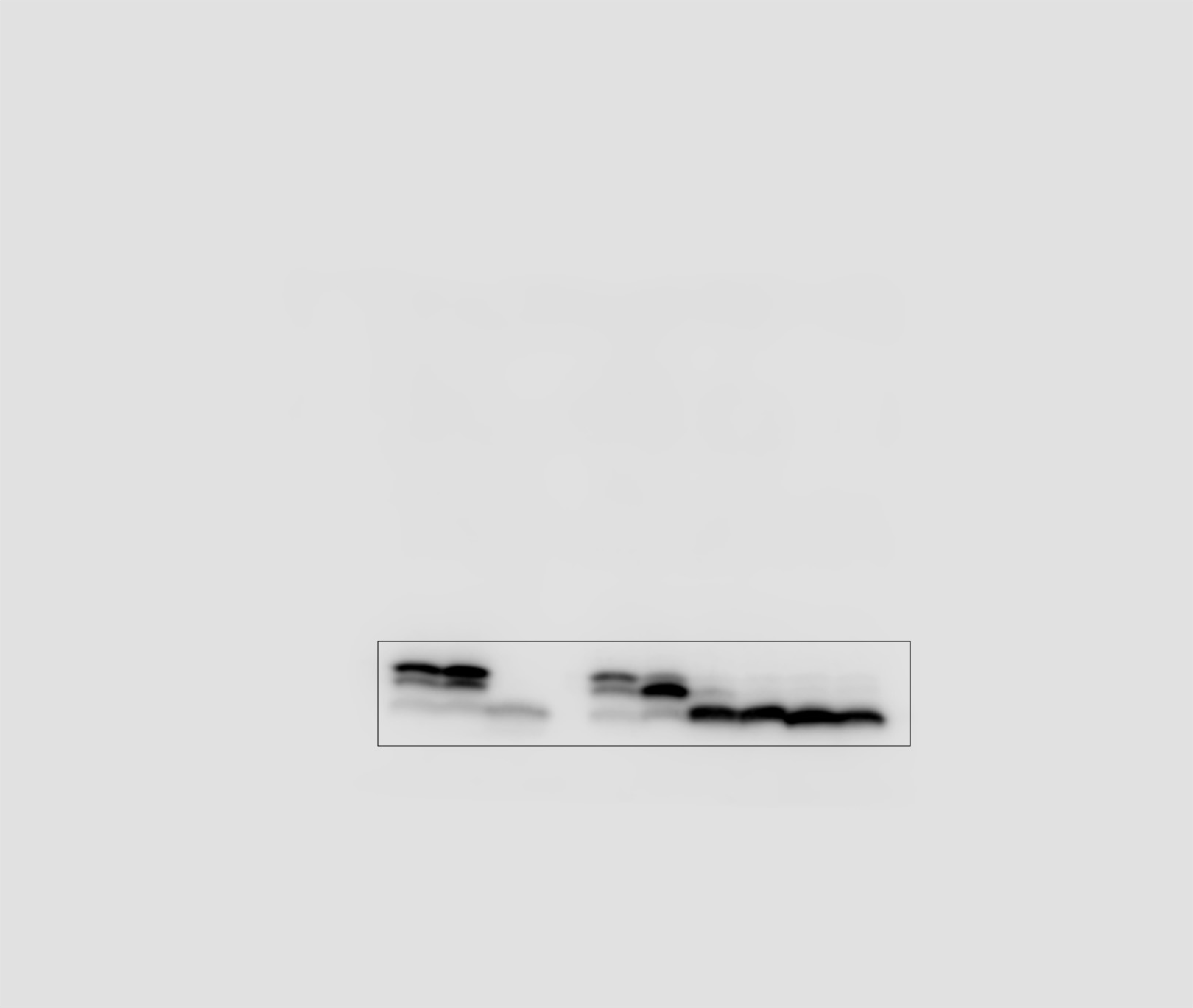

Cropped area for Figure 8D
Wild-type mito, TOM20
