# Supplementary material for: Mitochondrial protein import clogging as a mechanism of disease
Source: eLife. 2023 May 2;12:e84330. doi: 10.7554/eLife.84330 (PMC10208645; doi:10.7554/eLife.84330)
Supplement: Figure 8—figure supplement 1—source data 2. [file elife-84330-fig8-figsupp1-data2.zip › Figure 8-figure supplement 1-source data 1/Figure 8-figure supplement 1-source data annotated.pdf]

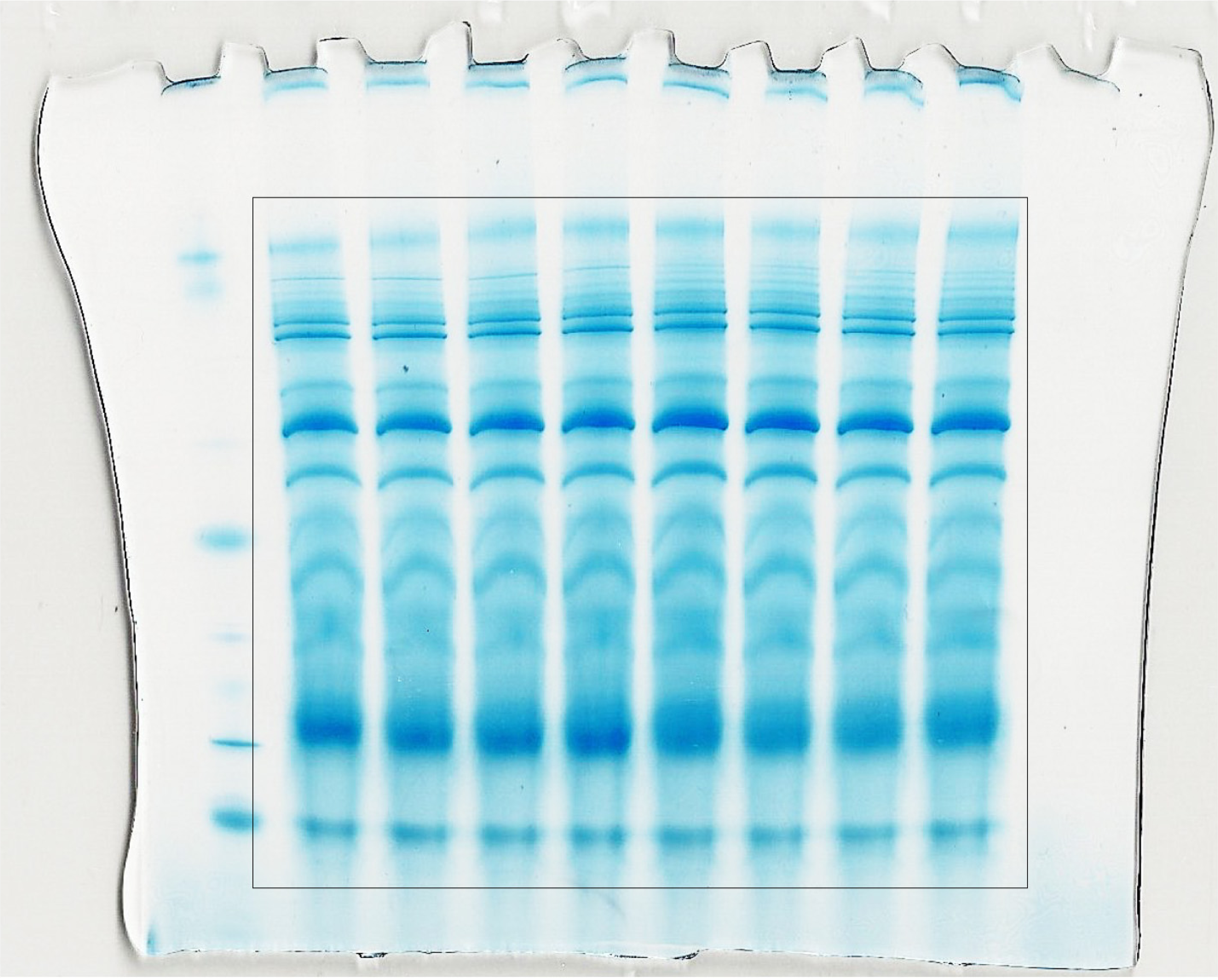

Cropped area for Figure 8-figure supplement 1C
Coomassie-stained BN-PAGE gel of mouse muscle mito

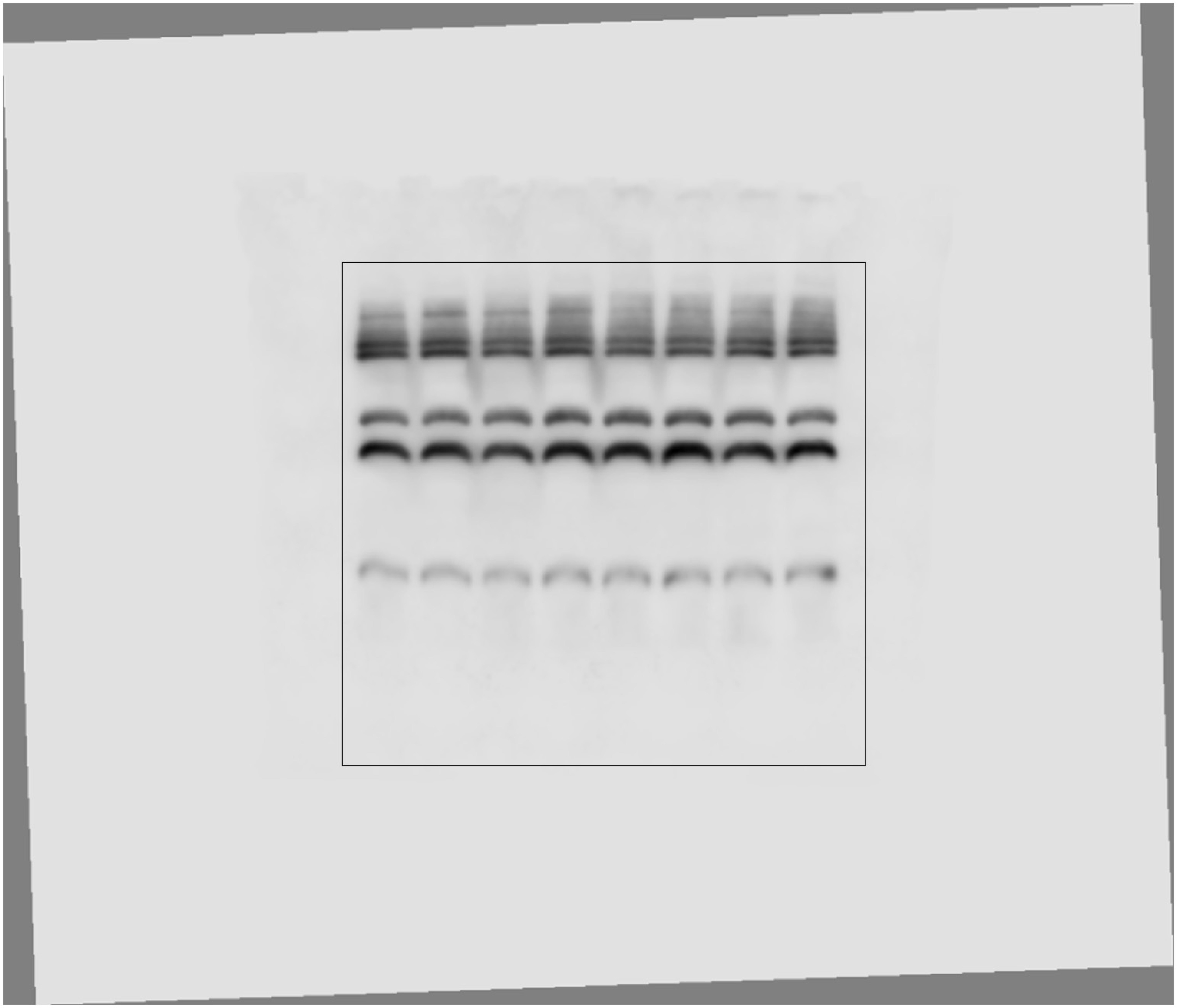

Cropped area for Figure 8-figure supplement 1D
BN-PAGE_Oxphos cocktail

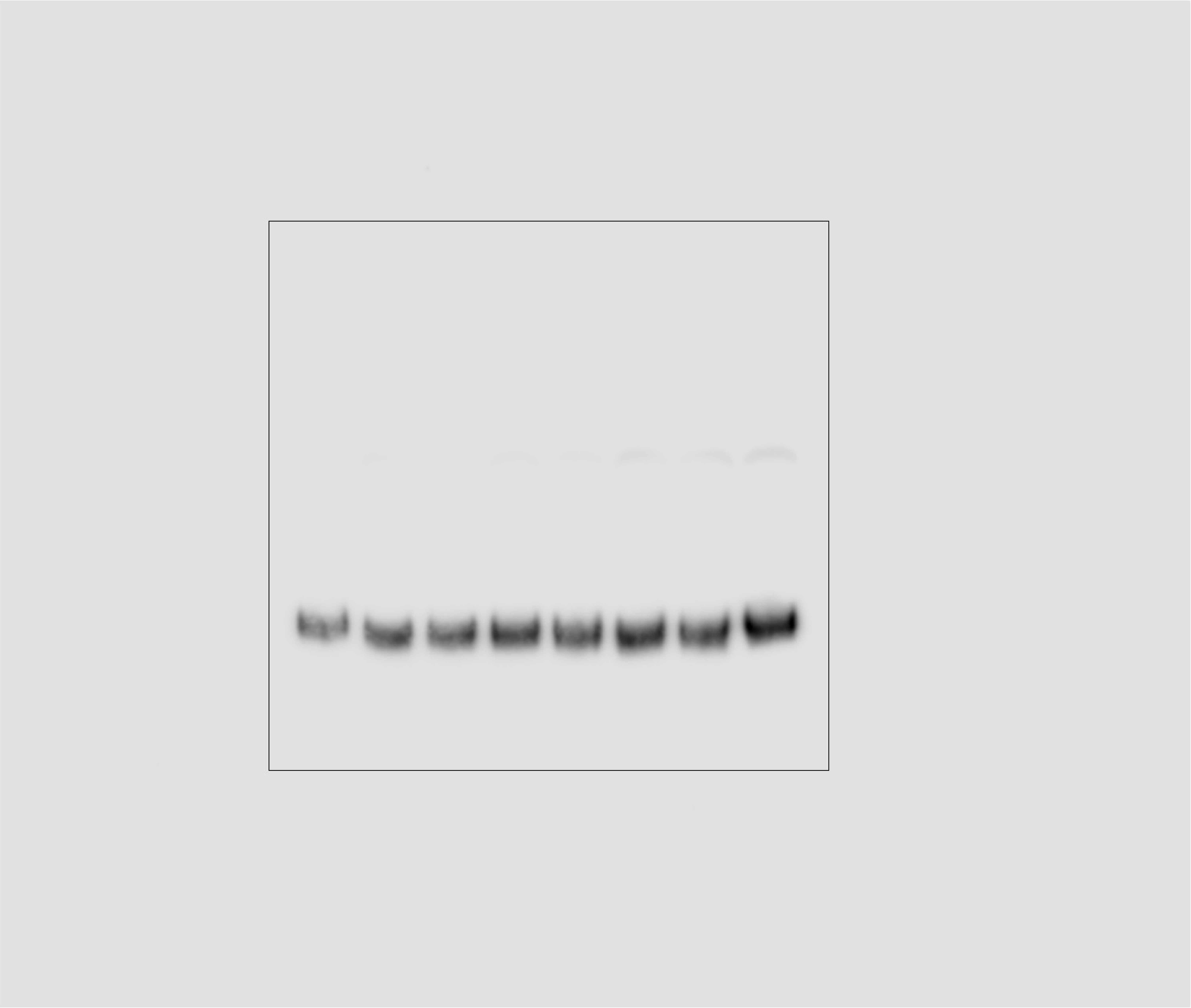

Cropped area for Figure 8-figure supplement 1E
BN-PAGE_Tim23

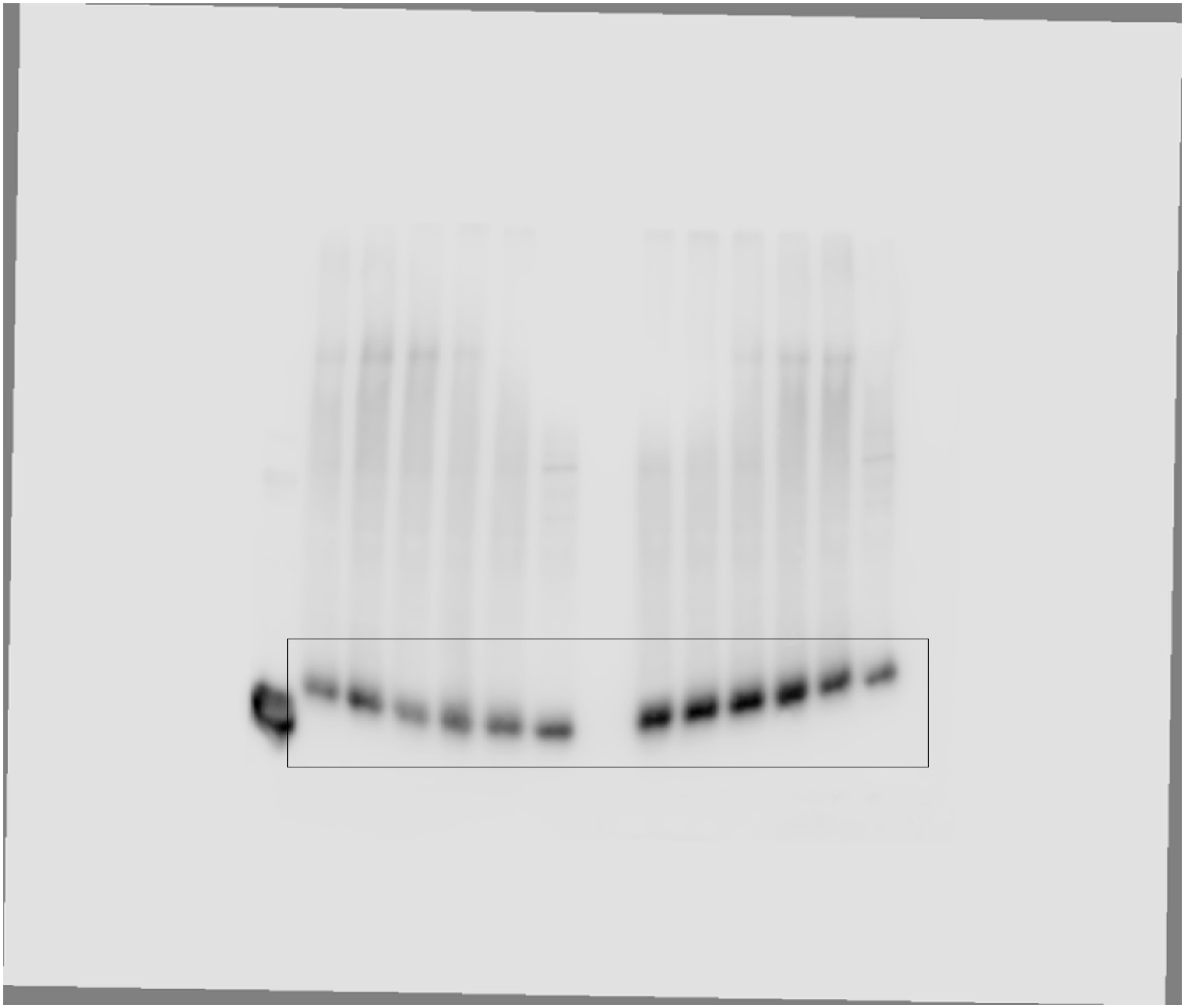

Cropped area for Figure 8-figure supplement 1I
Tim22

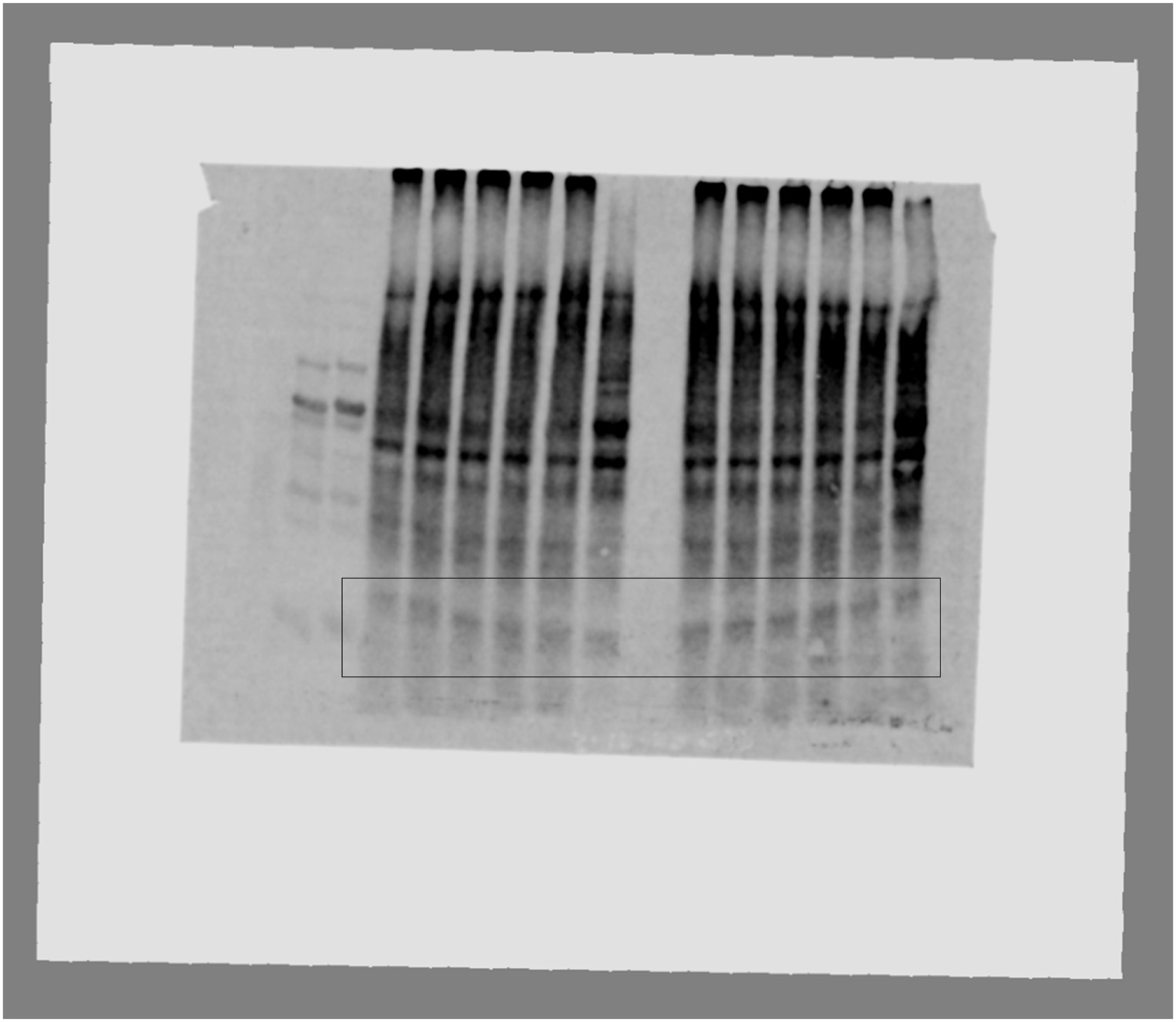

Cropped area for Figure 8-figure supplement 1I
TPS
