# Supplementary material for: Mitochondrial protein import clogging as a mechanism of disease
Source: eLife. 2023 May 2;12:e84330. doi: 10.7554/eLife.84330 (PMC10208645; doi:10.7554/eLife.84330)
Supplement: Figure 8—figure supplement 2—source data 2. [file elife-84330-fig8-figsupp2-data2.zip › Figure 8-figure supplement 2-source data 1/Figure 8-figure supplement 2-source data annotated.pdf]

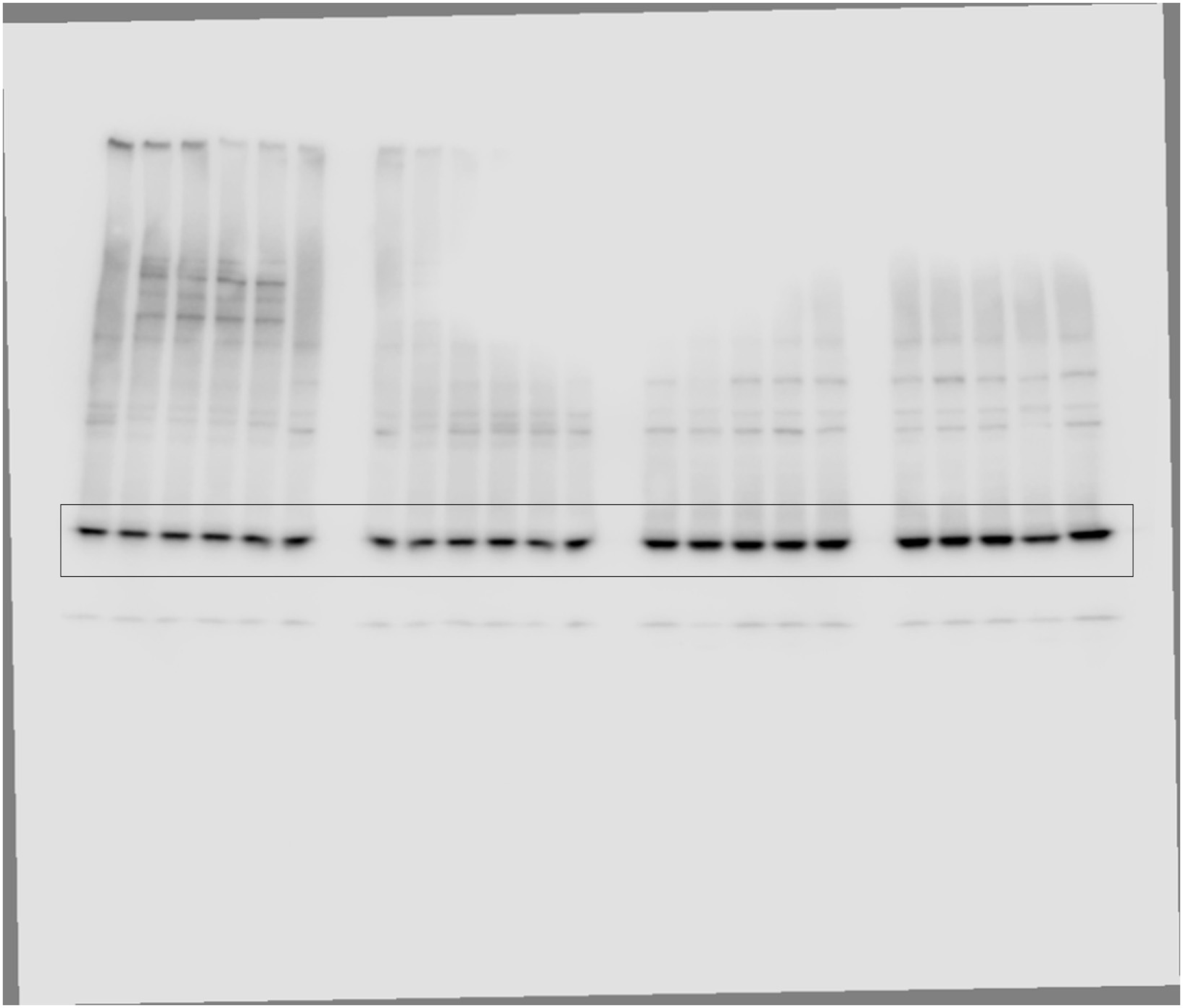

Cropped area for Figure 8-figure supplement 2A
eIF2alpha

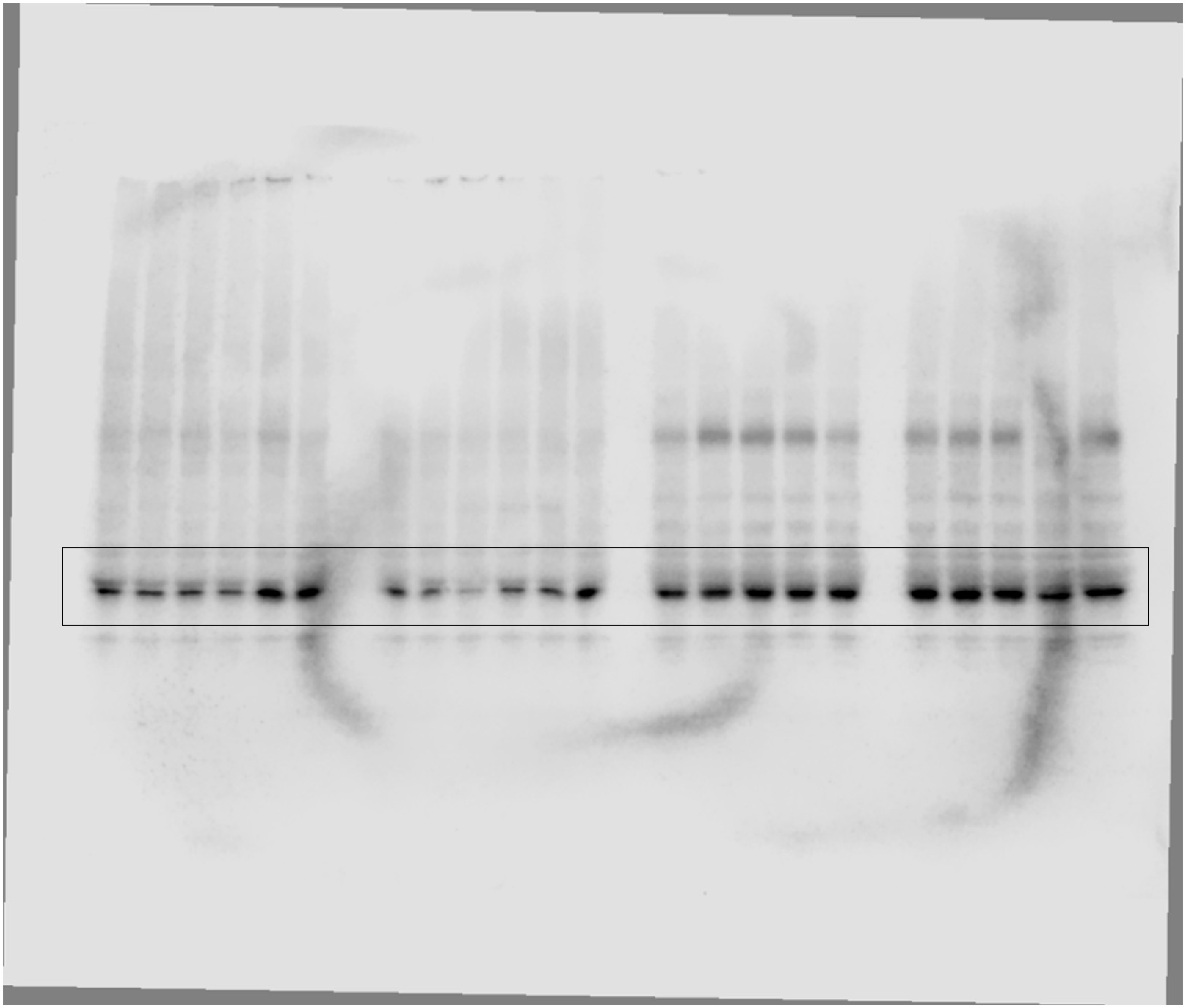

Cropped area for Figure 8-figure supplement 2A
phospho-eIF2alpha

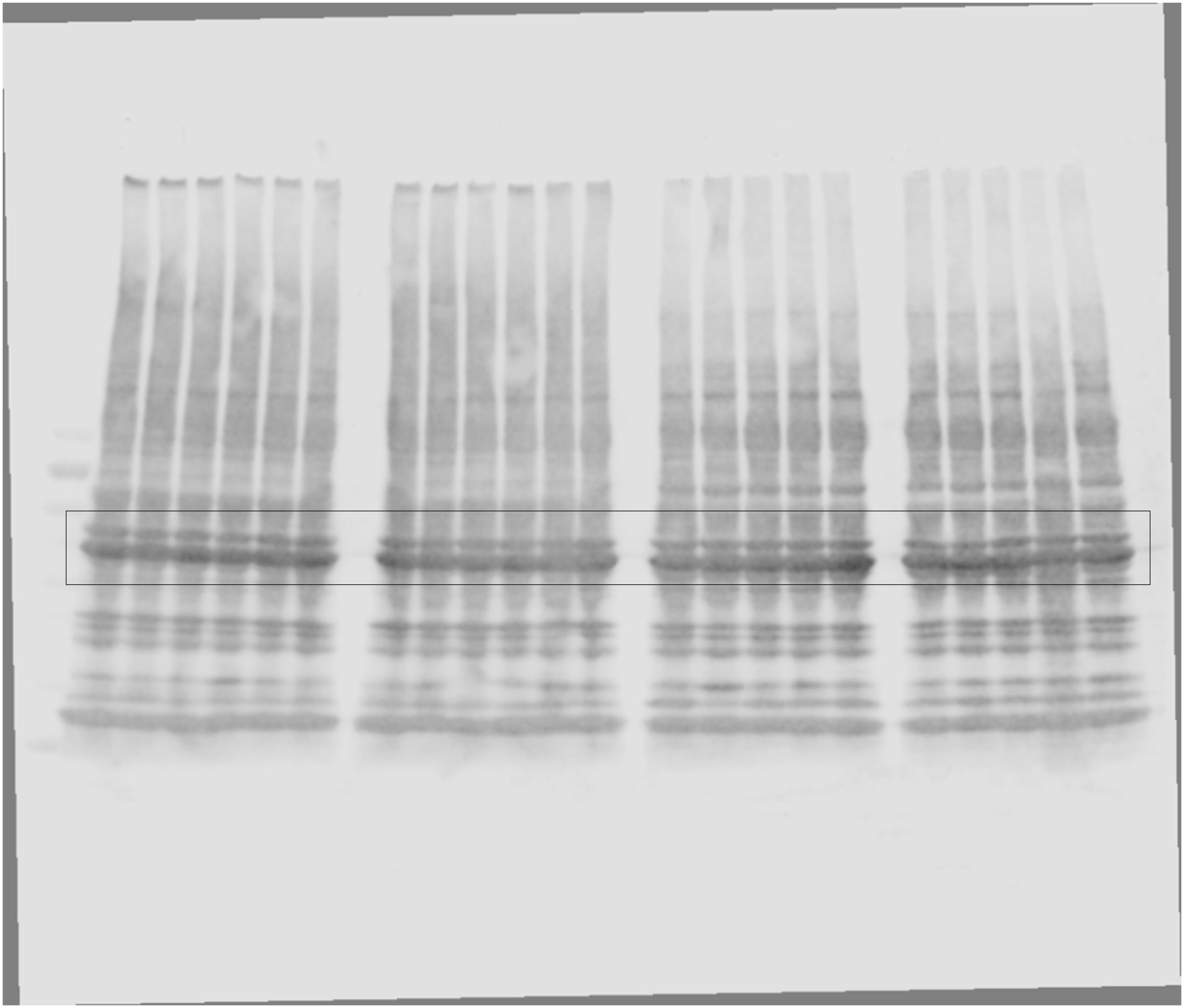

Cropped area for Figure 8-figure supplement 2A
Total protein stain_bottom

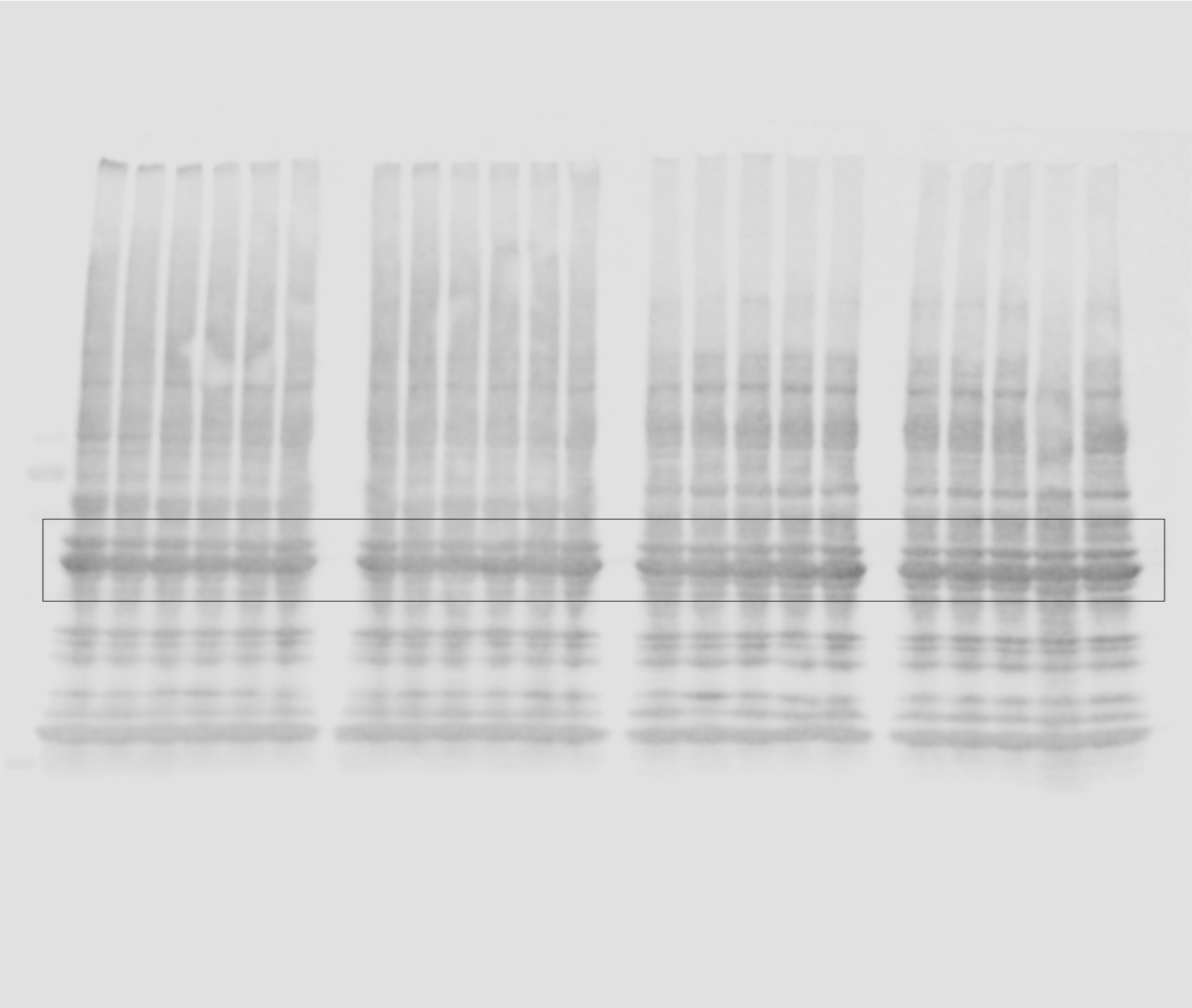

Cropped area for Figure 8-figure supplement 2A
Total protein stain_top
